# Synthesis of Dihydropyridine Spirocycles by Semi-Pinacol-Driven Dearomatization of Pyridines

**DOI:** 10.1021/acs.orglett.2c04095

**Published:** 2023-01-10

**Authors:** Joseph C. Abell, Christian P. Bold, Laia Vicens, Tom Jentsch, Noelia Velasco, Jasper L. Tyler, Robert N. Straker, Adam Noble, Varinder K. Aggarwal

**Affiliations:** † School of Chemistry, University of Bristol, Cantock’s Close, BristolBS8 1TS, U.K.; ‡ UCB Pharma, 208 Bath Road, SloughSL1 3WE, U.K.

## Abstract

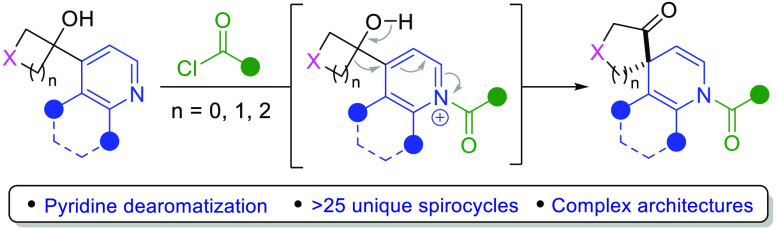

The identification of the beneficial pharmacokinetic properties of aza-spirocycles has led to the routine incorporation of these highly rigid and three-dimensional structures in pharmaceuticals. Herein, we report an operationally simple synthesis of spirocyclic dihydropyridines via an electrophile-induced dearomative semi-pinacol rearrangement of 4-(1′-hydroxycyclobutyl)pyridines. The various points for diversification of the spirocyclization precursors, as well as the synthetic utility of the amine and ketone functionalities in the products, provide the potential to rapidly assemble medicinally relevant spirocycles.

Nitrogen heterocycles are highly prevalent in FDA-approved drug molecules, with the piperidine ring system representing the most common saturated N-heterocycle in marketed pharmaceuticals.^1,2^ Incorporating piperidine and dihydropyridine rings into spirocyclic systems provides the potential to generate inherently rigid three-dimensional structures that retain the beneficial pharmacokinetic properties of N-heterocycles.^3,4^ The high fraction of sp^3^-hybridized carbons (*F*_sp^3^_), structural novelty, and limited conformational flexibility are predicted to give such structures a greater chance of achieving clinical success in drug discovery programs.^5^ Accordingly, such heterocyclic moieties have been incorporated into numerous approved drugs and promising clinical leads (Figure 1a).^6−10^

**Figure 1:**
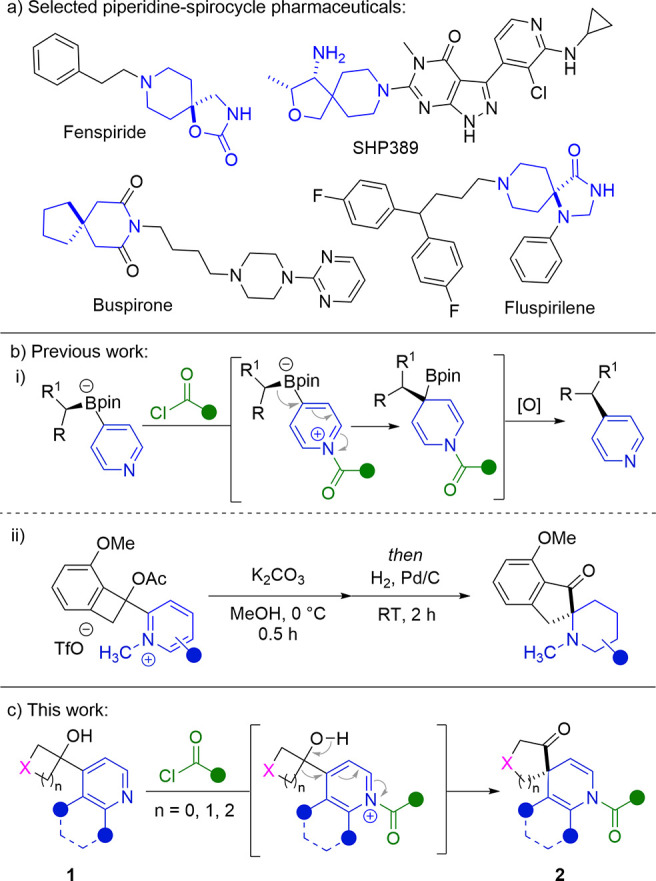
(a) Piperidine-based spirocyclic pharmaceuticals. (b) Previous work: (i) dearomative 1,2-boronate rearrangement and (ii) alkylpyridinium-induced semi-pinacol rearrangement. (c) Electrophile-induced dearomative semi-pinacol ring expansion.

Despite their potential, synthetic methods for accessing piperidine spirocycles are significantly underdeveloped.^11^ Syntheses commonly employ relatively expensive N-protected piperidin-4-ones as precursors^6^ and require lengthy cyclization sequences to generate the spirocenter. Strategies that have been used to induce ring closure include acylation,^12^ alkylation,^7,13^ ring-closing metathesis,^13^ and cycloaddition reactions.^14^ One of the few available methods for accessing dihydropyridine spirocycles involves the condensation of amines with 1,5-dialdehydes (glutaraldehydes) bearing a carbocycle in the backbone.^15^ However, the lengthy multistep syntheses of the glutaraldehyde precursors somewhat limit the appeal of this approach.

Dearomatization reactions in which feedstock aromatic systems are converted to more complex sp^3^ rich structures represent a highly desirable strategy for accessing nonplanar scaffolds such as spirocycles.^16^ Previous publications from the group of Ready and our own have reported the generation of N-heterocycle boronate complexes and subsequent 1,2-migration reactions facilitated by N-acylation to provide dihydropyridyl boronic esters (Figure 1b, i).^17,18^ In 2011, Hayashi and co-workers demonstrated the dearomative spirocyclization of *N*-alkylpyridinium benzocyclobutenol derivatives (Figure 1b, ii).^19^ In this case, a semi-pinacol rearrangement is triggered by alcohol deacetylation, and the piperidine spirocycle is obtained following hydrogenation.

We envisaged a more general and straightforward approach in which easy-to-access hydroxycyclobutylpyridines (**1**) could undergo an electrophile-induced dearomatizing semi-pinacol ring expansion to deliver dihydropyridine spirocycles (Figure 1c, **2**).^20^ We predicted that employing N-acylation activators should render these compounds isolable, giving the potential to either directly access these atypical structures or perform hydrogenation to the corresponding piperidine spirocycles.^19^ Herein, we report the successful synthesis of a broad range of dihydropyridine spirocycles through a dearomatizing semi-pinacol rearrangement, driven by the selective N-acylation of hydroxycyclobutylpyridines. The resulting products bearing a ketone and protected secondary amine provide the potential for bidirectional elaboration to assemble medicinally relevant spirocyclic scaffolds.

We began by synthesizing the requisite 4-hydroxycyclobutylpyridine starting materials (Scheme 1). This was achieved in a single step via the 1,2-addition of metalated pyridines, generated by deprotonation or halogen–metal exchange, to a variety of cyclic ketones.^21^

**Scheme 1:**
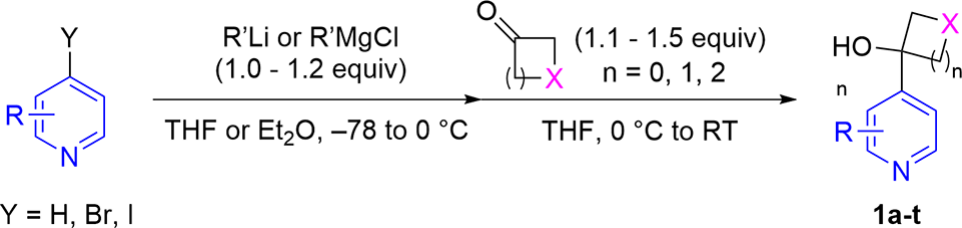
Synthesis of Hydroxycycloalkylpyridines (**1**)

With hydroxycyclobutylpyridine substrates in hand, we set about investigating the dearomative semi-pinacol spirocyclization reaction. Fluorine-labeled **1h** was selected as the model substrate allowing the use of ^19^F NMR to monitor reaction progress (Table 1). An initial screen of acylating agents showed that the combination of 2,2,2-trichloroethyl chloroformate (TrocCl) in chloroform allowed access to desired product **2** in 52% isolated yield (Table 1, entry 1). The presence of diisopropylethylamine (DIPEA) was required to sequester the HCl generated, preventing yield-limiting precipitation of the pyridine starting material hydrochloride salt. It must also be noted that under these conditions, the direct acylation of the hydroxy group was observed, resulting in minor product **3**. Although the use of the more sterically hindered acylating agent 1,1-dimethyl-2,2,2-trichloroethyl chloroformate (TCBocCl) successfully suppressed acylation of the tertiary alcohol (Table 1, entry 2), we were keen to explore other more common activators. We therefore turned to *tert*-butyloxycarbonyl as a hindered, commonly used, yet easily cleaved protecting group. Unfortunately, the application of di-*tert*-butyl dicarbonate (Boc_2_O) in chloroform resulted in a very slow reaction, requiring 6–7 days to achieve full conversion at reflux (Table 1, entries 3 and 4). Surprisingly, the inclusion of a substoichiometric amount of 4-(dimethylamino)pyridine (DMAP) was found to completely reverse the selectivity of the acylation, exclusively generating O-acylation product **3** (Table 1, entry 5). Finally, a screen of high-boiling point solvents showed that employing acetonitrile with 5 equiv of Boc_2_O gave an NMR yield of 85% after 24 h (Table 1, entries 6 and 7).^22^ While degradation of the dihydropyridine spirocycles was not directly observed, the discrepancy between the NMR yield and isolated yield may hint at minor levels of instability toward chromatographic purification.

**Table 1:**
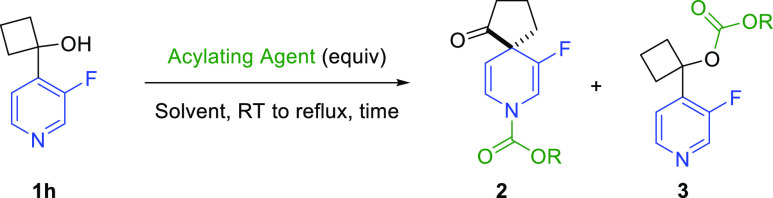
Optimization Studies

entry	acylating agent (equiv)	solvent, time	yield of **2** (%)^a^	yield of **3** (%)^a^
1^b^	TrocCl (1.5)	CHCl_3_, 18 h	60 (52)	10 (9)
2^b^	TCBocCl (1.5)	CHCl_3_, 18 h	85	0
3	Boc_2_O (1.5)	CHCl_3_, 7 days	81	0
4	Boc_2_O (5.0)	CHCl_3_, 6 days	88	0
5^c^	Boc_2_O (1.5)	CHCl_3_, 18 h	0	96
6	Boc_2_O (1.5)	MeCN, 48 h	44	0
7	Boc_2_O (5.0)	MeCN, 24 h	85 (68)	0

a Yield determined by ^19^F NMR. Isolated yields in parentheses.

b Reaction performed at room temperature with DIPEA (1.5 equiv).

c With DMAP (0.3 equiv).

Having established the optimal conditions, we then investigated the scope of the reaction (Scheme 2). Cyclopentanone spirocycle **2a** was obtained in an excellent yield of 96% from 4-(1-hydroxycyclobutyl)pyridine and isolated in comparable yield when prepared on a gram scale. The spirocyclization reaction also worked for the expansion of three- and five-membered cyclic alcohols to form products **2b** and **2c** in good yields. However, identical conditions were unsuccessful in generating the corresponding cycloheptanone product **2d**. In addition, starting materials bearing heteroatoms in the cyclobutane ring were amenable to this transformation, providing **2e** and **2f** in 78% and 64% yields, respectively. Employing an unsymmetrical 4-hydroxycyclobutylpyridine gives two possible products depending on the carbon–carbon bond that undergoes migration. Pleasingly, in the case of tricyclic product **2g**, a single regioisomer was obtained, demonstrating the greater migratory aptitude of a tertiary carbon over a secondary carbon.^23^ We then explored the effect of substituents and functional handles on the pyridine ring. It was observed that 3-substituted pyridines were well tolerated in this transformation, allowing the generation of dihydropyridine spirocycles bearing halogen (**2h** and **2i**), diisopropylamide (**2j**), and aromatic groups (**2k**). Due to the increase in steric hindrance around the pyridine nitrogen, the formation of 2-methyl substrate **2l** required the addition of an additional 5 equiv of Boc_2_O to achieve full conversion. It was noted that the addition of the acylating agent in installments over the course of the reaction was superior to increasing the initial reaction stoichiometry, which was attributed to the Boc_2_O gradually decomposing under the reaction conditions. Employing the analogous 2-phenylpyridine starting material was found to show no conversion under the reaction conditions. To overcome this, the more reactive acylating agent 2,2,2-trichloro-1,1-dimethylethyl chloroformate (TCBocCl) was utilized and enabled spirocycle **2m** to be produced in 77% yield. This effect was also observed upon extending the protocol to the dearomatization of quinolines. With Boc_2_O, competing O-acylation was found to dominate at increased temperatures and increasing the number of equivalents of the electrophile had a negligible impact on product formation. However, the use of TCBocCl enabled spirocyclization products **2n–2s** containing chloro, bromo, fluoro, and methoxy functionalities to be accessed in synthetically useful yields. The limit of reactivity was found upon employing 2-fluoropyridine starting materials, for which the deactivating effect of the fluorine atom prevented any observable **2t** formation with either Boc_2_O or TCBocCl.

**Scheme 2:**
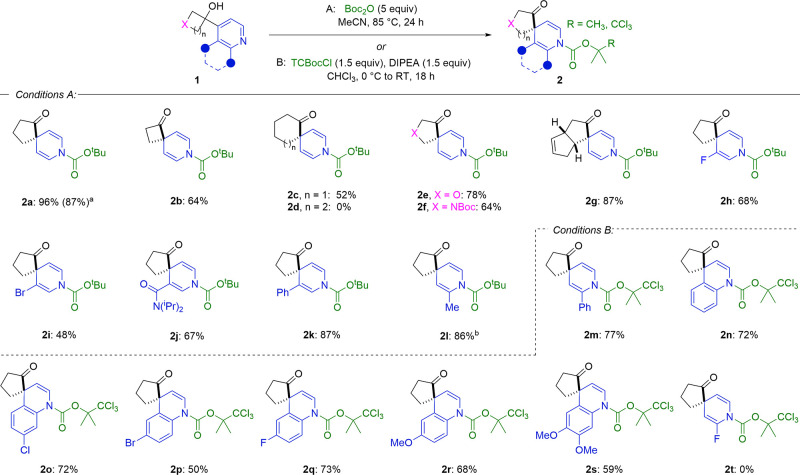
Substrate Scope for the Dearomative Spirocyclization Reaction Reaction performed with 6.7 mmol of **1a**. An additional 5.0 equiv of Boc_2_O was added after 12 h of reaction time. All reactions were performed on a 0.25 mmol scale. Isolated yields are given.

Next, the scope of activating agents for enacting the spirocyclization was investigated (Scheme 3). Chloroformates were generally successful in achieving the desired reactivity without the need for increased temperatures or a large excess of an activating agent. The ratio of the spirocycle to the O-acylation product varied from reagent to reagent, broadly correlating with the relative steric bulk of the electrophile. Methyl chloroformate and TrocCl gave the corresponding spirocycle (**2aa** and **2ab**) and O-acylation (**3aa** and **3ab**) products in an approximate 2:1 ratio. Minor competing O-acylation was also observed for allyl chloroformate and benzyl chloroformate, which provided the desired dihydropyridine spirocycles (**2ac** and **2ad**) in 68% and 71% yields, respectively. However, no O-acylation was observed using the more hindered TCBocCl or 9*H*-fluorenylmethyl chloroformate (FmocCl), which provided spirocycles **2ae** and **2af**(24) in high yields. Beyond chloroformates, pivaloyl chloride was demonstrated to be a suitable acylating agent for the reaction, giving spirocyclic amide **2ag** in a modest yield. In addition, triflic anhydride and tosyl chloride were found to promote spirocyclization under the same reaction conditions, providing sulfonamides **2ah** and **2ai**. The yield of product formation was noticeably lower with nosyl chloride (**2aj**) due to a lack of solubility of this reagent in chloroform. It must also be noted that the ability to install a broad range of protecting groups in the spirocyclic products provides the opportunity to deprotect the amine functionality orthogonal to any sensitive groups present in the molecule.^25^

**Scheme 3:**
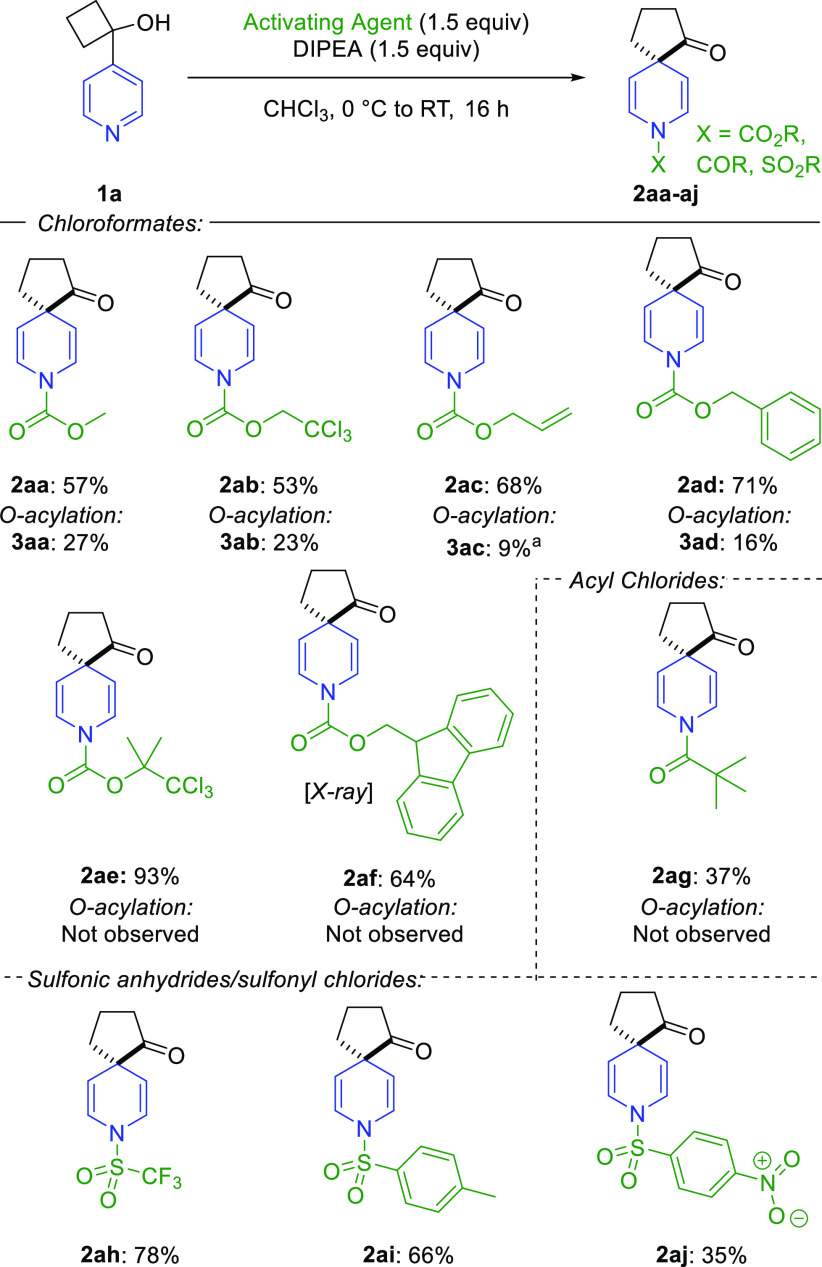
Activator Scope for Dearomative Spirocyclization Reaction NMR yield calculated using using dibromomethane as an internal standard. All reactions were performed on a 0.25 mmol scale. Isolated yields are given.

Finally, dihydropyridine spirocycles **2a** and **2k** were efficiently converted to highly valuable piperidine spirocycles (**4a** and **4b**) by reduction with hydrogen gas over palladium on carbon (Scheme 4). In the case of **2k**, hydrogenation proceeded with excellent diastereoselectivity, highlighting the synthetic utility of this approach. This transformation demonstrates the utility of this dearomatization procedure, allowing access to both dihydropyridine and piperidine spirocyclic scaffolds.

**Scheme 4:**
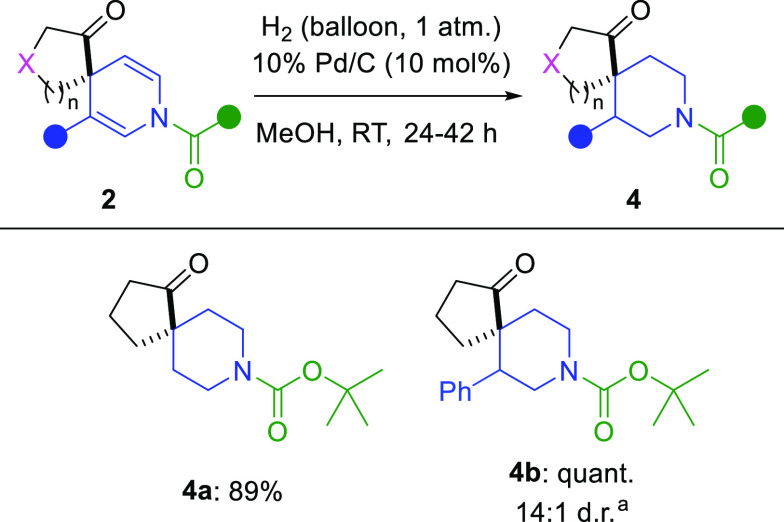
Hydrogenation of Spirocyclic Dihydropyridines Identity of the major diastereomer unknown. Isolated yields are given.

In conclusion, we have developed a highly efficient protocol for the dearomatizing spirocyclization of 4-(1′-hydroxycycloalkyl)pyridines. The starting materials, accessed in a single step from commercially available pyridine precursors, were observed to undergo semi-pinacol ring expansion reactions upon N-acylation. The applicability of this strategy was demonstrated through the synthesis of a diverse family of dihydropyridine spirocycles in which both the scope of the pyridine precursors and the activating agent were explored. Finally, the hydrogenation of the dihydropyridine spirocyclic products to their corresponding piperidine analogues was demonstrated. The various points for diversification of the spirocyclization precursors (ring size and pyridine substitution pattern), as well as the synthetic utility of the secondary amine and ketone functionalities in the products, provide the potential to rapidly assemble medicinally relevant spirocyclic scaffolds.

## Data Availability

The data underlying this study are available in the published article and its Supporting Information.

## Supporting Information Available

Experimental procedures, characterization data, and ^1^H and ^13^C NMR spectra for all novel compounds (PDF)
