# Supplementary material for: Synthesis of Dihydropyridine Spirocycles by Semi-Pinacol-Driven Dearomatization of Pyridines
Source: Org Lett. 2023 Jan 10;25(2):400–4. doi: 10.1021/acs.orglett.2c04095 (PMC9872164; doi:10.1021/acs.orglett.2c04095)
Supplement: Supplementary file 1 — ol2c04095_si_001.pdf [file ol2c04095_si_001.pdf]

SUPPORTING INFORMATION

Synthesis of Dihydropyridine Spirocycles by Semi-Pinacol Driven Dearomatization of Pyridines

Joseph C. Abell,[a] Christian P. Bold,[a] Laia Vicens,[a] Tom Jentsch,[a] Noelia Velasco,[a] Jasper L. Tyler,[a] Robert N. Straker,[b] Adam Noble,[a] and Varinder K. Aggarwal*[a]

[a] School of Chemistry, University of Bristol, Cantock's Close, Bristol BS8 1TS, United Kingdom

[b] UCB Pharma, 208 Bath Road, Slough SL1 3WE, United Kingdom

*e-mail: v.aggarwal@bristol.ac.uk

TABLE OF CONTENTS

1. MATERIALS AND GENERAL METHODS S3
1.1. Glassware, Solvents and Reagents S3
1.2. Chromatography and Instrumentation S3
1.3. Naming of Compounds S3
2. EXPERIMENTAL DATA S4
2.1. Optimization Studies S4
2.1.1. Reaction optimization with TrocCl S4
2.1.2. Screen of acylating agents S5
2.1.3. Reaction optimization with Boc_2O S6
2.2. Synthesis of Non-Literature Known Precursors S7
2.2.1. 6-fluoro-4-iodoquinoline (SI-1) S7
2.2.2. 4-iodo-6-methoxyquinoline (SI-2) S7
2.2.3. 4-iodo-6,7-dimethoxyquinoline (SI-3) S8
2.3. Synthesis of hydroxycycloalkylpyridines (1) S9
2.3.1. 1-(pyridin-4-yl)cyclobutan-1-ol (1a) S9
2.3.2. 1-(pyridin-4-yl)cyclopropan-1-ol (1b) S9
2.3.3. 1-(pyridin-4-yl)cyclopentan-1-ol (1c) S10
2.3.4. 1-(pyridin-4-yl)cyclohexan-1-ol (1d) S10
2.3.5. 3-(pyridin-4-yl)oxetan-3-ol (1e) S11
2.3.6. *tert*-butyl 3-hydroxy-3-(pyridin-4-yl)azetidine-1-carboxylate (1f) S11
2.3.7. (1*R*,5*S*)-6-(pyridin-4-yl)bicyclo[3.2.0]hept-2-en-6-ol (1g) S12
2.3.8. 1-(3-fluoropyridin-4-yl)cyclobutan-1-ol (1h) S12
2.3.9. 1-(3-bromopyridin-4-yl)cyclobutan-1-ol (1i) S13
2.3.10. 4-(1-hydroxycyclobutyl)-*N*,*N*-diisopropylnicotinamide (1j) S13
2.3.11. 1-(3-phenylpyridin-4-yl)cyclobutan-1-ol (1k) S14
2.3.12. 1-(2-methylpyridin-4-yl)cyclobutan-1-ol (1l) S15
2.3.13. 1-(2-phenylpyridin-4-yl)cyclobutan-1-ol (1m) S15
2.3.14. 1-(quinolin-4-yl)cyclobutan-1-ol (1n) S16
2.3.15. 1-(7-chloroquinolin-4-yl)cyclobutan-1-ol (1o) S16
2.3.16. 1-(6-bromoquinolin-4-yl)cyclobutan-1-ol (1p) S17
2.3.17. 1-(6-fluoroquinolin-4-yl)cyclobutan-1-ol (1q) S17
2.3.18. 1-(6-methoxyquinolin-4-yl)cyclobutan-1-ol (1r) S18
2.3.19. 1-(6,7-dimethoxyquinolin-4-yl)cyclobutan-1-ol (1s) S18
2.3.20. 1-(2-fluoropyridin-4-yl)cyclobutan-1-ol (1t) S19
2.4. Substrate Scope for the Dearomative Spirocyclization Reaction S20
2.4.1. General Procedure A (GP1) S20
2.4.2. General Procedure B (GP2) S20
2.4.3. *tert*-butyl 1-oxo-8-azaspiro[4.5]deca-6,9-diene-8-carboxylate (2a) S21
2.4.4. *tert*-butyl 1-oxo-7-azaspiro[3.5]nona-5,8-diene-7-carboxylate (2b) S21
2.4.5. *tert*-butyl 7-oxo-3-azaspiro[5.5]undeca-1,4-diene-3-carboxylate (2c) S22

2.4.6. *tert*-butyl 4-oxo-2-oxa-8-azaspiro[4.5]deca-6,9-diene-8-carboxylate (2e) S22
2.4.7. di-*tert*-butyl 4-oxo-2,8-diazaspiro[4.5]deca-6,9-diene-2,8-dicarboxylate (2f) S22
2.4.8. *tert*-butyl-2-oxo-3,3a,6,6a-tetrahydro-1'*H*,2*H*-spiro[pentalene-1,4'-pyridine]-1'-carboxylate (2g) S23
2.4.9. *tert*-butyl 6-fluoro-1-oxo-8-azaspiro[4.5]deca-6,9-diene-8-carboxylate (2h) S23
2.4.10. *tert*-butyl 6-bromo-1-oxo-8-azaspiro[4.5]deca-6,9-diene-8-carboxylate (2i) S23
2.4.11. *tert*-butyl 6-(diisopropylcarbamoyl)-1-oxo-8-azaspiro[4.5]deca-6,9-diene-8-carboxylate (2j) S24
2.4.12. *tert*-butyl 1-oxo-6-phenyl-8-azaspiro[4.5]deca-6,9-diene-8-carboxylate (2k) S24
2.4.13. *tert*-butyl 7-methyl-1-oxo-8-azaspiro[4.5]deca-6,9-diene-8-carboxylate (2l) S25
2.4.14. 1,1,1-trichloro-2-methylpropan-2-yl 1-oxo-7-phenyl-8-azaspiro[4.5]deca-6,9-diene-8-carboxylate (2m) S25
2.4.15. 1,1,1-trichloro-2-methylpropan-2-yl 2-oxo-1'*H*-spiro[cyclopentane-1,4'-quinoline]-1'-carboxylate (2n) S25
2.4.16. 1,1,1-trichloro-2-methylpropan-2-yl 7'-chloro-2-oxo-1'*H*-spiro[cyclopentane-1,4'-quinoline]-1'-carboxylate (2o) S26
2.4.17. 1,1,1-trichloro-2-methylpropan-2-yl 6'-bromo-2-oxo-1'*H*-spiro[cyclopentane-1,4'-quinoline]-1'-carboxylate (2p) S26
2.4.18. 1,1,1-trichloro-2-methylpropan-2-yl 6'-fluoro-2-oxo-1'*H*-spiro[cyclopentane-1,4'-quinoline]-1'-carboxylate (2q) S27
2.4.19. 1,1,1-trichloro-2-methylpropan-2-yl 6'-methoxy-2-oxo-1'*H*-spiro[cyclopentane-1,4'-quinoline]-1'-carboxylate (2r) S27
2.4.20. 1,1,1-trichloro-2-methylpropan-2-yl 6',7'-dimethoxy-2-oxo-1'*H*-spiro[cyclopentane-1,4'-quinoline]-1'-carboxylate (2s) S28

2.5. Activator Scope for Dearomative Spirocyclization Reaction S29
2.5.1. General Procedure C (GP3) S29
2.5.2. methyl 1-oxo-8-azaspiro[4.5]deca-6,9-diene-8-carboxylate (2aa) S29
2.5.3. 2,2,2-trichloroethyl 1-oxo-8-azaspiro[4.5]deca-6,9-diene-8-carboxylate (2ab) S30
2.5.4. allyl 1-oxo-8-azaspiro[4.5]deca-6,9-diene-8-carboxylate (2ac) S30
2.5.5. benzyl 1-oxo-8-azaspiro[4.5]deca-6,9-diene-8-carboxylate (2ad) S31
2.5.6. 1,1,1-trichloro-2-methylpropan-2-yl 1-oxo-8-azaspiro[4.5]deca-6,9-diene-8-carboxylate (2ae) S31
2.5.7. (9*H*-fluoren-9-yl)methyl 1-oxo-8-azaspiro[4.5]deca-6,9-diene-8-carboxylate (2af) S32
2.5.8. 8-pivaloyl-8-azaspiro[4.5]deca-6,9-dien-1-one (2ag) S32
2.5.9. 8-((trifluoromethyl)sulfonyl)-8-azaspiro[4.5]deca-6,9-dien-1-one (2ah) S33
2.5.10. 8-tosyl-8-azaspiro[4.5]deca-6,9-dien-1-one (2ai) S33
2.5.11. 8-((4-nitrophenyl)sulfonyl)-8-azaspiro[4.5]deca-6,9-dien-1-one (2aj) S34

2.6. Hydrogenation of Spirocyclic Dihydropyridines S35
2.6.1. *tert*-butyl 1-oxo-8-azaspiro[4.5]decane-8-carboxylate (4a) S35
2.6.2. *tert*-butyl 1-oxo-6-phenyl-8-azaspiro[4.5]decane-8-carboxylate (4b) S35

3. X-RAY CRYSTALLOGRAPHIC ANALYSIS S37
3.1. (9*H*-fluoren-9-yl)methyl 1-oxo-8-azaspiro[4.5]deca-6,9-diene-8-carboxylate (2af) S37

4. SPECTROSCOPIC DATA S40

5. REFERENCES S96

1. MATERIALS AND GENERAL METHODS

1.1. Glassware, Solvents and Reagents

All manipulations were performed with oven-dried (130 °C for a minimum of 12 h) or flame-dried glassware using standard Schlenk techniques under an atmosphere of nitrogen, unless otherwise stated.

All anhydrous solvents were commercially supplied or dried using an Anhydrous Engineering alumina column drying system (THF, MeCN, Et_2O). Reagents were purchased from commercial sources and used as received.

1.2. Chromatography and Instrumentation

Thin layer chromatography (TLC) was performed using Merck Kieselgel 60 F254 fluorescent treated silica, which was visualized under UV light, or by staining with aqueous basic potassium permanganate followed by heating or vanilin solution followed by heating, as stated.

Flash column chromatography (FCC) was carried out using Sigma-Aldrich silica gel (60 Å, 230-400 mesh, 40-63 μm).

NMR spectra were recorded at various field strengths, as indicated, using Bruker 400 MHz, Varian VNMR 400 MHz, Varian VNMR 500 MHz, or Bruker Cryo 500 MHz for ^{1}H, ^{13}C and ^{19}F acquisitions. All NMR spectra were recorder at 25 °C unless otherwise stated. Chemical shifts (δ) are reported in parts per million (ppm) and referenced $CDCl_3$ (^{1}H: 7.26 ppm; ^{13}C: 77.0 ppm). Coupling constants (*J*) are given in Hertz (Hz) and refer to apparent multiplicities (s = singlet, d = doublet, t = triplet, q = quartet, quin = quintet, hex = hextet, hept = heptet, m = multiplet, br = broad signal, dd = doublet of doublets, etc.). The ^{1}H NMR spectra are reported as follows: chemical shift (multiplicity, coupling constants, number of protons)

High resolution mass spectra (HRMS) were recorded on a Bruker Daltonics MicrOTOF II by Electrospray Ionisation (ESI); a Thermo Scientific QExactive by Electron Ionisation (EI); a Thermo Scientific Orbitrap Elite by ESI or Atmospheric Pressure Chemical Ionisation (APCI); or a Bruker UltrafleXtreme by Matrix-assisted Laser Desorption/Ionisation (MALDI).

IR spectra were recorded neat as a thin film on a Perkin Elmer Spectrum One FT-IR. Selected absorption maxima (ν_{max}) are reported in wavenumbers (cm^{-1}).

Optical rotations ($[\alpha]_D^T$) were recorded using a Bellingham & Stanley ADP 220 Polarimeter.

Melting points were obtained in open capillary tubes using a Stuart melting point apparatus SMP30 and are uncorrected.

1.3. Naming of Compounds

Compound names are those generated by ChemDraw Professional 20.0 software (PerkinElmer), following the IUPAC nomenclature.

2. EXPERIMENTAL DATA

2.1. Optimization Studies

2.1.1. Reaction optimization with TrocCl

Table 1: Reaction optimization with TrocCl

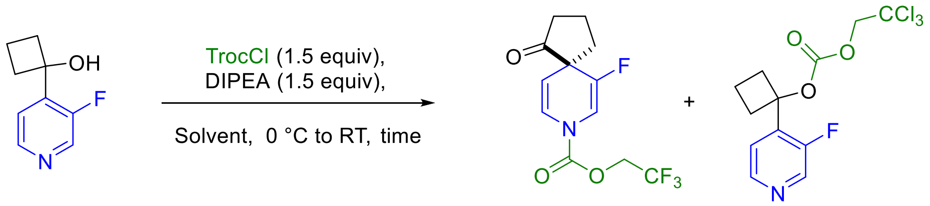

Entry	Solvent	Time / h	**2**; % Yield[a]	**3**;% Yield[a]
1[b]	$CHCl_3$	3	31	49
**2**	**$CHCl_3$**	**3**	**60**	**10**
3[c]	$CHCl_3$	3	35	37
4	MeCN	19	35	48
5	TBME	3	4	19
6	DCM	17	47	27

[a] ^{19}F NMR yield using 4-fluorotoluene as internal standard. [b] 1.0 equiv of Et_3N as base. [c] TrocCl added at −40 °C before warming to rt.

Observations: The combination of 2,2,2-trichloroethyl chloroformate (TrocCl) with Et_3N allowed access to desired product 2 in 31% yield, with 49% of the corresponding O-acylation species 3 (Table 1, entry 1). Using DIPEA as a base was shown to give a significant improvement on selectivity (entry 2). Decreasing the temperature at which the chloroformate was added to -40 °C was found to reduce the selectivity (compared to adding the chloroformate at 0 °C) and indeed the yield of 2 (entry 3). The reaction had a higher yield of 2 and more favourable selectivity when performed in chloroform compared to acetonitrile, tert-butyl methyl ether (TBME) or dichloromethane (entries 4 to 6).

2.1.2. Screen of acylating agents

Table 2: Screen of acylating agents

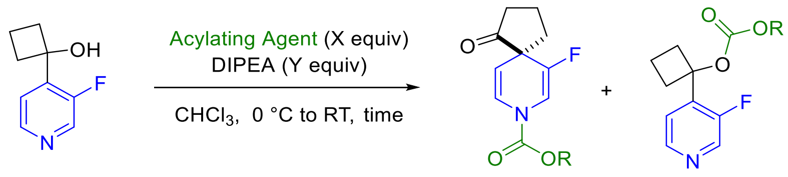

1h **2** **3**

Entry	Acylating agent (X equiv)	Y equiv	Time / h	**2**; % Yield[a]	**3**;% Yield[a]
1	TrocCl (1.5)	1.5	3	60	10
2	MeOCOCl (1.5)	1.5	3	64	11
3	CBzCl (1.5)	1.5	18	86	3
4	TCBocCl (1.5)	1.5	3	85	0
5	FmocCl (2.0)	2.0	18	85	0
6	TFAA (1.5)	1.5	18	0	97
7	Boc_2O (1.5)	0	17	0	0
**8[b]**	**Boc_2O (1.5)**	**0**	**7 d**	**81**	**0**

[a] ^{19}F NMR yield using 4-fluorotoluene as internal standard. [b] Reaction heated to 65 °C (oil bath).

Observations: Methyl chloroformate (table 2, entry 2) was found to have a similar selectivity for N-acylation/ring expansion as 2,2,2-trichloroethyl chloroformate (TrocCl, entry 1), while benzyl chloroformate (Cbz-Cl) gave almost exclusively the spirocycle product (2) with very little O-acylation (entry 3). The more sterically bulky 1,1-dimethyl-2,2,2-trichloroethyl chloroformate (TCBocCl, entry 4) and 9*H*-fluorenylmethyl chloroformate (FmocCl, entry 5) were found to exclusively give spirocyclic products 2 without any competing O-acylation. In contrast, trifluoroacetic anhydride (TFAA) gave exclusively the undesired O-acylation product 3 (entry 6). No reaction was observed between the starting material and di-tert-butyl dicarbonate (Boc_2O) at room temperature (entry 7). Heating the reaction to 65 °C gave full conversion of the starting material after 7 days to give an 81% yield of the spirocyclic product (entry 8).

2.1.3. Reaction optimization with Boc_2O

Table 3: Reaction optimization with Boc_2O

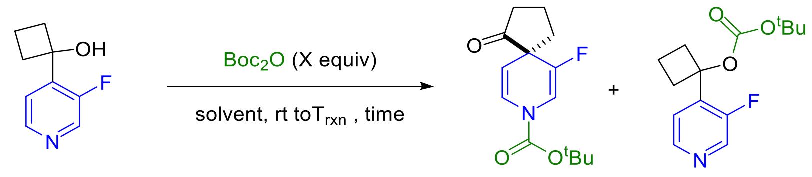

1h 2 3

Entry	Solvent	X equiv	T_{rxn} / °C	Time / h	**2**; % Yield[a]	**3**;% Yield[a]
1	$CHCl_3$	1.5	65	7 d	81	0
2[b]	$CHCl_3$	1.5	65	18	0	96
3	$CHCl_3$	5.0	65	6 d	88	0
4	1,2-DCB	1.5	120	18	3	0
5	1,2-DCE	1.5	85	48	25	0
6	1,4-dioxane	1.5	100	48	17	0
7	MeCN	1.5	85	48	44	0
**8**	MeCN	**5.0**	**85**	**24**	**85**	**0**

[a] ^{19}F NMR yield using 4-fluorotoluene as internal standard. [b] With 0.3 equiv of DMAP.

Observations: Addition of a sub-stoichiometric amount of 4-dimethylaminopyridine (DMAP) completely reversed selectivity in favour of O-acylation product 3 (entry 2). Increasing the stoichiometry of Boc_2O to 5 equivalents was found to have little impact on the rate of reaction in chloroform, with the reaction taking 6 days for full conversion of starting material (entry 3). Different reaction solvents were then screened, with MeCN proving optimal (entries 4 to 7). Increasing to 5 equivalents of Boc_2O was found to drastically improve the rate of reaction, giving full conversion of the starting material and an 85% yield of **2** after 24 hours at 85 °C (entry 8).

2.2. Synthesis of Non-Literature Known Precursors

2.2.1. 6-fluoro-4-iodoquinoline (SI-1)

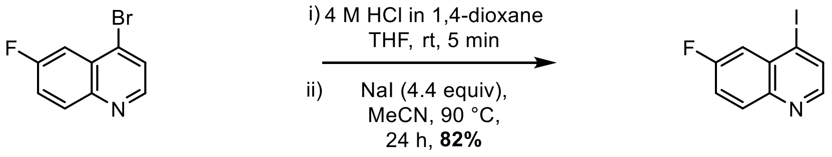

4-bromo-6-fluoroquinoline **SI-1**

According to a modified literature procedure:[1] To a solution of 4-bromo-6-fluoroquinoline (500 mg, 2.21 mmol, 1.0 equiv) in THF (7.4 mL) HCl (4 M in 1,4-dioxane, 0.72 mL) was added. The mixture was stirred for 5 min at rt and then concentrated under reduced pressure. The residue and NaI (dried previously at 120 °C under high vacuum, 1.46 g, 9.73 mmol, 4.4 equiv) were suspended in dry MeCN (11 mL) and stirred for 24 h at 90 °C (reflux, oil bath). Then, the solvent was removed under reduced pressure and a 1:1:1 mixture of H_2O, aq. sat. $NaHCO_3$ and aq. sat. $Na_2S_2O_3$ (10 mL) was added. The mixture was extracted with EtOAc (3x 30 mL). The org. layers were washed with aq. sat. $NaHCO_3$ (40 mL) and brine (2x 40 mL), combined, dried over $MgSO_4$, filtered, and concentrated under reduced pressure. The crude residue (937 mg) was purified by flash column chromatography (pentane:acetone 9:1) to give **SI-1** as a white solid (495 mg, 1.81 mmol, 82%).

Remark: The product contains approx. 6% unreacted starting material as impurity.

TLC (SiO_2; pentane:acetone 9:1, UV, vanilin): R_f = 0.43. **^{1}H NMR** (400 MHz, $CDCl_3$) δ 8.42 (d, *J* = 4.6 Hz, 1H), 8.06 (dd, *J* = 9.2, 5.5 Hz, 1H), 7.99 (d, *J* = 4.5 Hz, 1H), 7.70 (dd, *J* = 9.8, 2.8 Hz, 1H), 7.54 – 7.45 (m, 1H). **^{13}C NMR** (101 MHz, $CDCl_3$) δ 161.8 (d, *J* = 249.6 Hz), 149.2, 145.3, 133.3, 133.1 (d, *J* = 9.5 Hz), 131.9, 120.8 (d, *J* = 25.7 Hz), 115.6 (d, J = 24.9 Hz), 110.5. **IR** (thin film): υ = 2923, 2853, 1623, 1564, 1501, 1345, 1223, 855, 836 cm^{-1}. **HRMS** (ESI) m/z: $[M+H]^+$ Calcd for C_9H_6FIN 273.9523; Found 273.9529. **mp** = 97 – 98 °C. (see NMR spectra)

2.2.2. 4-iodo-6-methoxyquinoline (SI-2)

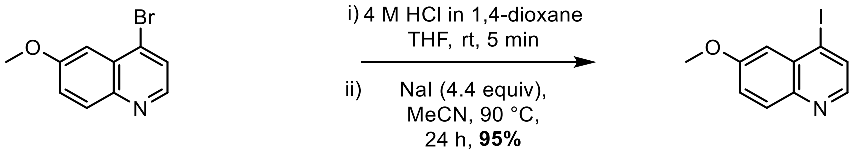

4-bromo-6-methoxyquinoline **SI-2**

According to a modified literature procedure:[1] To a solution of 4-bromo-6-methoxyquinoline (200 mg, 0.84 mmol, 1.0 equiv) in THF (1.4 mL) HCl (4 M in 1,4-dioxane, 0.28 mL) was added. The mixture was stirred for 5 min at rt and then concentrated under reduced pressure. The residue and NaI (dried previously at 120 °C (oil bath) under high vacuum, 554 mg, 3.70 mmol, 4.4 equiv) were suspended in dry MeCN (4.2 mL) and stirred for 24 h at 90 °C (reflux, oil bath). Then, the volatiles were removed under reduced pressure and a 1:1:1 mixture of H_2O, aq. sat. $NaHCO_3$ and aq. sat. $Na_2S_2O_3$ (5.0 mL) was added. The mixture was extracted with EtOAc (3x 15 mL). The org. layers were washed with aq. sat. $NaHCO_3$ (25 mL) and brine (2x 25 mL), combined, dried over $MgSO_4$, filtered, and concentrated under reduced pressure. The crude residue (443 mg) was purified by flash column chromatography (pentane:acetone 92.5:7.5) to give **SI-2** as a white solid (227 mg, 0.80 mmol, 95%).

TLC (SiO_2; pentane:acetone 9:1, UV, vanilin): R_f = 0.44. **^{1}H NMR** (400 MHz, $CDCl_3$) δ 8.36 (d, *J* = 4.6 Hz, 1H), 8.04 – 7.90 (m, 2H), 7.41 (dd, *J* = 9.2, 2.8 Hz, 1H), 7.26 (d, *J* = 2.8 Hz, 1H), 4.02 (s, 3H). **^{13}C NMR** (101 MHz, $CDCl_3$) δ 159.1, 147.1, 143.9, 132.6, 131.6, 131.5, 123.0, 110.3, 109.5, 55.6. **IR** (thin film): υ = 2921, 2852, 1617, 1499, 1232, 838 cm^{-1}. **HRMS** (EI) m/z: $[M]^+$ Calcd for $C_{10}H_8INO$ 284.9645; Found 284.9643. **mp** = 127 – 128 °C. (see NMR spectra)

2.2.3. 4-iodo-6,7-dimethoxyquinoline (SI-3)

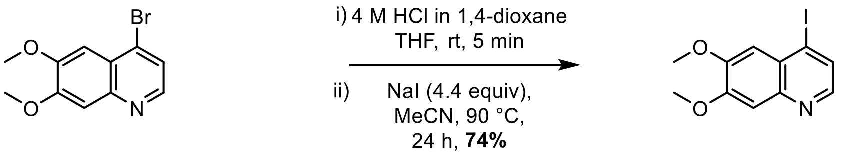

4-bromo-6,7-dimethoxyquinoline **SI-3**

According to a modified literature procedure:[1] To a solution of 4-bromo-6,7-dimethoxyquinoline (500 mg, 1.87 mmol, 1.0 equiv) in THF (6.2 mL) HCl (4 M in 1,4-dioxane, 0.61 mL) was added. The mixture was stirred for 5 min at rt and then concentrated under reduced pressure. The residue and NaI (dried previously at 120 °C (oil bath) under high vacuum, 1.23 g, 8.21 mmol, 4.4 equiv) were suspended in dry MeCN (9.3 mL) and stirred for 24 h at 90 °C (reflux, oil bath). Then the volatiles were removed under reduced pressure and a 1:1:1 mixture of H_2O, aq. sat. $NaHCO_3$ and aq. sat. $Na_2S_2O_3$ (10 mL) was added. The mixture was extracted with EtOAc (3x 30 mL). The org. layers were washed with aq. sat. $NaHCO_3$ (40 mL) and brine (2x 40 mL), combined, dried over $MgSO_4$, filtered, and concentrated under reduced pressure. The crude residue (748 mg) was purified by flash column chromatography (pentane:acetone 9:1 to 1:1) to give **SI-3** as a white solid (437 mg, 1.39 mmol, 74%).

Remark: The product contains approx. 24% unreacted starting material as an inseparable impurity.

TLC (SiO_2; pentane:acetone 9:1, UV, vanilin): R_f = 0.13. **^{1}H NMR** (400 MHz, $CDCl_3$) δ 8.27 – 8.19 (m, 1H), 7.85 – 7.78 (m, 1H), 7.39 – 7.33 (m, 1H), 7.26 – 7.20 (m, 1H), 4.06 (s, 3H), 4.04 (s, 3H). **^{13}C NMR** (101 MHz, $CDCl_3$) δ 153.1, 151.3, 147.5, 144.8, 130.7, 126.4, 109.7, 109.6, 108.4, 56.5, 56.3. **IR** (thin film): υ = 2922, 2852, 1499, 1249, 1224, 1137, 1008, 843 cm^{-1}. **HRMS** (EI) m/z: $[M]^+$ Calcd for $C_{11}H_{11}INO_2$ 315.9829; Found 315.9836. **mp** = 147 – 149 °C. (see NMR spectra)

2.3. Synthesis of hydroxycycloalkylpyridines (1)

2.3.1. 1-(pyridin-4-yl)cyclobutan-1-ol (1a)

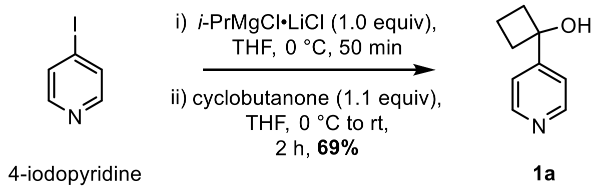

To a solution of 4-iodopyridine (615 mg, 3.0 mmol, 1.0 equiv) in THF (10 mL), cooled to 0 °C, was added Turbo Grignard (isopropyl magnesium chloride lithium chloride complex, 1.3 M in THF) (2.31 mL, 3.0 mmol, 1.0 equiv) dropwise over 20 min. The reaction mixture was stirred for a further 30 min at 0 °C before adding cyclobutanone (0.25 mL, 3.3 mmol, 1.1 equiv) as a solution in THF (2.0 mL) dropwise over 20 min. The reaction mixture was then stirred at 0 °C for 1 h before removing the ice bath and allowing the reaction mixture to warm to rt over 30 min. The reaction was then quenched by addition of sat. aq. NH_4Cl (10 mL) and extracted with EtOAc (3 x 20 mL). The combined organic layers were washed with brine (20 mL) and dried over $MgSO_4$ before concentrating *in vacuo*. The crude residue was purified by flash column chromatography (petroleum ether: acetone 88:12 to 100% acetone) to give **1a** (307 mg, 2.06 mmol, 69%) as a white solid.

^{1}H NMR (400 MHz, $CDCl_3$) δ 8.49 – 8.36 (m, 2H), 7.48 – 7.35 (m, 2H), 4.31 (s, 1H), 2.56 – 2.28 (m, 4H), 2.18 – 1.93 (m, 1H), 1.78 (m, 1H). **^{13}C NMR** (101 MHz, $CDCl_3$) δ 156.3, 149.4, 120.2, 75.5, 37.3, 13.1. **IR** (thin film): υ = 3203, 2988, 2940, 1602, 1411, 1250, 1147 cm^{-1}. **HRMS** (ESI) m/z: $[M+H]^+$ Calcd for $C_9H_{12}NO$ 150.0913; Found 150.0921. **mp** = 102 – 107 °C. (see NMR spectra)

2.3.2. 1-(pyridin-4-yl)cyclopropan-1-ol (1b)

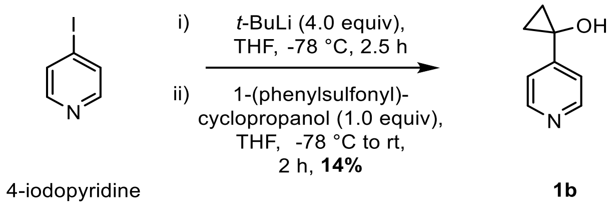

To a solution of 4-iodopyridine (1230 mg, 6.0 mmol, 2.0 equiv) in THF (16 mL), cooled to -78 °C was added *t*-butyllithium (1.48 M, 8.1 mL, 12.0 mmol, 4.0 equiv) dropwise over 30 min. The reaction was then stirred for 2 h at -78 °C before adding 1-(phenylsulfonyl)cyclopropanol[2] (595 mg, 3.0 mmol, 1.0 equiv) as a solution in THF (4 mL) dropwise over 20 min. The reaction was then warmed to -30 °C and stirred for 2 h. The reaction mixture was then warmed to rt and quenched with sat. aq. NH_4Cl. The layers were separated, and the aq. layer was extracted with EtOAc. The combined organic phases were dried over $MgSO_4$ and concentrated *in vacuo*. The crude residue was purified by flash column chromatography (petroleum ether:acetone 88:12 to 100% acetone) to give **1b** (58 mg, 0.43 mmol, 14%) as a beige solid.

^{1}H NMR (400 MHz, $CDCl_3$) δ 8.43 – 8.41 (m, 2H), 7.15 – 7.14 (m, 2H), 1.41 (dd, J = 7.8, 5.4 Hz, 2H), 1.11 (dd, J = 7.8, 5.4 Hz, 2H). **^{13}C NMR** (101 MHz, $CDCl_3$) δ 155.5, 149.3, 118.7, 54.9, 20.4. **IR** (thin film): υ = 3193, 3079, 2861, 1603, 1438, 1413, 1262, 1120 cm^{-1}. **HRMS** (ESI) m/z: $[M+H]^+$ Calcd for $C_8H_{10}NO$ 136.0755 Found: 136.0754. **mp** = 162– 164 °C. (see NMR spectra)

2.3.3. 1-(pyridin-4-yl)cyclopentan-1-ol (1c)

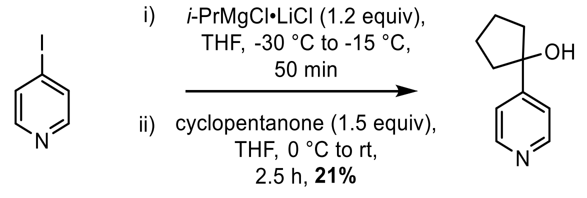

4-iodopyridine **1c**

To a solution of 4-iodopyridine (615 mg, 3.0 mmol, 1.0 equiv) in THF (10 mL), cooled to -30 °C, was added Turbo Grignard (isopropyl magnesium chloride lithium chloride complex, 1.3 M in THF) (2.80 mL, 3.6 mmol, 1.2 equiv) dropwise over 20 min. The reaction was allowed to warm to -15 °C over 30 min before adding cyclopentanone (0.40 mL, 4.5 mmol, 1.5 equiv) as a solution in THF (2.5 mL) dropwise over 20 min. The reaction was then allowed to warm to ambient temperature over 2 h. The reaction was then quenched by addition of sat. aq. NH_4Cl (10 mL) and extracted with EtOAc (3 x 20 mL). The combined organic layers were washed with brine (20 mL) and dried over $MgSO_4$ before concentrating *in vacuo*. The crude residue was purified by flash column chromatography (petroleum ether:acetone 88:12 to 100% acetone) to give **1c** (104 mg, 0.64 mmol, 21%) as a white solid.

^{1}H NMR (400 MHz, $CDCl_3$) δ 8.54 – 8.52 (m, 2H), 7.40 – 7.38 (m, 2H), 2.05 – 1.85 (m, 8H). **^{13}C NMR** (101 MHz, $CDCl_3$) δ 157.0, 149.4, 120.5, 82.4, 42.6, 24.3. **IR** (thin film): υ = 3164, 2965, 1403, 1601, 1407, 1240, 1003 cm^{-1}. **HRMS** (ESI) m/z: $[M+H]^+$ Calcd for $C_{10}H_{14}NO$ 164.1070; Found 164.1066. **mp** = 84 – 89 °C. (see NMR spectra)

2.3.4. 1-(pyridin-4-yl)cyclohexan-1-ol (1d)

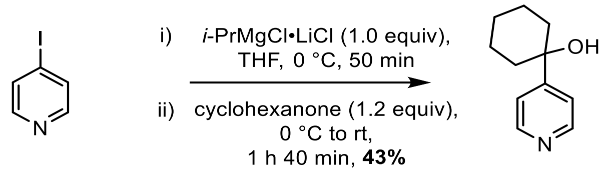

4-iodopyridine **1d**

To a solution of 4-iodopyridine (615 mg, 3.0 mmol, 1.0 equiv) in THF (10 mL), cooled to 0 °C, was added Turbo Grignard (isopropyl magnesium chloride lithium chloride complex, 1.3 M in THF) (2.31 mL, 3.0 mmol, 1.0 equiv) dropwise over 20 min. The reaction was stirred at 0 °C for 30 min before adding cyclohexanone (neat, 0.38 mL, 3.6 mmol, 1.2 equiv) dropwise over 10 min. The reaction mixture was then stirred at 0 °C for 30 min before warming to rt and stirring for 1 h. The reaction was then quenched by addition of sat. aq. NH_4Cl (10 mL) and extracted with EtOAc (3 x 20 mL). The combined organic layers were washed with brine (20 mL) and dried over $MgSO_4$ before concentrating *in vacuo*. The crude residue was purified by flash column chromatography (petroleum ether:acetone 88:12 to 100% acetone) to yield **1d** (231 mg, 1.30 mmol, 43%) as a white solid.

^{1}H NMR (400 MHz, $CDCl_3$) δ 8.53 – 8.9 (m, 2H), 7.48 – 7.31 (m, 2H), 2.68 – 2.12 (br. s, 1H), 1.85 – 1.57 (m, 9H), 1.38 – 1.20 (m, 1H). **^{13}C NMR** (101 MHz, $CDCl_3$) δ 149.8, 149.7, 120.0, 72.8, 38.4, 25.4, 21.9. **IR** (thin film): υ = 3206, 2935, 2914, 2858, 1597, 1410, 1268, 988, 816, 725, 643, 559 cm^{-1}. **HRMS** (ESI) m/z: $[M+H]^+$ Calcd for $C_{11}H_{16}NO$ 178.1226; Found 178.1221. **mp** = 146 – 151 °C. (see NMR spectra)

2.3.5. 3-(pyridin-4-yl)oxetan-3-ol (1e)

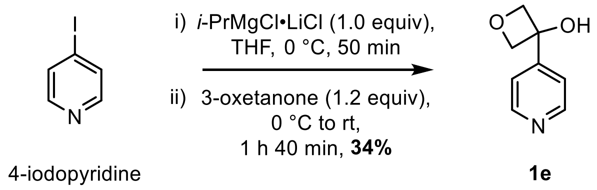

To a solution of 4-iodopyridine (615 mg, 3.0 mmol, 1.0 equiv) in THF (10 mL), cooled to 0 °C, was added Turbo Grignard (isopropyl magnesium chloride lithium chloride complex, 1.3 M in THF) (2.31 mL, 3.0 mmol, 1.0 equiv) dropwise over 20 min. The reaction was stirred at 0 °C for 30 min before adding 3-oxetanone (neat, 0.22 mL, 3.6 mmol, 1.2 equiv) dropwise over 10 min. The reaction was then stirred at 0 °C for 30 min before warming to rt and stirring for 1 h. The reaction was then quenched by addition of sat. aq. NH_4Cl (10 mL) and extracted with EtOAc (3 x 20 mL). The combined organic layers were washed with brine (20 mL) and dried over $MgSO_4$ before concentrating *in vacuo*. The crude residue was purified by flash column chromatography (petroleum ether:acetone 88:12 to 100% acetone) to yield **1e** (155 mg, 1.03 mmol, 34%) as a white solid.

^{1}H NMR (400 MHz, $CDCl_3$) δ 8.65 (d, J = 6.0 Hz, 2H), 7.65 – 7.60 (m, 2H), 4.95 (d, J = 7.7 Hz, 2H), 4.83 (d, J = 7.7 Hz, 2H), 3.38 (br. s, 1H). **^{13}C NMR** (101 MHz, $CDCl_3$) δ 151.7, 150.1, 119.5, 85.9, 74.7. **IR** (thin film): υ = 3158, 2923, 2852, 1734, 1265, 1261, 750 cm^{-1}. **HRMS** (ESI) m/z: [M+H]$^+$ Calcd for $C_8H_{10}NO_2$ 152.0706; Found 152.0703. **mp** = 172 – 177 °C. (see NMR spectra)

2.3.6. *tert*-butyl 3-hydroxy-3-(pyridin-4-yl)azetidine-1-carboxylate (1f)

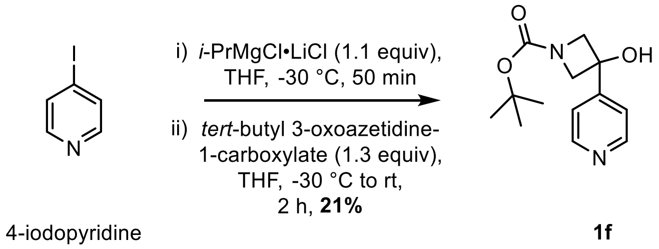

To a solution of 4-iodopyridine (615 mg, 3.0 mmol, 1.0 equiv) in THF (10 mL), cooled to -30 °C, was added Turbo Grignard (isopropyl magnesium chloride lithium chloride complex, 1.3 M in THF) (2.54 mL, 3.3 mmol, 1.1 equiv) dropwise over 20 min. The reaction was allowed to stir at -30 °C for 30 min before adding *tert*-butyl 3-oxoazetidine-1-carboxylate (668 mg, 3.9 mmol, 1.3 equiv) as a solution in THF (2.5 mL) dropwise over 20 min. The reaction was then allowed to warm to rt over 1.5 h. The reaction was then quenched by addition of sat. aq. NH_4Cl (10 mL) and extracted with EtOAc (3 x 20 mL). The combined organic layers were washed with brine (20 mL) and dried over $MgSO_4$ before concentrating *in vacuo*. The crude residue was purified by flash column chromatography (petroleum ether:acetone 88:12 to 100% acetone) to yield **1f** (104 mg, 0.64 mmol, 21%) as a white solid.

^{1}H NMR (400 MHz, $CDCl_3$) δ 8.63 – 8.47 (m, 2H), 7.49 (m, 2H), 4.26 – 4.04 (m, 4H), 1.46 (s, 9H). **^{13}C NMR** (101 MHz, $CDCl_3$) δ 156.6, 154.0, 149.4, 119.9, 80.5, 69.5, 64.9, 28.5. **IR** (thin film): υ = 3142, 2976, 2882, 1699, 1674, 1605, 1390, 1366, 1250, 1165, 1113 cm^{-1}. **HRMS** (ESI) m/z: [M+H]$^+$ Calcd for $C_{13}H_{19}N_2O_3$ 251.1390; Found 251.1383. **mp** = 162 – 167 °C. (see NMR spectra)

2.3.7. (1*R*,5*S*)-6-(pyridin-4-yl)bicyclo[3.2.0]hept-2-en-6-ol (1g)

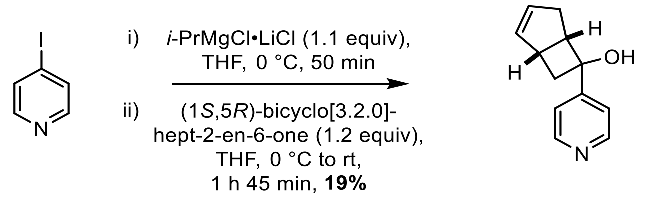

4-iodopyridine **1g**

To a solution of 4-iodopyridine (615 mg, 3.0 mmol, 1.0 equiv) in THF (10 mL), cooled to 0 °C, was added Turbo Grignard (isopropyl magnesium chloride lithium chloride complex, 1.3 M in THF) (2.31 mL, 3.0 mmol, 1.0 equiv) dropwise over 20 min. The reaction was stirred at 0 °C for 30 min before adding (1*S*,5*R*)-bicyclo[3.2.0]hept-2-en-6-one[3,4] (389 mg, 3.6 mmol, 1.2 equiv) as a solution in THF (2.5 mL), dropwise over 15 min. The reaction was then stirred at 0 °C for 30 min before warming to rt and stirring for 1 h. The reaction was then quenched by addition of sat. aq. NH_4Cl (10 mL) and extracted with EtOAc (3 x 20 mL). The combined organic layers were washed with brine (20 mL) and dried over $MgSO_4$ before concentrating *in vacuo*. The crude residue was purified by flash column chromatography (petroleum ether:acetone 93:7 to 40:60) to yield **1g** (106 mg, 0.57 mmol, 19%) as a beige solid.

^{1}H NMR (400 MHz, $CDCl_3$) δ 8.60 – 8.45 (m, 2H), 7.47 – 7.35 (m, 2H), 5.96 (m, 2H), 3.31 – 3.19 (m, 2H), 2.92 – 2.85 (m, 1H), 2.85 – 2.77 (m, 1H), 2.49 (dddd, *J* = 15.5, 7.5, 4.0, 2.0 Hz, 1H), 2.06 (dd, *J* = 13.5, 2.5 Hz, 1H). **^{13}C NMR** (101 MHz, $CDCl_3$) δ 156.5, 149.7, 135.5, 133.0, 119.8, 75.5, 48.6, 45.1, 39.7, 32.9. **IR** (thin film): υ = 3173, 3047, 2930, 2845, 1601, 1413, 1239, 1003 cm^{-1}. **HRMS** (ESI) m/z: $[M+H]^+$ Calcd for $C_{12}H_{14}NO$ 188.1070; Found 188.1064. **mp** = 76 – 81 °C. (see NMR spectra)

2.3.8. 1-(3-fluoropyridin-4-yl)cyclobutan-1-ol (1h)

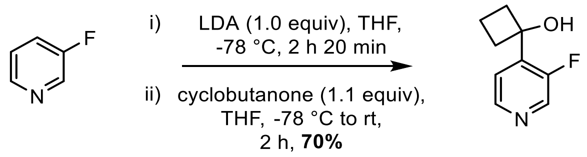

3-fluoropyridine **1h**

A solution of LDA in THF was prepared by dropwise addition of *n*-BuLi (1.6 M in hexane, 3.75 mL, 6.0 mmol, 1.0 equiv) to a solution of diisopropylamine (0.84 mL, 6.0 mmol, 1 equiv) in THF (10 mL) at -78 °C and stirring for 1 h at this temperature. To the LDA solution was added, dropwise over 20 min at -78 °C, a solution of 3-fluoropyridine (0.52 mL, 6.0 mmol, 1.0 equiv) in THF (2.5 mL). The reaction was stirred for 2 h at -78 °C, before dropwise addition of a solution of cyclobutanone (0.48 mL, 6.6 mmol, 1.1 equiv) in THF (2.5 mL). The reaction mixture was then warmed to 0 °C and stirred for 2 h at this temperature, before warming to rt and quenching with sat. aq. NH_4Cl (20 mL). The reaction was extracted with EtOAc (3 x 20 mL), organic layers combined and washed with brine (20 mL), dried over $MgSO_4$ and concentrated *in vacuo*. The crude residue was purified by flash column chromatography (pentane:acetone 93:7 to 40:60) to give **1h** (703 mg, 4.2 mmol, 70%) as a white solid.

^{1}H NMR (400 MHz, $CDCl_3$) δ 8.42 (d, *J* = 3.0 Hz, 1H), 8.38 (dd, *J* = 5.0, 1.0 Hz, 1H), 7.33 (dd, *J* = 7.0, 5.0 Hz, 1H), 2.75 – 2.57 (m, 3H), 2.46 – 2.30 (m, 2H), 2.18 (m, 1H), 1.90 – 1.76 (m, 1H). **^{13}C NMR** (101 MHz, $CDCl_3$) δ 158.2 (d, J = 256 Hz), 145.8 (d, J = 11.5 Hz), 141.2 (d, J = 25 Hz), 138.5 (d, J = 26.5 Hz), 121.3 (d, J = 9 Hz), 74.7, 35.7, 14.1. **^{19}F NMR** (101 MHz, $CDCl_3$) δ -129.6 **IR** (thin film): υ = 3256, 2992, 2950, 1416, 1220, 1133, 1060 cm^{-1}. **HRMS** (ESI) m/z: [M+H]$^+$ Calcd for $C_9H_{11}NOF$ 168.0819; Found 168.0813. **mp** = 55 – 60 °C. (see NMR spectra)

2.3.9. 1-(3-bromopyridin-4-yl)cyclobutan-1-ol (1i)

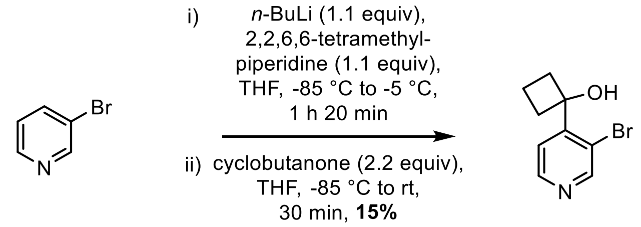

3-bromopyridine **1i**

To a solution of 2,2,6,6-tetramethylpiperidine (1.12 mL, 6.6 mmol, 1.1 equiv) in THF (12 mL) was added *n*-BuLi (1.6 M in hexanes, 4.13 mL, 6.6 mmol, 1.1 equiv) dropwise over 20 min at -40 °C. The reaction mixture was then warmed to -5 °C and stirred for 15 min at this temperature. The reaction mixture was then cooled to -85 °C and a solution of 3-bromopyridine (578 µL, 948 mg, 6.0 mmol, 1.0 equiv) in THF (2.5 mL) added dropwise over 20 min. The reaction was then stirred at 25 °C for 25 min before adding a solution of cyclobutanone (986 µL, 13.2 mmol, 2.2 equiv) in THF (1 mL) in one portion. The reaction mixture was stirred at -85 °C for 15 min and then warmed to rt. The reaction was quenched with aq. sat. $NaHCO_3$ (15 mL), extracted with EtOAc (3 x 20 mL), and organic layers combined and washed with brine (20 mL). Organic layers were dried over anhydrous $MgSO_4$ and concentrated *in vacuo*. The crude residue was purified by flash column chromatography on alumina (petroleum ether:EtOAc 80:20) to give **1i** (205 mg, 0.9 mmol, 15%) as a white solid.

^{1}H NMR (400 MHz, $CDCl_3$) δ 8.66 (s, 1H), 8.47 (dd, *J* = 5.0, 1.0 Hz, 1H), 7.28 (d, *J* = 5.0 Hz, 1H), 3.20 (s, 1H), 2.71 – 2.58 (m, 2H), 2.52 – 2.41 (m, 2H), 2.26 – 2.11 (m, 1H), 1.71 (dtt, *J* = 11.0, 9.0, 5.5 Hz, 1H). **^{13}C NMR** (101 MHz, $CDCl_3$) δ 153.2, 151.7, 148.8, 121.9, 120.3, 78.1, 34.9, 14.4. **IR** (thin film): υ = 3260, 2988, 2948, 1585, 1398, 1141, 1030 cm^{-1}. **HRMS** (ESI) m/z: [M+H]$^+$ Calcd for $C_9H_{11}NOBr$ 228.0019; Found 228.0028. **mp** = 85 – 90 °C. (see NMR spectra)

2.3.10. 4-(1-hydroxycyclobutyl)-*N*,*N*-diisopropylnicotinamide (1j)

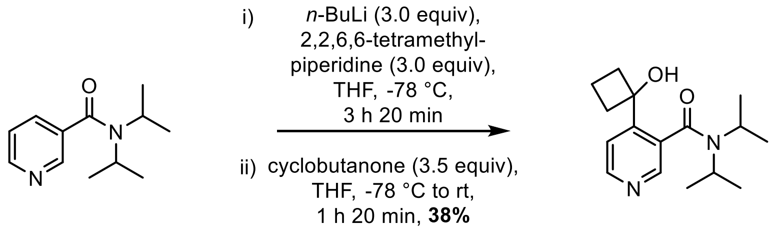

N,*N*-diisopropyl-nicotinamide **1j**

A solution of lithium 2,2,6,6-tetramethylpyridide (LiTMP) in THF was prepared by dropwise addition of *n*-BuLi

(1.6 M in hexanes, 5.62 mL, 9.0 mmol, 3.0 equiv) to a solution of 2,2,6,6-tetramethylpiperidine (1.53 mL, 9.0 mmol, 3.0 equiv) in THF (9.0 mL) at -78 °C and stirring for 1 h at this temperature. To the LiTMP solution was added, dropwise over 20 minutes at -78 °C, a solution of *N*,*N*-diisopropylnicotinamide (619 mg, 3.0 mmol, 1.0 equiv) in THF (9.0 mL). The reaction was stirred for 3 h at -78 °C, before dropwise addition over 20 minutes of cyclobutanone (0.79 mL, 10.5 mmol, 3.5 equiv). The reaction was then warmed to rt and stirred for 1 h at this temperature, before quenching with sat. aq. NH_4Cl (20 mL). The reaction was extracted with DCM (3 x 20 mL), organic layers combined, dried over $MgSO_4$ and concentrated *in vacuo*. The crude residue was purified by flash column chromatography (petroleum ether:EtOAc 40:60) to give **1j** (316 mg, 1.14 mmol, 38%) as a white solid.

^{1}H NMR (400 MHz, $CDCl_3$) δ 8.61 – 8.56 (d, *J* = 5.0 Hz, 1H), 8.41 (s, 1H), 7.29 (d, *J* = 5.0 Hz, 1H), 5.07 (s, 1H), 3.93 (qq, *J* = 7.0, 7.0 Hz, 1H), 3.55 (qq, *J* = 7.0 Hz, 1H), 2.68 – 2.55 (m, 1H), 2.40 – 2.18 (m, 4H), 1.79 – 1.67 (m, 1H), 1.55 (d, *J* = 7.0 Hz, 3H), 1.51 (d, *J* = 7.0 Hz, 3H), 1.29 (d, *J* = 7.0 Hz, 3H), 1.17 (d, *J* = 7.0 Hz, 3H). **^{13}C NMR** (101 MHz, $CDCl_3$) δ 170.2, 153.3, 150.9, 146.9, 130.8, 120.9, 76.5, 51.9, 46.7, 36.2, 32.4, 21.0, 21.0, 20.3, 20.3, 15.0. **IR** (thin film): υ = 3353, 2972, 1609, 1444, 1370, 1344, 1032 cm^{-1}. **HRMS** (ESI) m/z: $[M+H]^+$ Calcd for $C_{16}H_{25}N_2O_2$ 277.1911; Found 277.1904. **mp** = 113 – 118 °C. (see NMR spectra)

2.3.11. 1-(3-phenylpyridin-4-yl)cyclobutan-1-ol (1k)

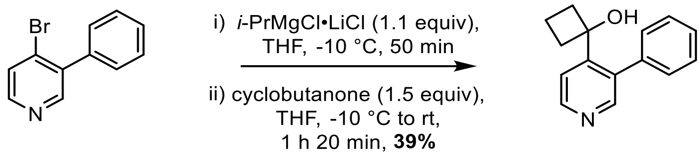

4-bromo-3-phenylpyridine **1k**

To a solution of 4-bromo-3-phenylpyridine[5] (700 mg, 2.9 mmol, 1.0 equiv) in THF (6 mL), cooled to -10 °C, was added Turbo Grignard (isopropyl magnesium chloride lithium chloride complex, 1.3 M in THF) (2.45 mL, 3.2 mmol, 1.1 equiv) dropwise over 20 min. The reaction was stirred for a further 30 min at -10 °C before adding cyclobutanone (0.33 mL, 4,35 mmol, 1.5 equiv) as a solution in THF (1.5 mL) dropwise over 20 min. The reaction mixture was then allowed to warm to rt over 1 h. The reaction was then quenched by addition of sat. aq. NH_4Cl (10 mL) and extracted with EtOAc (3 x 20 mL). The combined organic layers were washed with brine (20 mL) and dried over $MgSO_4$ before concentrating *in vacuo*. The crude residue was purified by flash column chromatography (petroleum ether:EtOAc 93:7 to 40:60) to give **1k** (252 mg, 1.12 mmol, 39%) as a white solid.

^{1}H NMR (400 MHz, $CDCl_3$) δ 8.52 (d, *J* = 5.0 Hz, 1H), 8.40 (s, 1H), 7.51 – 7.33 (m, 5H), 7.28 (d, *J* = 5.0 Hz, 1H), 3.21 (br. s, 1H), 2.36 – 2.25 (m, 2H), 2.17 – 2.03 (m, 1H), 2.01 – 1.90 (m, 2H), 1.67 – 1.55 (m, 1H). **^{13}C NMR** (101 MHz, $CDCl_3$) δ 152.0, 151.4, 148.6, 138.3, 136.4, 129.9, 128.3, 128.1, 120.7, 77.9, 36.0, 15.2. **IR** (thin film): υ = 2946, 1592, 1401, 1234, 1135, 756, 700 cm^{-1}. **HRMS** (EI) m/z: $[M]^+$ Calcd for $C_{15}H_{15}NO$ 225.1148; Found 225.1146. **mp** = 97 – 102 °C. (see NMR spectra)

2.3.12. 1-(2-methylpyridin-4-yl)cyclobutan-1-ol (1l)

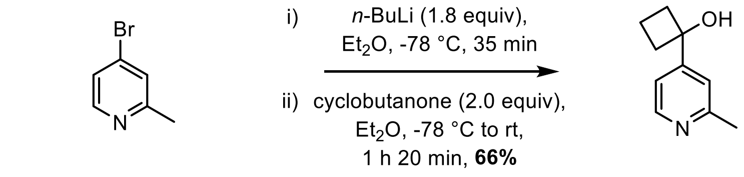

4-bromo-2-methylpyridine **1l**

To a solution of 4-bromo-2-methylpyridine (145 mg, 0.84 mmol, 1.0 equiv) in Et_2O (50 mL, 0.02 M) cooled to -78 °C was added *n*-butyllithium (1.6 M in hexanes, 0.95 mL, 1.52 mmol, 1.8 equiv) dropwise over 20 min. The reaction mixture was then stirred at -78 °C for 15 min before adding a solution of cyclobutanone (0.13 mL, 1.68 mmol, 2.0 equiv) in Et_2O (2.5 mL) dropwise over 20 min at -78 °C. The reaction mixture was then warmed to rt and stirred for 1 h, before quenching the reaction with sat. aq. NH_4Cl, extracting with EtOAc (3 x 20 mL), drying over $MgSO_4$ and concentrating *in vacuo*. The crude residue was purified by flash column chromatography (petroleum ether:EtOAc 70:30 to 50:50) to give **1l** (90 mg, 0.54 mmol, 66%) as a white solid.

^{1}H NMR (400 MHz, $CDCl_3$) δ 8.34 (dd, *J* = 5.0, 1.0 Hz, 1H), 7.28 (dd, *J* = 1.5, 1.0 Hz, 1H), 7.21 (ddd, *J* = 5.0, 1.5, 0.5 Hz, 1H), 3.80 (s, 1H), 2.51 (s, 3H), 2.50 – 2.43 (m, 2H), 2.43 – 2.33 (m, 2H), 2.12 – 1.99 (m, 1H), 1.76 (m, 1H). **^{13}C NMR** (101 MHz, $CDCl_3$) δ 158.4, 156.3, 148.9, 119.6, 117.2, 75.7, 37.2, 24.4, 13.1. **IR** (thin film): υ = 3201, 2986, 2940, 1606, 1553, 1385, 1249, 1141 cm^{-1}. **HRMS** (ESI) m/z: $[M+H]^+$ Calcd for $C_{10}H_{14}NO$ 164.1070; Found 164.1075. **mp** = 60 – 65 °C. (see NMR spectra)

2.3.13. 1-(2-phenylpyridin-4-yl)cyclobutan-1-ol (1m)

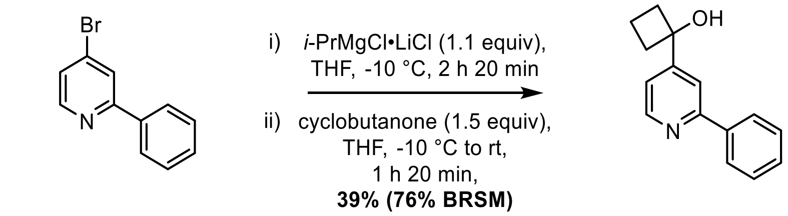

4-bromo-2-phenylpyridine **1m**

To a solution of 4-bromo-2-phenylpyridine[6] (142 mg, 0.6 mmol, 1.0 equiv) in THF (1.2 mL), cooled to -10 °C, was added Turbo Grignard (isopropyl magnesium chloride lithium chloride complex, 1.3 M in THF) (0.51 mL, 0.66 mmol, 1.1 equiv) dropwise over 20 min (solution turned red). The reaction was stirred for a further 2 h at -10 °C before adding cyclobutanone (0.07 mL, 0.9 mmol, 1.5 equiv) as a solution in THF (0.75 mL) dropwise over 20 min. The reaction mixture was then allowed to warm to rt over 1 h. The reaction was quenched by addition of aq. sat. NH_4Cl (10 mL) and extracted with EtOAc (3 x 20 mL). The combined organic layers were washed with brine (20 mL) and dried over $MgSO_4$ before concentrating *in vacuo*. The crude residue (191 mg) was purified by flash column chromatography (pentane:acetone 93:7) to give **1m** (53.5 mg, 0.24 mmol, 39%) as a colorless oil. In addition, 4-bromo-2-phenylpyridine (68.7 mg, 0.29 mmol, 48%) was recovered.

TLC (SiO_2; pentane:acetone 9:1, UV, vanillin): R_f = 0.17. **^{1}H NMR** (400 MHz, $CDCl_3$) δ 8.67 – 8.57 (m, 1H), 7.96 (d, *J* = 6.8 Hz, 2H), 7.83 (s, 1H), 7.52 – 7.37 (m, 3H), 7.33 (d, *J* = 5.7 Hz, 1H), 2.82 (br s, 1H), 2.60 – 2.47 (m, 2H), 2.47 – 2.35 (m, 2H), 2.17 – 2.02 (m, 1H), 1.88 – 1.74 (m, 1H). **^{13}C NMR** (101 MHz, $CDCl_3$) δ 157.9, 156.3, 149.8, 139.6, 129.1, 128.9, 127.2, 118.5, 117.0, 76.2, 37.3, 13.2. **IR** (thin film): υ = 3293, 2986, 2939, 1599,

1394, 1249, 1143 cm^{-1}. **HRMS** (ESI) m/z: $[M+H]^+$ Calcd for $C_{15}H_{16}NO$ 226.1226; Found 226.1228. (see NMR spectra)

2.3.14. 1-(quinolin-4-yl)cyclobutan-1-ol (1n)

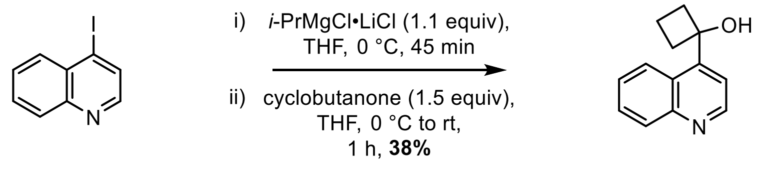

4-iodoquinoline **1n**

To a solution of 4-iodoquinoline[1] (co-evaporated with toluene, 350 mg, 1.37 mmol, 1.0 equiv) in THF (5.5 mL), cooled to 0 °C, was added Turbo Grignard (isopropyl magnesium chloride lithium chloride complex, 1.3 M in THF) (1.16 mL, 1.51 mmol, 1.1 equiv) dropwise over 5 min. The reaction mixture was stirred for a further 40 min at 0 °C, before adding cyclobutanone (0.15 mL, 144 mg, 2.06 mmol, 1.5 equiv) dropwise over 5 min. The reaction mixture was stirred at 0 °C for 60 min and then warmed to rt, quenched with sat. aq. NH_4Cl (10 mL) and extracted with EtOAc (3x 15 mL). The combined organic layers were washed with brine and dried over $MgSO_4$ before concentrating *in vacuo*. The crude residue (286 mg) was purified by flash column chromatography (pentane:acetone 80:20 to 50:50) to give **1n** (105 mg, 0.53 mmol, 38%) as an off-white solid.

TLC (SiO_2; pentane:acetone 4:1, UV, vanillin): R_f = 0.34. **^{1}H NMR** (400 MHz, $CDCl_3$) δ 8.67 (d, *J* = 4.5 Hz, 1H), 8.29 (dd, *J* = 8.5, 1.5 Hz, 1H), 8.05 (dd, *J* = 8.5, 1.5 Hz, 1H), 7.66 (ddd, *J* = 8.5, 7.0, 1.5 Hz, 1H), 7.52 (ddd, *J* = 8.5, 7.0, 1.5 Hz, 1H), 7.32 – 7.19 (m, 1H), 3.35 (s, 1H), 2.86 – 2.68 (m, 2H), 2.60 (ddd, *J* = 12.5, 9.0, 7.0 Hz, 2H), 2.15 (dddd, *J* = 11.0, 9.5, 5.5, 3.5 Hz, 1H), 1.69 (dddd, *J* = 13.0, 8.5, 6.5, 4.5 Hz, 1H). **^{13}C NMR** (101 MHz, $CDCl_3$) δ 149.9, 149.7, 149.2, 130.2, 129.2, 126.5, 126.3, 126.1, 117.3, 77.4, 36.5, 14.4. **IR** (thin film): υ = 3215, 2941, 1590, 1509, 1248, 1117, 761 cm^{-1}. **HRMS** (ESI) m/z: $[M+H]^+$ Calcd for $C_{13}H_{14}NO$ 200.1070; Found 200.1064. **mp** = 60 – 65 °C. (see NMR spectra)

2.3.15. 1-(7-chloroquinolin-4-yl)cyclobutan-1-ol (1o)

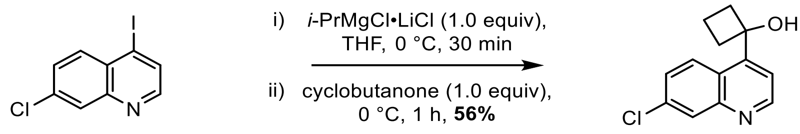

7-chloro-4-iodoquinoline **1o**

To a solution of 7-chloro-4-iodoquinoline[1] (578 mg, 2.00 mmol, 1.0 equiv) in THF (8.0 mL), cooled to 0 °C, was added Turbo Grignard (isopropyl magnesium chloride lithium chloride complex, 1.3 M in THF) (1.54 mL, 2.00 mmol, 1.0 equiv) dropwise over 20 min. The yellow reaction mixture was stirred for a further 10 min at 0 °C, before adding cyclobutanone (neat, 0.15 mL, 140 mg, 2.00 mmol, 1.0 equiv) dropwise over 5 min. The resulting colorless reaction mixture was stirred at 0 °C for 60 min, quenched with sat. aq. NH_4Cl (10 mL) and extracted with EtOAc (3x 20 mL). The combined organic layers were washed with brine and dried over $MgSO_4$ before concentrating *in vacuo*. The crude residue (488 mg) was purified by flash column chromatography (pent:EtOAc 2:1) to give **1o** (262 mg, 1.12 mmol, 56%) as a white solid. For analytical purposes this material was

recrystallized in EtOAc.

TLC (SiO_2; pentane:EtOAc 2:1, UV, $KMnO_4$): R_f = 0.26. **^{1}H NMR** (500 MHz, $CDCl_3$) δ 8.73 (d, *J* = 4.6 Hz, 1H), 8.26 (d, *J* = 9.1 Hz, 1H), 8.05 (d, *J* = 2.2 Hz, 1H), 7.48 (dd, *J* = 9.1, 2.2 Hz, 1H), 7.30 (d, *J* = 4.5 Hz, 1H), 2.97 (br s, 1H), 2.76 (ddd, 2H), 2.59 (ddd, *J* = 12.7, 9.3, 7.0 Hz, 2H), 2.17 (dtt, *J* = 11.2, 9.3, 5.6 Hz, 1H), 1.71 (dtt, *J* = 11.2, 8.8, 7.1 Hz, 1H). **^{13}C NMR** (126 MHz, $CDCl_3$) δ 150.9, 150.0, 149.7, 135.2, 129.0, 128.0, 127.3, 124.5, 117.4, 77.3, 36.6, 14.3. **IR** (thin film): υ = 3240, 2955, 2932, 1727, 1604, 1588, 1495, 1251, 1148, 1119, 1077, 878, 828 cm^{-1}. **HRMS** (ESI) m/z: $[M+H]^+$ Calcd for $C_{13}H_{13}ClNO$ 234.0680; Found 234.0680. **mp** = 160 – 165 °C. (see NMR spectra)

2.3.16. 1-(6-bromoquinolin-4-yl)cyclobutan-1-ol (1p)

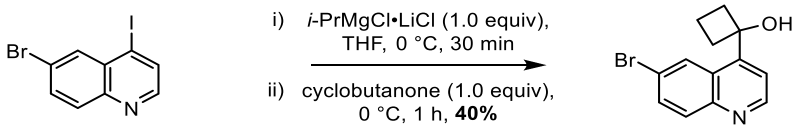

6-bromo-4-iodoquinoline **1p**

To a solution of 6-bromo-4-iodoquinoline (500 mg, 1.50 mmol, 1.0 equiv) in THF (6.0 mL), cooled to 0 °C, was added Turbo Grignard (isopropyl magnesium chloride lithium chloride complex, 1.3 M in THF) (1.15 mL, 1.50 mmol, 1.0 equiv) dropwise over 20 min. The yellow reaction mixture was stirred for a further 10 min at 0 °C, before adding cyclobutanone (neat, 0.11 mL, 105 mg, 1.50 mmol, 1.0 equiv) dropwise over 5 min. The reaction mixture was stirred at 0 °C for 60 min, quenched with sat. aq. NH_4Cl (5 mL) and extracted with EtOAc (3x 15 mL). The combined organic layers were washed with brine and dried over $MgSO_4$ before concentrating *in vacuo*. The crude residue (507 mg) was purified by flash column chromatography (pentane:acetone 85:15 to 75:25) to give **1p** (165 mg, 0.59 mmol, 40%) as a yellow-white solid.

TLC (SiO_2; pentane:acetone 4:1, UV, vanilin): R_f = 0.53. **^{1}H NMR** (500 MHz, $CDCl_3$) δ 8.82 (d, J = 4.5 Hz, 1H), 8.48 (d, *J* = 2.2 Hz, 1H), 7.97 (d, *J* = 8.9 Hz, 1H), 7.76 (dd, *J* = 8.9, 2.2 Hz, 1H), 7.37 (d, *J* = 4.5 Hz, 1H), 2.82 – 2.73 (m, 2H), 2.64 – 2.56 (m, 2H), 2.23 – 2.13 (m, 1H), 1.77 – 1.66 (m, 1H). **^{13}C NMR** (126 MHz, $CDCl_3$) δ 150.3, 148.9, 147.8, 132.8, 131.9, 128.9, 127.3, 120.7, 118.0, 77.3, 36.6, 14.3. **IR** (thin film): υ = 3233, 2923, 1589, 1494, 1463, 1248, 1156, 1121, 846 cm^{-1}. **HRMS** (ESI) m/z: $[M+H]^+$ Calcd for $C_{13}H_{13}BrNO$ 278.0175; Found: 278.0174. **mp** = 183 – 185 °C. (see NMR spectra)

2.3.17. 1-(6-fluoroquinolin-4-yl)cyclobutan-1-ol (1q)

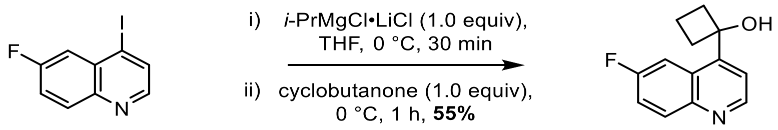

6-fluoro-4-iodoquinoline (**SI-1**) **1q**

To a solution of 6-fluoro-4-iodoquinoline (**SI-1**, 463 mg, 1.70 mmol, 1.0 equiv) in THF (6.8 mL), cooled to 0 °C, was added Turbo Grignard (isopropyl magnesium chloride lithium chloride complex, 1.3 M in THF) (1.30 mL, 1.70 mmol, 1.0 equiv) dropwise over 20 min. The yellow reaction mixture was stirred for a further 10 min at 0 °C, before adding cyclobutanone (neat, 0.124 mL, 119 mg, 1.70 mmol, 1.0 equiv) dropwise over 5 min. The reaction

mixture was stirred at 0 °C for 60 min, quenched with sat. aq. NH_4Cl (5 mL) and extracted with EtOAc (3x 15 mL). The combined organic layers were washed with brine and dried over $MgSO_4$ before concentrating *in vacuo*. The crude residue (406 mg) was purified by flash column chromatography (pentane:acetone 80:20) to give **1q** (204 mg, 0.94 mmol, 55%) as an off-white solid.

TLC (SiO_2; pentane:acetone 4:1, UV, vanilin): R_f = 0.32. **^{1}H NMR** (400 MHz, $CDCl_3$) δ 8.73 – 8.58 (m, 1H), 8.07 – 7.99 (m, 1H), 7.94 (dd, *J* = 10.6, 2.8 Hz, 1H), 7.48 – 7.37 (m, 1H), 7.34 – 7.28 (m, 1H), 3.33 (br s, 1H), 2.80 – 2.68 (m, 2H), 2.64 – 2.50 (m, 2H), 2.22 – 2.05 (m, 1H), 1.76 – 1.61 (m, 1H). **^{13}C NMR** (101 MHz, $CDCl_3$) δ 160.0 (d, *J* = 248.4 Hz), 149.1 (2x), 146.3, 132.5, 127.0, 119.5 (d, *J* = 25.7 Hz), 117.8, 110.4 (d, *J* = 23.6 Hz), 77.4, 36.5, 14.2. **IR** (thin film): υ = 3213, 2927, 2854, 1624, 1513, 1459, 1245, 1223, 1148, 922, 866, 849 cm^{-1}. **HRMS** (ESI) m/z: $[M+H]^+$ Calcd for $C_{13}H_{13}FNO$ 218.0976; Found 218.0975. **mp** = 148 – 151 °C. (see NMR spectra)

2.3.18. 1-(6-methoxyquinolin-4-yl)cyclobutan-1-ol (1r)

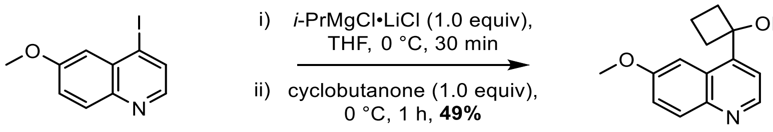

4-iodo-6-methoxyquinoline (**SI-2**) **1r**

To a solution of 4-iodo-6-methoxyquinoline (**SI-2**, 208 mg, 0.73 mmol, 1.0 equiv) in THF (3.0 mL), cooled to 0 °C, was added Turbo Grignard (isopropyl magnesium chloride lithium chloride complex, 1.3 M in THF) (0.56 mL, 0.73 mmol, 1.0 equiv) dropwise over 20 min. The yellow reaction mixture was stirred for a further 10 min at 0 °C, before adding cyclobutanone (neat, 54 µL, 51 mg, 0.73 mmol, 1.0 equiv) dropwise over 5 min. The reaction mixture was stirred at 0 °C for 60 min, quenched with sat. aq. NH_4Cl (5 mL) and extracted with EtOAc (3x 15 mL). The combined organic layers were washed with brine and dried over $MgSO_4$ before concentrating *in vacuo*. The crude residue (290 mg) was purified by flash column chromatography (pentane:acetone 90:10 to 70:30) to give **1r** (81.1 mg, 0.35 mmol, 49%) as a white solid.

TLC (SiO_2; pentane:acetone 4:1, UV, vanilin): R_f = 0.19. Mixture of rotamers, NMR data is reported for the major rotamer. **^{1}H NMR** (400 MHz, $CDCl_3$) δ 8.48 (d, *J* = 4.6 Hz, 1H), 7.93 (d, *J* = 9.2 Hz, 1H), 7.57 (d, *J* = 2.8 Hz, 1H), 7.30 (dd, *J* = 9.2, 2.8 Hz, 1H), 7.21 (d, *J* = 4.5 Hz, 1H), 3.90 (s, 3H), 3.59 (br s, 1H), 2.81 – 2.69 (m, 2H), 2.65 – 2.53 (m, 2H), 2.33 – 2.05 (m, 1H), 1.77 – 1.62 (m, 1H). **^{13}C NMR** (101 MHz, $CDCl_3$) δ 157.3, 148.5, 147.1, 144.8, 131.1, 127.2, 121.8, 117.5, 104.7, 77.4, 55.6, 36.3, 14.4. **IR** (thin film): υ = 3206, 2923, 2852, 1739, 1621, 1509, 1471, 1431, 1362, 1230, 1151, 1030, 849 cm^{-1}. **HRMS** (ESI) m/z: $[M+H]^+$ Calcd for $C_{14}H_{16}NO_2$ 230.1176; Found: 230.1178. **mp** = 179 – 185 °C. (see NMR spectra)

2.3.19. 1-(6,7-dimethoxyquinolin-4-yl)cyclobutan-1-ol (1s)

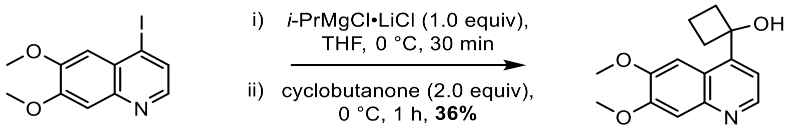

4-iodo-6,7-dimethoxyquinoline (**SI-3**) **1s**

To a solution of 4-iodo-6,7-dimethoxyquinoline (**SI-3**, 426 mg, 1.35 mmol, 1.0 equiv) in THF (5.4 mL), cooled to 0 °C, was added Turbo Grignard (isopropyl magnesium chloride lithium chloride complex, 1.3 M in THF) (1.04 mL, 1.35 mmol, 1.0 equiv) dropwise over 20 min. The brown reaction mixture was stirred for a further 10 min at 0 °C, before adding cyclobutanone (neat, 99 µL, 95 mg, 1.35 mmol, 1.0 equiv) dropwise over 5 min. The reaction mixture was stirred at 0 °C for 30 min. Then additional cyclobutanone (neat, 99 µL, 95 mg, 1.35 mmol, 1.0 equiv) was added dropwise. The reaction mixture was stirred at 0 °C for additional 30 min, quenched with sat. aq. NH_4Cl (5 mL) and extracted with EtOAc (3x 15 mL). The combined organic layers were washed with brine and dried over $MgSO_4$ before concentrating *in vacuo*. The crude residue (516 mg) was purified by flash column chromatography (pentane:acetone 70:30 to 25:75) to give **1s** (126 mg, 0.49 mmol, 36%) as a white solid.

TLC (SiO_2; pentane:acetone 7:3, UV, vanilin): R_f = 0.15. Mixture of rotamers, NMR data is reported for the major rotamer. **^{1}H NMR** (500 MHz, $CDCl_3$) δ 8.60 (d, *J* = 4.5 Hz, 1H), 7.56 (s, 1H), 7.41 (s, 1H), 7.20 (d, *J* = 4.6 Hz, 1H), 4.01 (s, 3H), 4.00 (s, 3H), 2.84 – 2.75 (m, 2H), 2.65 – 2.54 (m, 2H), 2.26 – 2.09 (m, 1H), 1.77 – 1.66 (m, 1H). **^{13}C NMR** (126 MHz, $CDCl_3$) δ 152.0, 149.2, 147.8, 147.7, 146.6, 121.5, 115.8, 108.7, 104.4, 77.6, 56.2, 56.1, 36.3, 14.4. **IR** (thin film): υ = 2923, 2852, 1724, 1621, 1589, 1506, 1434,1249, 1141, 1016, 857 cm^{-1}. **HRMS** (ESI) m/z: $[M+H]^+$ Calcd for $C_{15}H_{18}NO_3$ 260.1281; Found 260.1284. **mp** = decomposition at 204 °C. (see NMR spectra)

2.3.20. 1-(2-fluoropyridin-4-yl)cyclobutan-1-ol (1t)

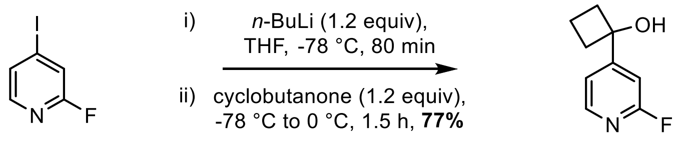

2-fluoro-4-iodopyridine **1t**

To a solution of 2-fluoro-4-iodopyridine (1.0 g, 4.48 mmol, 1.0 equiv) in THF (10 mL), cooled to -78 °C, was added *n*-butyllithium (1.6 M in THF, 3.75 mL, 5.38 mmol, 1.2 equiv) dropwise over 20 minutes. The reaction was stirred for a further 1 h at -78 °C before adding cyclobutanone (0.40 mL, 5.38 mmol, 1.2 equiv) as a solution in THF (4 mL). The reaction was then stirred at -78 °C for 30 minutes before replacing the acetone/dry ice bath with an ice bath and allowing the reaction to warm to 0 °C for 1 h. The reaction was then quenched by addition of aq. sat. NH_4Cl (10 mL) and extracted with EtOAc (3 x 20 mL). The combined organic layers were washed with brine (20 mL) and dried over $MgSO_4$ before concentrating *in vacuo*. The crude residue was purified by flash column chromatography (petroleum ether:acetone 93:7 to 40:60) to give **1t** (575 mg, 3.44 mmol, 77%) as a pale-yellow oil.

^{1}H NMR (400 MHz, $CDCl_3$) δ 8.22 – 8.16 (m, 1H), 7.32 (ddd, *J* = 5.5, 2.0, 1.5 Hz, 1H), 7.07 (td, *J* = 1.5, 0.5 Hz, 1H), 2.57 – 2.47 (m, 2H), 2.46 – 2.36 (m, 2H), 2.24 (s, 1H), 2.17 – 2.04 (m, 1H), 1.83 (m, 1H). **^{13}C NMR** (101 MHz, $CDCl_3$) δ 164.4 (d, *J* = 238 Hz), 161.6 (d, *J* = 7.0 Hz), 147.9 (d, *J* = 15 Hz), 117.8 (d, *J* = 4 Hz), 105.7 (d, *J* = 37.5 Hz), 76.0, 37.6, 13.1. **^{19}F NMR** (101 MHz, $CDCl_3$) δ -67.9. **IR** (thin film): υ 3350, 2947, 1774, 1614, 1557, 1403, 1295, 1141 cm^{-1}. **HRMS** (ESI) m/z: $[M+H]^+$ Calcd for $C_9H_{11}NOF$ 168.0819; Found: 168.0821. (see NMR spectra)

2.4. Substrate Scope for the Dearomative Spirocyclization Reaction

2.4.1. General Procedure A (GP1)

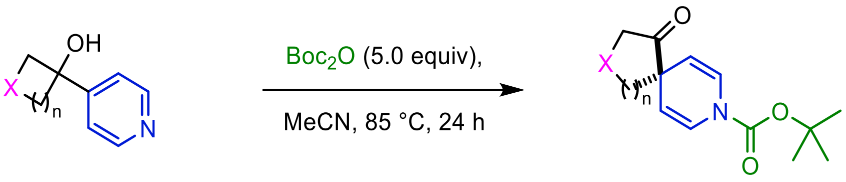

1a-1l **2a-2l**

A flame-dried microwave tube was charged with hydroxycycloalkylpyridine **1a-1l** (0.25 mmol, 1.0 equiv) and anhydrous MeCN (1.4 mL, 0.18 M). To this was added, dropwise at rt, a solution of di-tert-butyl dicarbonate (273 mg, 1.25 mmol, 5.0 equiv) in MeCN (0.7 mL. 1.8 M w.r.t. Boc_2O). The reaction mixture was then heated to 85 °C (oil bath) and stirred for 24 h. The reaction was then quenched with H_2O (5 mL) and extracted with DCM (3 x 5 mL). Organic layers were dried over $MgSO_4$ and concentrated *in vacuo*. Crude residues were purified by flash column chromatography.

2.4.2. General Procedure B (GP2)

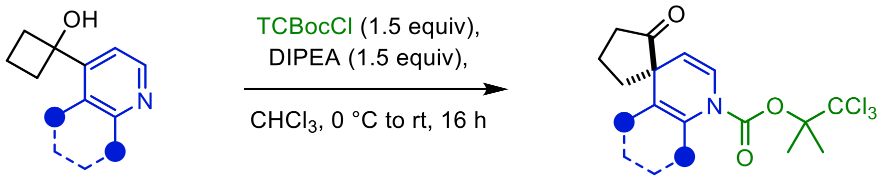

1m-1t **2m-2t**

A flame-dried microwave tube was charged with hydroxycycloalkylpyridine **1m-1t** (0.25 mmol, 1.0 equiv), diisopropylethylamine (DIPEA, 65 µL, 0.375 mmol, 1.5 equiv) and anhydrous $CHCl_3$ (1.4 mL, 0.18 M), and cooled to 0 °C using an ice bath. To this was added, dropwise at 0 °C, a solution of 2,2,2-trichloro-1,1-dimethylethyl chloroformate (TCBocCl, 90.0 mg, 0.375 mmol, 1.5 equiv) in $CHCl_3$ (0.7 mL). The reaction mixture was then warmed to rt and stirred for 16 h. The reaction was then quenched with H_2O (5 mL) and extracted with DCM (3 x 5 mL). Organic layers were dried over $MgSO_4$ and concentrated *in vacuo*. Crude residues were purified by flash column chromatography.

2.4.3. *tert*-butyl 1-oxo-8-azaspiro[4.5]deca-6,9-diene-8-carboxylate (2a)

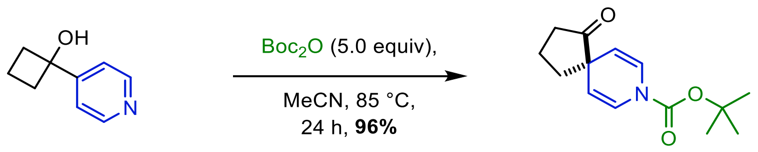

1a **2a**

Synthesized using GP1 with **1a** on a 0.25 mmol scale. Purified by flash column chromatography (petroleum ether/EtOAc 4:1) to give **2a** (57 mg, 0.24 mmol, 96%) as a pale-yellow solid.

^{1}H NMR (400 MHz, $CDCl_3$) δ 7.00 (d, *J* = 8.5 Hz, 1H), 6.87 (d, *J* = 8.5 Hz, 1H), 4.68 (d, *J* = 8.5 Hz, 1H), 4.55 (d, *J* = 8.5 Hz, 1H), 2.33 (td, *J* = 7.2, 3.5 Hz, 2H), 2.06 – 1.84 (m, 4H), 1.49 (s, 9H). **^{13}C NMR** (101 MHz, $CDCl_3$) δ 218.2, 149.7, 124.2, 123.5, 106.4, 106.0, 82.5, 50.4, 40.7, 35.8, 28.1, 18.4. **IR** (thin film): υ = 2975, 1716, 1686, 1367, 1335, 1318, 1163, 1116, 964, 857, 736 cm^{-1}. **HRMS** (ESI) m/z: $[M+Na]^+$ Calcd for $C_{14}H_{19}NO_3Na$ 272.1257; Found 272.1255. **mp** = 85 – 90 °C. (see NMR spectra)

Gram scale:

A 400 mL Schlenk tube was charged with 1-(pyridin-4-yl)cyclobutan-1-ol (**1a**, 1.00 g, 6.7 mmol, 1.0 equiv) and anhydrous MeCN (40 mL). To this was added, dropwise over 10 minutes at rt, a solution of di-tert-butyl dicarbonate (7.8 mL, 33.5 mmol, 5.0 equiv) in MeCN (10 mL). The reaction mixture was then heated to 85 °C (oil bath) and stirred for 24 h. The reaction was then quenched with H_2O (50 mL) and extracted with DCM (3 x 50 mL). Organic layers were dried over $MgSO_4$ and concentrated in vacuo. The crude residue was purified by flash column chromatography (pentane:acetone 98:2 to 80:20) to give **2a** as a pale-yellow solid (1.45 g, 5.83 mmol, 87%).

2.4.4. *tert*-butyl 1-oxo-7-azaspiro[3.5]nona-5,8-diene-7-carboxylate (2b)

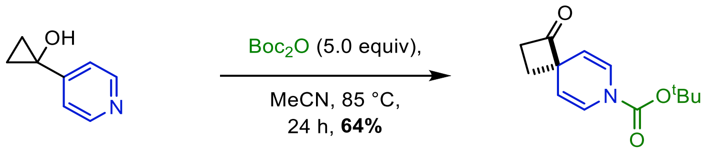

1b **2b**

Synthesized using GP1 with **1b** on a 0.25 mmol scale. Purified by flash column chromatography (pentane:acetone 95:5 to 60:40) to give **2b** (38 mg, 0.16 mmol, 64%) as an off-white solid.

^{1}H NMR (400 MHz, $CDCl_3$) δ 6.93 (m, 1H), 6.79 (m, 1H), 4.90 (m, 1H), 4.80 (m, 1H), 3.02 (ddd, *J* = 8.5, 8.5, 2.5 Hz, 2H), 2.04 (dd, *J* = 8.5, 8.5 Hz, 2H), 1.49 (s, 9H). **^{13}C NMR** (101 MHz, $CDCl_3$) δ 210.3, 149.7, 124.0, 123.5, 106.0, 105.3, 82.8, 64.6, 42.8, 31.2, 28.2. **IR** (thin film): υ = 2979, 1780, 1722, 1681, 1371, 1336, 1319, 1164, 1134 cm^{-1}. **HRMS** (APCI) m/z: $[M+H]^+$ Calcd for $C_{13}H_{18}NO_3$ 236.1281; Found: 236.1289. **mp** = 93 – 98 °C. (see NMR spectra)

2.4.5. *tert*-butyl 7-oxo-3-azaspiro[5.5]undeca-1,4-diene-3-carboxylate (2c)

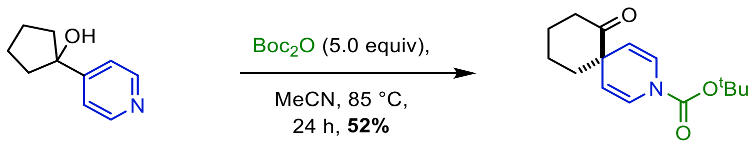

1c **2c**

Synthesized using GP1 with **1c** on a 0.25 mmol scale. Purified by flash column chromatography (pentane:acetone 95:5) to give **2c** (34 mg, 0.13 mmol, 52%) as a pale-yellow oil.

TLC (SiO_2; pentane:acetone 9:1, UV, vanilin): R_f = 0.53. **^{1}H NMR** (500 MHz, $CDCl_3$) δ 7.01 – 6.74 (m, 2H), 5.15 – 4.74 (m, 2H), 2.59 – 2.32 (m, 2H), 2.01 – 1.63 (m, 6H), 1.48 (s, 9H). **^{13}C NMR** (126 MHz, $CDCl_3$) δ 210.9, 150.0, 123.4, 123.1, 107.2, 82.5, 50.1, 43.5, 38.0, 28.2, 27.8, 19.9. **IR** (thin film): υ = 2921, 2851, 1721, 1456, 1371, 1340, 1321, 1164, 1138, 1123 cm^{-1}. **HRMS** (ESI) m/z: $[M+Na]^+$ Calcd for $C_{15}H_{21}NO_3Na$ 286.1414; Found 286.1422. (see NMR spectra)

2.4.6. *tert*-butyl 4-oxo-2-oxa-8-azaspiro[4.5]deca-6,9-diene-8-carboxylate (2e)

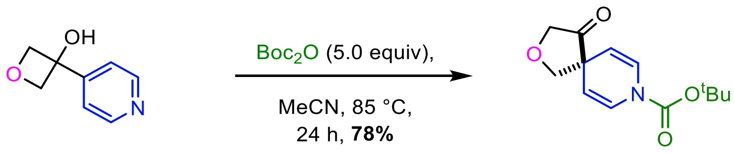

1e **2e**

Synthesized using GP1 with **1e** on a 0.25 mmol scale. Purified by flash column chromatography (pentane:acetone 95:5 to 60:40) to give **2e** (49 mg, 0.19 mmol, 78%) as a pale-yellow oil.

^{1}H NMR (400 MHz, $CDCl_3$) δ 7.08 (m, 1H), 6.95 (m, 1H), 4.75 (s, 1H), 4.64 (s, 1H), 4.06 (s, 2H), 3.92 (s, 2H), 1.50 (s, 9H). **^{13}C NMR** (101 MHz, $CDCl_3$) δ 213.4, 149.6, 125.8, 125.2, 103.3, 102.6, 83.0, 79.7, 69.7, 50.4, 28.2. **IR** (thin film): υ = 2978, 2870, 1762, 1720, 1333, 1317, 1166, 1129 cm^{-1}. **HRMS** (APCI) m/z: $[M+H]^+$ Calcd for $C_{13}H_{18}NO_4$ 252.1230; Found 252.1240. (see NMR spectra)

2.4.7. di-*tert*-butyl 4-oxo-2,8-diazaspiro[4.5]deca-6,9-diene-2,8-dicarboxylate (2f)

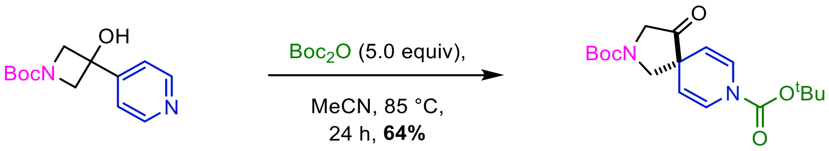

1f **2f**

Synthesized using GP1 with **1f** on a 0.25 mmol scale. Purified by flash column chromatography (pentane:acetone 95:5 to 60:40) to give **2f** (56 mg, 0.16 mmol, 64%) as an off-white solid.

^{1}H NMR (400 MHz, $CDCl_3$) δ 7.07 (m, 1H), 6.95 (m, 1H), 4.72 (m, 1H), 4.61 (m, 1H), 3.90 (m, 2H), 3.55 (s, 2H), 1.50 (s, 9H), 1.48 (s, 9H). **^{13}C NMR** (101 MHz, $CDCl_3$) δ 209.8,154.5, 149.6, 125.6, 124.9, 103.9, 103.1, 83.1, 80.8, 57.4, 51.0, 50.5, 28.5, 28.2. **IR** (thin film): υ = 2978, 1763, 1699, 1687, 1368, 1334, 1317, 1160, 1127 cm^{-1}. **HRMS** (ESI) m/z: $[M+H]^+$ Calcd for $C_{18}H_{27}N_2O_5$ 351.1914; Found 351.1922. **mp** = 127 – 132 °C. (see NMR

spectra)

2.4.8. *tert*-butyl-2-oxo-3,3a,6,6a-tetrahydro-1'*H*,2*H*-spiro[pentalene-1,4'-pyridine]-1'-carboxylate (2g)

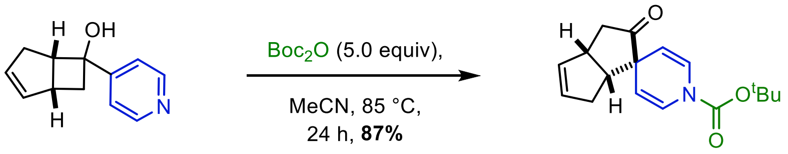

1g **2g**

Synthesized using GP1 with **1g** on a 0.25 mmol scale. Purified by flash column chromatography (pentane:acetone 95:5 to 60:40) to give **2g** (63 mg, 0.22 mmol, 87%) as a viscous orange oil.

^{1}H NMR (400 MHz, $CDCl_3$) δ 7.01 (d, *J* = 7.5 Hz, 1H), 6.89 (d, *J* = 7.5 Hz, 1H), 5.77 – 5.72 (m, 2H), 4.63 – 4.61 (m, 1H), 4.53 – 4.52 (m, 1H), 3.39 – 3.33 (m, 1H), 2.69 – 2.29 (m, 5H), 1.49 (s, 9H). Mixture of rotamers, NMR data is reported for both rotamers. **^{13}C NMR** (101 MHz, $CDCl_3$) δ 217.0, 168.6, 149.8, 134.5, 131.9, 125.0, 124.4, 124.3, 123.5, 107.8, 107.1, 104.6, 104.0, 82.6, 82.4, 57.8, 54.2, 51.7, 51.3, 42.5, 42.3, 38.8, 33.8, 28.2, 28.2, 28.0, 27.5. **IR** (thin film): υ = 2977, 1716, 1684, 1368, 1318, 1161, 1121, 971, 739 cm^{-1}. **HRMS** (APCI) m/z: [M-Boc+H]$^+$ Calcd for $C_{12}H_{13}NO$ 188.1070; Found: 188.1070. (see NMR spectra)

2.4.9. *tert*-butyl 6-fluoro-1-oxo-8-azaspiro[4.5]deca-6,9-diene-8-carboxylate (2h)

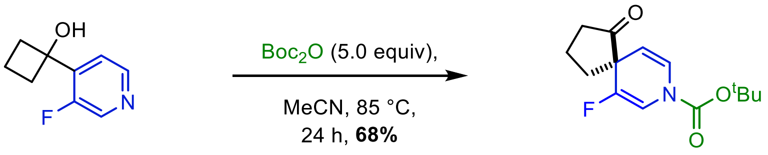

1h **2h**

Synthesized using GP1 with **1h** on a 0.25 mmol scale. Purified by flash column chromatography (petroleum ether:EtOAc 4:1) to give **2h** (46 mg, 0.17 mmol, 68%) as a yellow oil.

Mixture of rotamers (0.56:0.44), NMR data is repforted for both rotamers.**^{1}H NMR** (500 MHz, $CDCl_3$) δ 7.11 (d, *J* = 10.5 Hz, 1/2H), 6.97 (d, *J* = 10.6 Hz, 1/2H), 6.94 (d, *J* = 8.4 Hz, 1/2H), 6.79 (d, *J* = 8.3 Hz, 1/2H), 4.75 (t, *J* = 9.2 Hz, 1/2H), 4.62 (t, *J* = 9.1 Hz, 1/2H), 2.47 – 2.25 (m, 3H), 2.12 – 1.88 (m, 3H), 1.49 (s, 9H). **^{13}C NMR** (126 MHz, $CDCl_3$) δ 216.4, 216.1, 149.3 (d, *J* = 39.6 Hz), 148.8 (d, *J* = 244.8 Hz), 147.7 (d, *J* = 244.9 Hz), 123.2, 122.7, 109.9 (d, *J* = 43.7 Hz), 109.1 (d, *J* = 44.0 Hz), 106.5 (d, *J* = 11.0 Hz), 106.2 (d, *J* = 10.8 Hz), 83.1, 52.9, 52.7, 37.0, 36.8, 36.6, 36.3, 28.2, 19.2, 19.0. **IR** (thin film): υ = 2973, 2932, 1749, 1718, 1399, 1371, 1331, 1313, 1157, 1103 cm^{-1}. **HRMS** (APCI) m/z: [M+H]$^+$ Calcd for $C_{14}H_{19}NO_3F$ 268.1343; Found 268.1344. (see NMR spectra)

2.4.10. *tert*-butyl 6-bromo-1-oxo-8-azaspiro[4.5]deca-6,9-diene-8-carboxylate (2i)

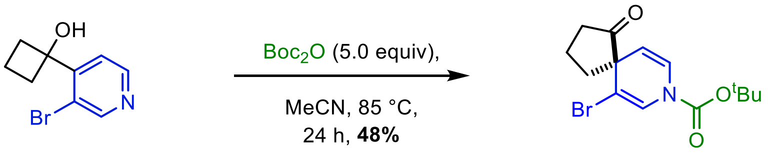

1i **2i**

Synthesized using GP1 with **1i** on a 0.25 mmol scale. Purified by flash column chromatography (pentane:acetone 9:1) to give **2i** (39 mg, 0.12 mmol, 48%) as a pale-yellow oil.

TLC (SiO_2; pentane:acetone 9:1, UV, vanilin): R_f = 0.46. **^{1}H NMR** (500 MHz, $CDCl_3$) δ 7.47 – 7.17 (m, 1H), 7.01 – 6.66 (m, 1H), 4.80 – 4.49 (m, 1H), 2.58 – 2.46 (m, 1H), 2.44 – 2.34 (m, 1H), 2.32 – 2.20 (m, 1H), 2.14 – 2.04 (m, 1H), 1.99 – 1.86 (m, 2H), 1.49 (s, 9H). Mixture of rotamers, NMR data is reported for both rotamers. **^{13}C NMR** (126 MHz, $CDCl_3$) δ 216.2, 216.0, 148.8, 126.5, 125.9, 122.3, 121.8, 105.68, 105.5, 105.3, 104.8, 83.5, 83.4, 55.5, 38.30, 38.2, 36.9, 36.7, 28.2, 18.8. **IR** (thin film): ʋ = 3121, 2975, 2930, 1722, 1680, 1356, 1315, 1150, 990 cm^{-1}. **HRMS** (ESI) m/z: $[M+H]^+$ Calcd for $C_{14}H_{19}BrNO_3$ 328.0543; Found 328.0553. (see NMR spectra)

2.4.11. *tert*-butyl 6-(diisopropylcarbamoyl)-1-oxo-8-azaspiro[4.5]deca-6,9-diene-8-carboxylate (2j)

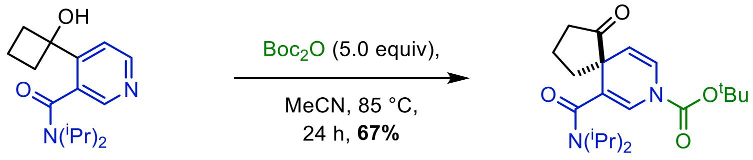

1j **2j**

Synthesized using GP1 with **1j** on a 0.25 mmol scale. Purified by flash column chromatography (petroleum ether:EtOAc 1:3) to give **2j** (64 mg, 0.17 mmol, 67%) as a colorless oil.

^{1}H NMR (400 MHz, $CDCl_3$) δ 7.03 (m, 1H), 6.86 (m, 1H), 4.66 (m, 1H), 2.59 – 2.44 (m, 1H), 2.33 – 2.08 (m, 2H), 1.99 – 1.90 (m, 3H), 1.90 – 1.80 (m, 2H), 1.48 (s, 9H), 1.34 – 1.20 (m, 12H). Mixture of rotamers, NMR data is reported for both rotamers. **^{13}C NMR** (101 MHz, $CDCl_3$) δ 217.5, 217.3, 168.4, 149.6, 149.4, 124.2, 123.3, 122.7, 122.1, 116.5, 115.6, 109.1, 108.6, 83.1, 50.7, 48.6, 48.5, 38.9, 38.7, 36.6, 36.5, 29.8, 28.2, 21.2, 20.7, 19.4, 14.3. **IR** (thin film): ʋ = 2969, 1721, 1615, 1365, 1296, 1146, 760 cm^{-1}. **HRMS** (ESI) m/z: $[M+H]^+$ Calcd for $C_{21}H_{33}N_2O_4$ 377.2435; Found: 377.2428. (see NMR spectra)

2.4.12. *tert*-butyl 1-oxo-6-phenyl-8-azaspiro[4.5]deca-6,9-diene-8-carboxylate (2k)

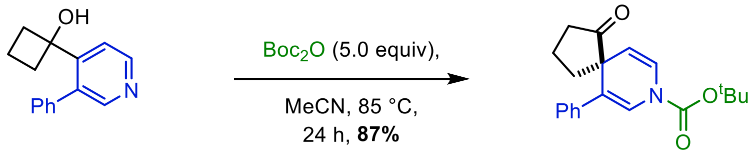

1k **2k**

Synthesized using GP1 with **1k** on a 0.25 mmol scale. Purified by flash column chromatography (pentane:acetone 95:5) to give **2k** (70 mg, 0.22 mmol, 87%) as an off-white foam.

TLC (SiO_2; pentane:acetone 9:1, UV, $KMnO_4$): R_f = 0.60. **^{1}H NMR** (500 MHz, $CDCl_3$) δ 7.33 – 7.20 (m, 3H), 7.15 – 7.10 (m, 2H), 7.10 – 6.81 (m, 2H), 4.72 (dd, *J* = 59.7, 8.4 Hz, 1H), 2.46 – 2.35 (m, 1H), 2.23 – 2.02 (m, 2H), 1.92 – 1.77 (m, 2H), 1.72 – 1.60 (m, 1H), 1.49 (s, 9H). Mixture of rotamers, NMR data is reported for both rotamers. **^{13}C NMR** (126 MHz, $CDCl_3$) δ 218.8, 149.9, 138.8, 138.6, 129.4, 128.5, 127.4, 123.9, 123.5, 122.8, 122.4, 120.0, 119.3, 107.1, 106.7, 82.8, 54.2, 37.4, 37.2, 37.0, 36.9, 28.2, 18.7. **IR** (thin film): ʋ = 2974, 1717,

1355, 1330, 1254, 1160, 1123, 992 cm^{-1}. **HRMS** (APCI) m/z: [M-Boc+H]$^+$ Calcd for $C_{15}H_{15}NO$ 226.1226; Found 226.1225. (see NMR spectra)

2.4.13. *tert*-butyl 7-methyl-1-oxo-8-azaspiro[4.5]deca-6,9-diene-8-carboxylate (2l)

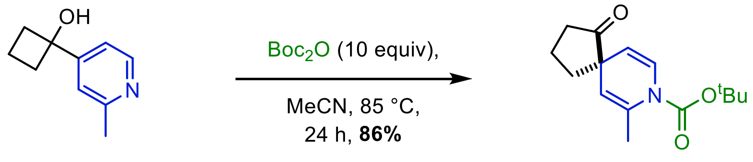

1l **2l**

Synthesized using a modified GP1 with **1l** on a 0.25 mmol scale. 5.0 equiv Boc_2O added at start, a further 5 equiv added after 10 h. Purified by flash column chromatography (petroleum ether:EtOAc 5:1) to give **2l** (32 mg, 0.22 mmol, 86%) as a pale-yellow oil.

^{1}**H NMR** (400 MHz, $CDCl_3$) δ 7.01 (m, 1H), 4.65 (m, 1H), 4.44 (m, 1H), 2.29 (m, 2H), 2.17 (s, 3H), 1.94 (m, 2H), 1.86 (m, 2H), 1.48 (s, 9H). ^{13}**C NMR** (101 MHz, $CDCl_3$) δ 218.3, 150.9, 134.6, 127.0, 108.5, 107.0, 82.4, 51.4, 40.1, 35.9, 28.3, 23.0, 18.4. **IR** (thin film): υ = 2977, 1743, 1613, 1403, 1285, 1252, 1165, 1127, 1111 cm^{-1}. **HRMS** (ESI) m/z: [M+Na]$^+$ Calcd for $C_{15}H_{21}NO_3Na$ 286.1414; Found: 286.1409. (see NMR spectra)

2.4.14. 1,1,1-trichloro-2-methylpropan-2-yl 1-oxo-7-phenyl-8-azaspiro[4.5]deca-6,9-diene-8-carboxylate (2m)

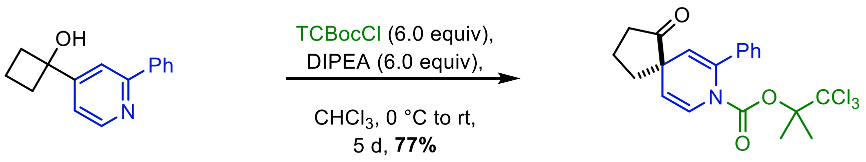

1m **2m**

Synthesized using a modified GP2 with **1m** on a 0.25 mmol scale. After 16 h additional diisopropylethylamine (DIPEA, 65 µL, 0.375 mmol, 1.5 equiv) and 2,2,2-Trichloro-1,1-dimethylethyl chloroformate (TCBocCl, 90.0 mg, 0.375 mmol, 1.5 equiv) in $CHCl_3$ (0.7 mL) were added to the reaction mixture. After 3.5 d another diisopropylethylamine (DIPEA, 130 µL, 0.750 mmol, 3.0 equiv) and 2,2,2-Trichloro-1,1-dimethylethyl chloroformate (TCBocCl, 180 mg, 0.750 mmol, 3.0 equiv) in $CHCl_3$ (1.4 mL) were added to the reaction mixture. The reaction mixture was stirred at rt for 5 d. Purified by flash column chromatography (pentane:acetone 95:5) to give **2m** (82.7 mg, 0.19 mmol, 77%) as an off-white foam.

TLC (SiO_2; pentane:acetone 9:1, UV, vanilin): R_f = 0.37. ^{1}**H NMR** (400 MHz, $CDCl_3$) δ 7.31 – 7.22 (m, 5H), 7.12 (d, *J* = 7.6 Hz, 1H), 4.95 (d, *J* = 2.1 Hz, 1H), 4.90 (dd, *J* = 7.5, 2.1 Hz, 1H), 2.42 – 2.24 (m, 2H), 2.06 – 1.93 (m, 4H), 1.84 (s, 3H), 1.82 (s, 3H). ^{13}**C NMR** (101 MHz, $CDCl_3$) δ 216.0, 149.1, 139.0, 137.7, 128.2, 128.0, 127.6, 126.2, 115.3, 111.3, 106.0, 90.3, 52.3, 39.0, 36.0, 21.6, 21.5, 18.8. **IR** (thin film): υ = 2956, 2924, 2854, 1739, 1675, 1351, 1310, 1150, 1107, 981, 795, 757, 729, 697 cm^{-1}. **HRMS** (ESI) m/z: [M+H]$^+$ Calcd for $C_{20}H_{21}Cl_3NO_3$ 428.0582; Found: 428.0572. (see NMR spectra)

2.4.15. 1,1,1-trichloro-2-methylpropan-2-yl 2-oxo-1'*H*-spiro[cyclopentane-1,4'-quinoline]-1'-carboxylate

(2n)

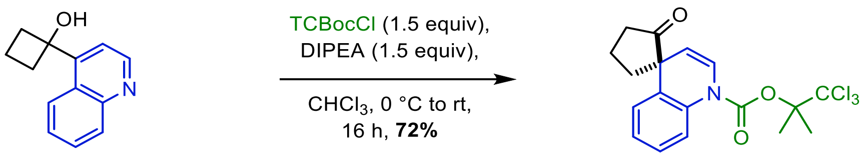

1n **2n**

Synthesized using GP2 with **1n** on a 0.25 mmol scale. Purified by flash column chromatography (pentane:acetone 95:5) to give **2n** (72.3 mg, 0.18 mmol, 72%) as a colorless oil.

TLC (SiO_2; pentane:acetone 9:1, UV, vanilin): R_f = 0.43. **^{1}H NMR** (400 MHz, $CDCl_3$) δ 8.11 (dd, *J* = 8.5, 1.3 Hz, 1H), 7.31 – 7.23 (m, 1H), 7.18 – 7.10 (m, 2H), 7.00 (dd, *J* = 7.8, 1.6 Hz, 1H), 5.13 (d, *J* = 7.7 Hz, 1H), 2.56 – 2.35 (m, 3H), 2.23 – 2.06 (m, 3H), 2.03 (s, 6H). **^{13}C NMR** (101 MHz, $CDCl_3$) δ 216.8, 149.8, 136.1, 130.4, 127.2, 126.8, 125.8, 125.3, 122.1, 112.0, 106.2, 90.4, 53.2, 38.9, 37.1, 21.7, 19.0. **IR** (thin film): υ = 2956, 2924, 2854, 1727, 1487, 1456, 1335, 1146, 1025, 788, 757 cm^{-1}. **HRMS** (ESI) m/z: $[M+H]^+$ Calcd for $C_{18}H_{19}Cl_3NO_3$ 402.0425; Found 402.0442. (see NMR spectra)

2.4.16. 1,1,1-trichloro-2-methylpropan-2-yl 7'-chloro-2-oxo-1'*H*-spiro[cyclopentane-1,4'-quinoline]-1'-carboxylate (2o)

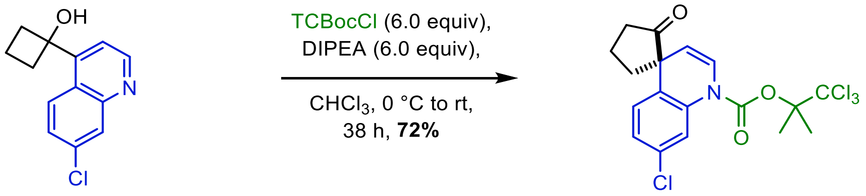

1o **2o**

Synthesized using a modified GP2 with **1o** on a 0.25 mmol scale. After 16 h additional diisopropylethylamine (DIPEA, 65 µL, 0.375 mmol, 1.5 equiv) and 2,2,2-Trichloro-1,1-dimethylethyl chloroformate (TCBocCl, 90.0 mg, 0.375 mmol, 1.5 equiv) in $CHCl_3$ (0.7 mL) were added to the reaction mixture. After 22 h another diisopropylethylamine (DIPEA, 130 µL, 0.750 mmol, 3.0 equiv) and 2,2,2-Trichloro-1,1-dimethylethyl chloroformate (TCBocCl, 180 mg, 0.750 mmol, 3.0 equiv) in $CHCl_3$ (1.4 mL) were added to the reaction mixture. The reaction mixture was stirred at rt for 38 h. Purified by flash column chromatography (pentane:acetone 95:5) to give **2o** (78.5 mg, 0.18 mmol, 72%) as an off-white foam.

TLC (SiO_2; pentane:acetone 4:1, UV, vanilin): R_f = 0.81. **^{1}H NMR** (500 MHz, $CDCl_3$) δ 8.36 (d, *J* = 2.2 Hz, 1H), 7.29 – 7.25 (m, 1H), 7.25 (d, *J* = 7.9 Hz, 1H), 7.07 (d, *J* = 8.4 Hz, 1H), 5.27 (d, *J* = 7.8 Hz, 1H), 2.70 – 2.61 (m, 1H), 2.58 – 2.45 (m, 2H), 2.38 – 2.30 (m, 1H), 2.30 – 2.22 (m, 2H), 2.18 (s, 6H). **^{13}C NMR** (126 MHz, $CDCl_3$) δ 216.2, 149.5, 137.1, 133.0, 128.5, 126.9, 126.4, 125.4, 122.1, 111.5, 106.0, 90.8, 53.0, 38.9, 36.8, 21.7, 18.9. **IR** (thin film): υ = 2955, 2923, 2853, 1729, 1488, 1333, 1149, 800 cm^{-1}. **HRMS** (APCI) m/z: $[M+H]^+$ Calcd for $C_{18}H_{18}Cl_4NO_3$ 436.0035; Found: 436.0028. (see NMR spectra)

2.4.17. 1,1,1-trichloro-2-methylpropan-2-yl 6'-bromo-2-oxo-1'*H*-spiro[cyclopentane-1,4'-quinoline]-1'-

carboxylate (2p)

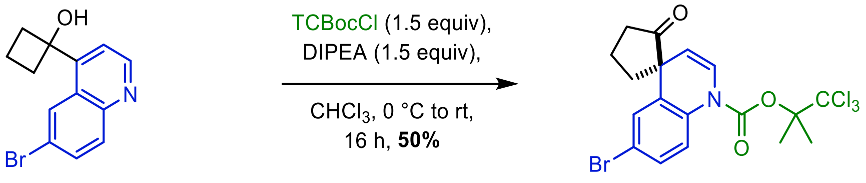

1p **2p**

Synthesized using GP2 with **1p** on a 0.25 mmol scale. Purified by flash column chromatography (pentane:acetone 95:5) to give **2p** (60.7 mg, 0.13 mmol, 50%) as an off-white foam.

TLC (SiO_2; pentane:acetone 9:1, UV, vanilin): R_f = 0.56. ^{1}**H NMR** (400 MHz, $CDCl_3$) δ 8.03 (d, *J* = 9.0 Hz, 1H), 7.42 – 7.32 (m, 1H), 7.15 – 7.06 (m, 2H), 5.10 (d, *J* = 7.8 Hz, 1H), 2.58 – 2.31 (m, 3H), 2.26 – 2.06 (m, 3H), 2.02 (s, 6H). ^{13}**C NMR** (101 MHz, $CDCl_3$) δ 215.7, 149.6, 135.4, 132.3, 130.2, 128.7, 126.7, 123.6, 118.3, 111.5, 106.1, 90.7, 53.20, 38.6, 36.8, 21.7, 18.9. **IR** (thin film): υ = 2956, 2923, 2853, 1729, 1481, 1336, 1146, 1024, 797 cm^{-1}. **HRMS** (MALDI) m/z: $[M+Na]^+$ Calcd for $C_{18}H_{17}BrCl_3NO_3Na$ 503.9326 (most abundant isotope peak); Found: 503.9337. (see NMR spectra)

2.4.18. 1,1,1-trichloro-2-methylpropan-2-yl 6'-fluoro-2-oxo-1'*H*-spiro[cyclopentane-1,4'-quinoline]-1'-carboxylate (2q)

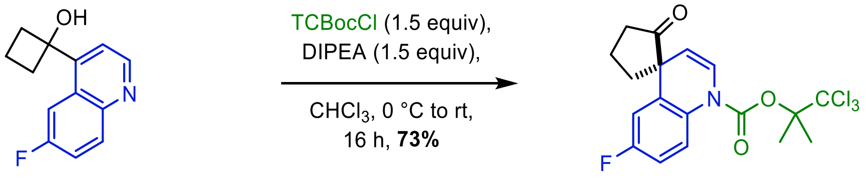

1q **2q**

Synthesized using GP2 with **1q** on a 0.25 mmol scale. Purified by flash column chromatography (pentane:acetone 95:5) to give **2q** (76.3 mg, 0.18 mmol, 73%) as an off-white foam.

TLC (SiO_2; pentane:acetone 9:1, UV, vanilin): R_f = 0.60. ^{1}**H NMR** (400 MHz, $CDCl_3$) δ 8.15 – 8.05 (m, 1H), 7.11 (d, *J* = 7.7 Hz, 1H), 7.02 – 6.92 (m, 1H), 6.72 (dd, *J* = 9.3, 2.9 Hz, 1H), 5.10 (d, *J* = 7.7 Hz, 1H), 2.57 – 2.31 (m, 3H), 2.25 – 2.07 (m, 3H), 2.02 (s, 6H). ^{13}**C NMR** (101 MHz, $CDCl_3$) δ 215.7, 160.0 (d, *J* = 244.6 Hz), 149.8, 132.4, 132.3, 126.9, 123.7 (d, *J* = 7.9 Hz), 114.1 (d, *J* = 22.4 Hz), 112.3 (d, *J* = 23.2 Hz), 111.4, 106.2, 90.5, 53.4, 38.5, 36.8, 21.7, 18.9. **IR** (thin film): υ = 2956, 2923, 2853, 1725, 1492, 1343, 1287, 1155, 1026, 798 cm^{-1}. **HRMS** (ESI) m/z: $[M+H]^+$ Calcd for $C_{18}H_{18}Cl_3FNO_3$ 420.0331; Found: 420.0345. (see NMR spectra)

2.4.19. 1,1,1-trichloro-2-methylpropan-2-yl 6'-methoxy-2-oxo-1'*H*-spiro[cyclopentane-1,4'-quinoline]-1'-

carboxylate (2r)

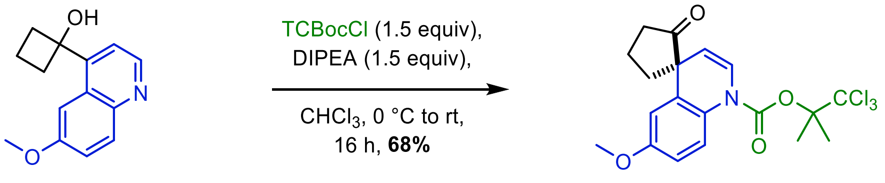

1r **2r**

Synthesized using GP2 with **1r** on a 0.25 mmol scale. Purified by flash column chromatography (pentane:acetone 95:5) to give **2r** (73.4 mg, 0.17 mmol, 68%) as an off-white foam.

TLC (SiO_2; pentane:acetone 9:1, UV, vanilin): R_f = 0.44. **^{1}H NMR** (400 MHz, $CDCl_3$) δ 8.06 (d, *J* = 9.2 Hz, 1H), 7.11 (d, *J* = 7.7 Hz, 1H), 6.81 (dd, *J* = 9.2, 2.9 Hz, 1H), 6.52 (d, *J* = 2.9 Hz, 1H), 5.08 (d, *J* = 7.8 Hz, 1H), 3.78 (s, 3H), 2.56 – 2.34 (m, 3H), 2.22 – 2.06 (m, 3H), 2.02 (s, 6H). **^{13}C NMR** (101 MHz, $CDCl_3$) δ 216.5, 156.8, 149.8, 131.7, 129.6, 126.91, 123.1, 111.9, 111.6, 111.4, 106.3, 90.3, 55.6, 53.4, 38.78, 37.1, 21.8, 21.7, 19.0. **IR** (thin film): υ = 2956, 2924, 2853, 1723, 1496, 1345, 1286, 1141, 1021, 798 cm^{-1}. **HRMS** (ESI) m/z: $[M+H]^+$ Calcd for $C_{19}H_{21}Cl_3NO_4$ 432.0531; Found 432.0531. (see NMR spectra)

2.4.20. 1,1,1-trichloro-2-methylpropan-2-yl 6',7'-dimethoxy-2-oxo-1'*H*-spiro[cyclopentane-1,4'-quinoline]-1'-carboxylate (2s)

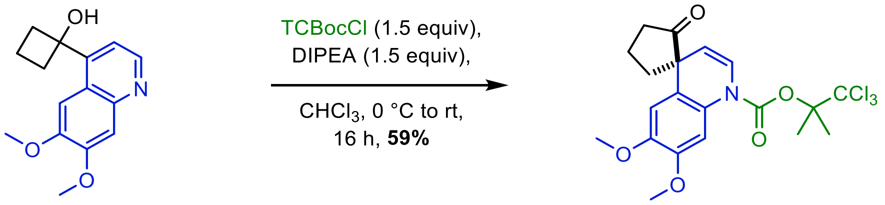

1s **2s**

Synthesized using GP2 with **1s** on a 0.25 mmol scale. Purified by flash column chromatography (pentane:acetone 9:1) to give **2s** (68.5 mg, 0.15 mmol, 59%) as a white solid.

TLC (SiO_2; pentane:acetone 4:1, UV, vanilin): R_f = 0.52. **^{1}H NMR** (400 MHz, $CDCl_3$) δ 7.87 (s, 1H), 7.12 (d, *J* = 7.8 Hz, 1H), 6.43 (s, 1H), 5.07 (d, *J* = 7.8 Hz, 1H), 3.90 (s, 3H), 3.83 (s, 3H), 2.57 – 2.47 (m, 1H), 2.45 – 2.31 (m, 2H), 2.24 – 2.07 (m, 3H), 2.02 (s, 6H). **^{13}C NMR** (101 MHz, $CDCl_3$) δ 216.8, 149.7, 147.8, 146.5, 129.9, 126.6, 121.5, 110.9, 108.7, 106.3, 106.0, 90.3, 56.3, 56.2, 53.0, 39.1, 36.9, 21.8, 21.7, 18.9. **IR** (thin film): υ = 2957, 2924, 2854, 1724, 1513, 1322, 1258, 1156, 1113, 794 cm^{-1}. **HRMS** (ESI) m/z: $[M+H]^+$ Calcd for $C_{20}H_{23}Cl_3NO_5$ 462.0636; Found: 462.0640. **mp** = 162 – 165 °C. (see NMR spectra)

2.5. Activator Scope for Dearomative Spirocyclization Reaction

2.5.1. General Procedure C (GP3)

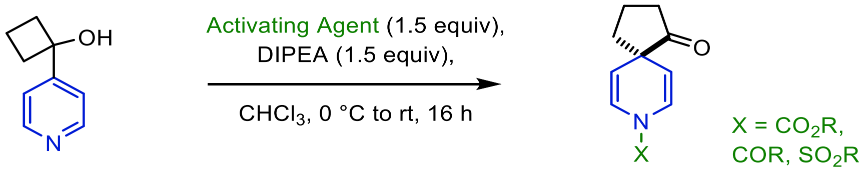

1a **2aa-aj**

A flame-dried microwave tube was charged with 1-(pyridin-4-yl)cyclobutan-1-ol (**1a**, 37.3 mg, 0.25 mmol, 1.0 equiv), diisopropylethylamine (DIPEA, 65 µL, 0.375 mmol, 1.5 equiv) and anhydrous $CHCl_3$ (1.4 mL, 0.18 M), and cooled to 0 °C using an ice bath. To this was added, dropwise at 0 °C, a solution of the activating agent (0.375 mmol, 1.5 equiv) in $CHCl_3$ (0.7 mL). The reaction mixture was then warmed to rt and stirred for 16 h. The reaction was then quenched with H_2O (5 mL) and extracted with DCM (3 x 5 mL). Organic layers were dried over $MgSO_4$ and concentrated *in vacuo*. Crude residues were purified by flash column chromatography.

2.5.2. methyl 1-oxo-8-azaspiro[4.5]deca-6,9-diene-8-carboxylate (2aa)

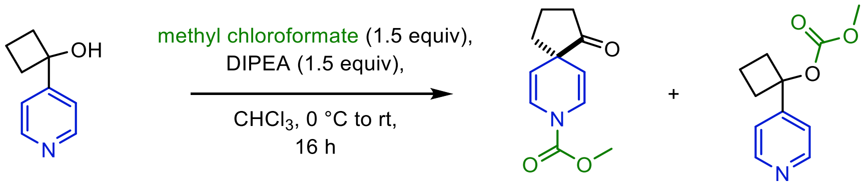

1a **2aa** **57%** **3aa** **27%**

Synthesized using GP3 with **1a** on a 0.25 mmol scale using methyl chloroformate (30 µL, 0.375 mmol, 1.5 equiv). Purified by flash column chromatography (pentane:acetone 95:5 to 60:40) to give **2aa** (29 mg, 0.14 mmol, 57%) as a white solid. In addition, **3aa** (14 mg, 0.07 mmol, 27%) was isolated as a colorless oil.

2aa: **^{1}H NMR** (400 MHz, $CDCl_3$) δ 7.00 (d, J = 8.0 Hz, 1H), 6.94 – 6.82 (d, J = 8.0 Hz, 1H), 4.72 (d, J = 8.0 Hz, 1H), 4.61 (d, J = 8.0 Hz, 1H), 3.80 (s, 3H), 2.32 (m, 2H), 2.05 – 1.81 (m, 4H). **^{13}C NMR** (101 MHz, $CDCl_3$) δ 217.8, 151.7, 123.8, 123.6, 107.3, 107.0, 53.7, 50.4, 40.5, 35.8, 18.5. **IR** (thin film): υ = 2958, 1717, 1687, 1627, 1440, 1368, 1334, 1314, 1213, 1118 cm^{-1}. **HRMS** (ESI) m/z: $[M+H]^+$ Calcd for $C_{11}H_{14}NO_3$ 208.0968; Found 208.0959. (see NMR spectra)

3aa: **^{1}H NMR** (400 MHz, $CDCl_3$) δ 8.62 (m, 2H), 7.36 (m, 2H), 3.68 (s, 3H), 2.72 – 2.51 (m, 4H), 2.12 – 1.93 (m, 1H), 1.78 (m, 2H). **^{13}C NMR** (101 MHz, $CDCl_3$) δ 153.8, 151.1, 150.3, 120.1, 82.3, 54.7, 34.2, 13.7. **IR** (thin film): υ = 2996, 2956, 1747, 1599, 1441, 1271, 1249, 1131, 1056 cm^{-1}. **HRMS** (ESI) m/z: $[M+H]^+$ Calcd for $C_{11}H_{14}NO_3$ 208.0968; Found 208.0963. (see NMR spectra)

2.5.3. 2,2,2-trichloroethyl 1-oxo-8-azaspiro[4.5]deca-6,9-diene-8-carboxylate (2ab)

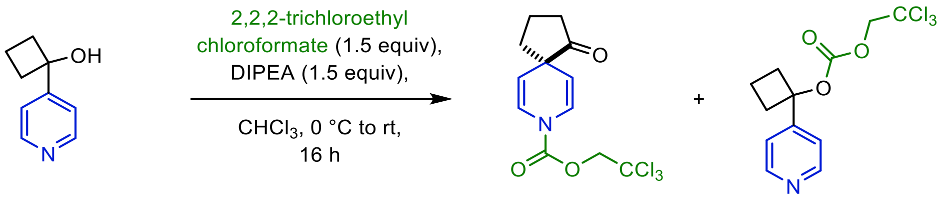

1a **2ab** **53%** **3ab** **23%**

Synthesized using GP3 with **1a** on a 0.25 mmol scale using 2,2,2-trichloroethyl chloroformate (Troc-Cl, 52 µL, 0.375 mmol, 1.5 equiv). Purified by flash column chromatography (pentane:acetone 95:5 to 60:40) to give **2ab** (43 mg, 0.13 mmol, 53%) as a white solid. In addition, **3ab** (18 mg, 0.06 mmol, 23%) was isolated as an amorphous solid.

2ab: **^{1}H NMR** (400 MHz, $CDCl_3$) δ 7.05 – 6.95 (m, 2H), 4.90 – 4.80 (m, 2H), 4.79 – 4.71 (m, 2H), 2.36 (m, 2H), 2.06 – 1.90 (m, 4H). **^{13}C NMR** (101 MHz, $CDCl_3$) δ 217.4, 149.6, 123.5, 123.1, 108.8, 108.3, 94.7, 75.7, 50.4, 40.3, 35.9, 18.5. **IR** (thin film): υ = 2960, 1725, 1690, 1629, 1417, 1384, 1333, 1308, 1215, 1121 cm^{-1}. **HRMS** (ESI) m/z: $[M+H]^+$ Calcd for $C_{12}H_{13}Cl_3NO_3$ 323.9956; Found: 323.9953. **mp** = 102 – 107 °C. (see NMR spectra)

3ab: **^{1}H NMR** (400 MHz, $CDCl_3$) δ 8.68 – 8.53 (m, 2H), 7.43 – 7.32 (m, 2H), 4.65 (s, 2H), 2.80 – 2.55 (m, 4H), 2.16 – 1.99 (m, 1H), 1.90 – 1.72 (m, 1H). **^{13}C NMR** (101 MHz, $CDCl_3$) δ 151.9, 150.4, 120.1, 94.4, 83.6, 76.6, 34.2, 13.6. **IR** (thin film): υ = 2998, 2955, 1759, 1599, 1375, 1238, 1048 cm^{-1}. **HRMS** (ESI) m/z: $[M+H]^+$ Calcd for $C_{12}H_{13}Cl_3NO_3$ 323.9956; Found: 323.9961. (see NMR spectra)

2.5.4. allyl 1-oxo-8-azaspiro[4.5]deca-6,9-diene-8-carboxylate (2ac)

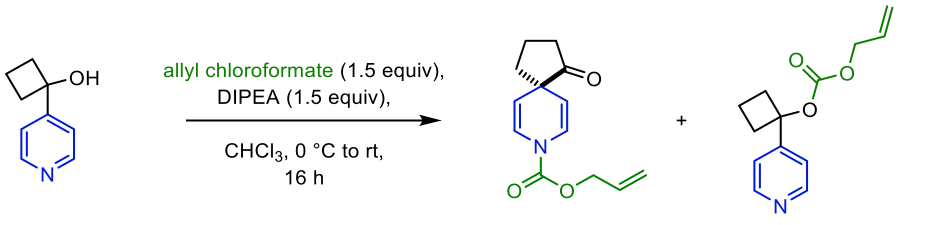

1a **2ac** **68%** **3ac** **9%** (by ^{1}H-NMR)

Synthesized using GP3 with **1a** on a 0.25 mmol scale using allyl chloroformate (40 µL, 0.375 mmol, 1.5 equiv). Purified by flash column chromatography (pentane:acetone 95:5 to 60:40) to give **2ac** (39 mg, 0.17 mmol, 68%) as a colorless oil. The amount of **3ac** (9%) was determined by ^{1}H-NMR with ISTD of the crude mixture.

2ac: ^{1}H NMR (400 MHz, $CDCl_3$) δ 7.00 (d, *J* = 7.0 Hz, 1H), 6.93 (d, *J* = 7.0 Hz, 1H), 5.92 (ddt, *J* = 17.0, 10.5, 5.5 Hz, 1H), 5.33 (dq, *J* = 17.0, 1.5 Hz, 2H), 5.25 (dq, *J* = 10.5, 1.5 Hz, 1H), 4.73 (d, *J* = 7.0 Hz, 1H), 4.68 (dt, *J* = 5.5, 1.5 Hz, 2H), 4.65 – 4.56 (d, *J* = 7.0 Hz, 1H), 2.37 – 2.27 (m, 2H), 1.95 (m, 4H). **^{13}C NMR** (101 MHz, $CDCl_3$) δ 217.7, 150.9, 131.9, 123.6, 118.9, 107.4, 107.1, 67.3, 50.4, 40.5, 35.8, 18.5. **IR** (thin film): υ = 2960, 1716, 1687, 1415, 1378, 1332, 1305, 1212, 1111, 958, 736 cm^{-1}. **HRMS** (EI) m/z: $[M]^+$ Calcd for $C_{13}H_{15}NO_3$ 233.1046; Found 233.1046. (see NMR spectra)

2.5.5. benzyl 1-oxo-8-azaspiro[4.5]deca-6,9-diene-8-carboxylate (2ad)

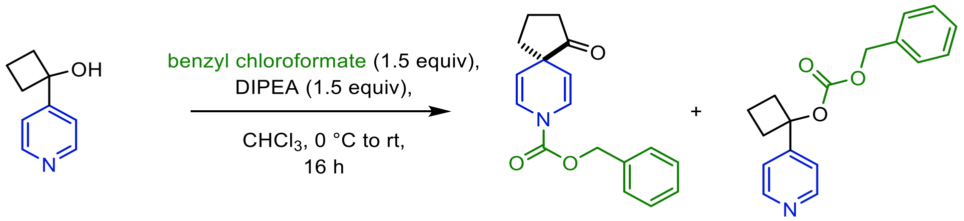

1a **2ad** 71% **3ad** 16%

Synthesized using GP3 with **1a** on a 0.25 mmol scale using benzyl chloroformate (60 µL, 0.375 mmol, 1.5 equiv). Purified by flash column chromatography (pentane:acetone 95:5 to 60:40) to give **2ad** (51 mg, 0.18 mmol, 71%) as a pale-yellow solid. In addition, **3ad** (11 mg, 0.04 mmol, 16%) was isolated as a yellow oil.

2ad: ^{1}H NMR (400 MHz, $CDCl_3$) δ 7.42 – 7.28 (m, 5H), 7.03 (d, *J* = 8.5 Hz, 1H), 6.94 (d, *J* = 8.5 Hz, 1H), 5.26 – 5.16 (s, 2H), 4.80 – 4.70 (d, *J* = 8.5 Hz, 1H), 4.61 (d, *J* = 8.5 Hz, 1H), 2.33 (td, *J* = 7.5, 2.5 Hz, 2H), 1.95 (m, 4H). **^{13}C NMR** (101 MHz, $CDCl_3$) δ 217.8, 151.1, 135.5, 128.8, 128.7, 128.5, 123.8, 123.6, 107.5, 107.1, 68.5, 50.4, 40.5, 35.9, 18.5. **IR** (thin film): υ = 2959, 1715, 1686, 1387, 1332, 1301, 1212, 1109, 957, 735 cm^{-1}. **HRMS** (ESI) m/z: $[M+Na]^+$ Calcd for $C_{17}H_{17}NO_3Na$ 306.1101; Found 306.1095. **mp** = 102 – 107 °C. (see NMR spectra)

3ad: ^{1}H NMR (400 MHz, $CDCl_3$) δ 8.64 – 8.58 (m, 2H), 7.38 – 7.28 (m, 7H), 5.05 (s, 2H), 2.74 – 2.55 (m, 4H), 2.12 – 1.97 (m, 1H), 1.79 (m, 1H). **^{13}C NMR** (101 MHz, $CDCl_3$) δ 153.1, 151.2, 150.3, 135.1, 128.8, 128.4, 120.1, 82.5, 69.7, 34.3, 13.7. **IR** (thin film): υ = 2953, 1744, 1599, 1267, 1244, 1047, 698 cm^{-1}. **HRMS** (ESI) m/z: $[M+H]^+$ Calcd for $C_{17}H_{18}NO_3$ 284.1281; Found 284.1273. (see NMR spectra)

2.5.6. 1,1,1-trichloro-2-methylpropan-2-yl 1-oxo-8-azaspiro[4.5]deca-6,9-diene-8-carboxylate (2ae)

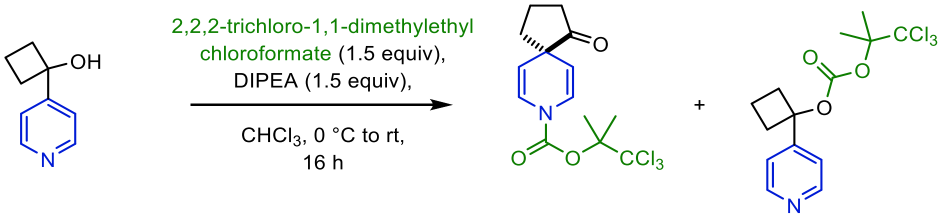

1a **2ae** 93% **3ae** not observed

Synthesized using GP3 with **1a** on a 0.25 mmol scale using 2,2,2-trichloro-1,1-dimethylethyl chloroformate (90 mg, 0.375 mmol, 1.5 equiv). Purified by flash column chromatography (pentane:acetone 95:5 to 60:40) to give **2ae** (82 mg, 0.23 mmol, 93%) as a white solid.

2ae: ^{1}**H NMR** (400 MHz, $CDCl_3$) δ 6.96 (m, 2H), 4.71 (m, 2H), 2.35 (dd, *J* = 8.0, 6.9 Hz, 2H), 2.06 – 1.85 (m, 4H), 1.95 (s, 3H), 1.93 (s, 3H). ^{13}**C NMR** (101 MHz, $CDCl_3$) δ 217.9, 148.4, 123.8, 123.3, 107.9, 107.4, 106.0, 90.3, 50.5, 40.6, 36.0, 21.6, 21.5, 18.5. **IR** (thin film): υ = 2959, 1721, 1689, 1628, 1457, 1367, 1337, 1320, 1157, 1115 cm^{-1}. **HRMS** (ESI) m/z: $[M+H]^+$ Calcd for $C_{14}H_{17}Cl_3NO_3$ 352.0269; Found 352.0266. **mp** = 133 – 138 °C. (see NMR spectra)

2.5.7. (9*H*-fluoren-9-yl)methyl 1-oxo-8-azaspiro[4.5]deca-6,9-diene-8-carboxylate (2af)

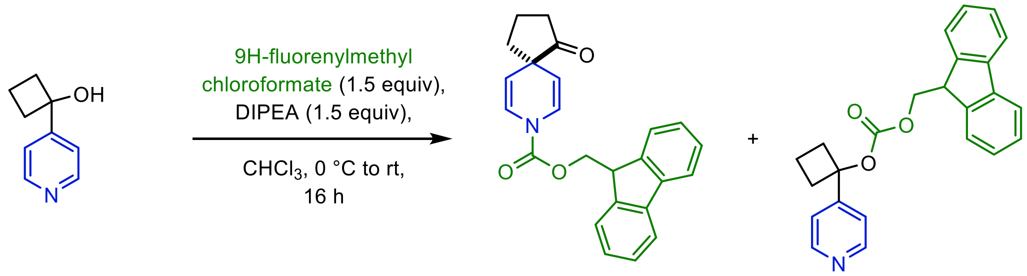

1a **2af** **64%** **3af** not observed

Synthesized using a modified GP3 with **1a** on a 0.25 mmol scale using 9*H*-fluorenylmethyl chloroformate (129 mg, 0.50 mmol, 2.0 equiv) and diisopropylethylamine (0.09 mL, 0.50 mmol, 2.0 equiv). Purified by flash column chromatography (toluene:EtOAc 15:1) to give **2af** (59 mg, 0.16 mmol, 64%) as a white solid.

2af: ^{1}**H NMR** (400 MHz, $CDCl_3$) δ 7.77 (ddd, *J* = 7.5, 1.0, 1.0 Hz, 2H), 7.57 (ddd, *J* = 7.5, 1.0, 1.0 Hz, 2H), 7.42 (ddd, *J* = 7.5, 7.5, 1.0 Hz, 2H), 7.33 (ddd, *J* = 7.5, 7.5, 1.0 Hz, 2H), 7.04 (d, *J* = 8.0 Hz, 1H), 6.88 (d, *J* = 8.0 Hz, 1H), 4.76 (d, *J* = 8.0 Hz, 1H), 4.68 (d, *J* = 8.0 Hz, 1H), 4.52 (d, *J* = 7.0 Hz, 2H), 4.27 (t, *J* = 7.0 Hz, 1H), 2.39 – 2.32 (m, 2H), 2.05 – 1.90 (m, 4H). ^{13}**C NMR** (101 MHz, $CDCl_3$) δ 217.8, 151.0, 143.5, 141.4, 128.0, 127.3, 125.0, 123.7, 123.3, 120.2, 107.6, 107.4, 68.7, 50.4, 47.0, 40.5, 35.9, 18.5. **IR** (thin film): υ = 2958, 1717, 1686, 1391, 1334, 1305, 1212, 1113 cm^{-1}. **HRMS** (ESI) m/z: $[M+Na]^+$ Calcd for $C_{24}H_{21}NO_3Na$ 394.1414; Found: 394.1429. **mp** = 102 – 107 °C. (see NMR spectra)

2.5.8. 8-pivaloyl-8-azaspiro[4.5]deca-6,9-dien-1-one (2ag)

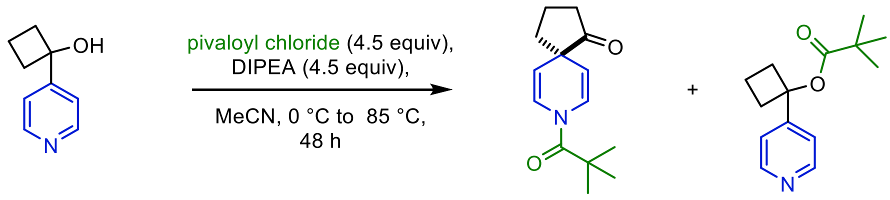

1a **2ag** **37%** **3ag** not observed

Synthesized using a modified GP3: A flame-dried microwave tube was charged with 1-(pyridin-4-yl)cyclobutan-1-ol (**1a**, 37.3 mg, 0.25 mmol, 1.0 equiv), diisopropylethylamine (DIPEA, 65 μL, 0.375 mmol, 1.5 equiv) and

anhydrous MeCN (1.4 mL, 0.18 M), and cooled to 0 °C using an ice bath. To this was added, dropwise at 0 °C, a solution of pivaloyl chloride (46 µL, 45.2 mg, 0.375 mmol, 1.5 equiv) in MeCN (0.7 mL). The reaction mixture was then heated to 85 °C (oil bath) and stirred for 48 h. After 24 h additional pivaloyl chloride (92 µL, 90.4 mg, 0.75 mmol, 3.0 equiv) in MeCN (0.7 mL) and diisopropylethylamine (130 µL, 0.75 mmol, 3.0 equiv) were added to the reaction mixture. After 48 h the reaction was then quenched with H_2O (5 mL) and extracted with DCM (3 x 5 mL). Organic layers were dried over $MgSO_4$ and concentrated *in vacuo*. Crude residues were purified by flash column chromatography (pentane:acetone 95:5) to give **2ag** (21.6 mg, 0.093 mmol, 37%) as an off-white solid.

2ag: TLC (SiO_2; pentane:acetone 9:1, UV, vanilin): R_f = 0.33. **^{1}H NMR** (400 MHz, $CDCl_3$) δ 7.20 (d, *J* = 8.1 Hz, 2H), 4.72 (d, *J* = 8.6 Hz, 2H), 2.34 (t, *J* = 7.8 Hz, 2H), 2.05 – 1.88 (m, 4H), 1.33 (s, 9H). **^{13}C NMR** (101 MHz, $CDCl_3$) δ 217.5, 173.8, 124.9, 107.5, 50.6, 40.3, 39.6, 36.0, 28.4, 18.5. **IR** (thin film): υ = 2959, 2923, 2853, 1742, 1659, 1622, 1415, 1322, 1297, 1194, 966, 740 cm^{-1}. **HRMS** (ESI) m/z: $[M+H]^+$ Calcd for $C_{14}H_{20}NO_2$ 234.1489; Found 234.1493. **mp** = 116 – 118 °C. (see NMR spectra)

2.5.9. 8-((trifluoromethyl)sulfonyl)-8-azaspiro[4.5]deca-6,9-dien-1-one (2ah)

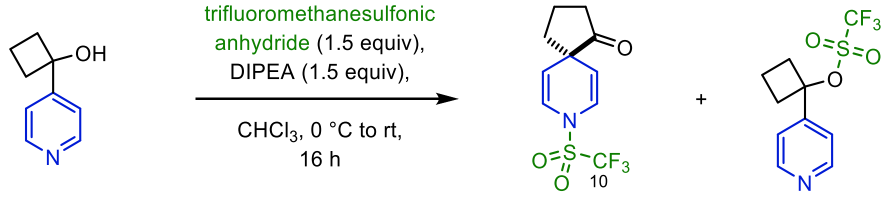

1a **2ah** 78% **3ah** not observed

Synthesized using GP3 with **1a** on a 0.25 mmol scale using trifluoromethanesulfonic anhydride (1 M in DCM, 0.38 mL, 0.375 mmol, 1.5 equiv). Purified by flash column chromatography (pentane:acetone 95:5 to 60:40) to give **2ah** (55 mg, 0.20 mmol, 78%) as a colorless oil.

2ah: ^{1}H NMR (400 MHz, $CDCl_3$) δ 6.60 (d, *J* = 8.0 Hz, 2H), 4.92 (d, *J* = 8.0 Hz, 2H), 2.36 (td, *J* = 7.5, 7.0, 1.5 Hz, 2H), 2.12 – 1.91 (m, 4H). **^{13}C NMR** (101 MHz, $CDCl_3$) δ 215.5, 122.3, 111.1, 49.7, 39.9, 35.8, 18.6 (C_{10} not observed due to 19F splitting). **IR** (thin film): υ = 2967, 1744, 1407, 1229, 1160, 1060, 936, 703, 658, 591 cm^{-1}. **HRMS** (APCI) m/z: $[M+H]^+$ Calcd for $C_{10}H_{11}F_3NO_3S$ 282.0406; Found 282.0403. (see NMR spectra)

2.5.10. 8-tosyl-8-azaspiro[4.5]deca-6,9-dien-1-one (2ai)

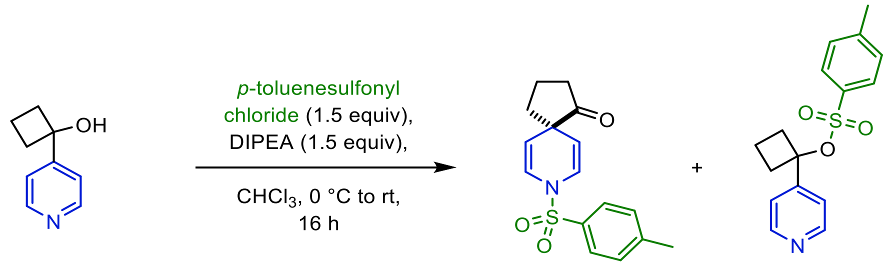

1a **2ai** 66% **3ai** not observed

Synthesized using GP3 with **1a** on a 0.25 mmol scale using *p*-toluenesulfonyl chloride (72 mg, 0.375 mmol, 1.5 equiv). Purified by flash column chromatography (pentane:acetone 95:5 to 60:40) to give **2ai** (50 mg, 0.17 mmol, 66%) as a yellow solid.

2ai: **^{1}H NMR** (400 MHz, $CDCl_3$) δ 7.76 – 7.56 (m, 2H), 7.41 – 7.28 (m, 2H), 6.76 – 6.61 (m, 2H), 4.74 – 4.58 (m, 2H), 2.43 (s, 3H), 2.26 (t, *J* = 7.5 Hz, 2H), 1.92 (dt, *J* = 14.0, 7.0 Hz, 2H), 1.85 – 1.78 (m, 2H). **^{13}C NMR** (101 MHz, $CDCl_3$) δ 216.7, 144.5, 135.2, 130.1, 127.0, 123.5, 108.6, 50.0, 40.6, 35.7, 21.8, 18.4. **IR** (thin film): υ = 2962, 1741, 1372, 1347, 1170, 711, 668 cm^{-1}. **HRMS** (ESI) m/z: $[M+H]^+$ Calcd for $C_{16}H_{18}NO_3S$ 304.1002; Found 304.0998. **mp** = 127 – 132 °C. (see NMR spectra)

2.5.11. 8-((4-nitrophenyl)sulfonyl)-8-azaspiro[4.5]deca-6,9-dien-1-one (2aj)

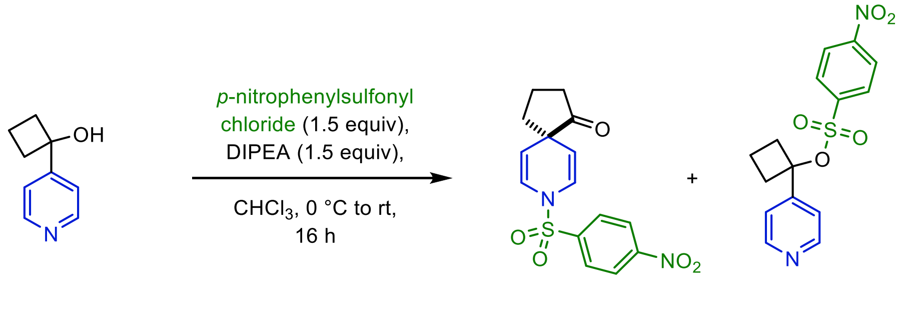

1a **2aj** 35% **3aj** not observed

Synthesized using GP3 with **1a** on a 0.25 mmol scale using *p*-nitrophenylsulfonyl chloride (83 mg, 0.375 mmol, 1.5 equiv). Purified by flash column chromatography (pentane:acetone 94:6 to 50:50) to give **2aj** (29 mg, 0.09 mmol, 35%) as an amorphous orange solid.

2aj: **^{1}H NMR** (400 MHz, $CDCl_3$) δ 8.50 – 8.28 (m, 2H), 8.09 – 7.90 (m, 2H), 6.67 (d, *J* = 8.0 Hz, 2H), 4.77 (d, *J* = 8.0 Hz, 2H), 2.24 (t, *J* = 7.5 Hz, 2H), 1.98 – 1.89 (m, 2H), 1.86 (m, 2H). **^{13}C NMR** (101 MHz, $CDCl_3$) δ 215.8, 150.6, 143.1, 128.4, 124.7, 123.0, 111.1, 50.2, 40.0, 35.7, 18.5. **IR** (thin film): υ = 3106, 1740, 1530, 1377, 1346, 1176, 955, 739, 606 cm^{-1}. **HRMS** (ESI) m/z: $[M+H]^+$ Calcd for $C_{15}H_{15}N_2O_5S$ 335.0696; Found 335.0693. (see NMR spectra)

2.6. Hydrogenation of Spirocyclic Dihydropyridines

2.6.1. *tert*-butyl 1-oxo-8-azaspiro[4.5]decane-8-carboxylate (4a)

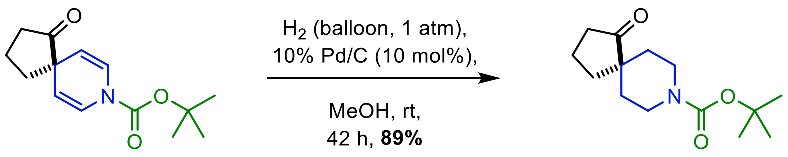

2a **4a**

In a 50 mL round-bottom flask, diene **2a** (100 mg, 0.401 mmol, 1.0 equiv) was dissolved in dry methanol (10 mL). To the pale-yellow solution Pd/C (10 wt%, 42.7 mg, 40.1 µmol, 10 mol%) was added and the reaction vessel was three times evacuated and vented with N_2. Then the reaction vessel was three times evacuated and vented with H_2 (balloon). The resulting black suspension was stirred under an H_2 atmosphere for 42 h at rt, before the reaction mixture was filtered through a celite pad (2 cm). The residue was washed with EtOAc (150 mL) and the filtrate was concentrated under reduced pressure to give **4a** (90.0 mg, 0.357 mmol, 89%) as a white solid.

TLC (SiO_2; pentane:EtOAc 3:1, UV, $KMnO_4$): R_f = 0.33. **^{1}H NMR** (400 MHz, $CDCl_3$) δ 3.84 (dt, *J* = 13.6, 4.7 Hz, 2H), 3.05 (ddd, *J* = 13.7, 10.3, 3.4 Hz, 2H), 2.35 – 2.22 (m, 2H), 1.96 – 1.84 (m, 4H), 1.64 (ddd, *J* = 13.5, 10.3, 4.3 Hz, 2H), 1.44 (s, 9H), 1.37 – 1.26 (m, 2H). **^{13}C NMR** (101 MHz, $CDCl_3$) δ 222.0, 154.9, 79.6, 47.4, 40.2, 37.9, 34.2, 31.8, 28.6, 18.8. **IR** (thin film): υ = 2969, 2935, 2856, 1732, 1686, 1421, 1365, 1249, 1148 cm^{-1}. **HRMS** (ESI) m/z: $[M+Na]^+$ Calcd for $C_{14}H_{23}NO_3Na$ 276.1576; Found 276.1577. **mp** = 72 – 73 °C. (see NMR spectra)

2.6.2. *tert*-butyl 1-oxo-6-phenyl-8-azaspiro[4.5]decane-8-carboxylate (4b)

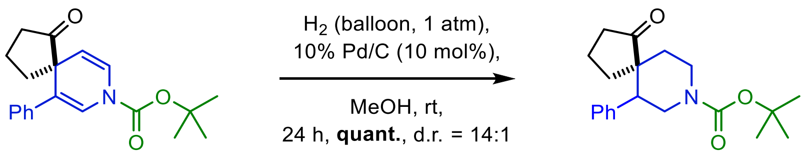

2k **4b**

In a 25 mL round-bottom flask, diene **2k** (17.1 mg, 52.5 µmol, 1.0 equiv) was dissolved in dry methanol (3.0 mL). To the colorless solution Pd/C (10 wt%, 5.6 mg, 5.3 µmol, 10 mol%) was added and the reaction vessel was three times evacuated and vented with N_2. Then the reaction vessel was three times evacuated and vented with H_2 (balloon). The resulting black suspension was stirred under an H_2 atmosphere for 24 h at rt, before the reaction mixture was filtered through a celite pad (2 cm). The residue was washed with EtOAc (150 mL), and the filtrate was concentrated under reduced pressure to give **4b** (17.3 mg, 52.5 µmol, quant., d.r. = 14:1) as a colorless oil.

TLC (SiO_2; pentane:Et_2O 3:2, UV, $KMnO_4$): major diastereoisomer: R_f = 0.42; minor diastereoisomer: R_f = 0.33. Mixture of rotamers and diastereoisomers (d.r. = 14:1), NMR data is reported for the major rotamer of the major diastereoisomer. **^{1}H NMR** (500 MHz, $CDCl_3$) δ 7.33 – 7.19 (m, 3H), 7.15 (d, *J* = 7.2 Hz, 2H), 4.26 – 3.68 (br. m, 3H), 3.54 – 3.23 (br. m, 1H), 2.71 – 2.60 (m, 1H), 2.22 (ddd, *J* = 18.6, 8.4, 4.7 Hz, 1H), 2.09 – 1.95 (m, 1H), 1.91 – 1.74 (m, 2H), 1.73 – 1.50 (m, 3H), 1.44 (s, 9H), 1.35 – 1.21 (m, 1H). **^{13}C NMR** (126 MHz, $CDCl_3$) δ 222.6, 155.2, 140.0, 129.2, 128.7, 127.4, 79.6, 50.8, 50.0, 45.4, 40.9, 39.2, 37.0, 34.4, 28.6, 18.9. **IR** (thin film): υ = 2955, 2931, 2889, 1724, 1689, 1418, 1275, 1250, 1153, 1137, 704 cm^{-1}. **HRMS** (ESI) m/z: $[M+Na]^+$ Calcd for $C_{20}H_{27}NO_3Na$ 352.1889; Found: 352.1881. (see NMR spectra)

3. X-RAY CRYSTALLOGRAPHIC ANALYSIS

3.1. (9*H*-fluoren-9-yl)methyl 1-oxo-8-azaspiro[4.5]deca-6,9-diene-8-carboxylate (2af)

Crystal preparation: **2af** was dissolved in a minimum amount of $CHCl_3$. Hexane was overlayed carefully to form a biphasic solution. After 3 d the crystals could be harvested.

X-ray diffraction experiments on **2af** were carried out at 100(2) K on a Bruker D8 Venture diffractometer using Cu-K_α radiation (λ = 1.54178 Å). Data collections were performed using a Bruker CPAD detector. Intensities were integrated in SAINT[7] and absorption corrections based on equivalent reflections were applied using SADABS.[8] The structure was solved using ShelXT[9] and refined by full matrix least squares against F^2 in ShelXL[10,11] using Olex2.[12] All of the non-hydrogen atoms were refined anisotropically. While all of the hydrogen atoms were located geometrically and refined using a riding model. The crystal structure and refinement data are given in Table 4. Crystallographic data for compound **2af** has been deposited with the Cambridge Crystallographic Data Centre as supplementary publication CCDC 2216520. Copies of the data can be obtained free of charge on application to CCDC, 12 Union Road, Cambridge CB2 1EZ, UK [fax(+44) 1223 336033, e-mail: deposit@ccdc.cam.ac.uk].

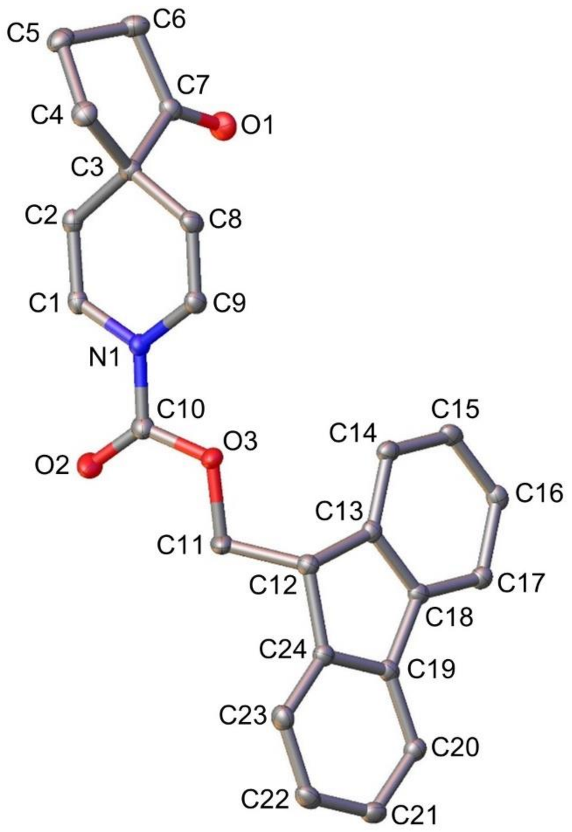

Figure 1. Crystal structure of **2af** with the anisotropic displacement parameters depicted at the 50% probability level and hydrogens omitted for clarity.

Table 4 Crystal data and structure refinement for 2af

CCDC number	2216520
Empirical formula	$C_{24}H_{21}NO_3$
Formula weight	371.42
Temperature/K	100.0
Crystal system	monoclinic
Space group	$P2_1/n$
a/Å	13.2168(4)
b/Å	5.9722(2)
c/Å	23.2479(7)
α/°	90
β/°	92.9010(10)
γ/°	90
Volume/Å^3	1832.68(10)
Z	4
ρ_{calc}g/cm^3	1.346
μ/mm^{-1}	0.711
F(000)	784.0
Crystal size/mm^3	0.392 × 0.133 × 0.125
Radiation	CuKα (λ = 1.54178)
2Θ range for data collection/°	7.534 to 144.658
Index ranges	-16 ≤ h ≤ 16, -7 ≤ k ≤ 7, -28 ≤ l ≤ 28

Reflections collected	27720
Independent reflections	3621 [R_{int} = 0.0346, R_{sigma} = 0.0200]
Data/restraints/parameters	3621/0/253
Goodness-of-fit on F^2	1.052
Final R indexes [I>=2σ (I)]	R_1 = 0.0380, wR_2 = 0.0999
Final R indexes [all data]	R_1 = 0.0397, wR_2 = 0.1013
Largest diff. peak/hole / e Å^{-3}	0.27/-0.25

4. SPECTROSCOPIC DATA

^{1}H NMR (400 MHz, $CDCl_3$) of **SI-1** (see procedure)

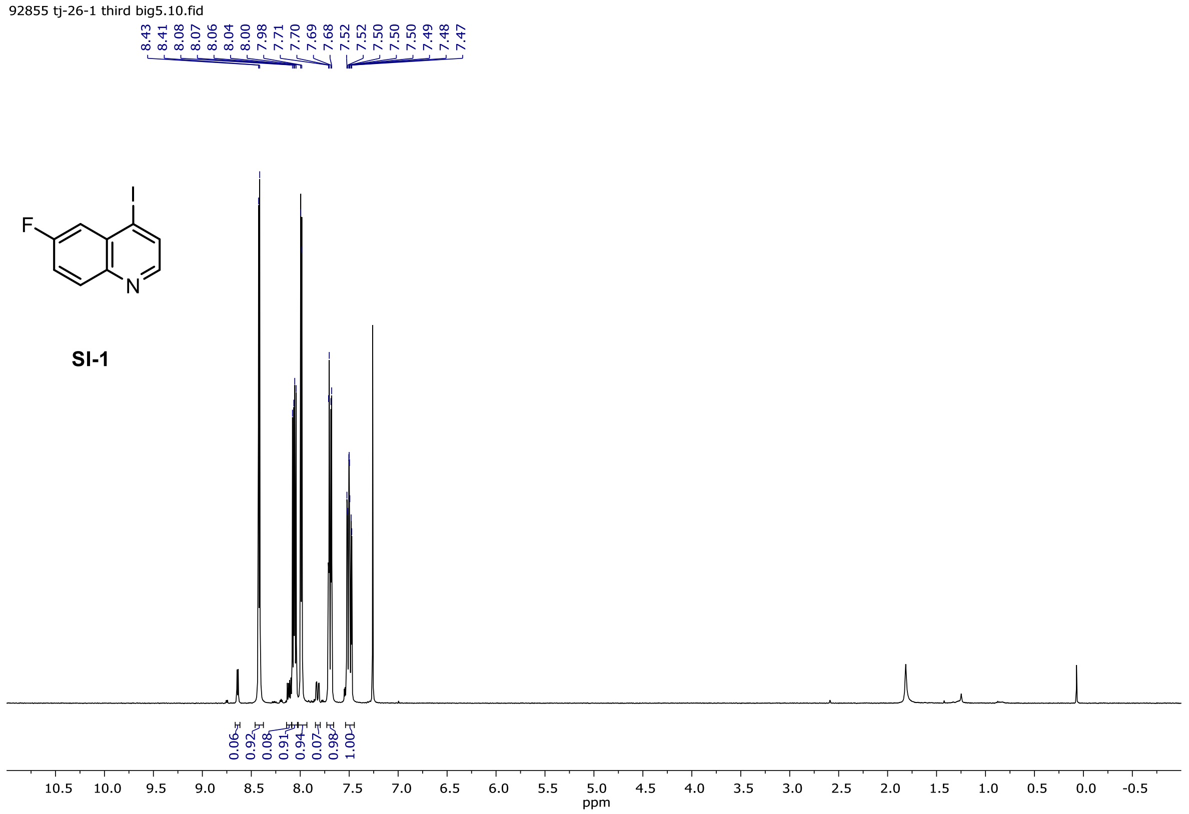

SI-1

^{13}C NMR (101 MHz, $CDCl_3$) of **SI-1**

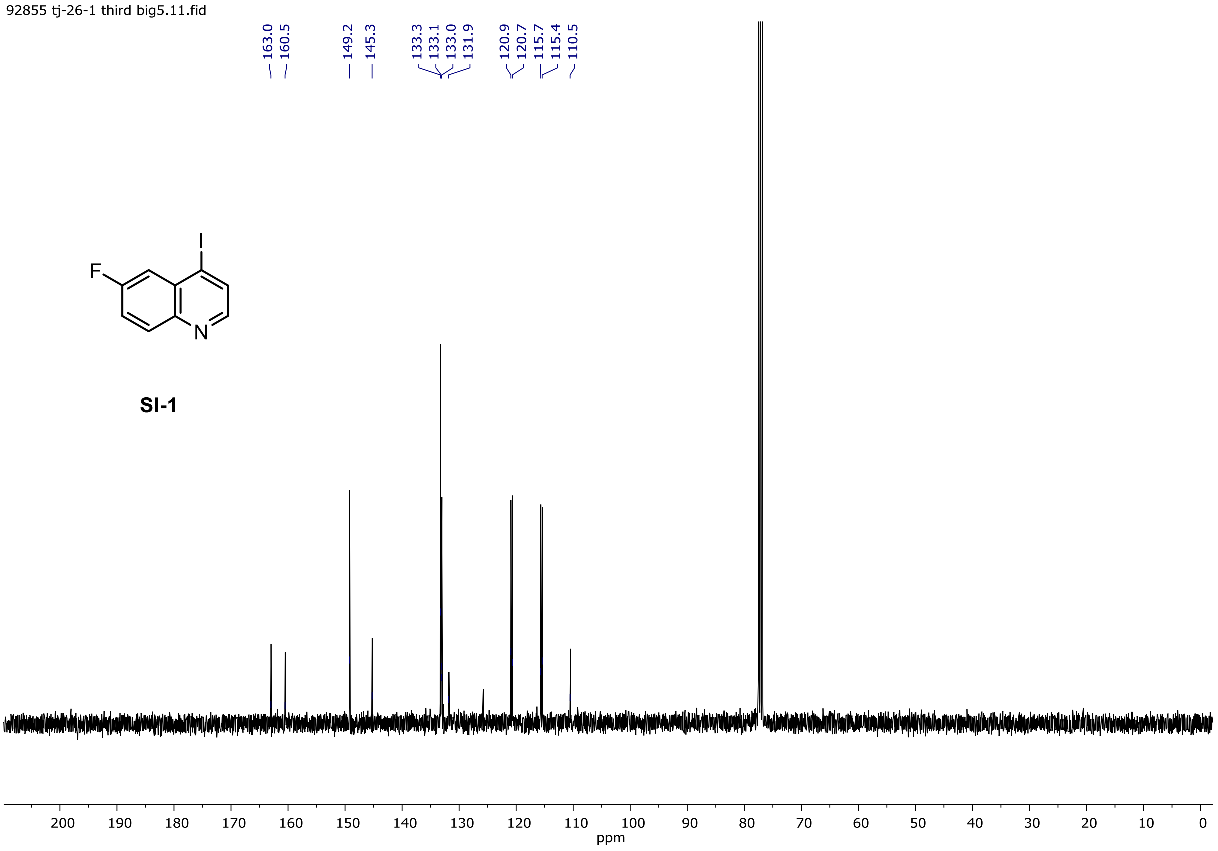

SI-1

^{1}H NMR (400 MHz, $CDCl_3$) of **SI-2** (see procedure)

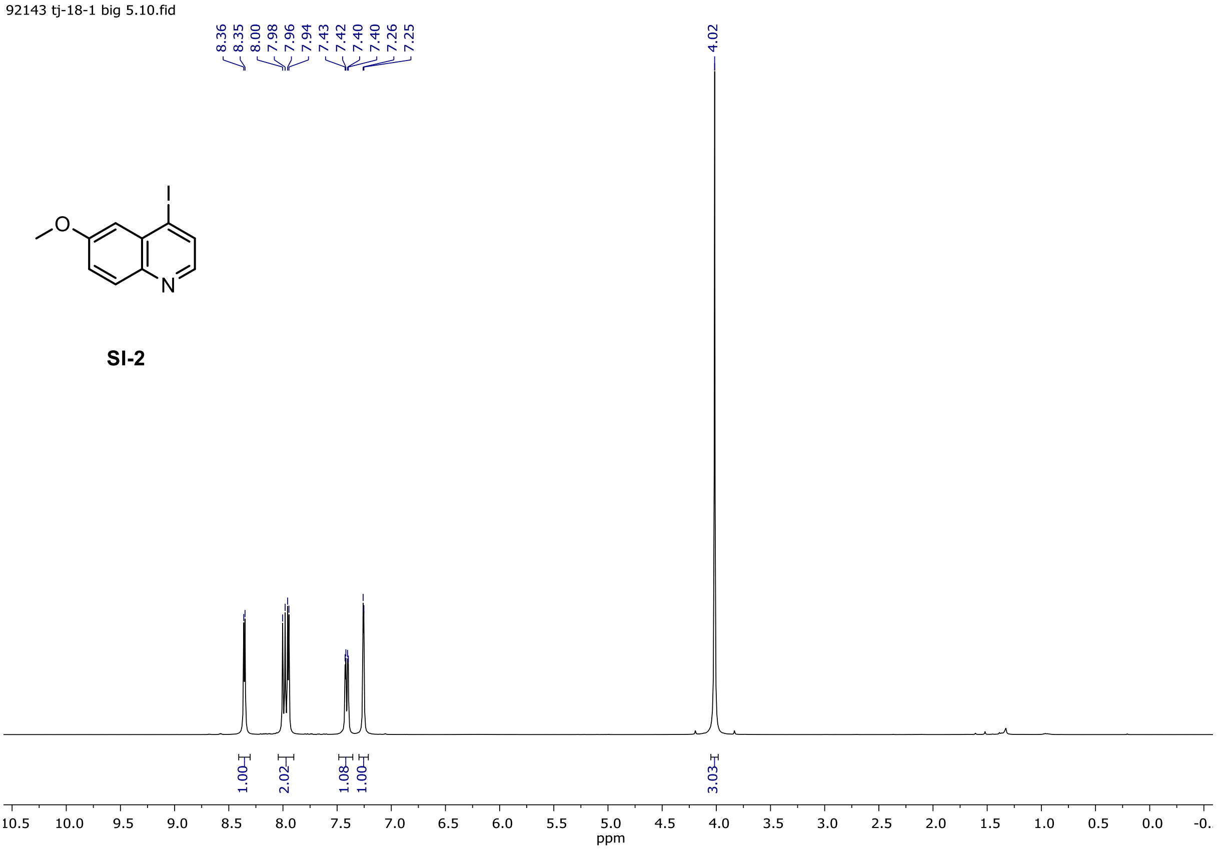

^{13}C NMR (101 MHz, $CDCl_3$) of **SI-2**

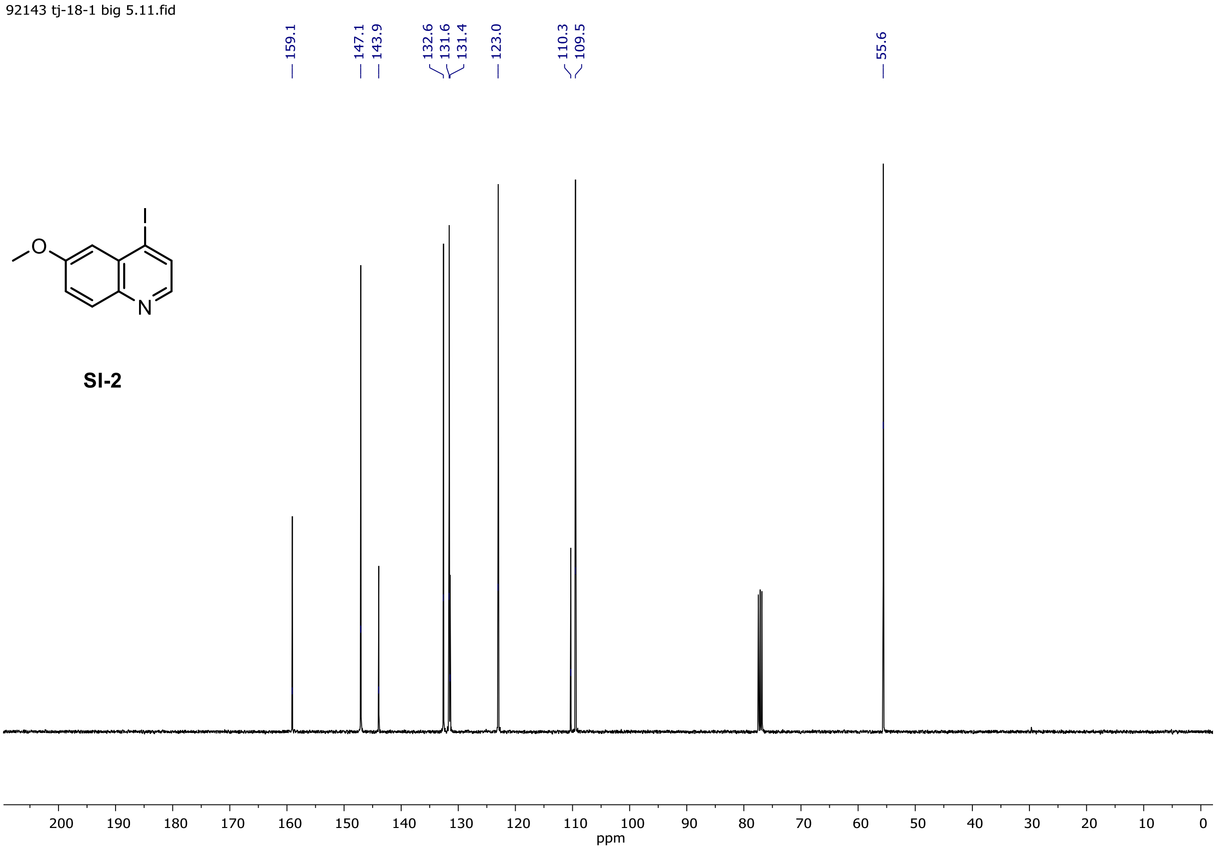

^{1}H NMR (400 MHz, $CDCl_3$) of **SI-3** (see procedure)

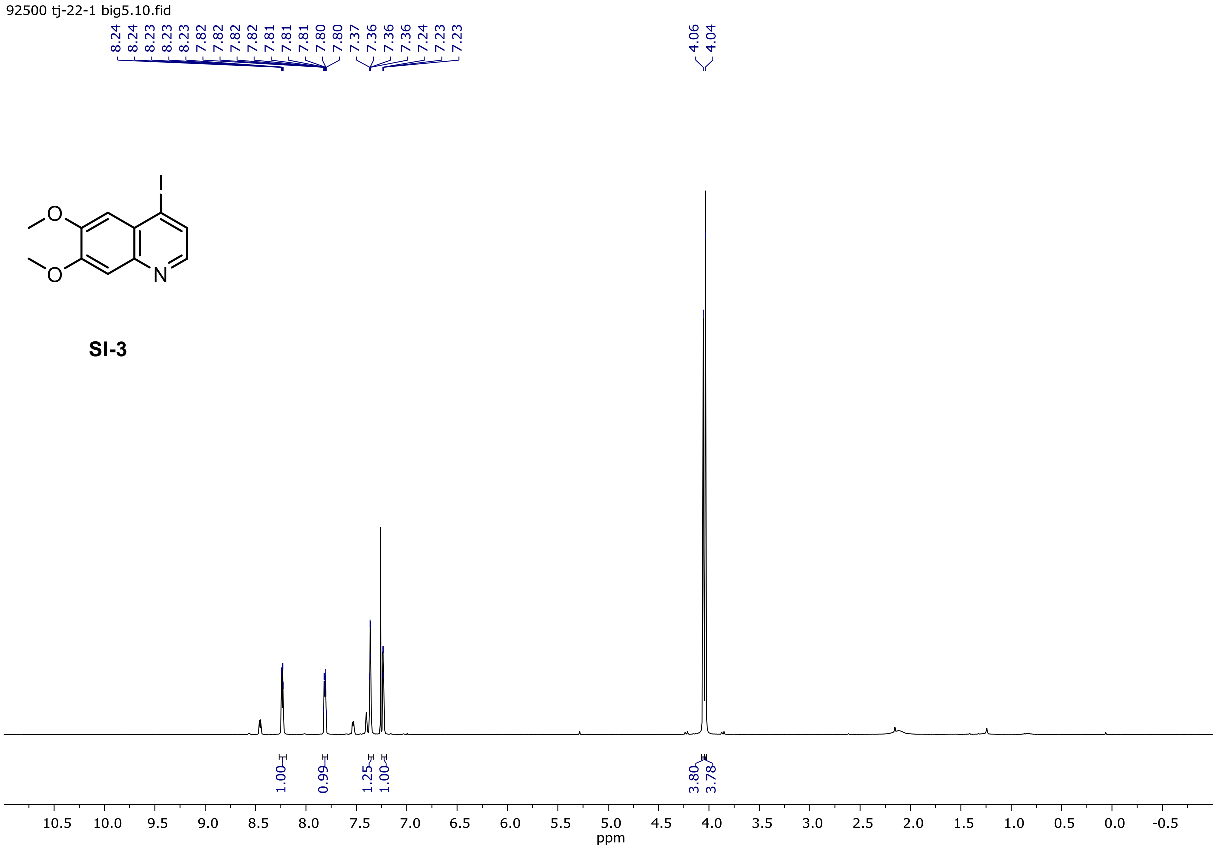

^{13}C NMR (101 MHz, $CDCl_3$) of **SI-3**

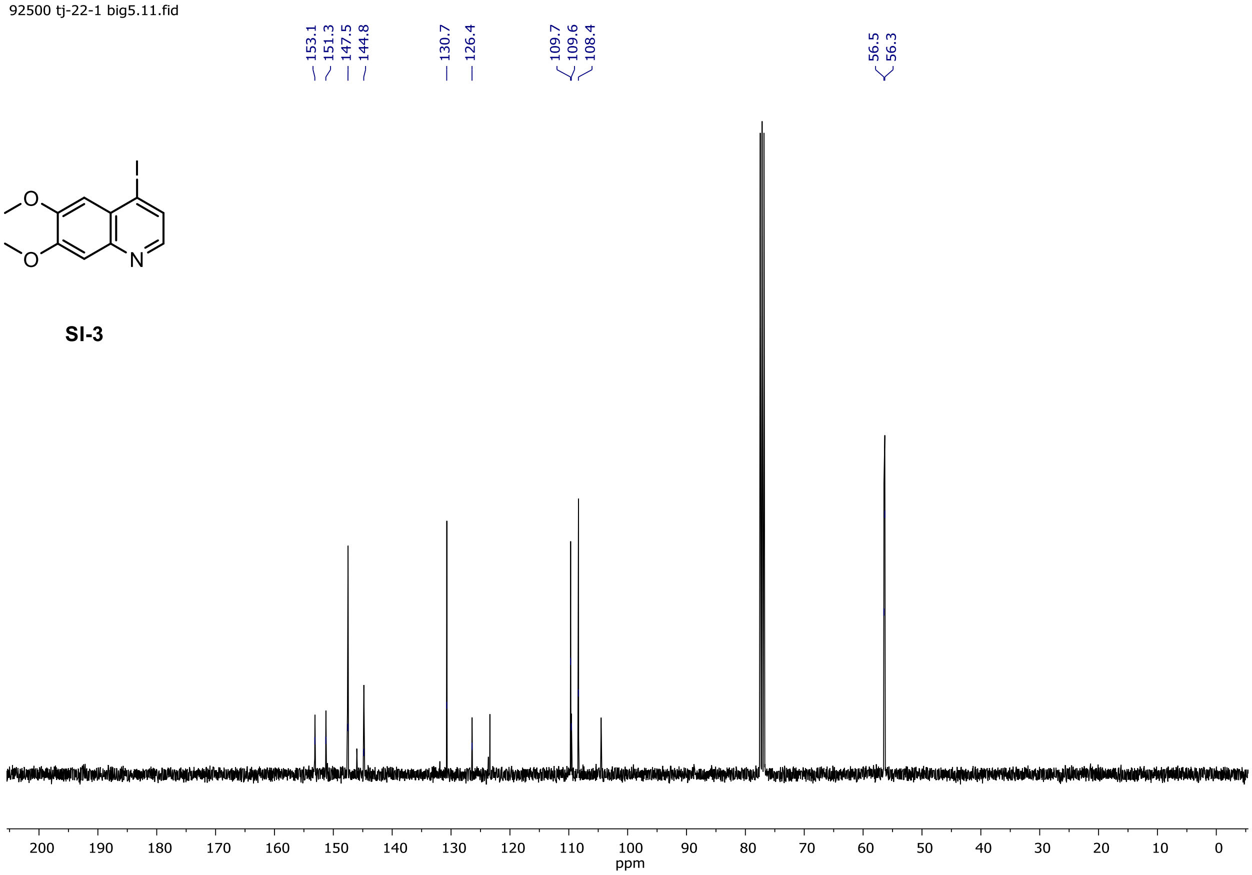

^{1}H NMR (400 MHz, $CDCl_3$) of **1a** (see procedure)

va/lv30264 LV003_Substrate

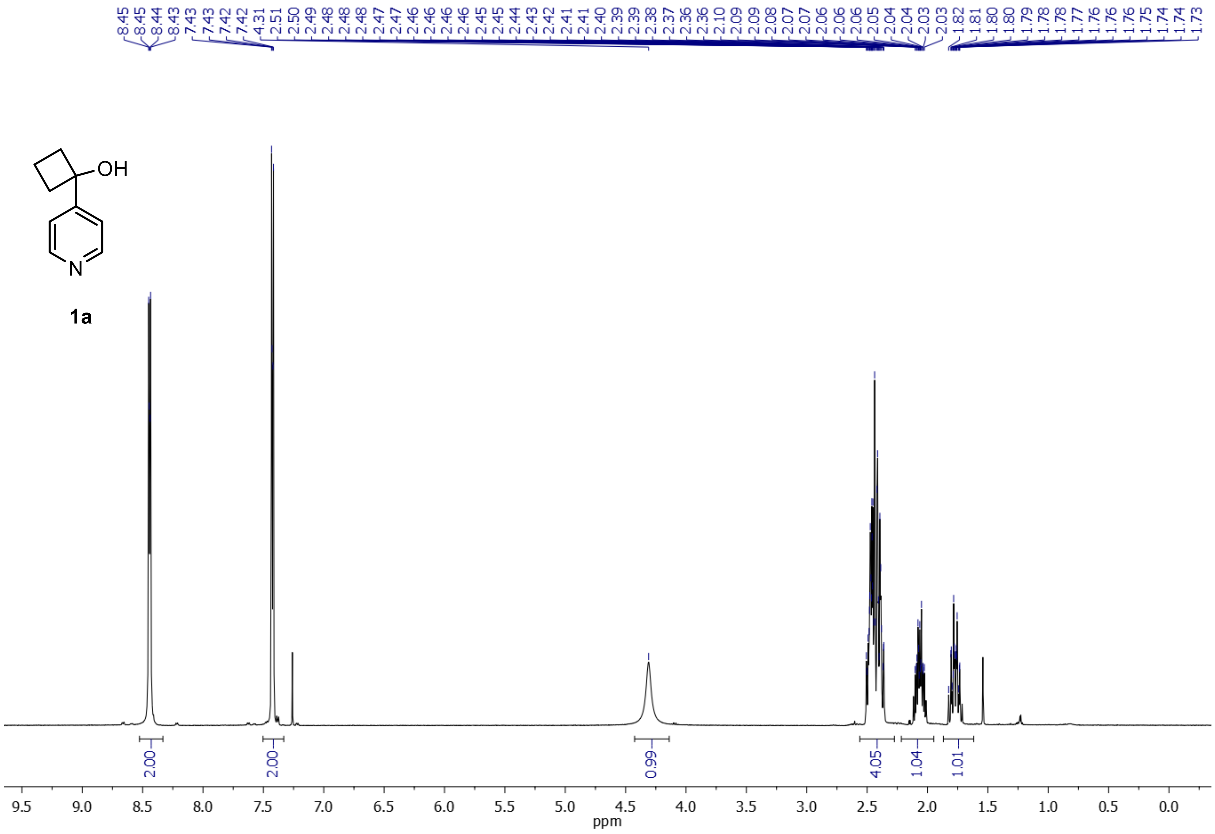

^{13}C NMR (101 MHz, $CDCl_3$) of **1a**

va/lv30264 LV003_Substrate

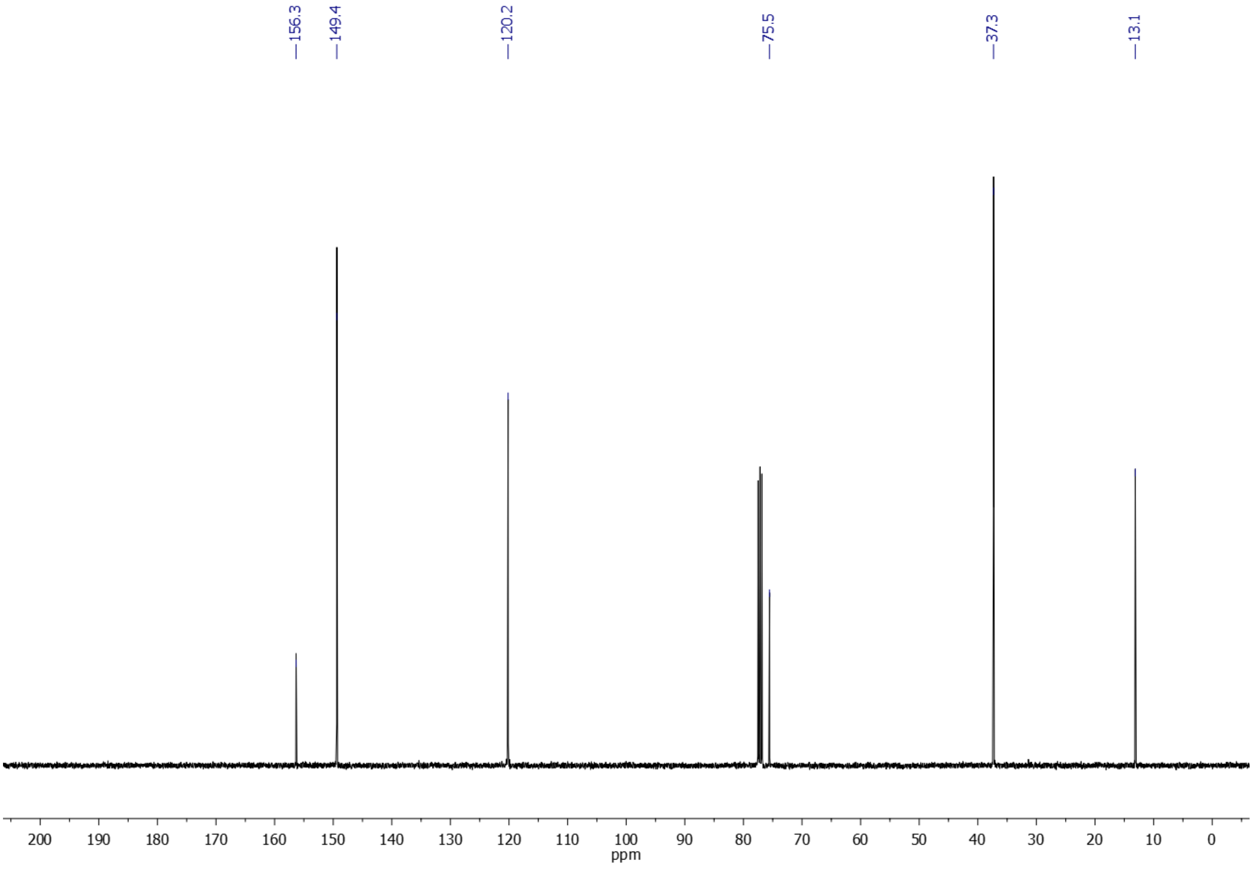

^{1}H NMR (400 MHz, $CDCl_3$) of **1b** (see procedure)

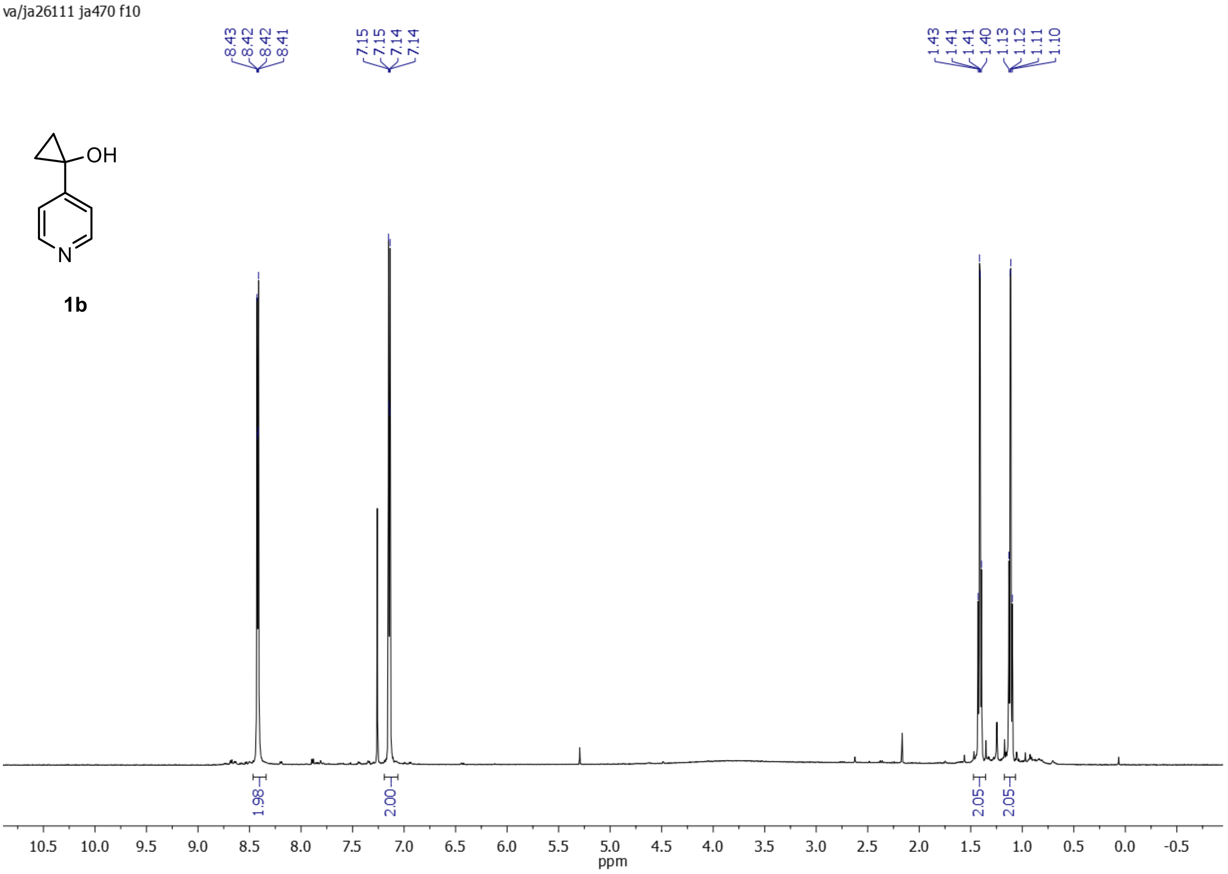

^{13}C NMR (101 MHz, $CDCl_3$) of **1b**

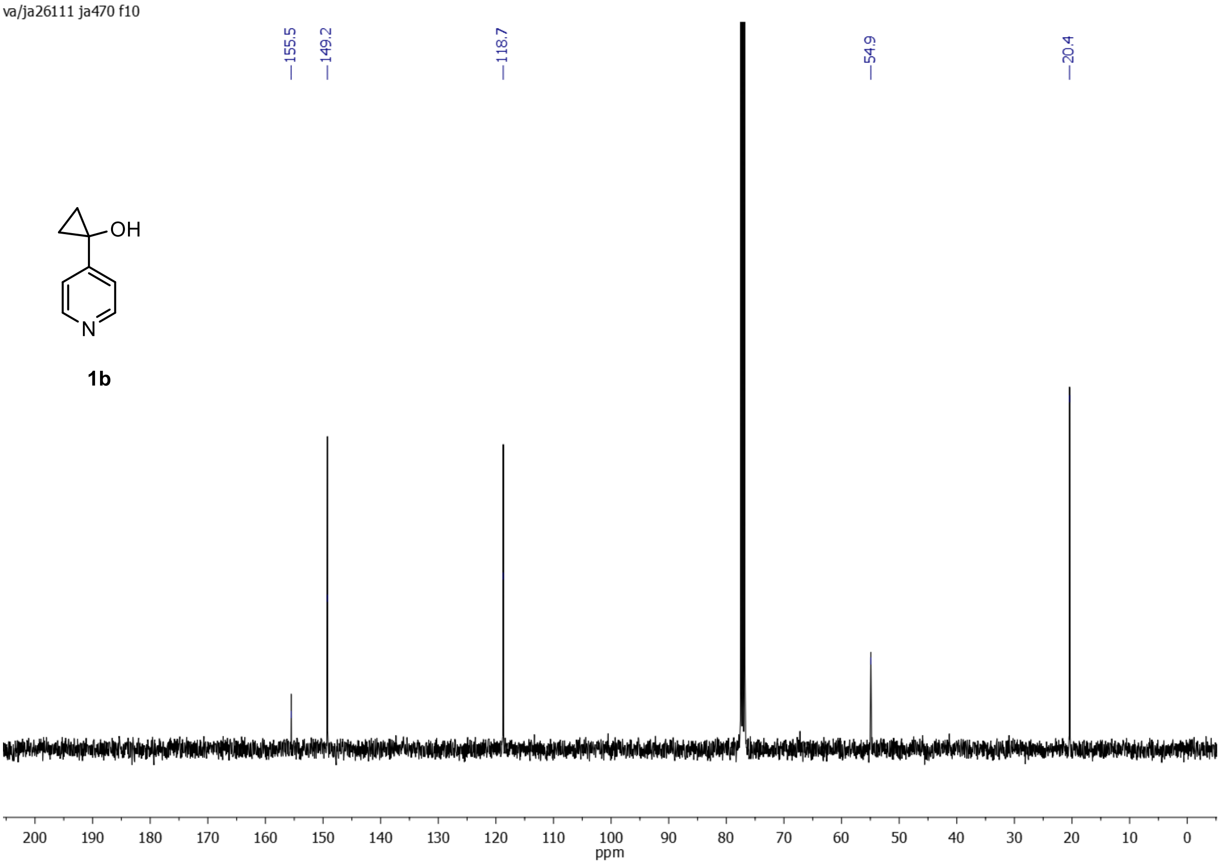

^{1}H NMR (400 MHz, $CDCl_3$) of **1c** (see procedure)

va/ja27313 ja477 prod dilute

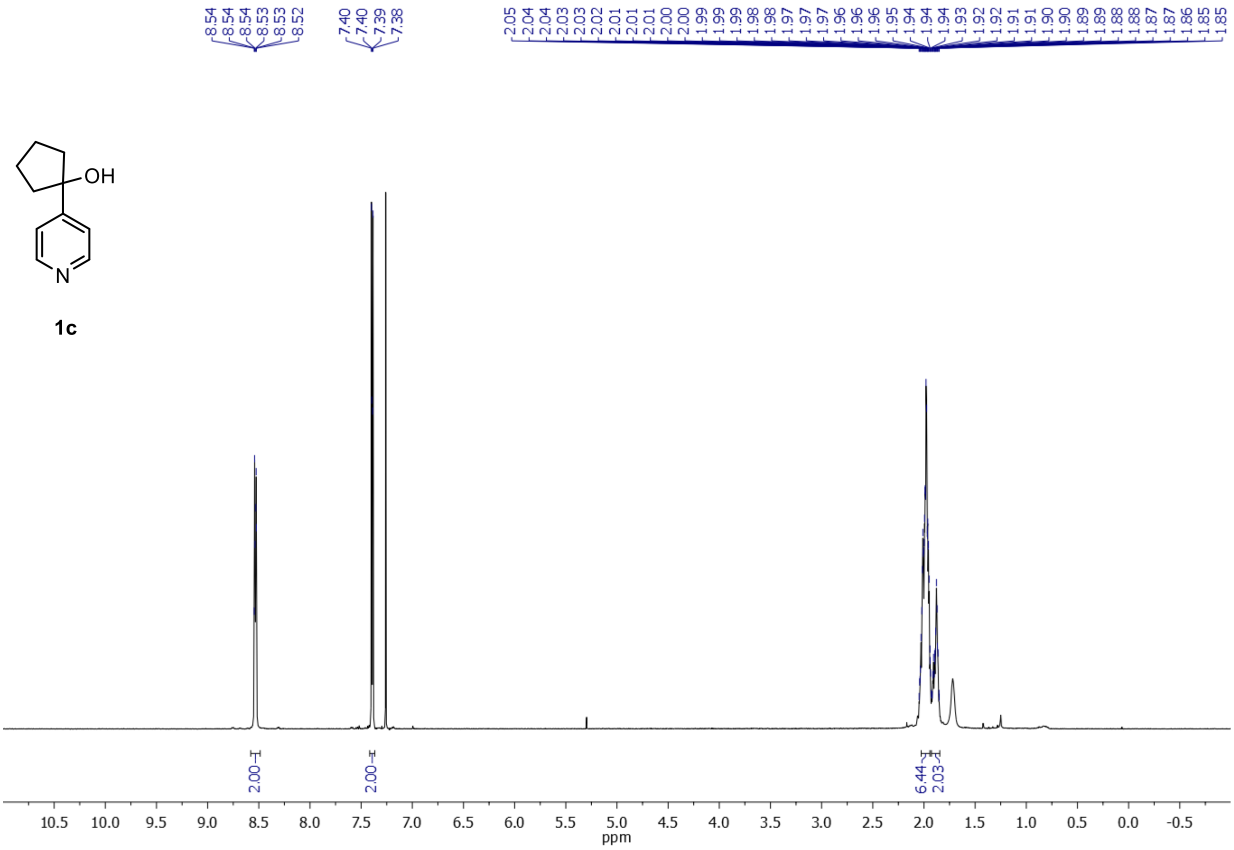

^{13}C NMR (101 MHz, $CDCl_3$) of **1c**

va/ja27111 ja477 prod conc

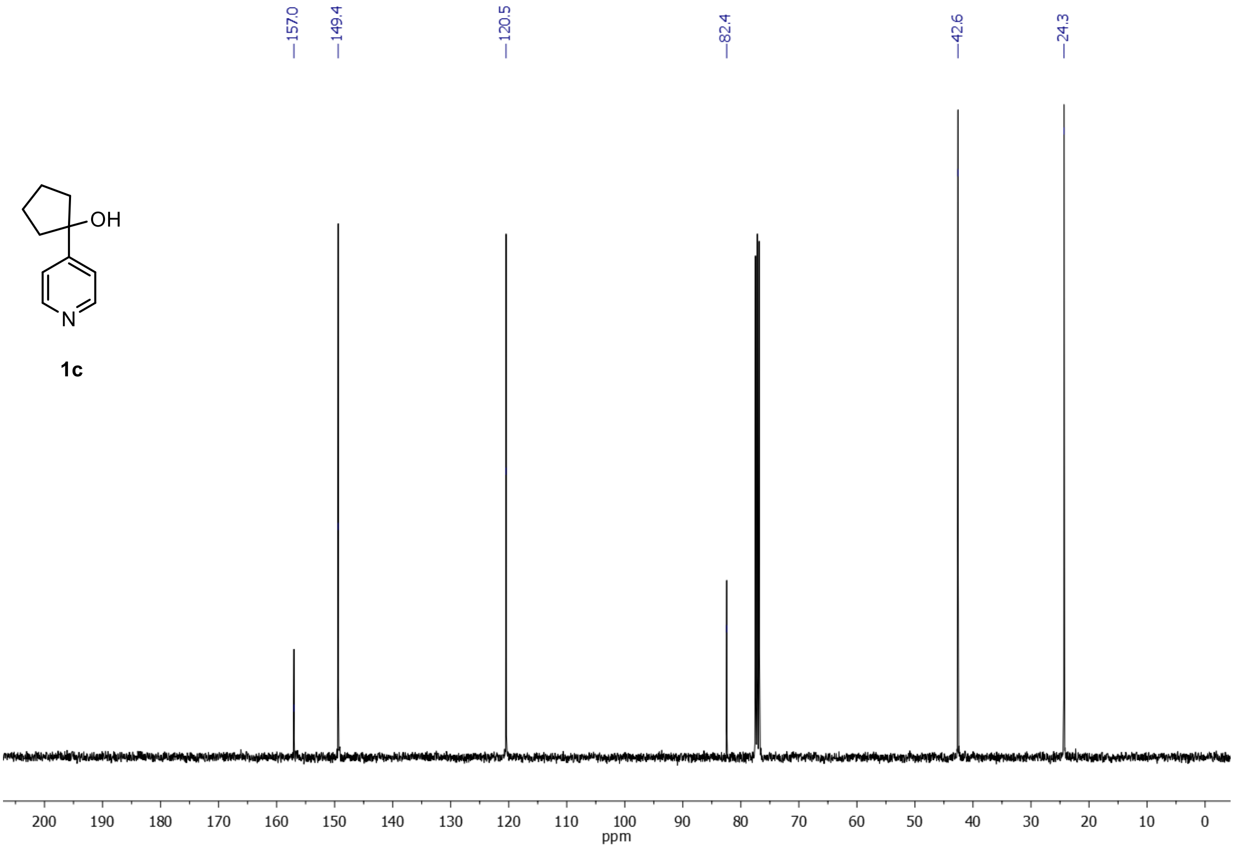

^{1}H NMR (400 MHz, $CDCl_3$) of **1d** (see procedure)

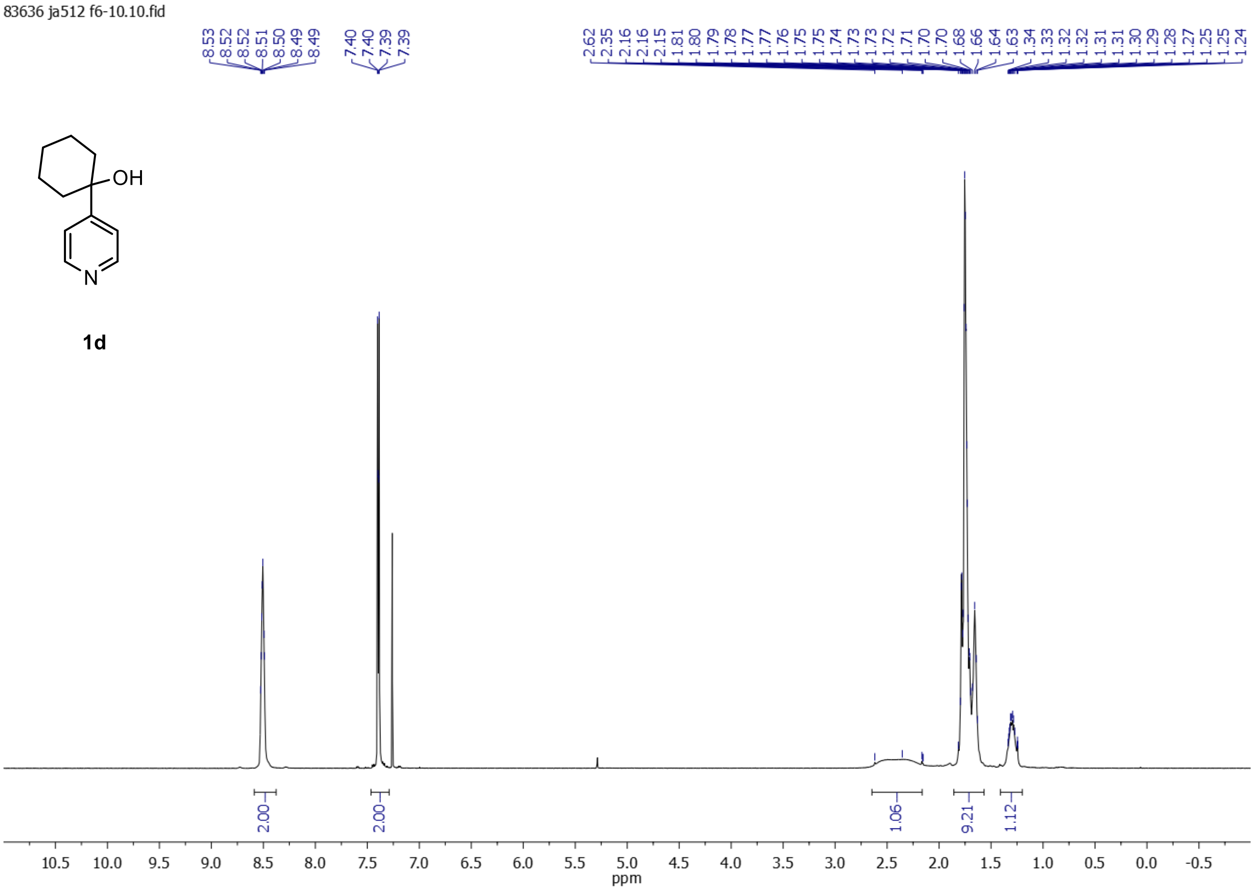

^{13}C NMR (101 MHz, $CDCl_3$) of **1d**

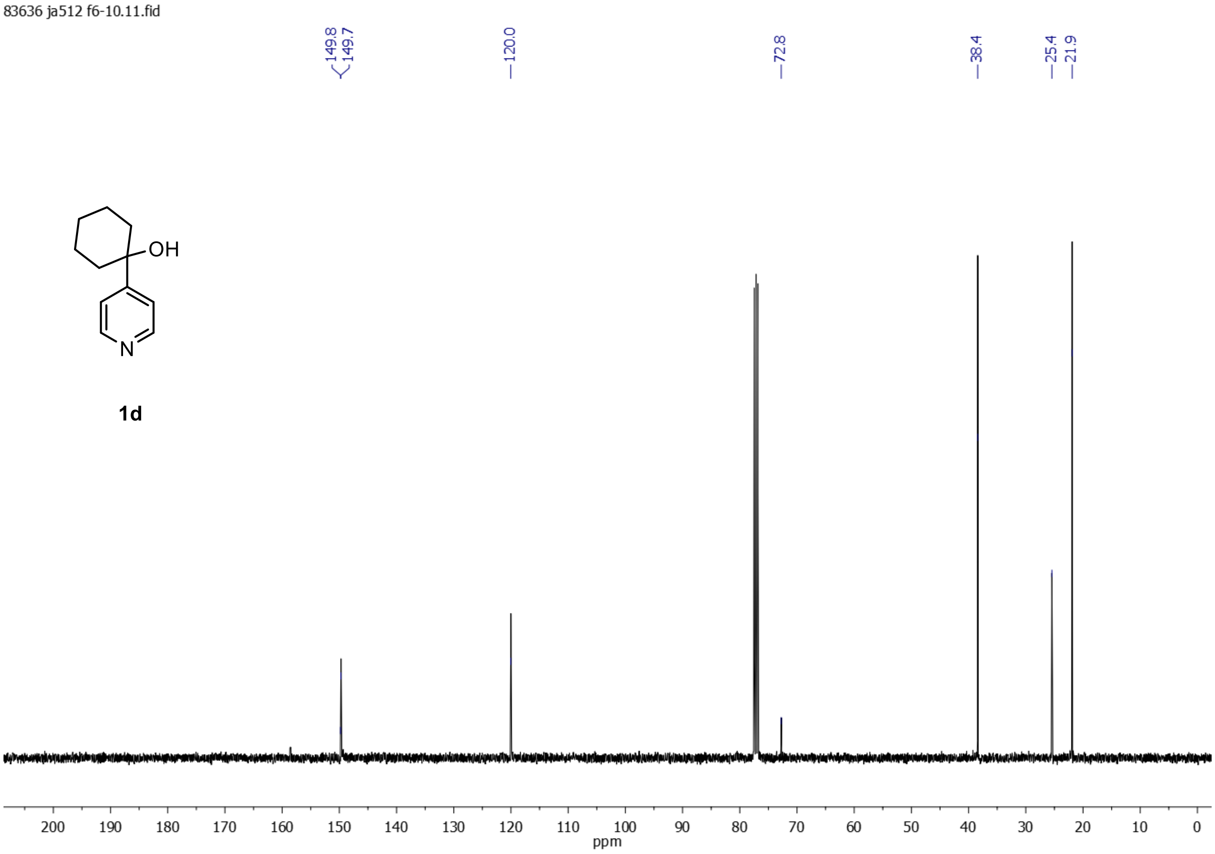

^{1}H NMR (400 MHz, $CDCl_3$) of **1e** (see procedure)

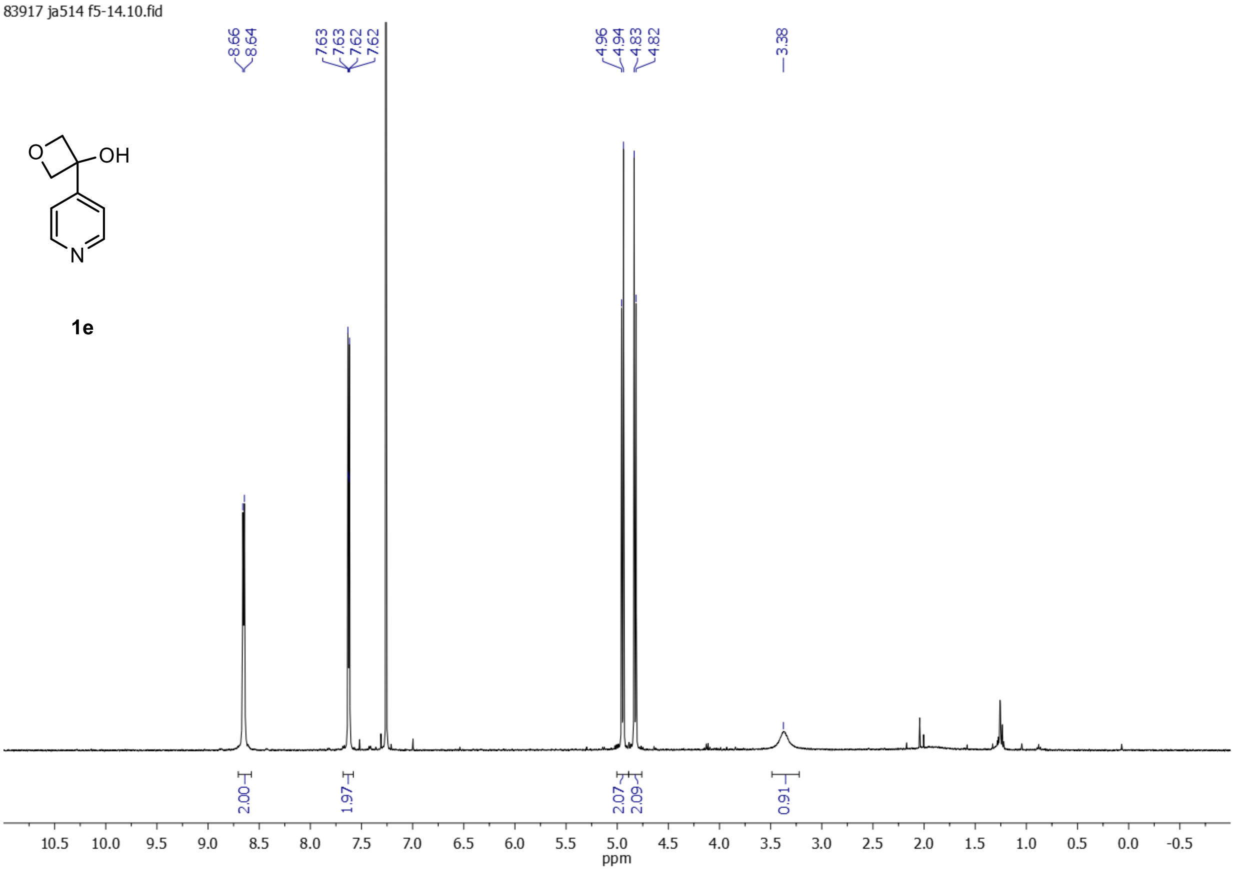

^{13}C NMR (101 MHz, $CDCl_3$) of **1e**

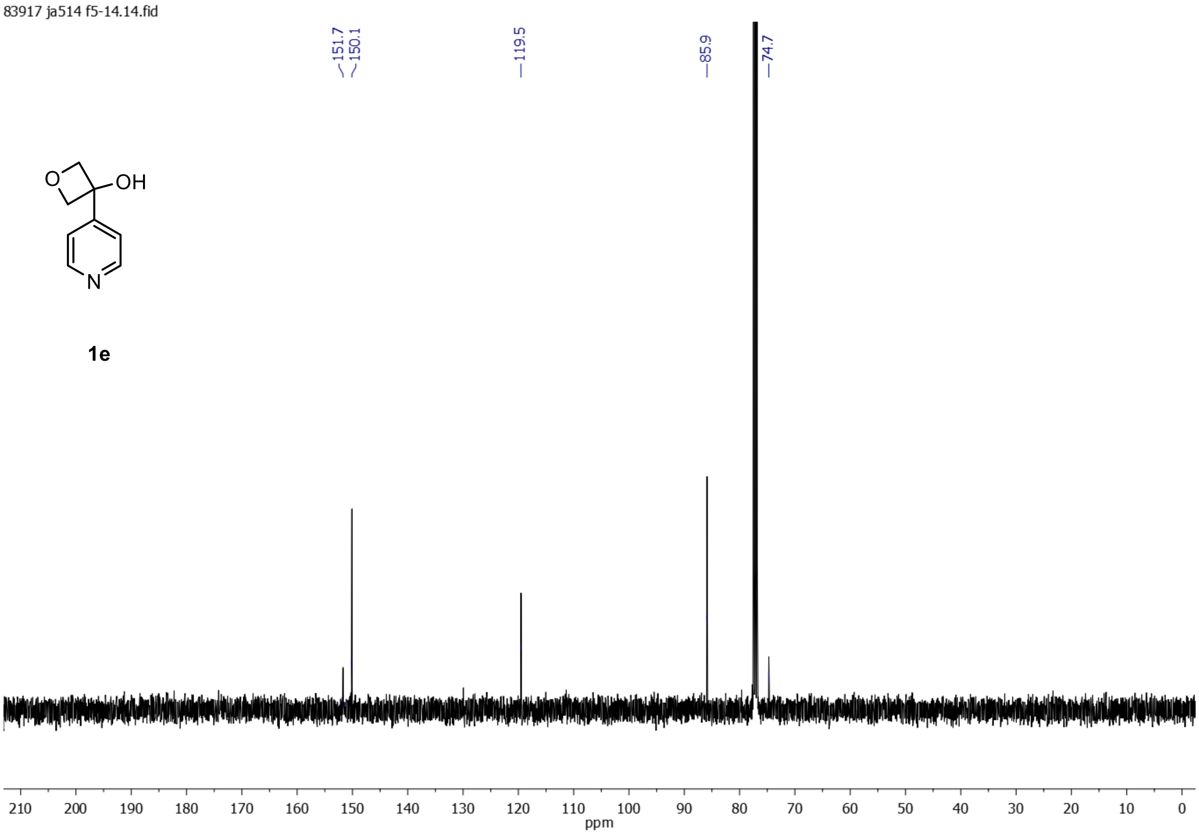

^{1}H NMR (400 MHz, $CDCl_3$) of **1f** (see procedure)

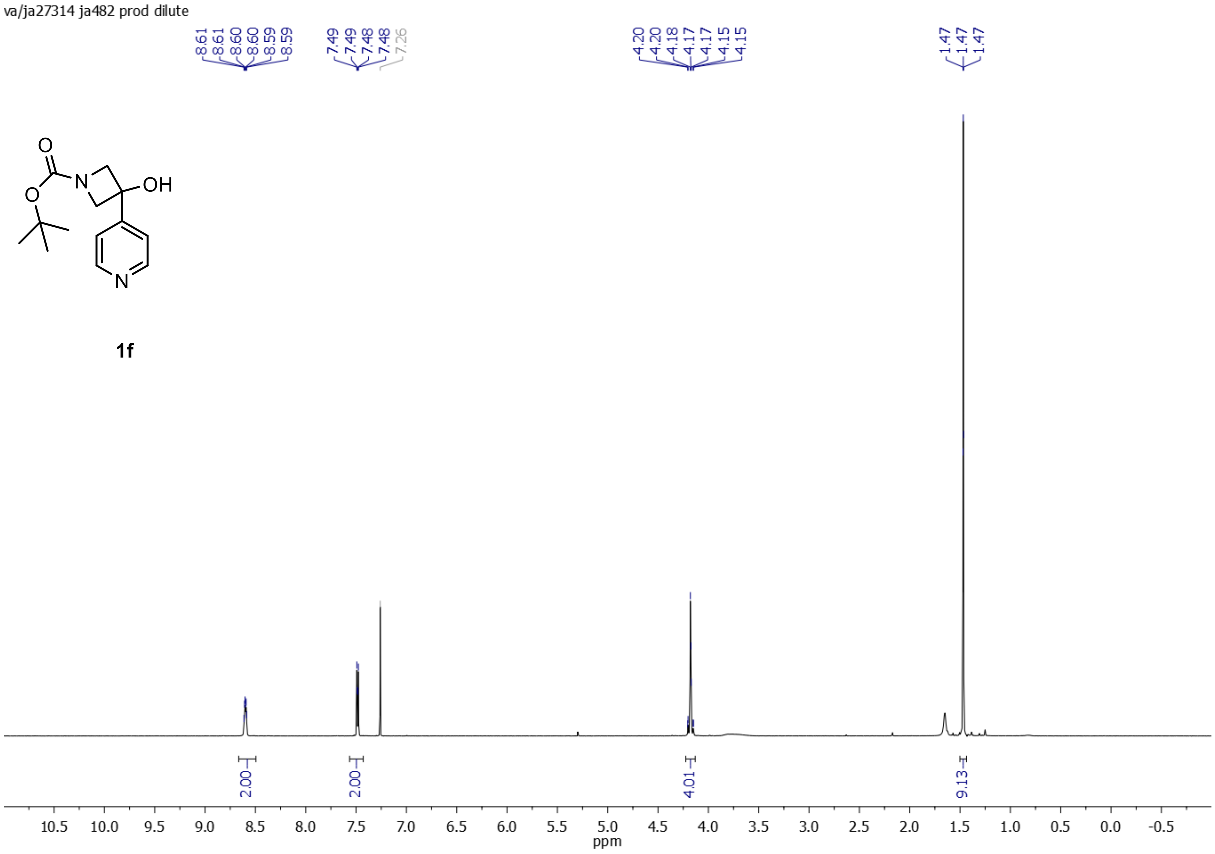

1f

^{13}C NMR (101 MHz, $CDCl_3$) of **1f**

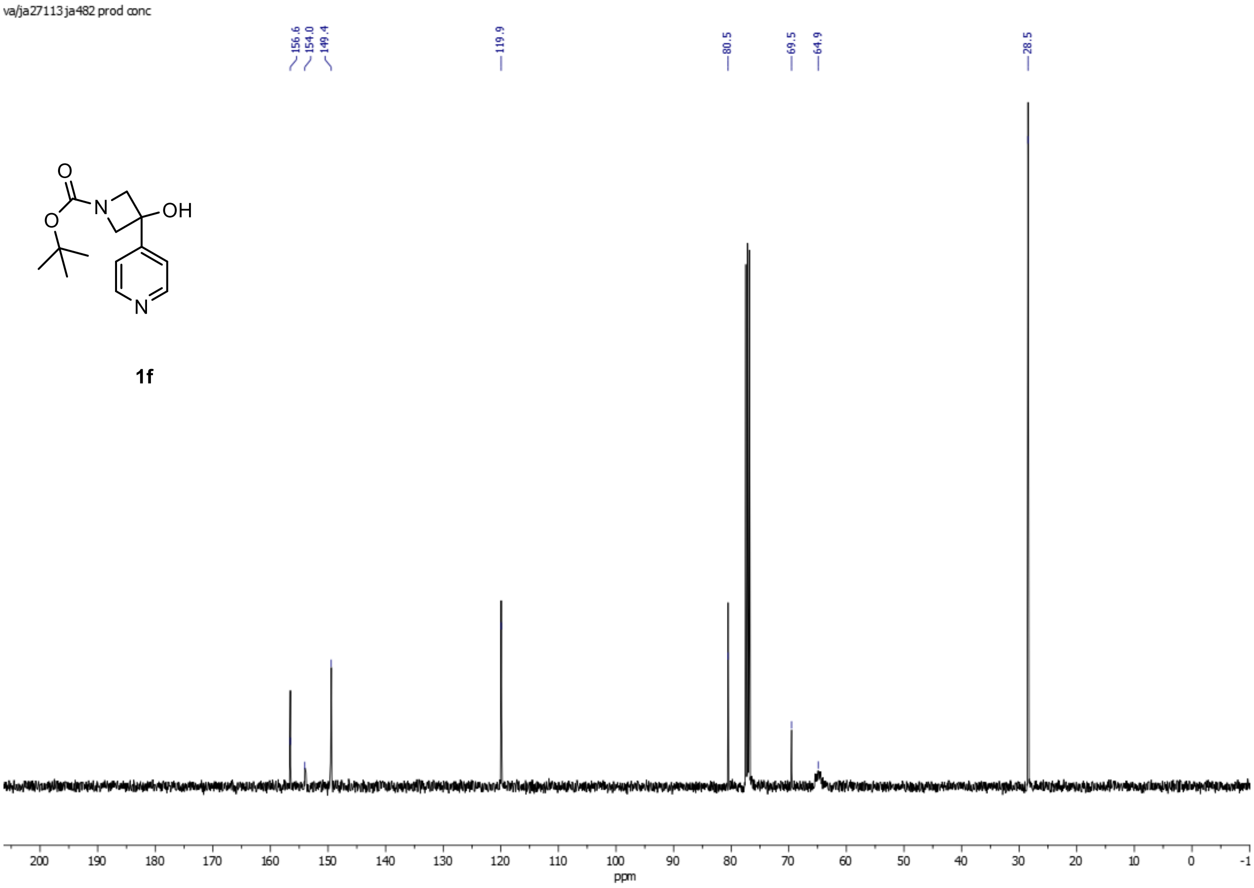

1f

^{1}H NMR (400 MHz, $CDCl_3$) of **1g** (see procedure)

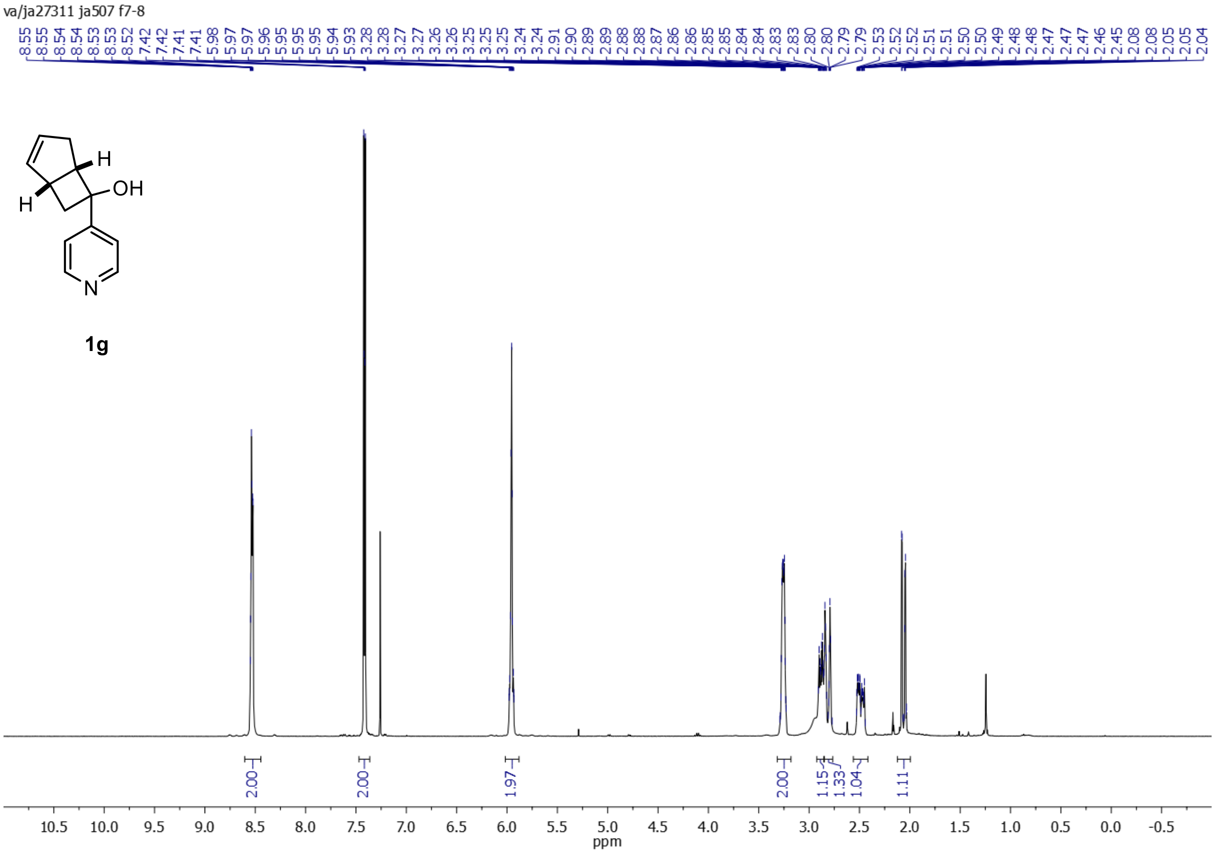

^{13}C NMR (101 MHz, $CDCl_3$) of **1g**

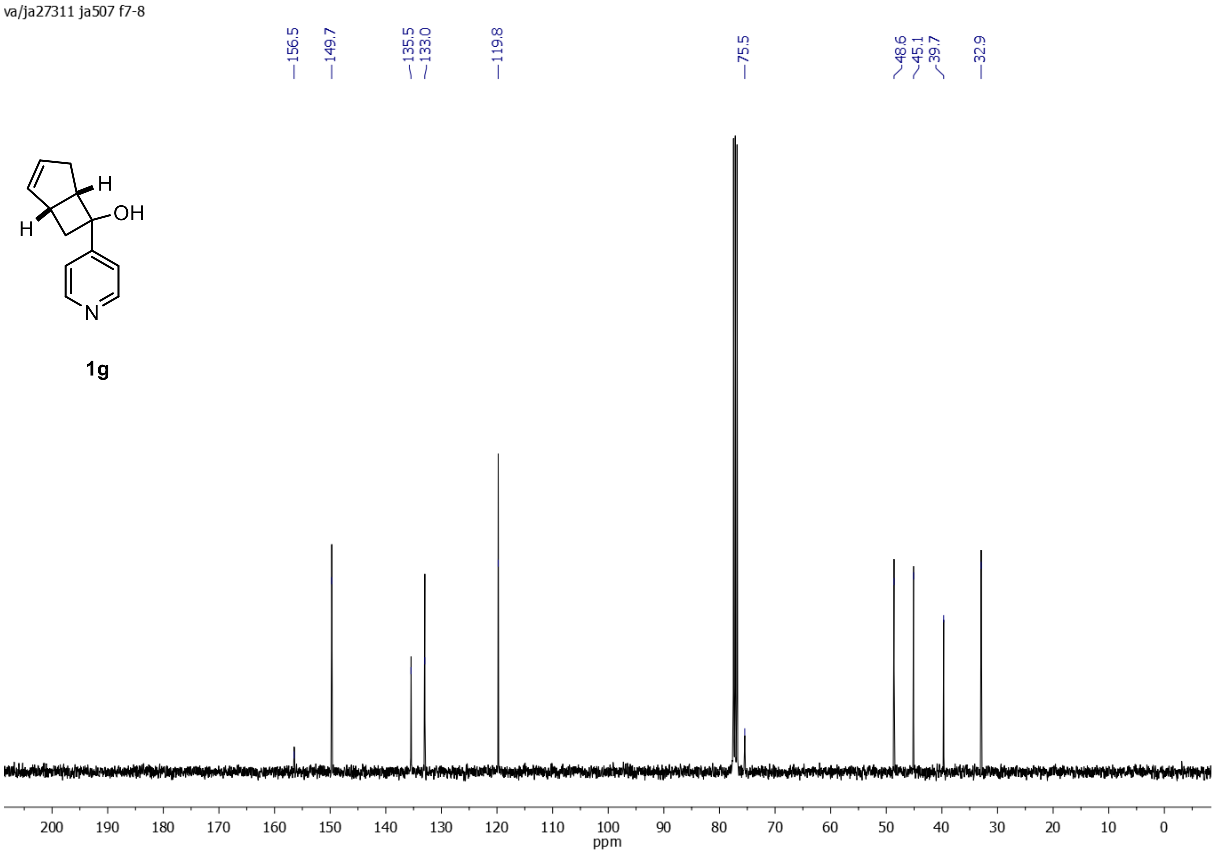

^{1}H NMR (400 MHz, $CDCl_3$) of **1h** (see procedure)

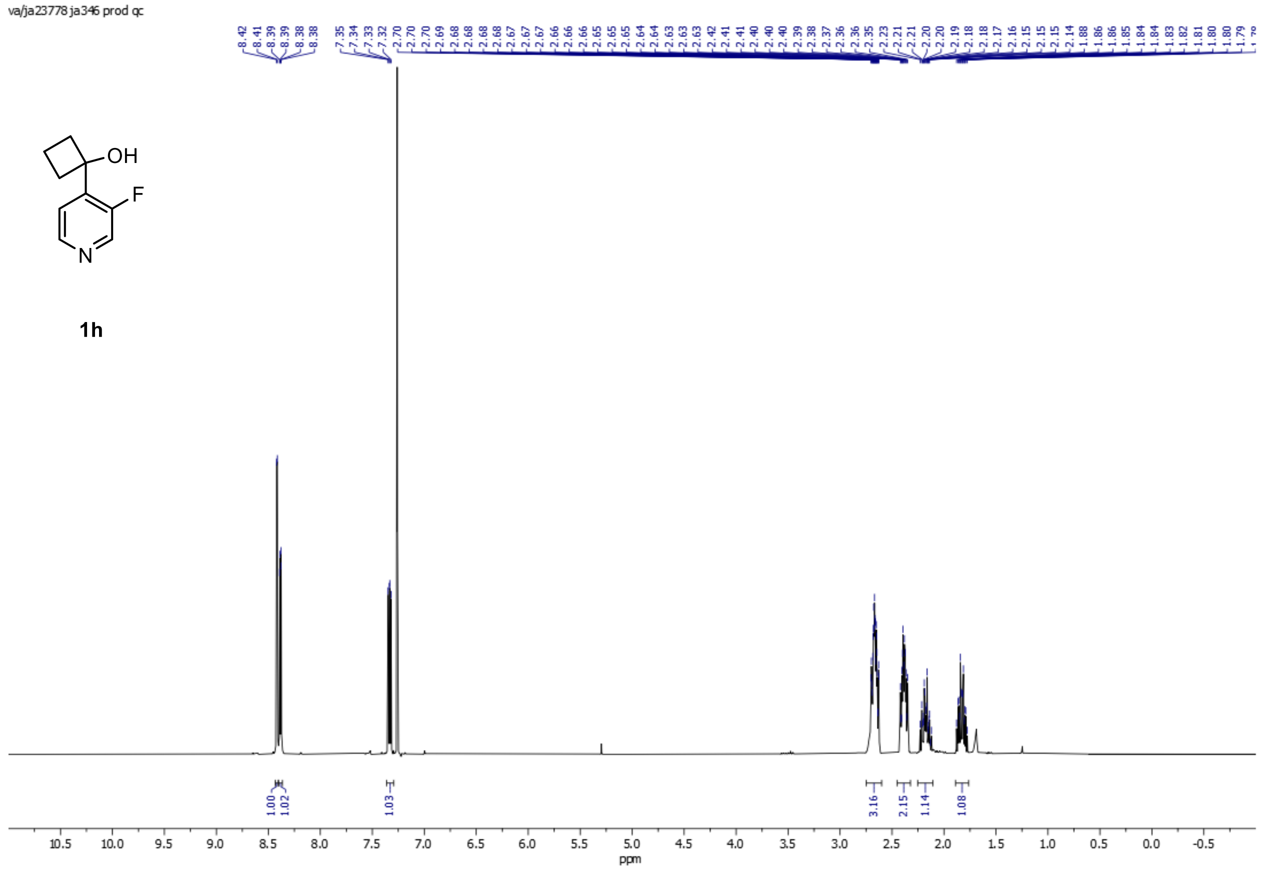

^{13}C NMR (101 MHz, $CDCl_3$) of **1h**

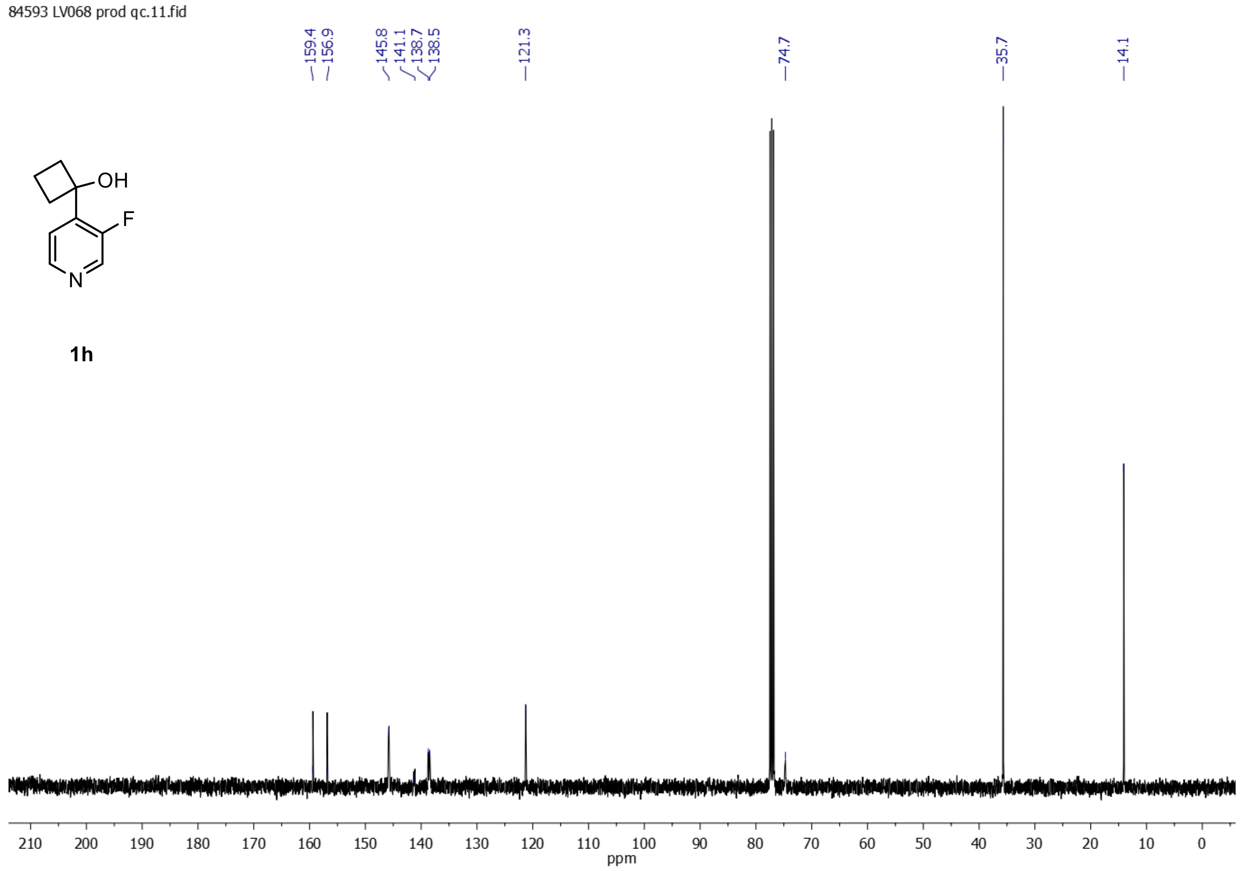

^{1}H NMR (400 MHz, $CDCl_3$) of **1i** (see procedure)

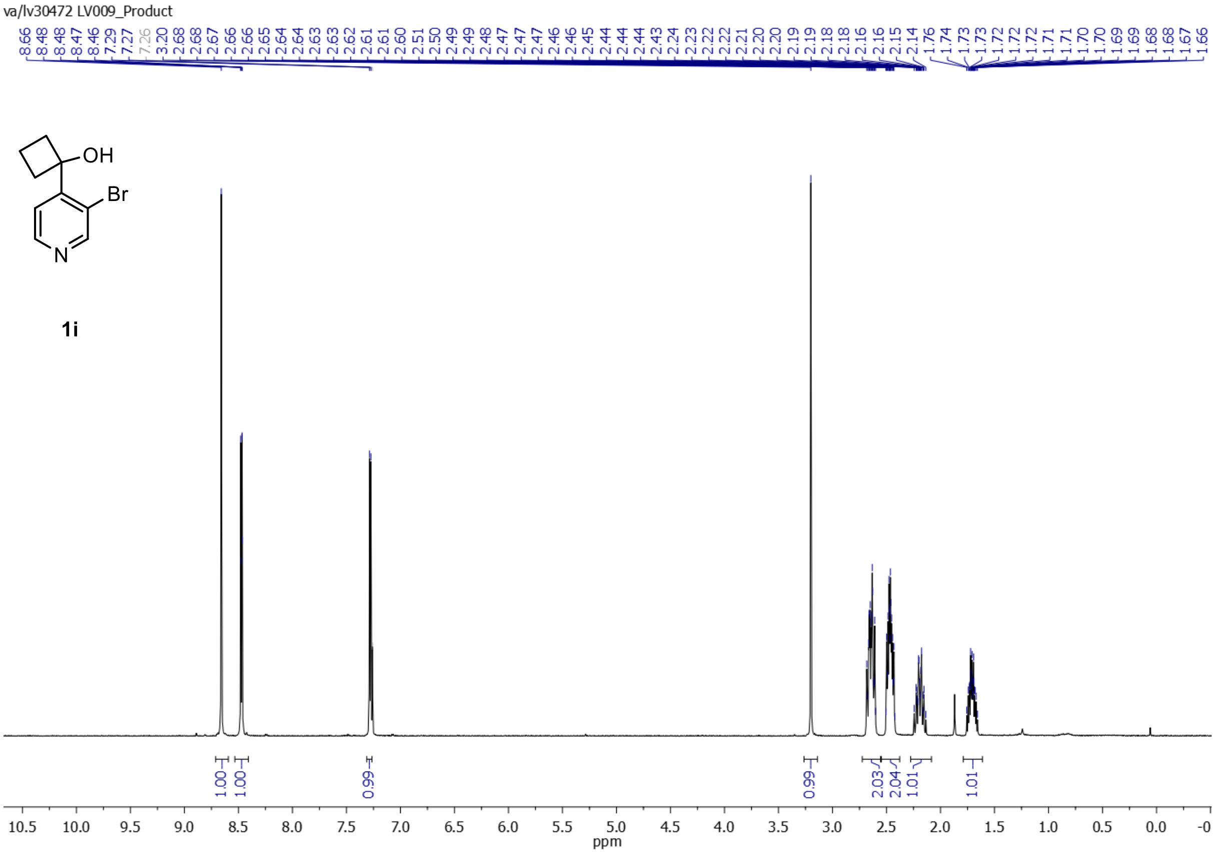

^{13}C NMR (101 MHz, $CDCl_3$) of **1i**

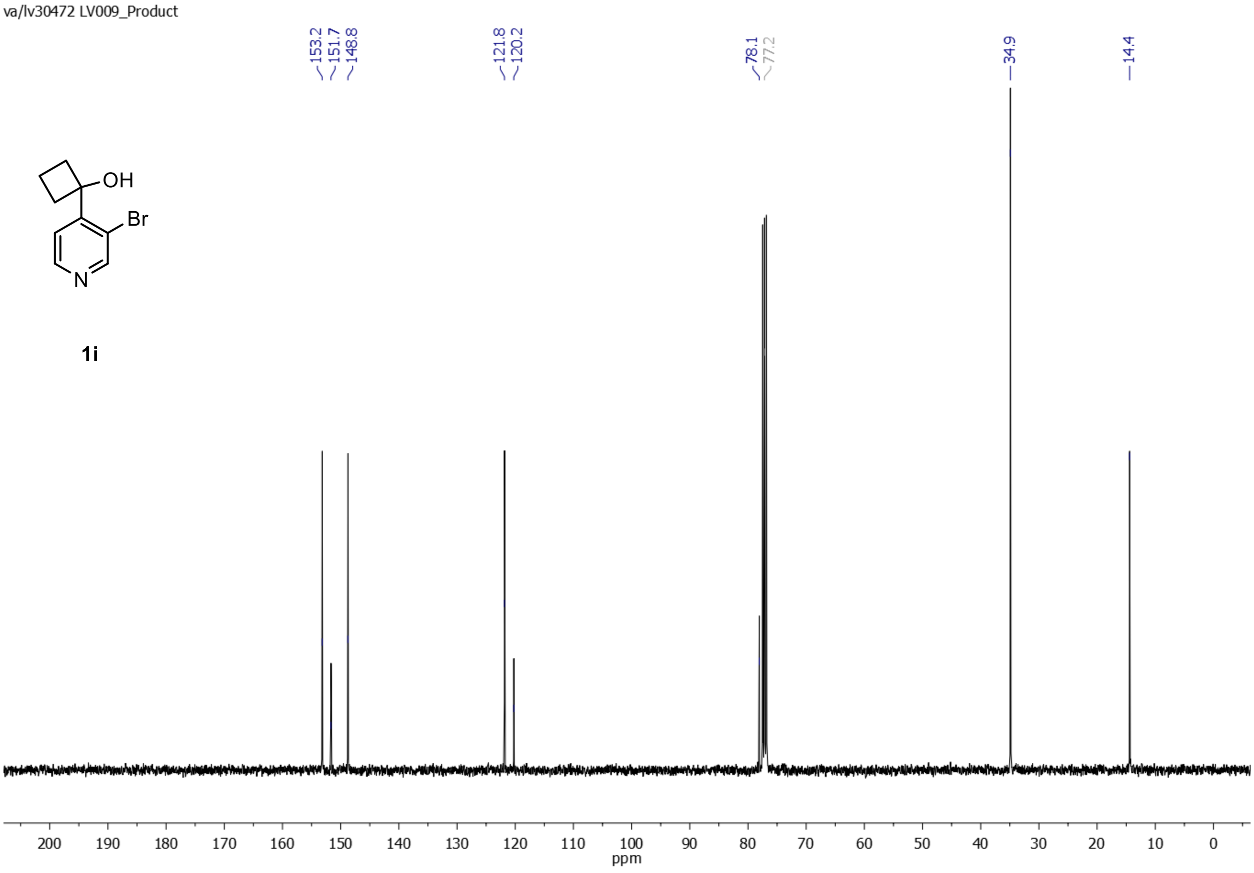

^{1}H NMR (400 MHz, $CDCl_3$) of **1j** (see procedure)

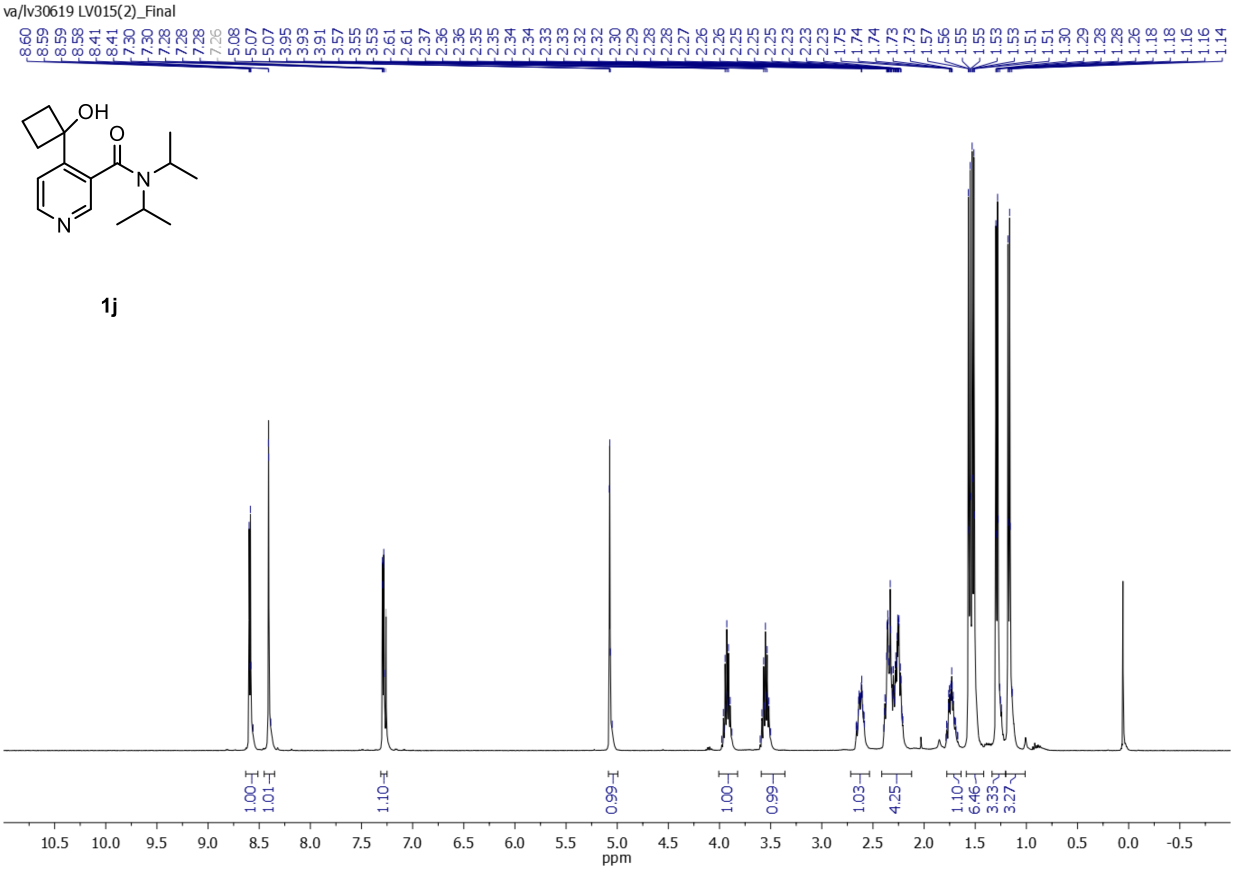

^{13}C NMR (101 MHz, $CDCl_3$) of **1j**

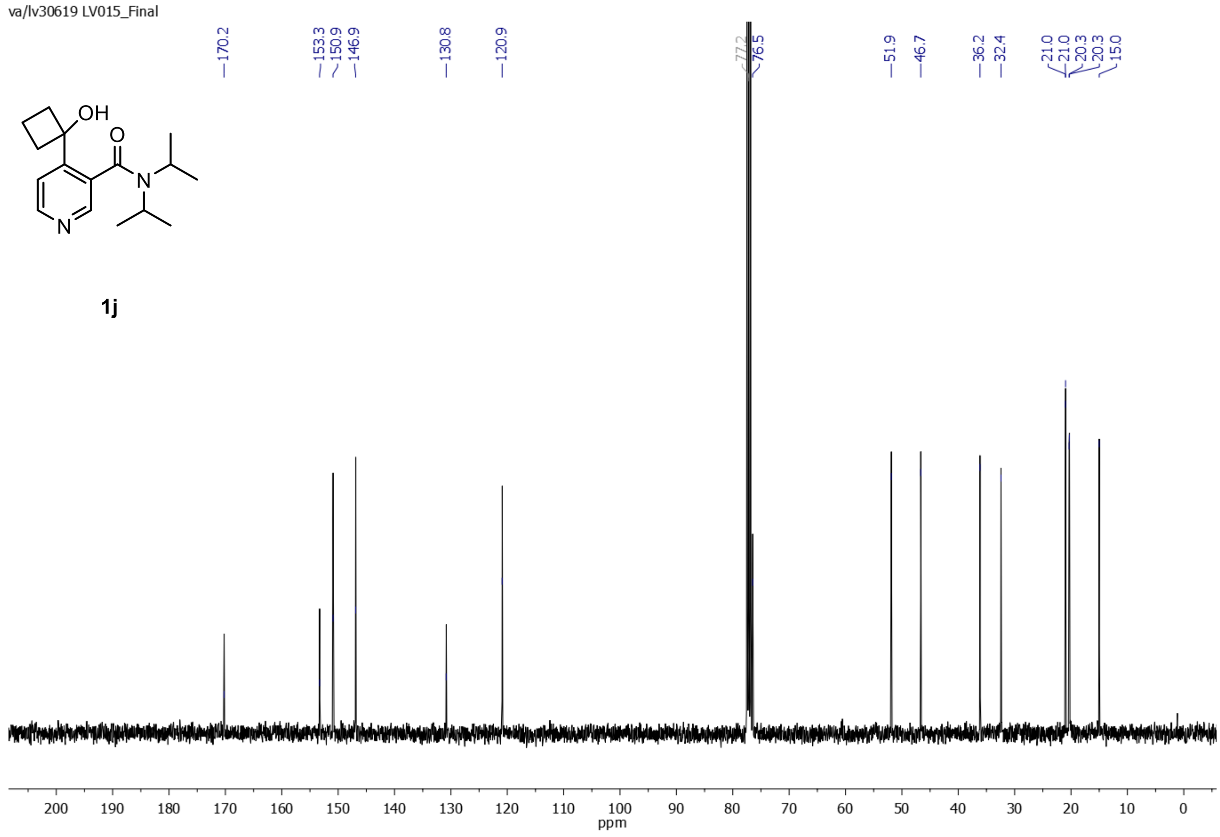

^{1}H NMR (400 MHz, $CDCl_3$) of **1k** (see procedure)

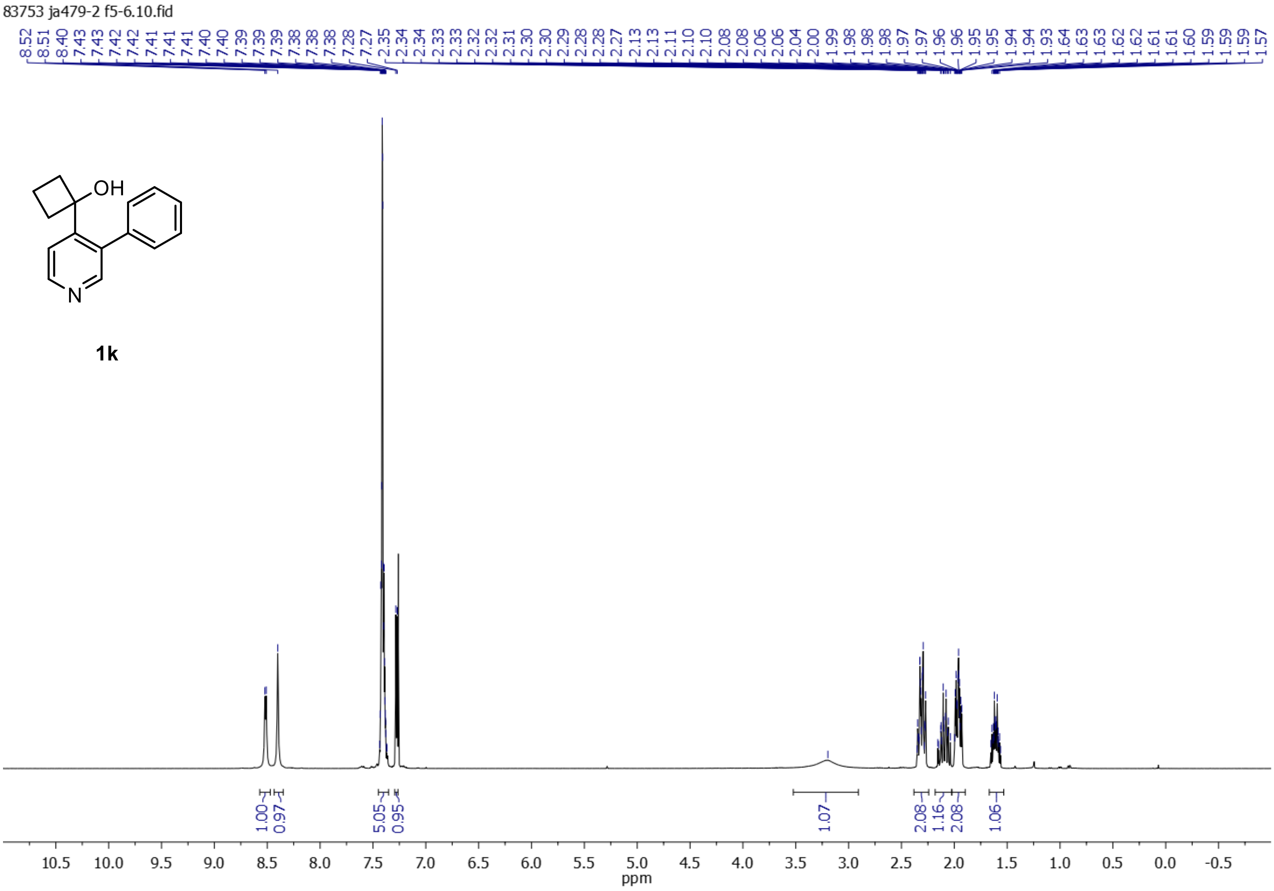

^{13}C NMR (101 MHz, $CDCl_3$) of **1k**

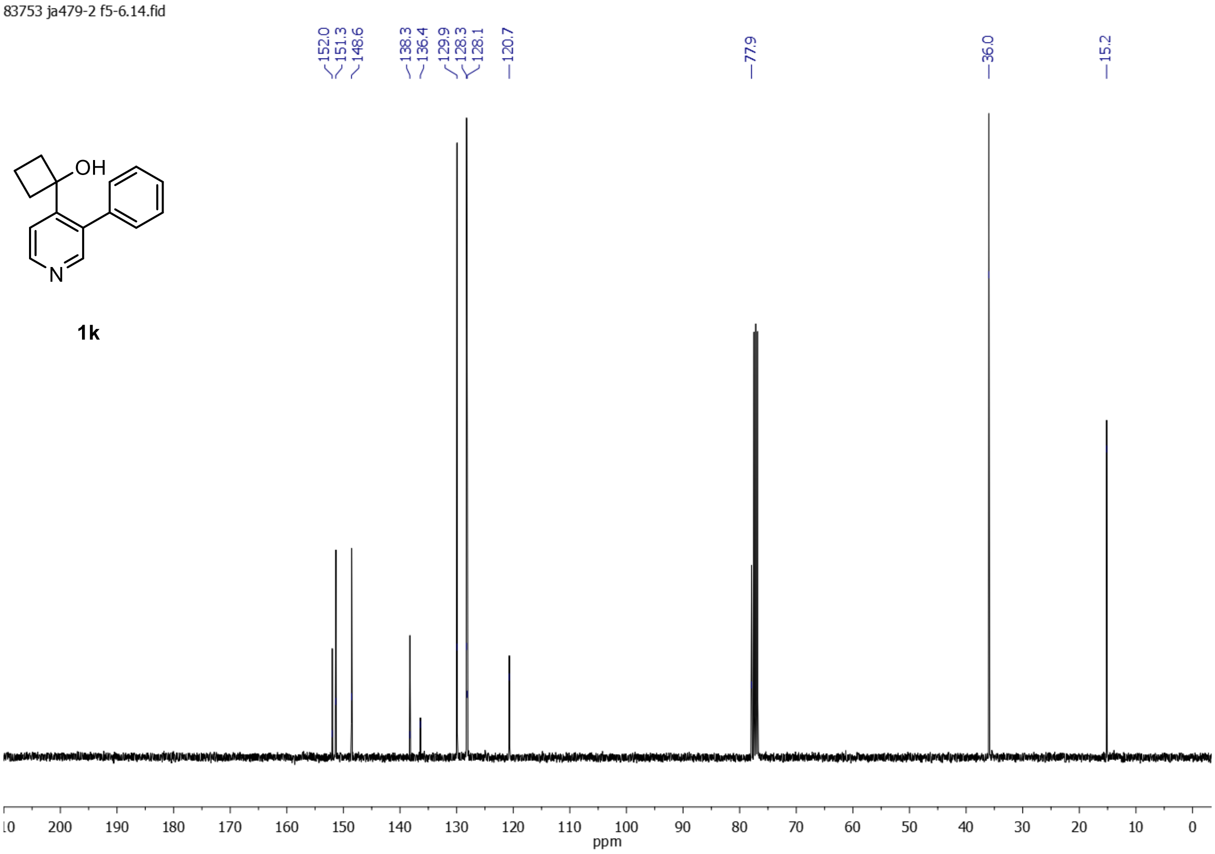

^{1}H NMR (400 MHz, CDCl$_3$) of **1l** (see procedure)

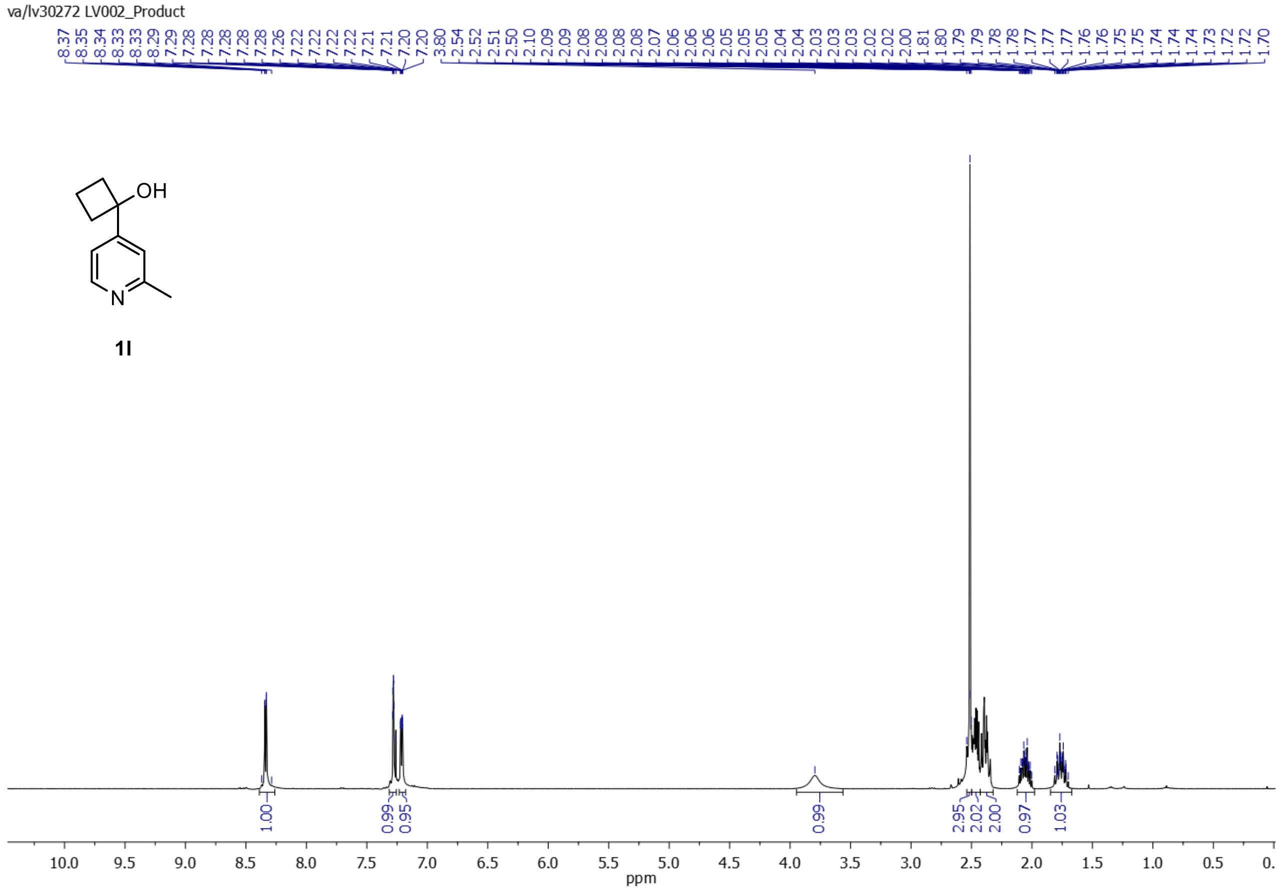

^{13}C NMR (101 MHz, CDCl$_3$) of **1l**

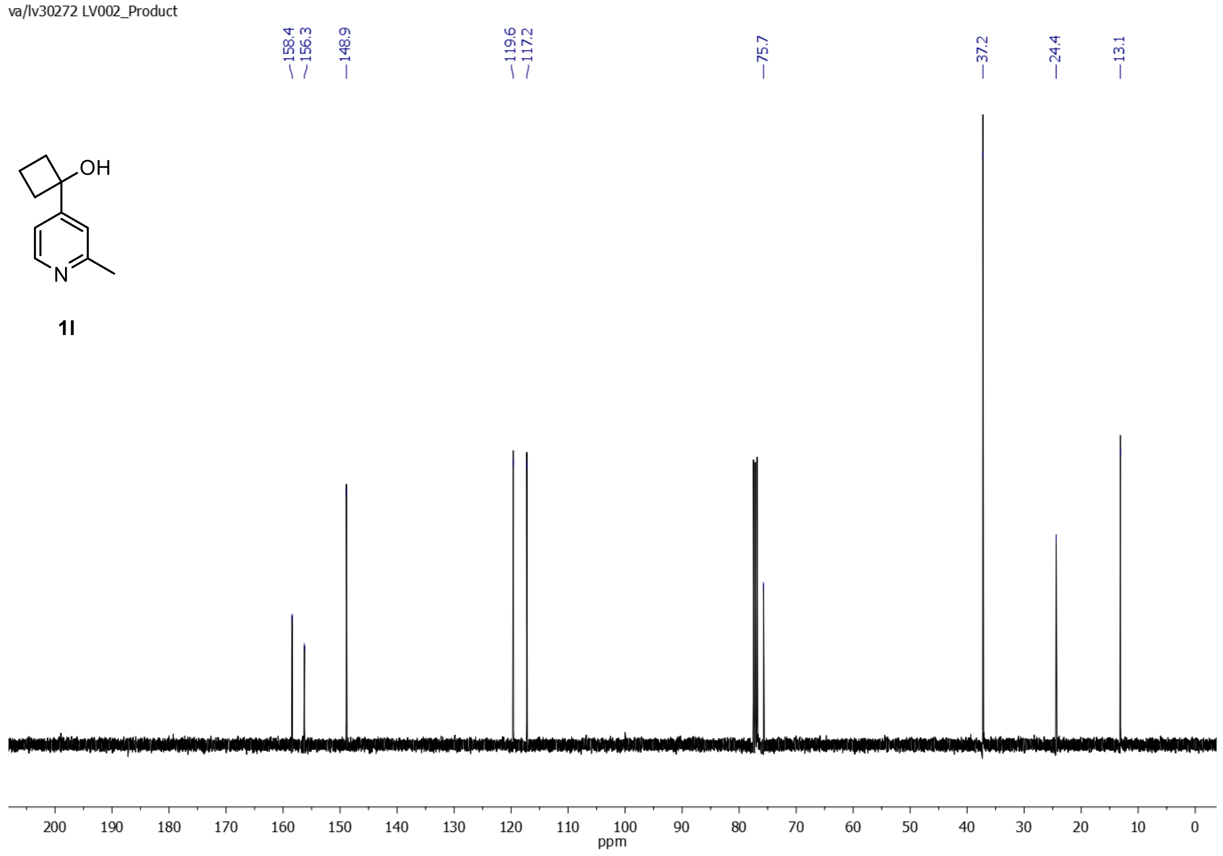

^{1}H NMR (400 MHz, $CDCl_3$) of **1m** (see procedure)

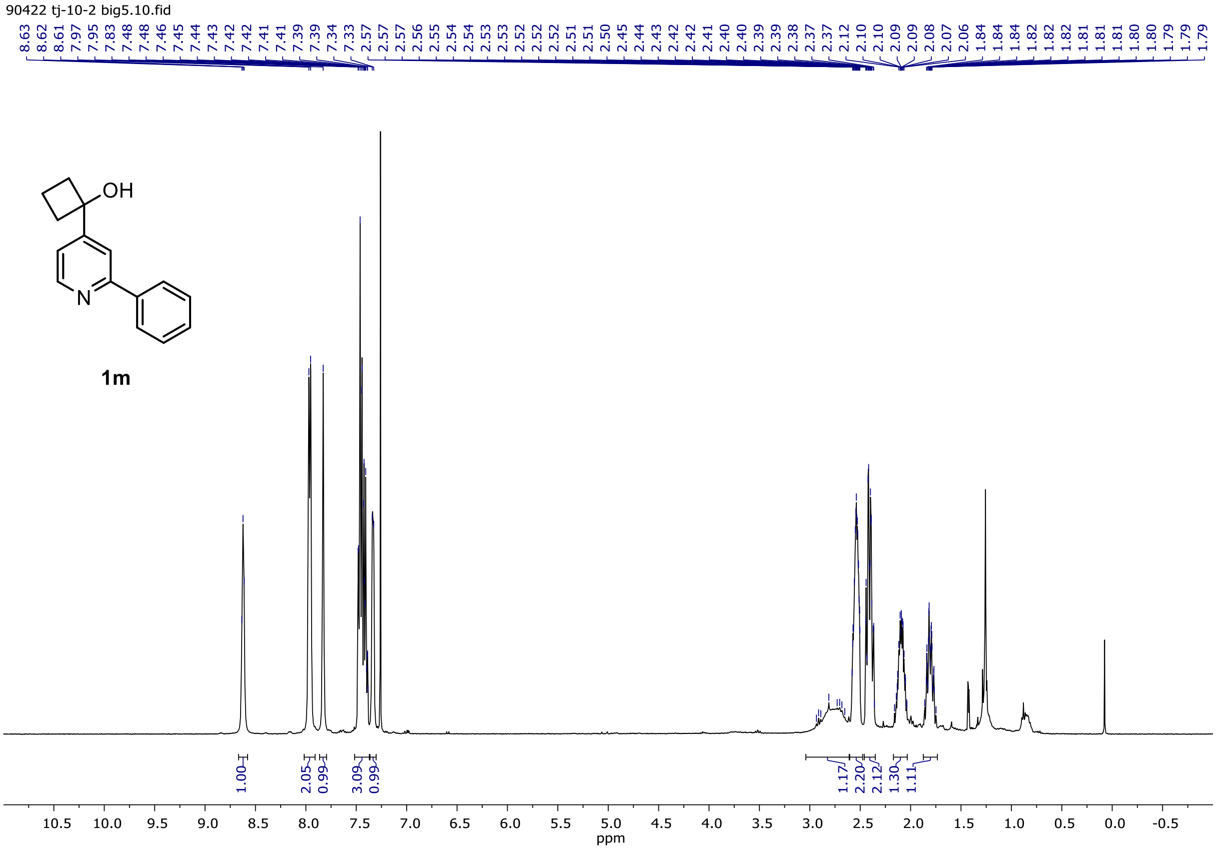

^{13}C NMR (101 MHz, $CDCl_3$) of **1m**

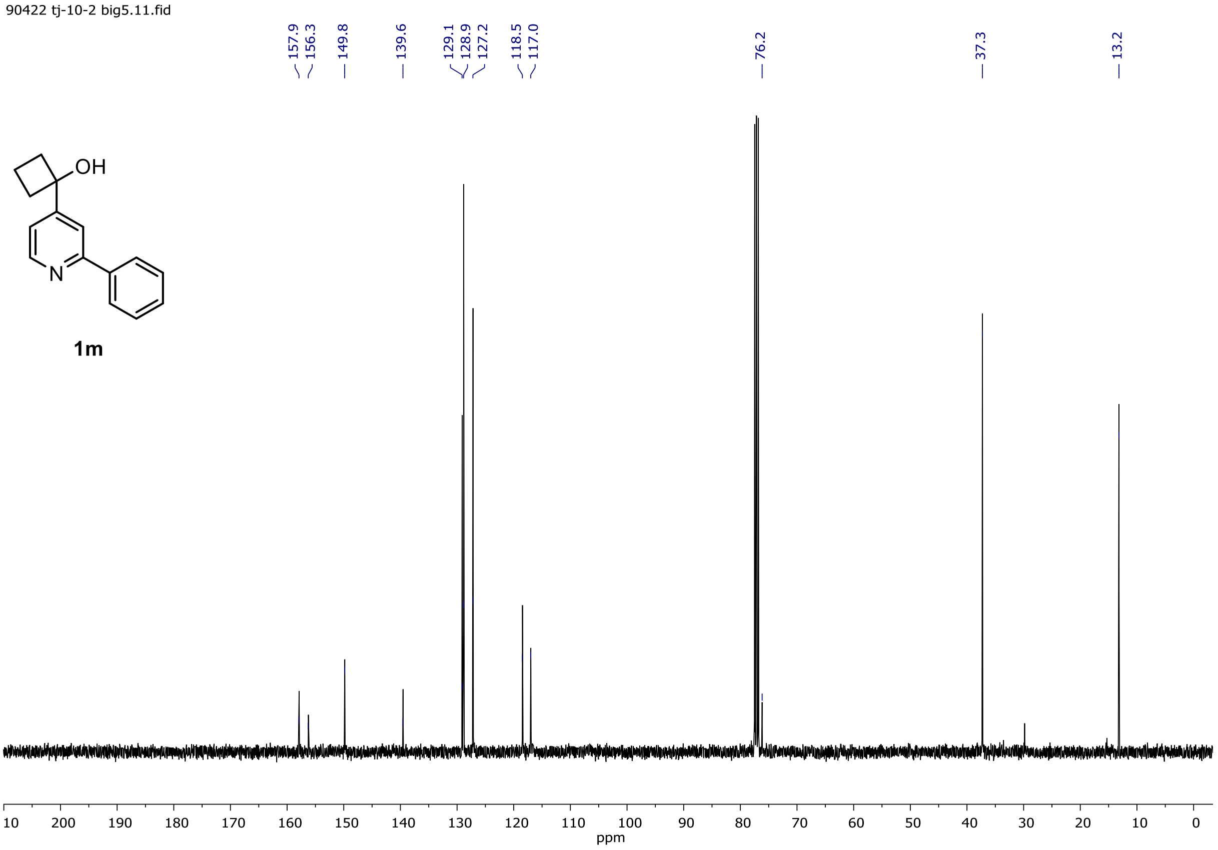

^{1}H NMR (400 MHz, $CDCl_3$) of **1n** (see procedure)

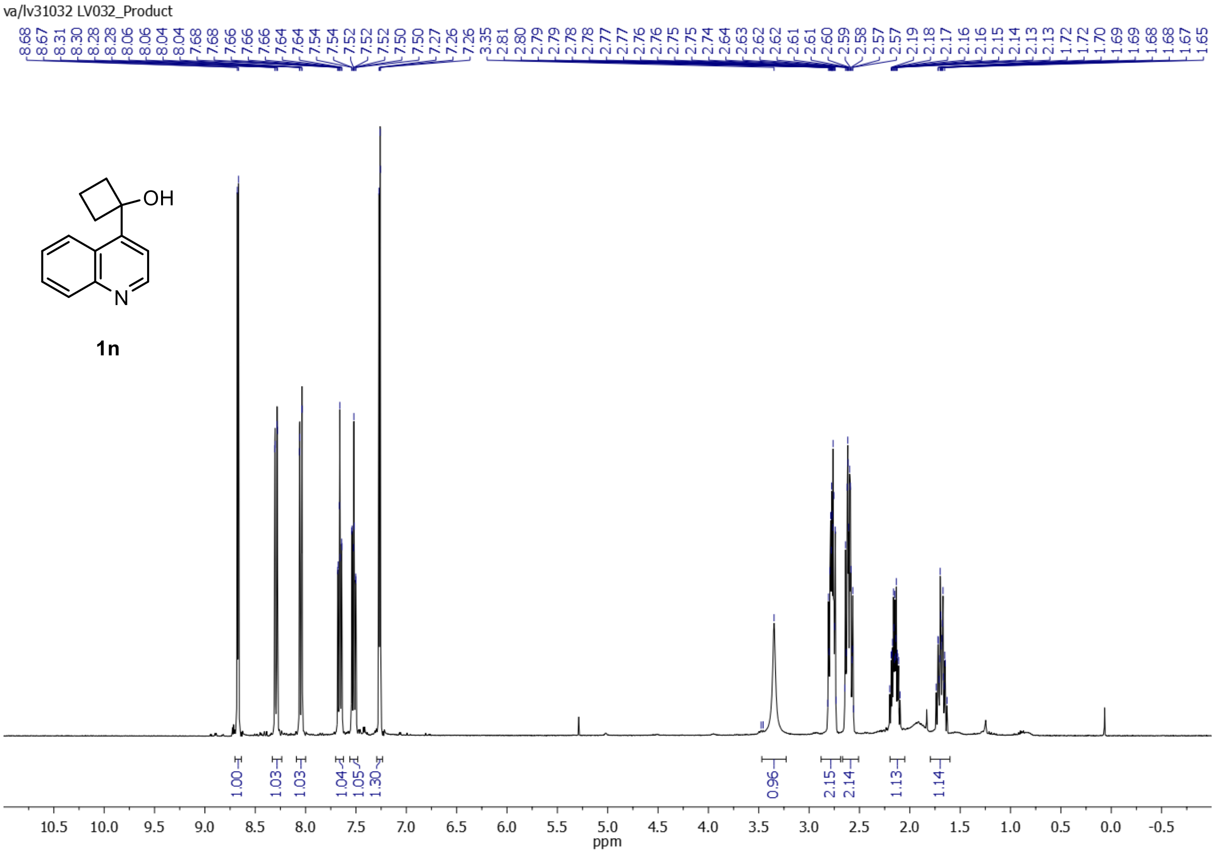

^{13}C NMR (101 MHz, $CDCl_3$) of **1n**

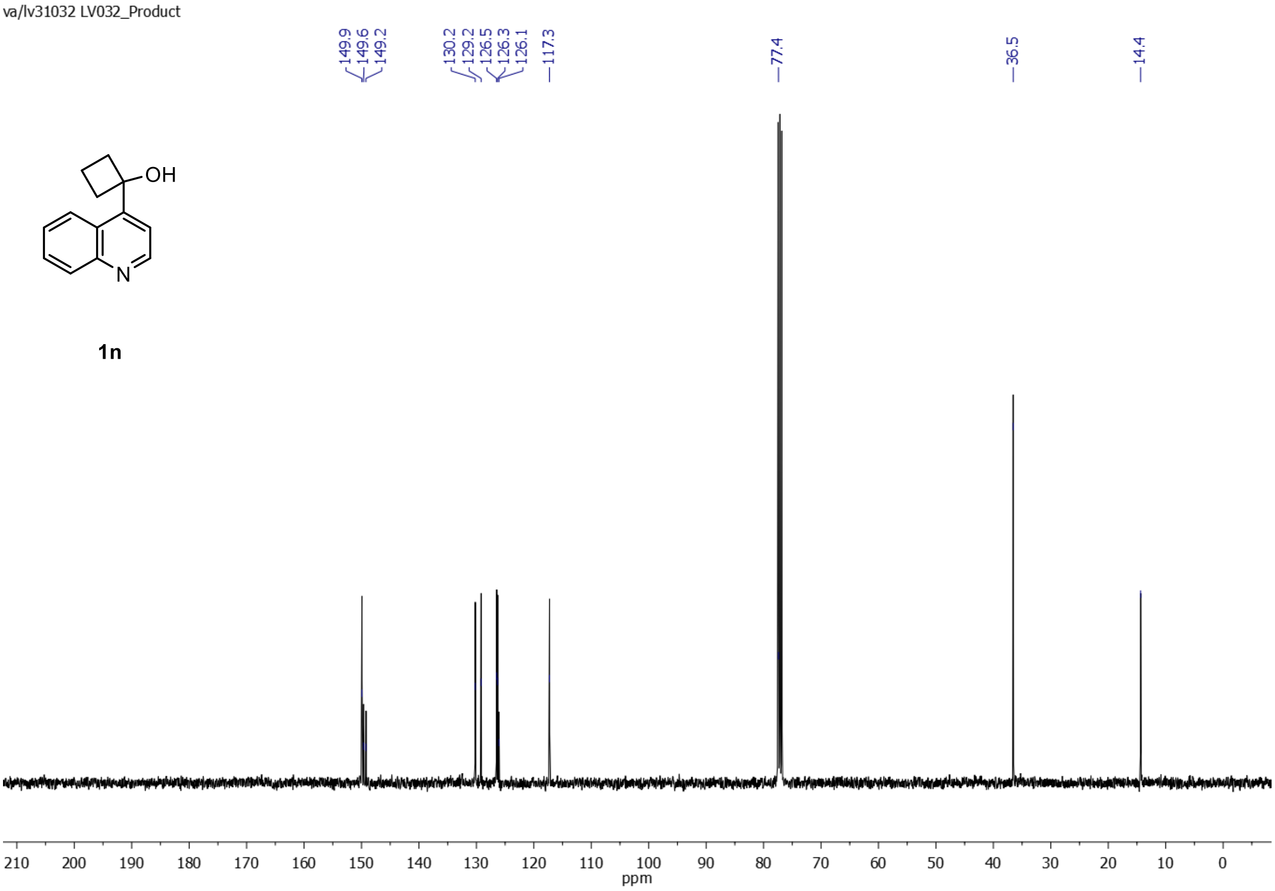

^{1}H NMR (500 MHz, $CDCl_3$) of **1o** (see procedure)

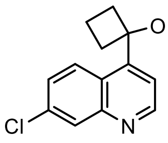

1o

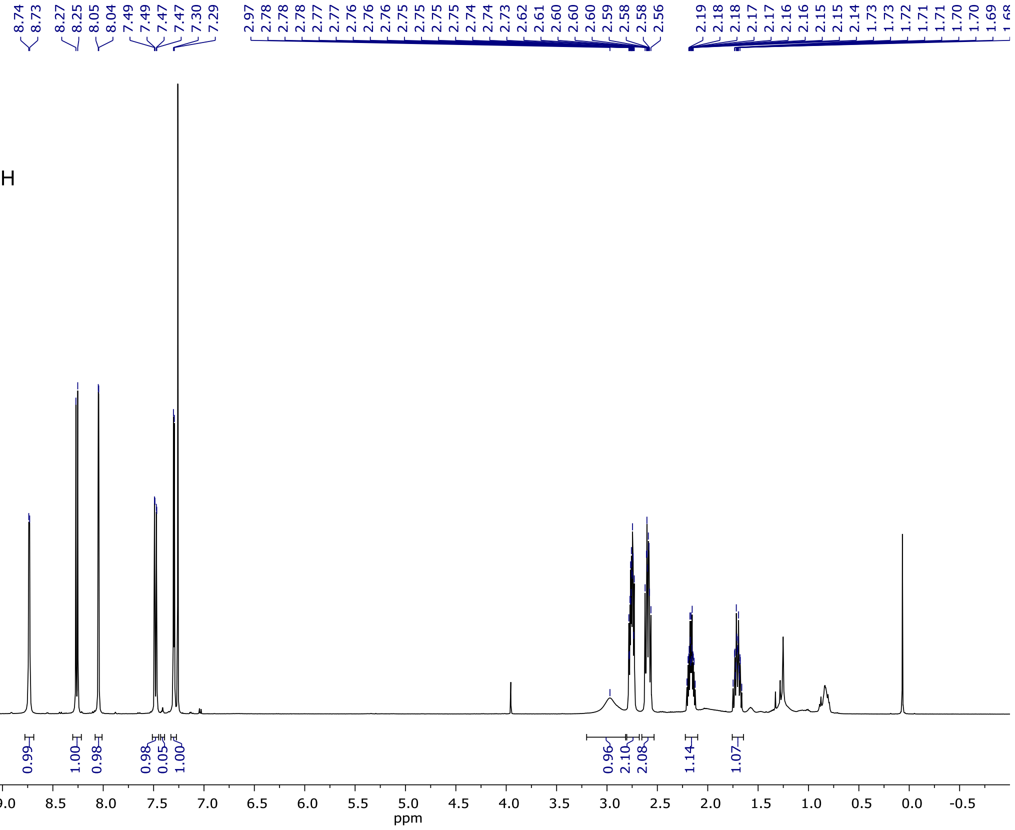

^{13}C NMR (126 MHz, $CDCl_3$) of **1o**

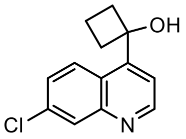

1o

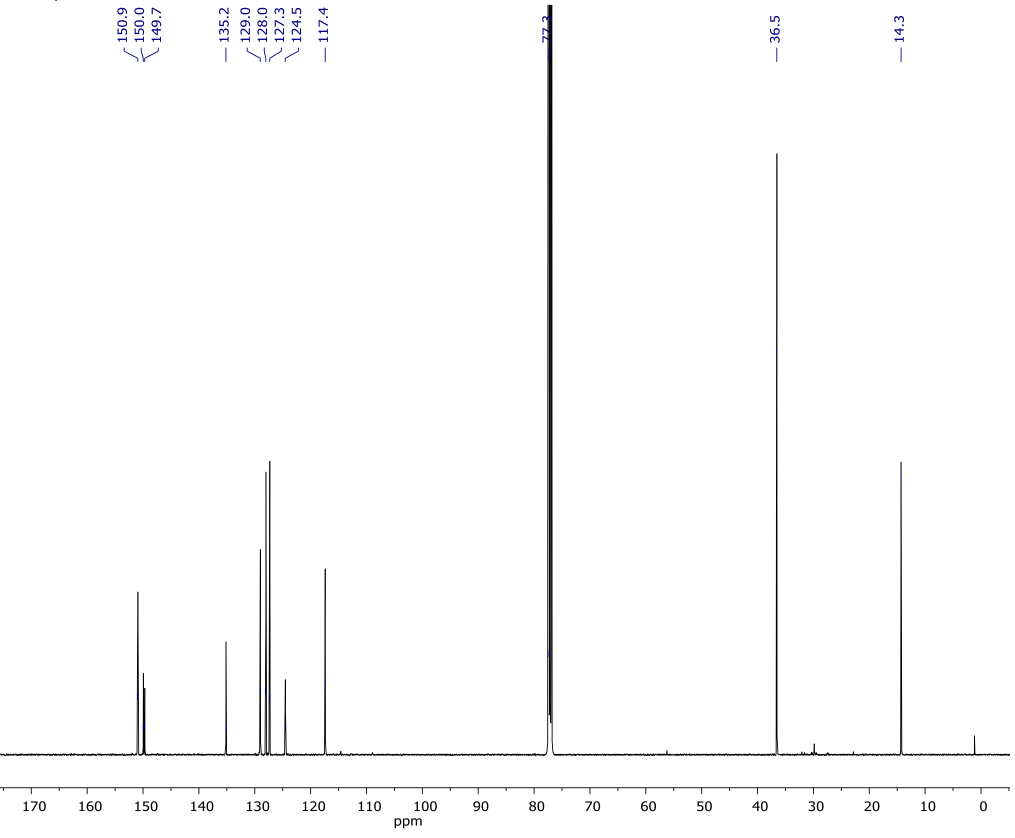

^{1}H NMR (500 MHz, $CDCl_3$) of **1p** (see procedure)

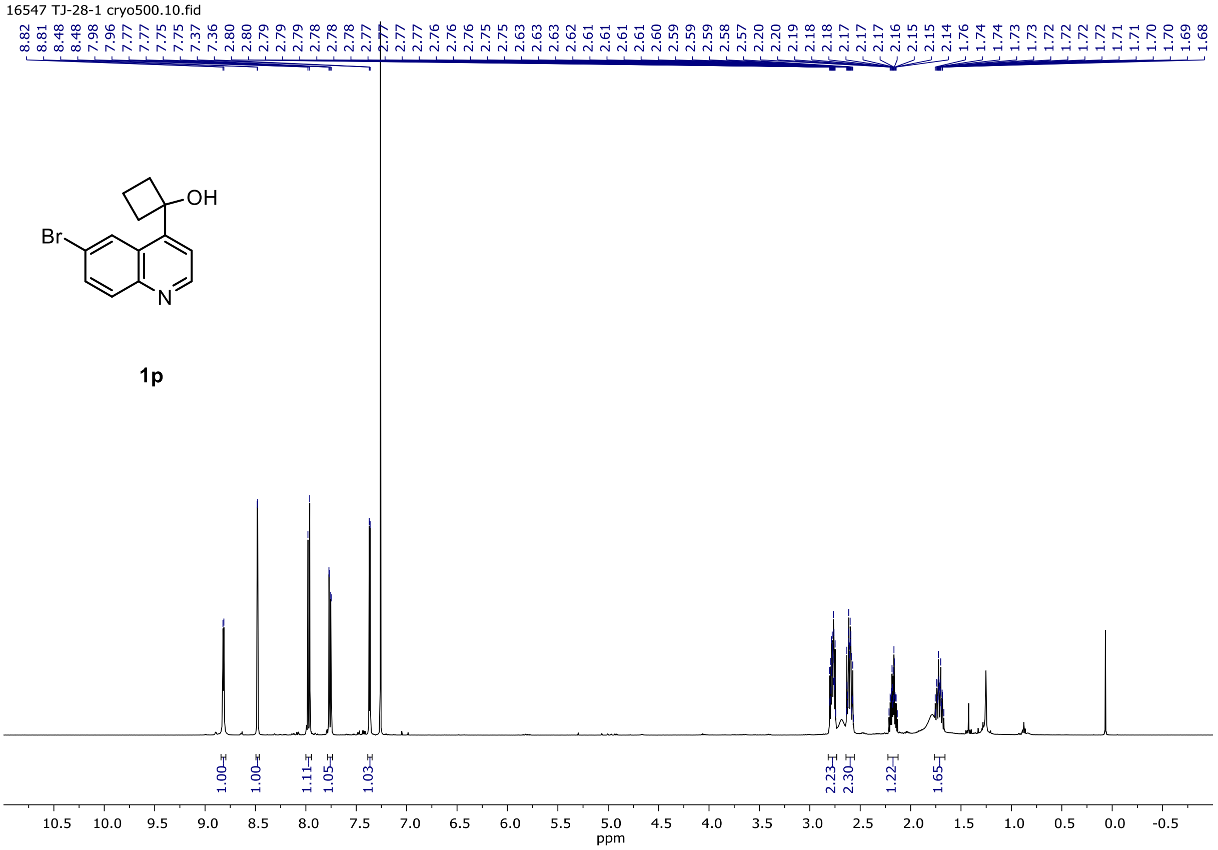

^{13}C NMR (126 MHz, $CDCl_3$) of **1p**

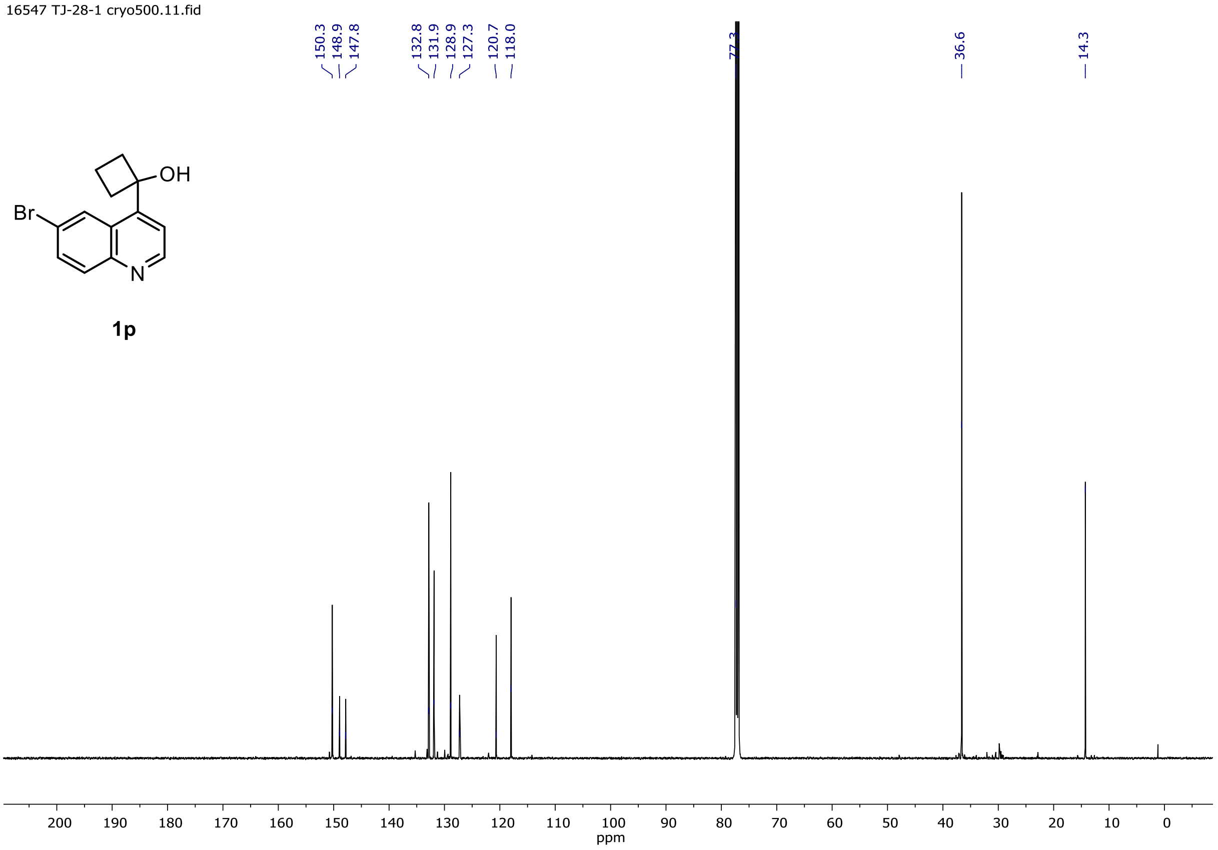

^{1}H NMR (400 MHz, $CDCl_3$) of **1q** (see procedure)

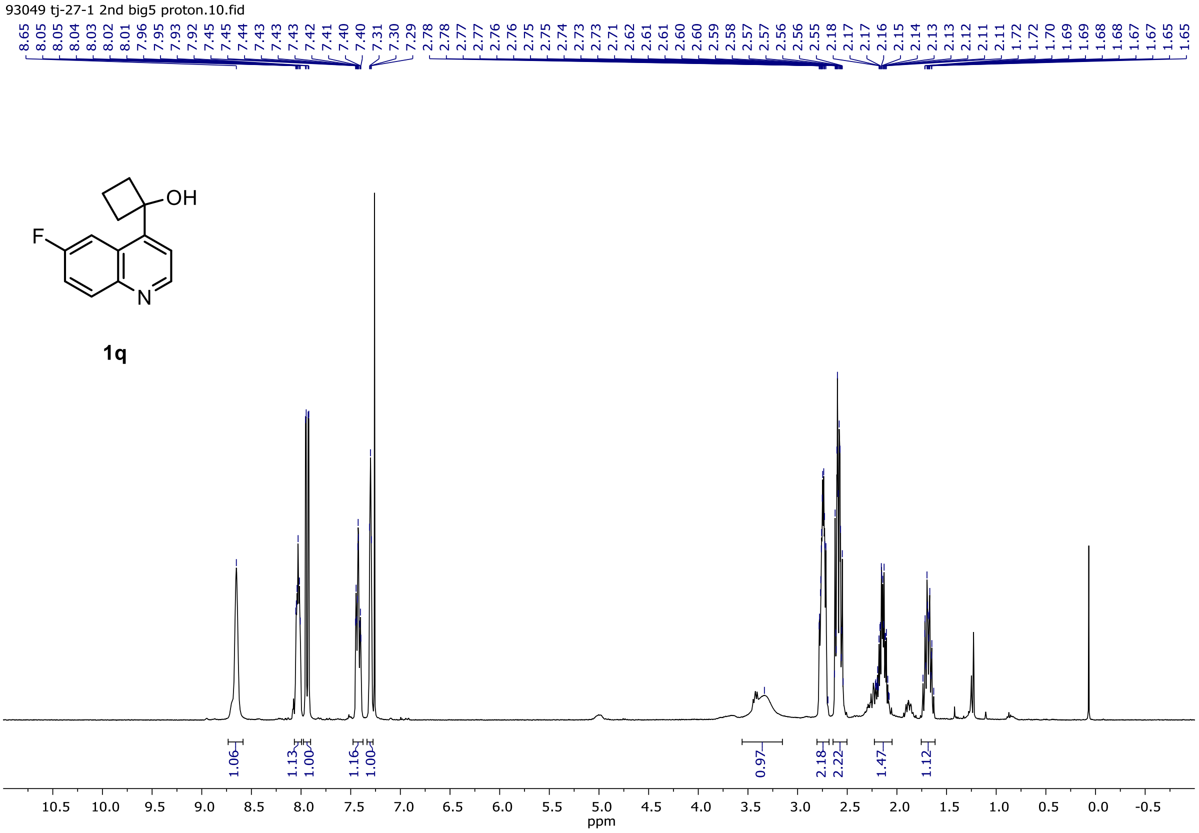

^{13}C NMR (101 MHz, $CDCl_3$) of **1q**

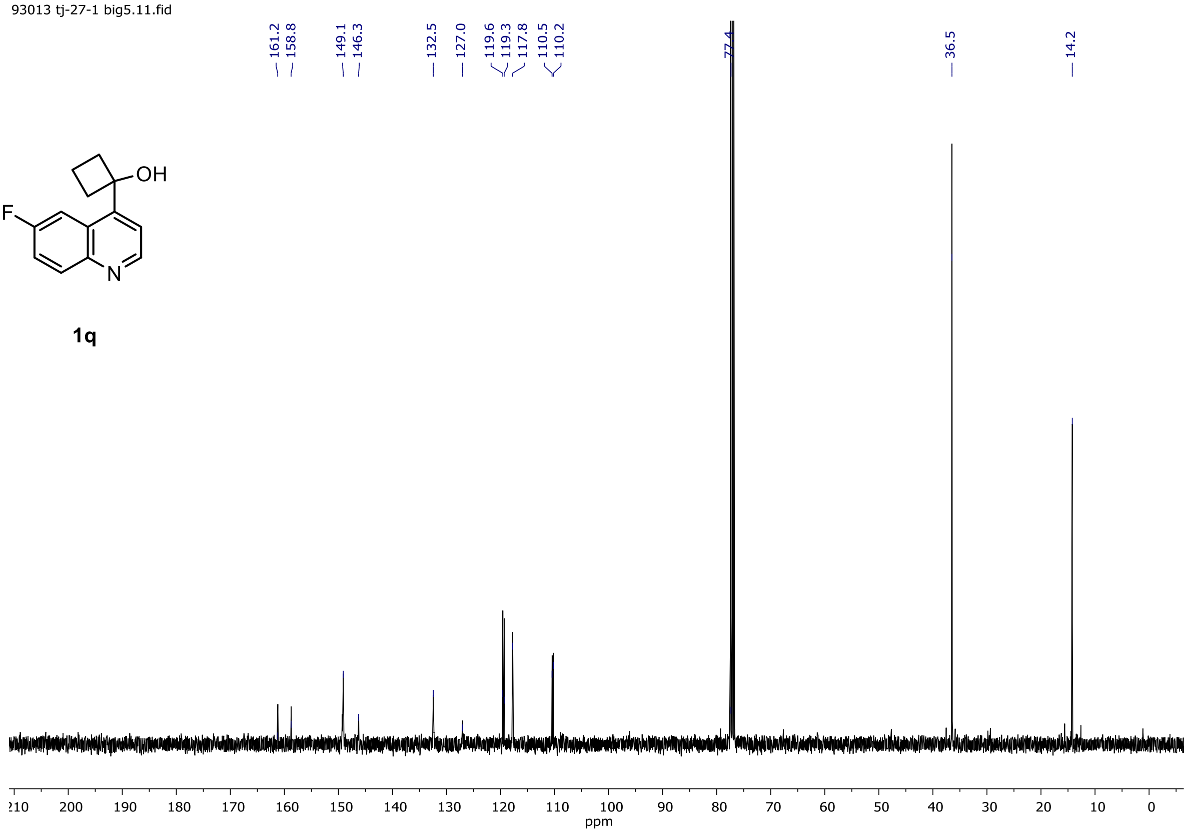

^{1}H NMR (400 MHz, $CDCl_3$) of **1r** (see procedure)

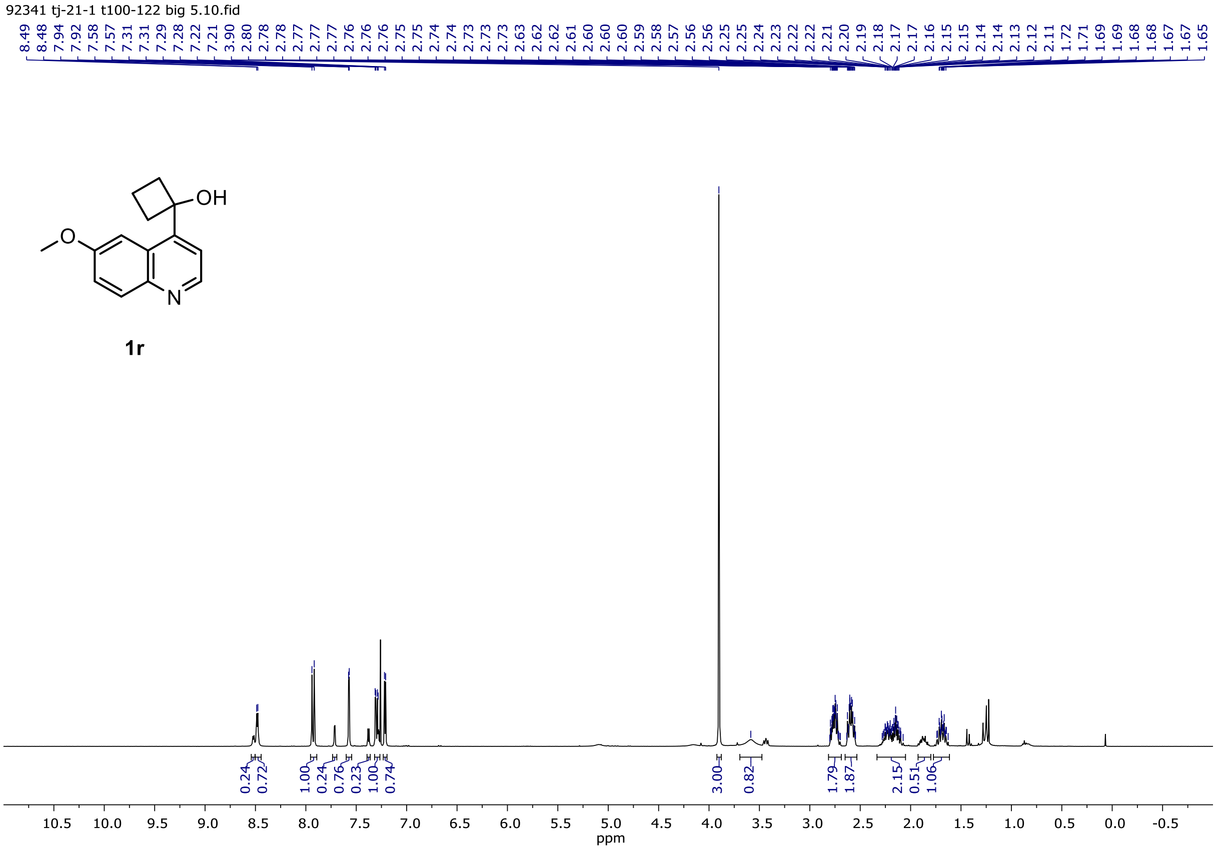

^{13}C NMR (101 MHz, $CDCl_3$) of **1r**

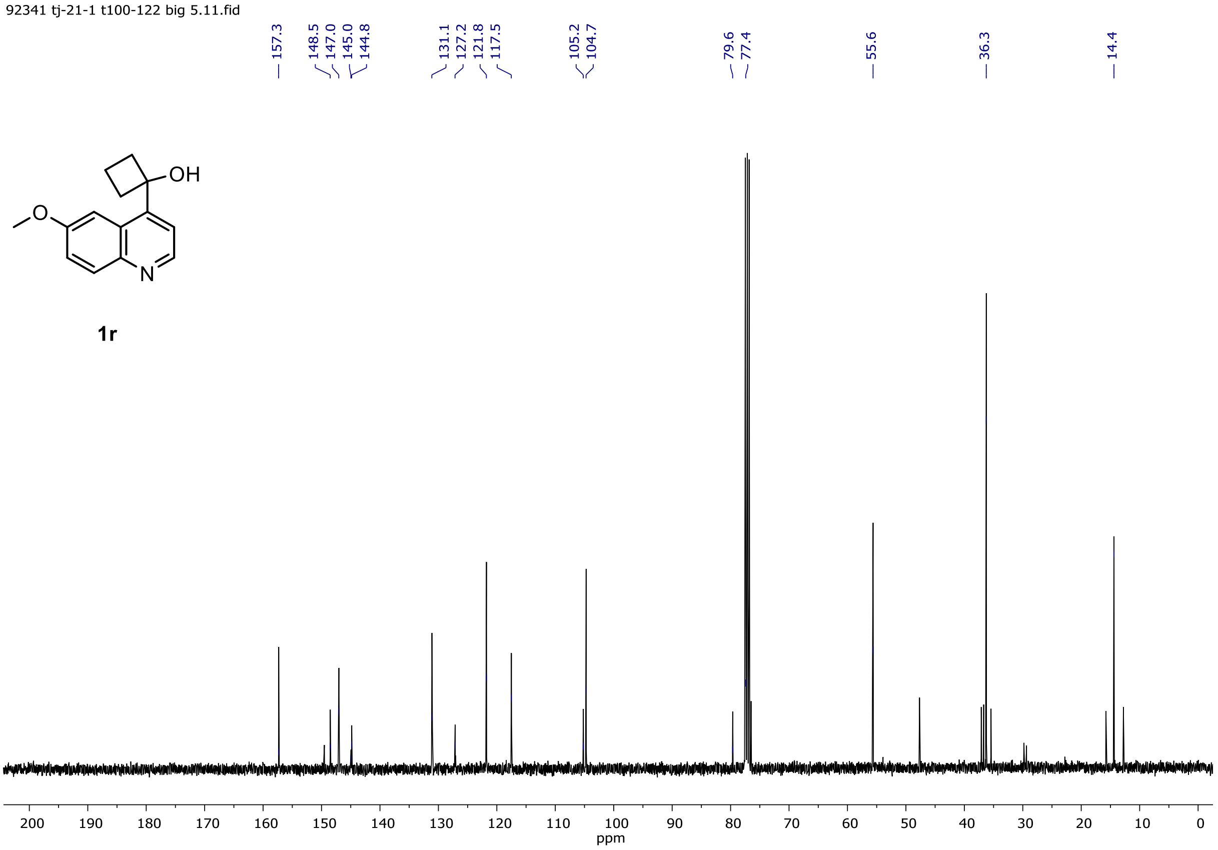

^{1}H NMR (500 MHz, $CDCl_3$) of **1s** (see procedure)

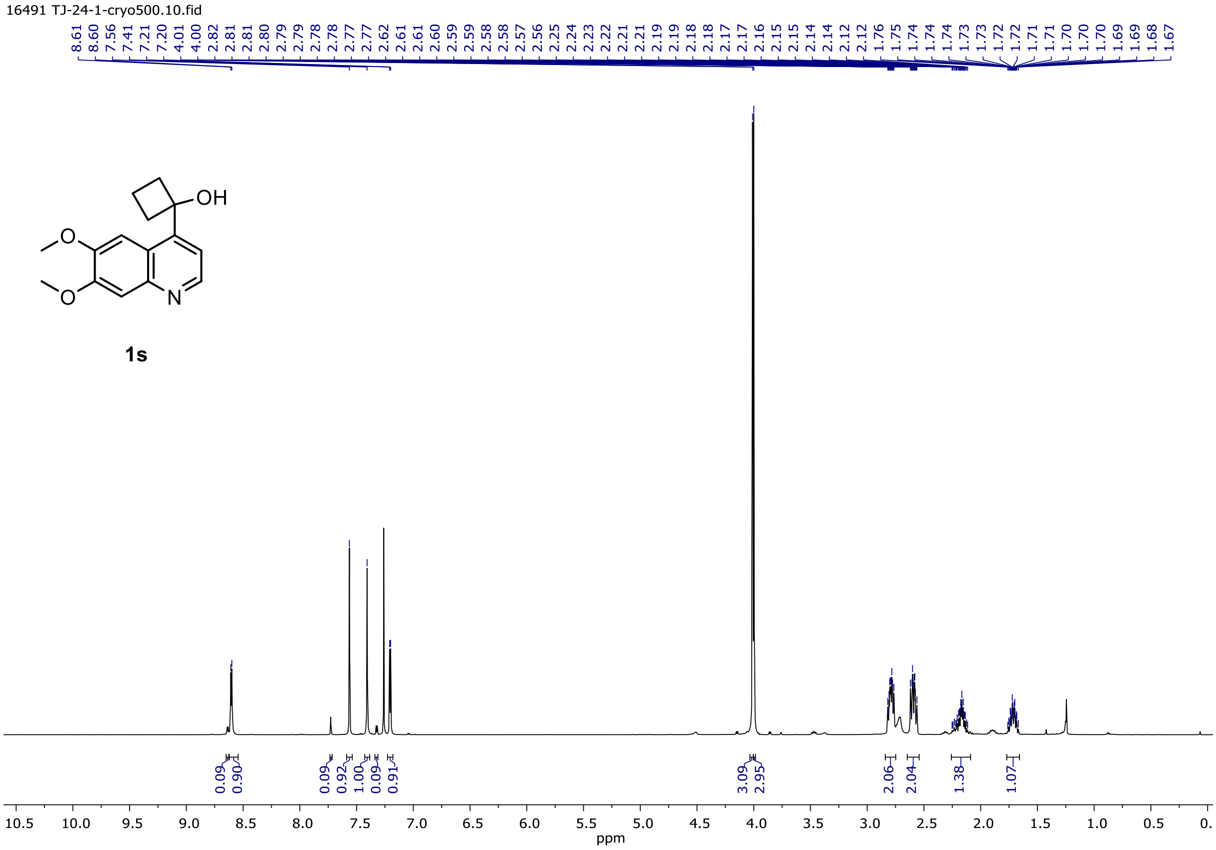

^{13}C NMR (126 MHz, $CDCl_3$) of **1s**

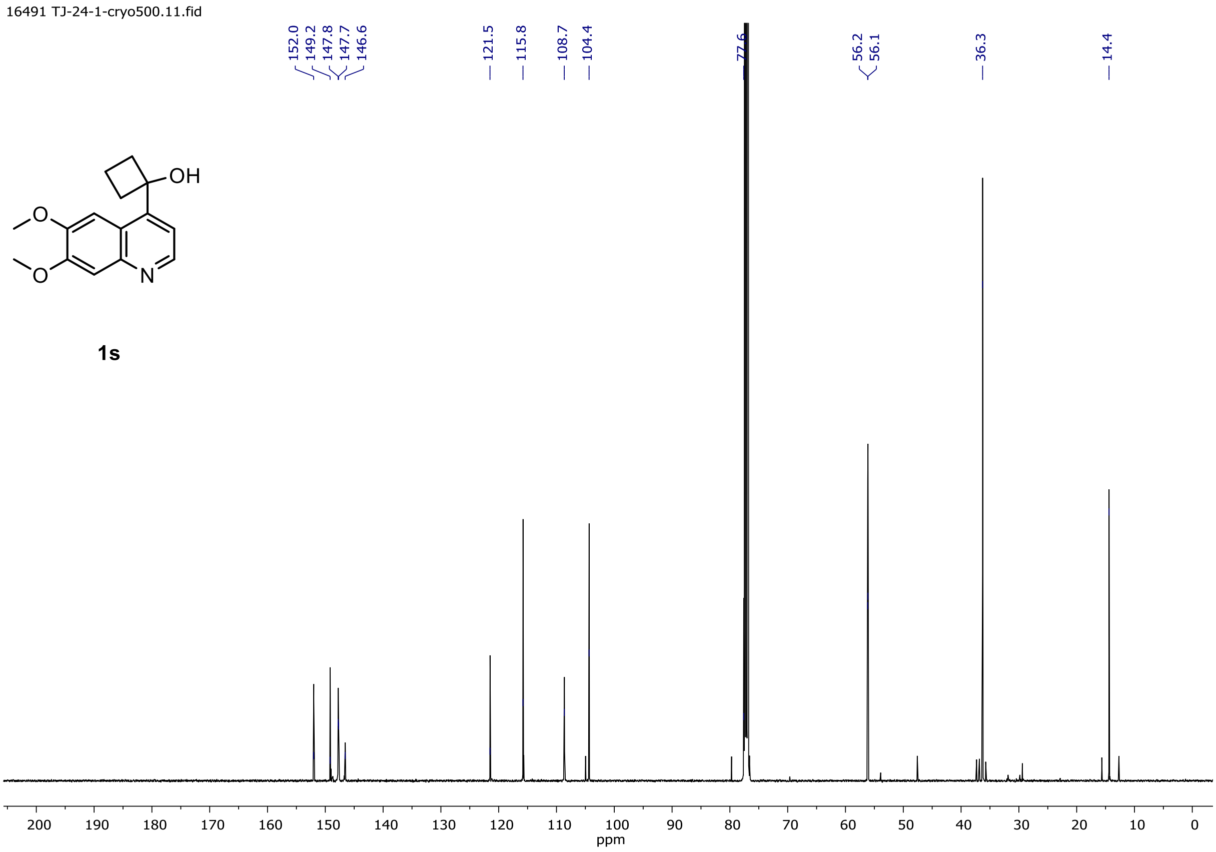

^{1}H NMR (400 MHz, CDCl$_3$) of **1t** (see procedure)

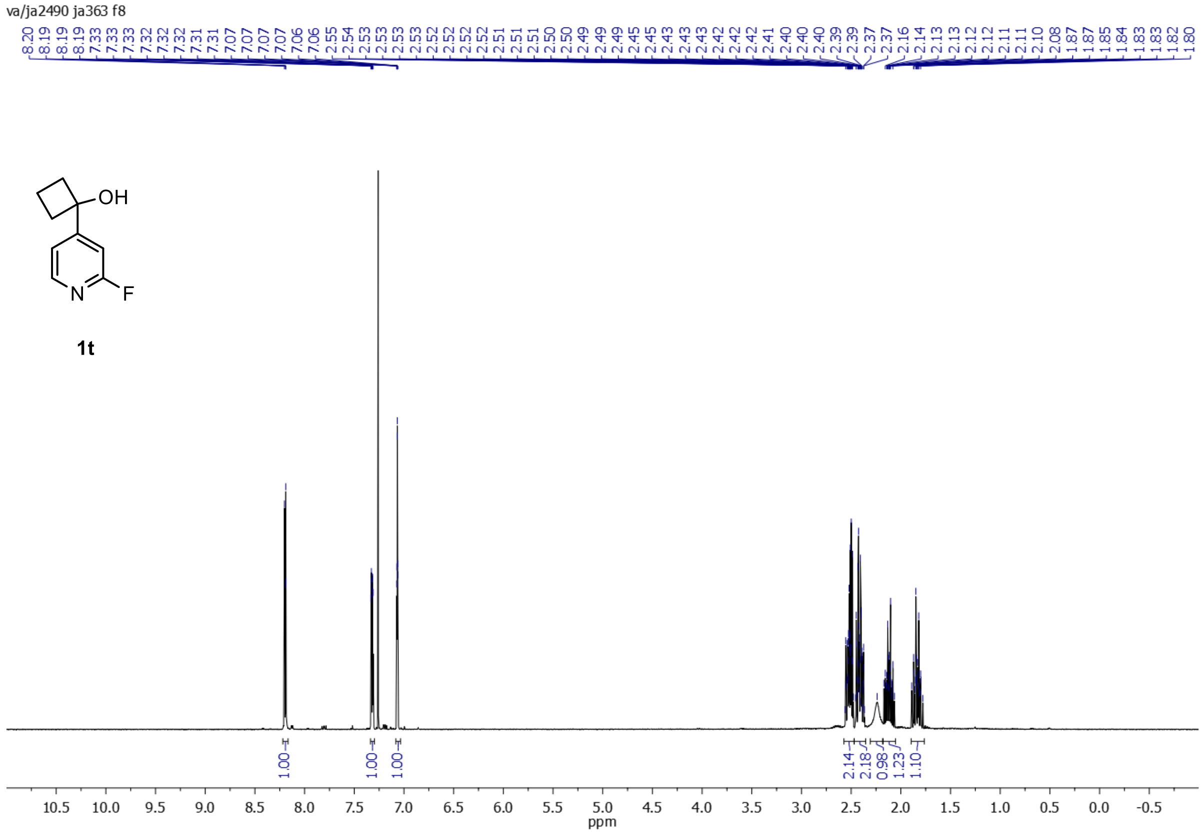

^{13}C NMR (101 MHz, CDCl$_3$) of **1t**

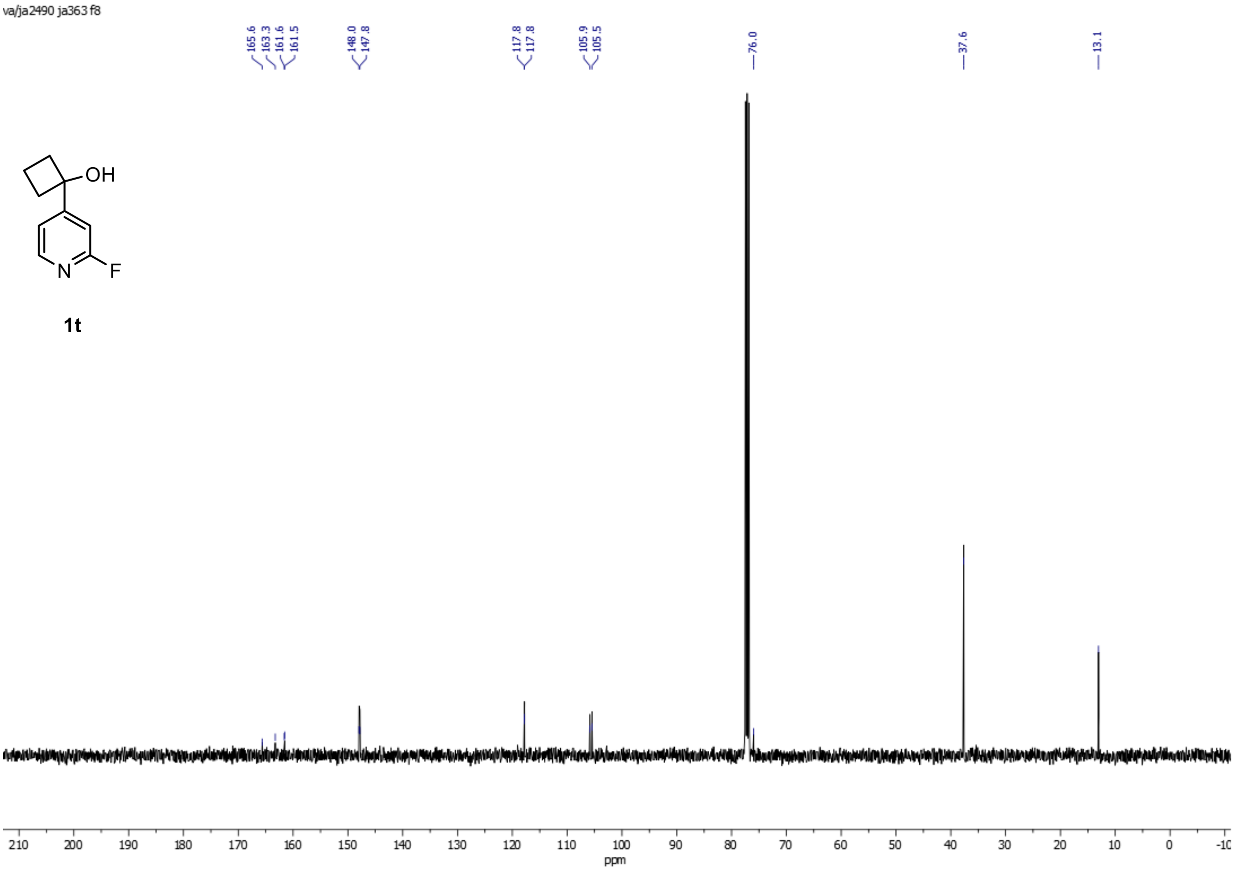

^{1}H NMR (400 MHz, $CDCl_3$) of **2a** (see procedure)

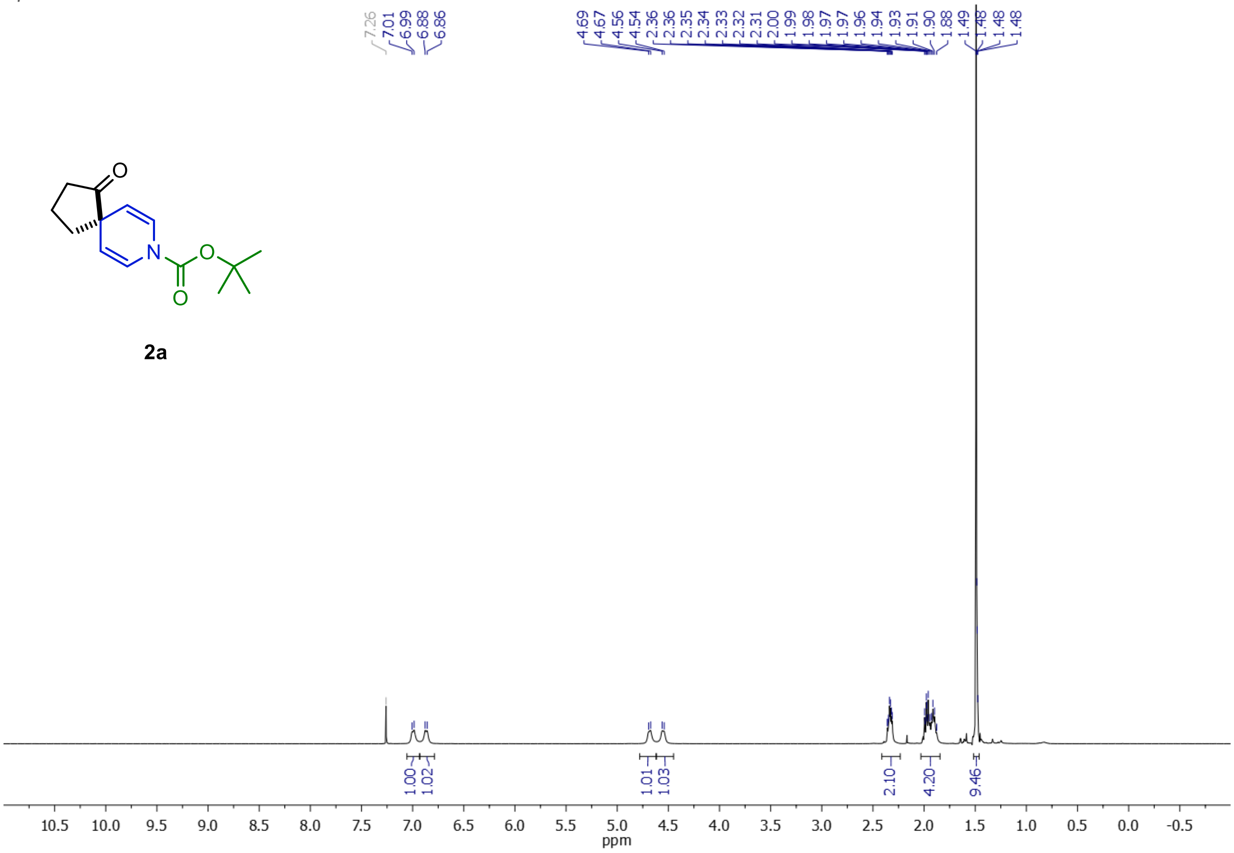

^{13}C NMR (101 MHz, $CDCl_3$) of **2a**

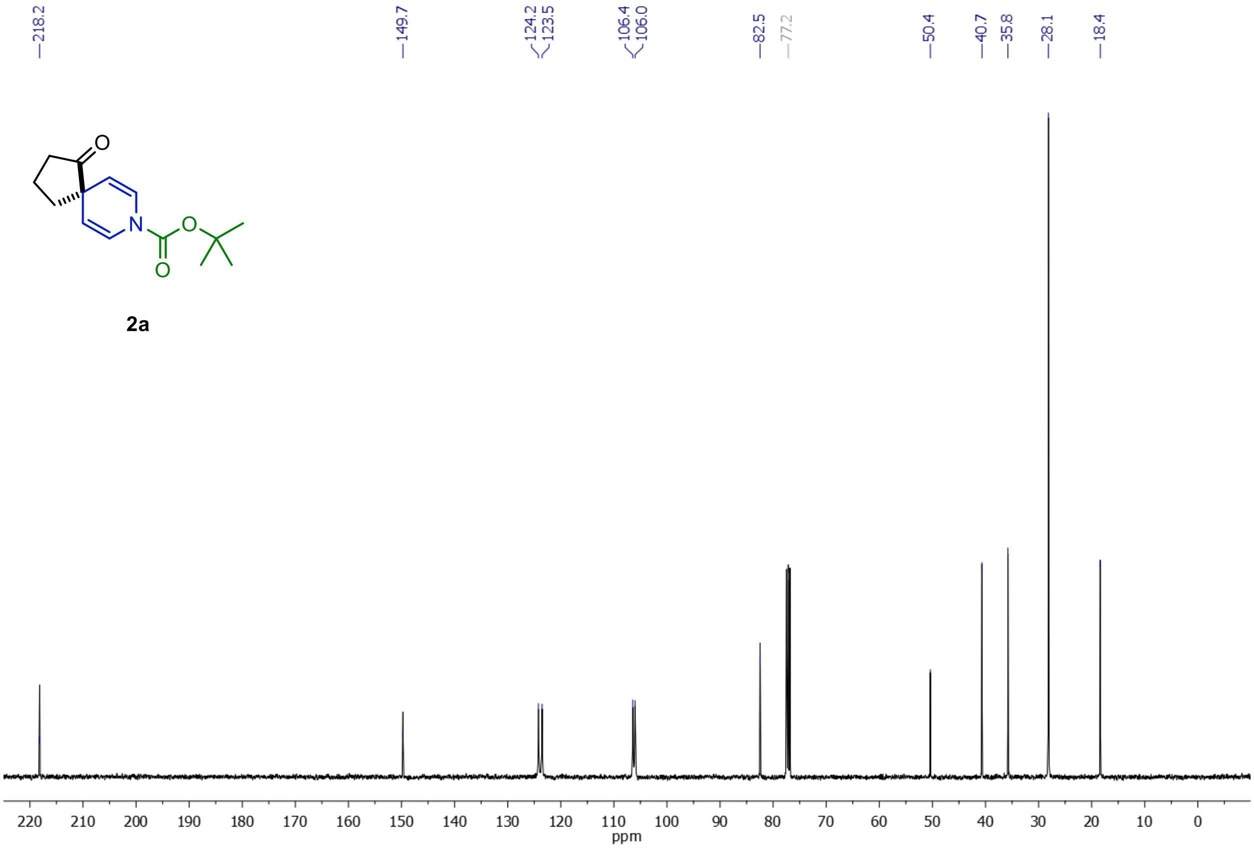

^{1}H NMR (400 MHz, $CDCl_3$) of **2b** (see procedure)

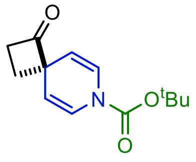

2b

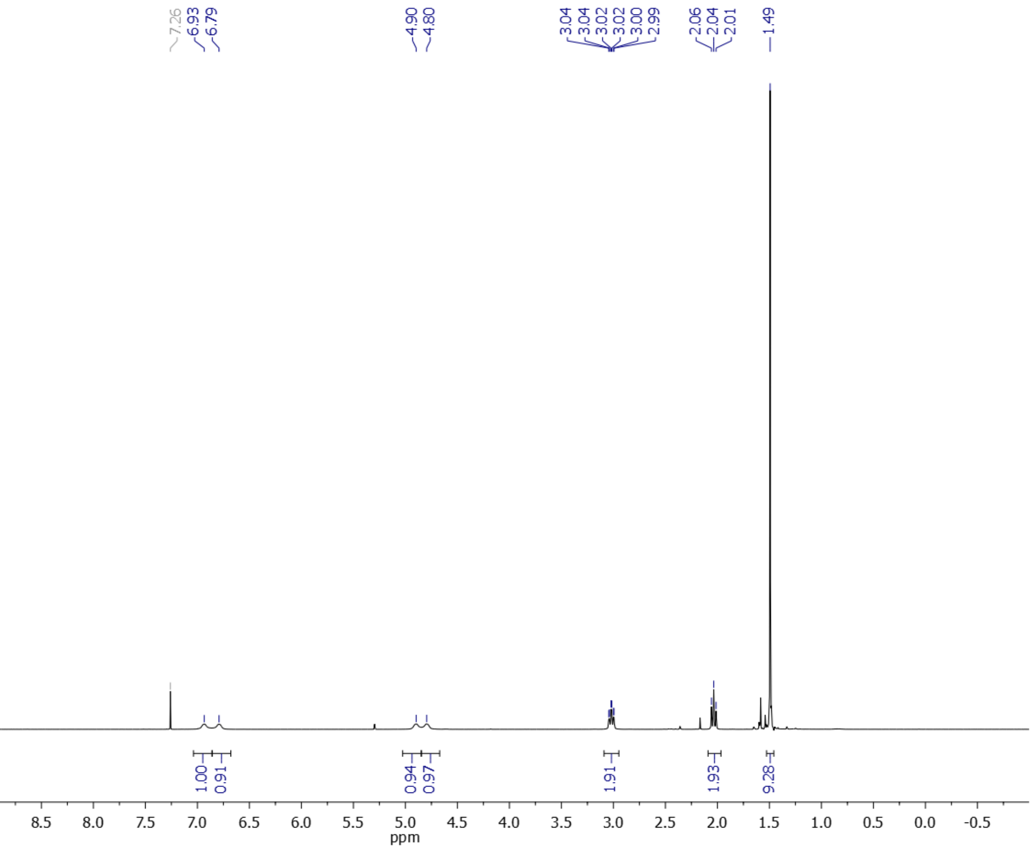

^{13}C NMR (101 MHz, $CDCl_3$) of **2b**

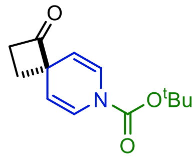

2b

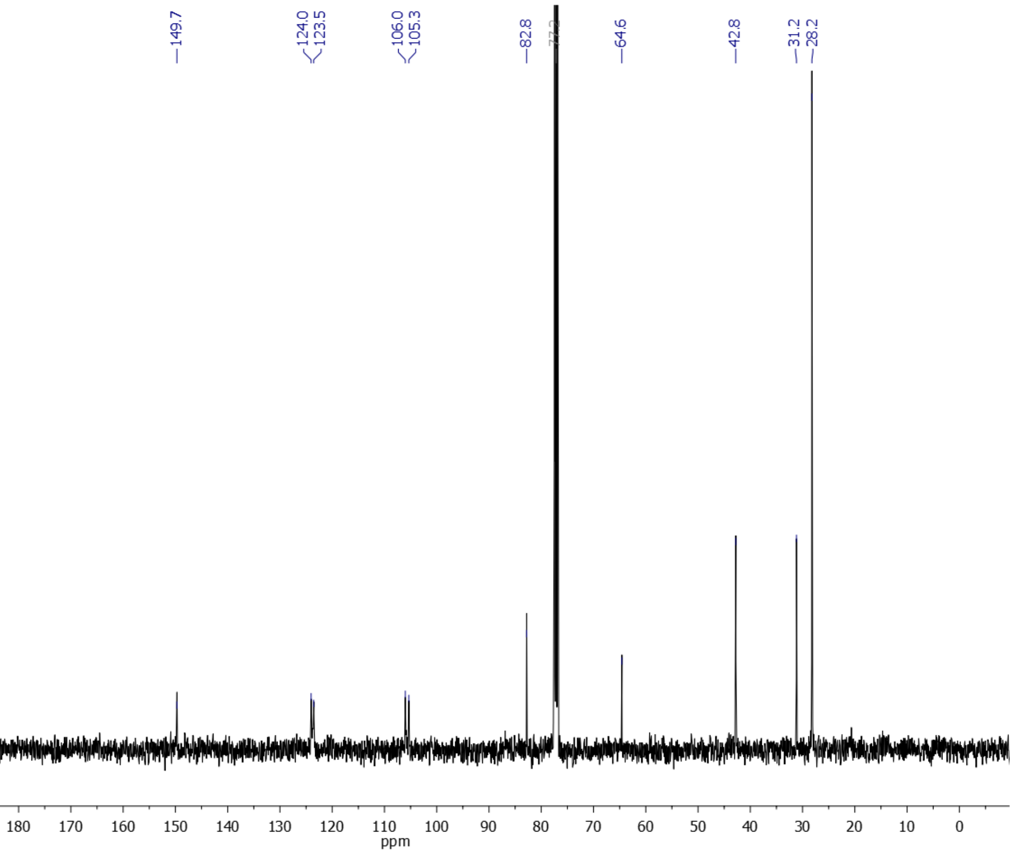

^{1}H NMR (500 MHz, $CDCl_3$) of **2c** (see procedure)

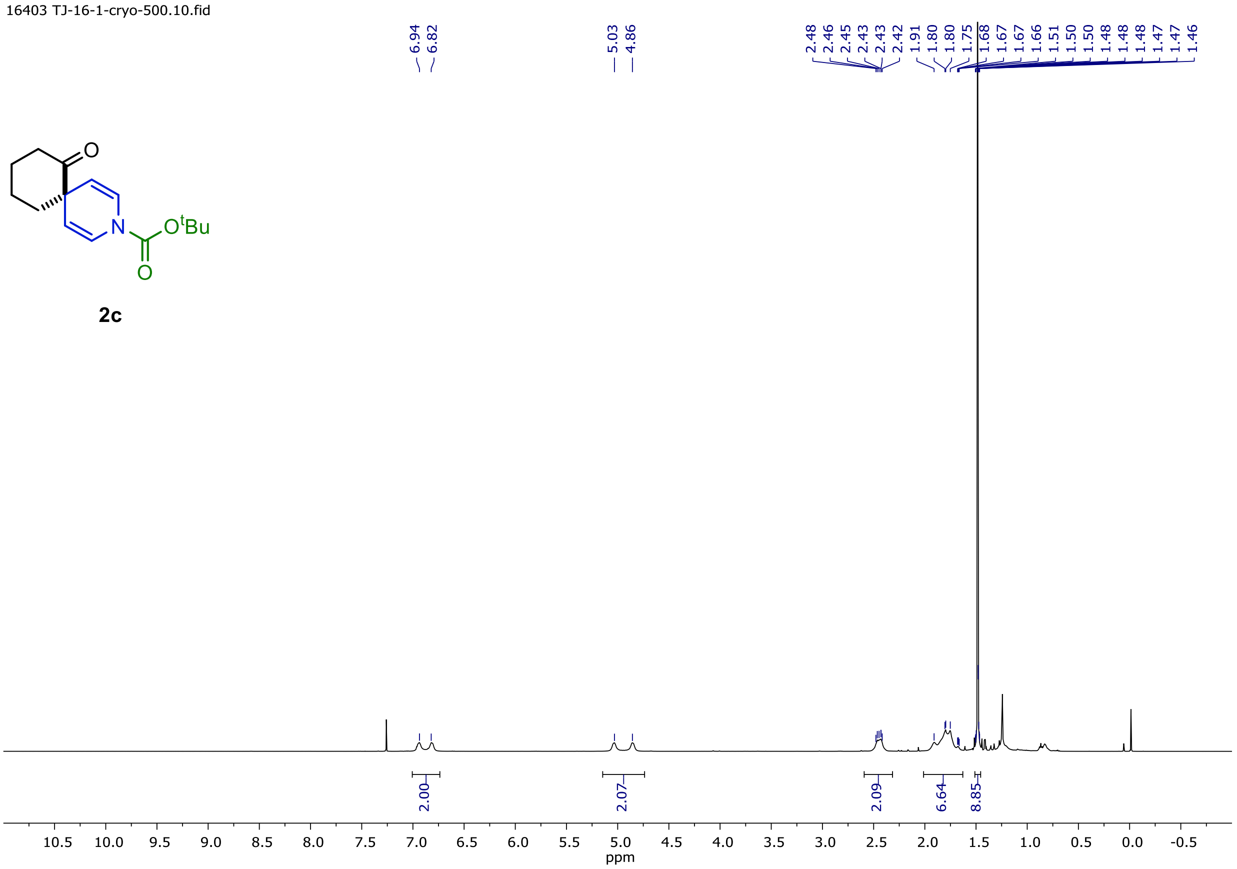

2c

^{13}C NMR (126 MHz, $CDCl_3$) of **2c**

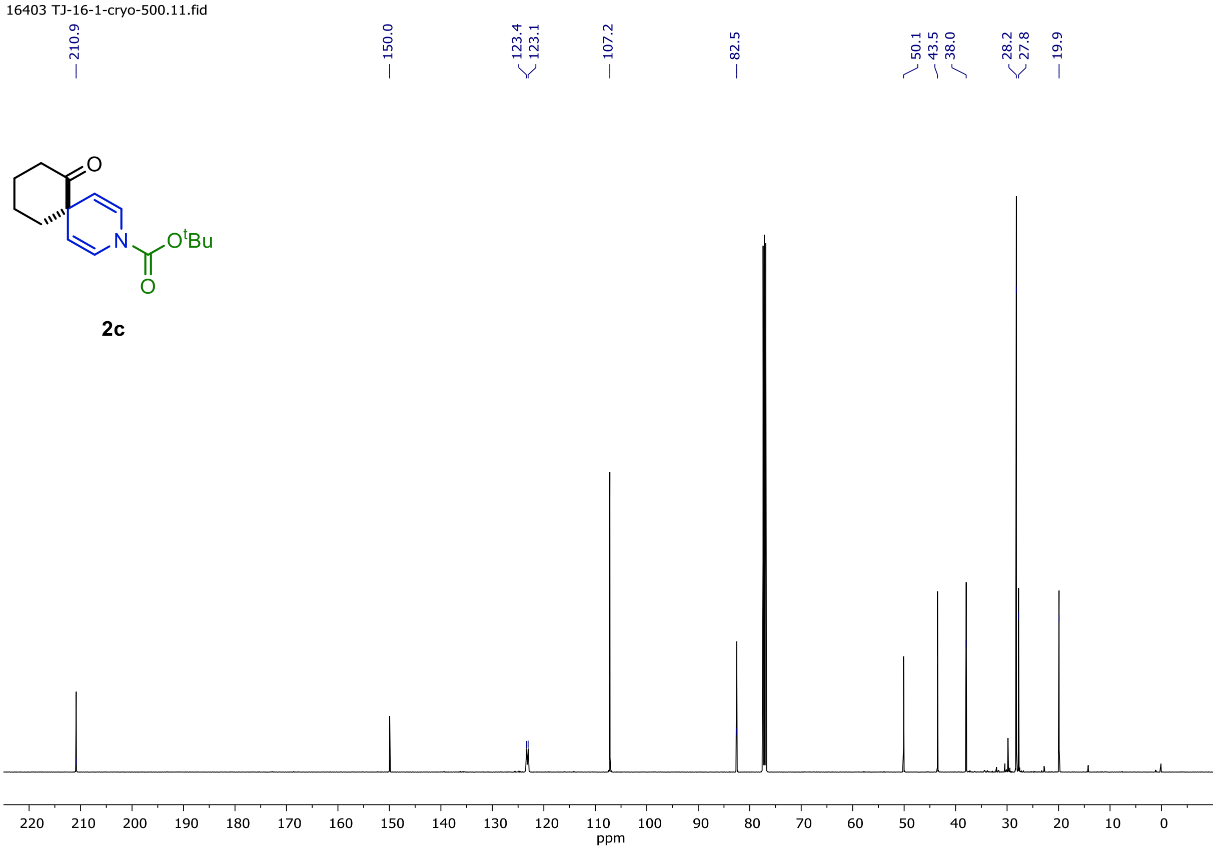

2c

^{1}H NMR (400 MHz, $CDCl_3$) of **2e** (see procedure)

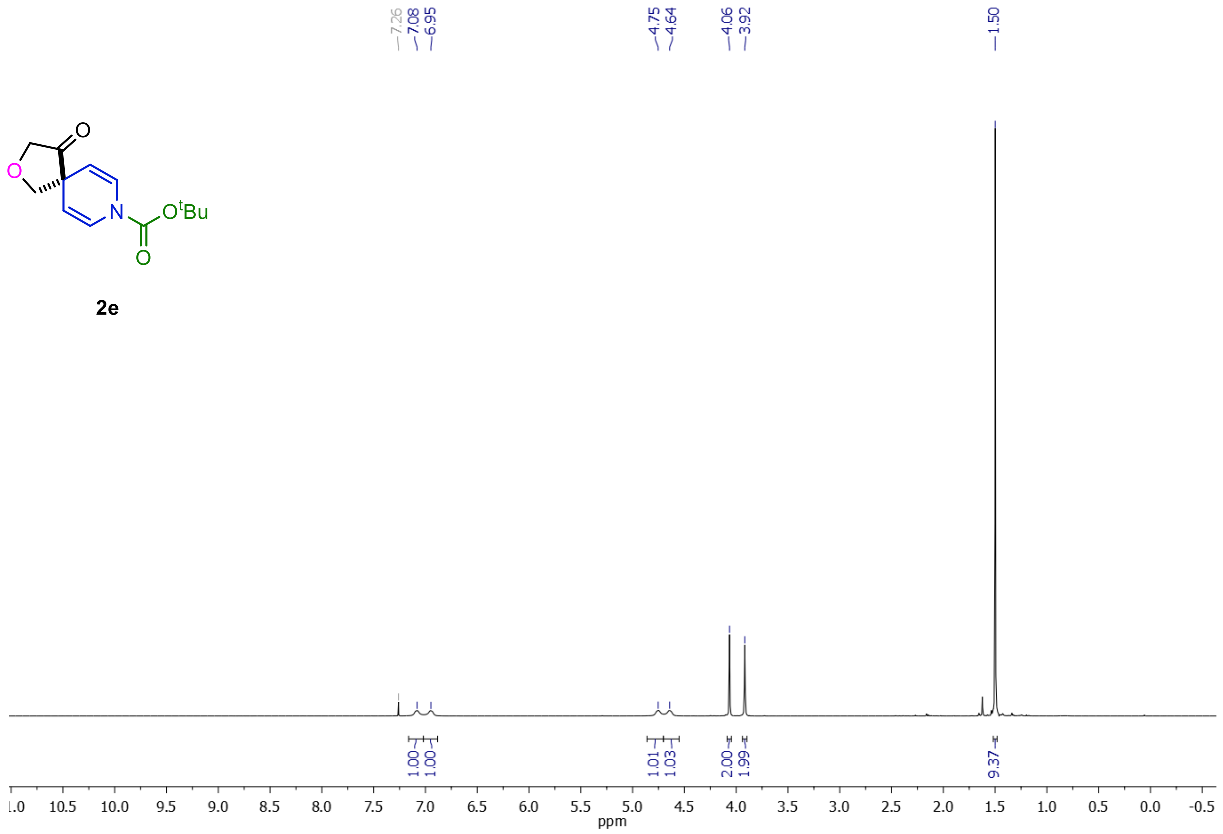

^{13}C NMR (101 MHz, $CDCl_3$) of **2e**

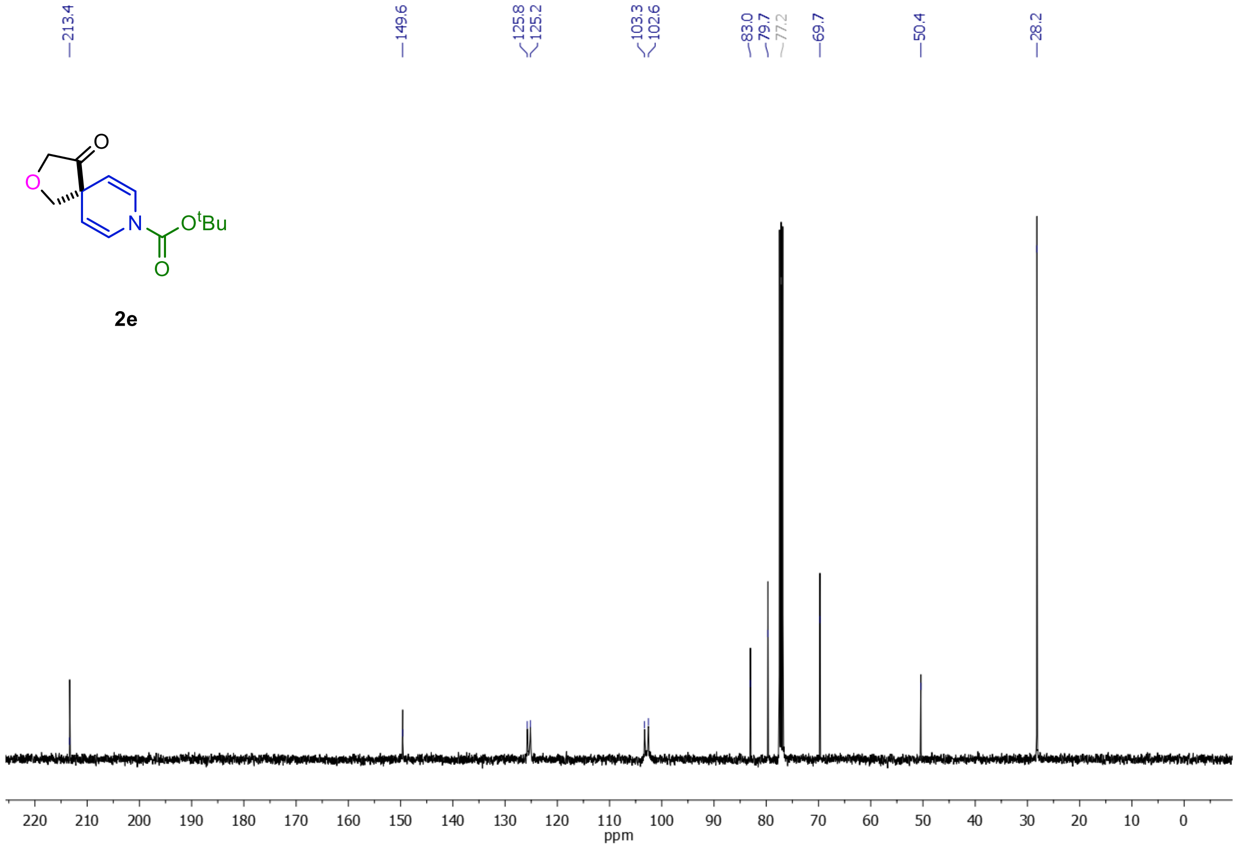

^{1}H NMR (400 MHz, $CDCl_3$) of **2f** (see procedure)

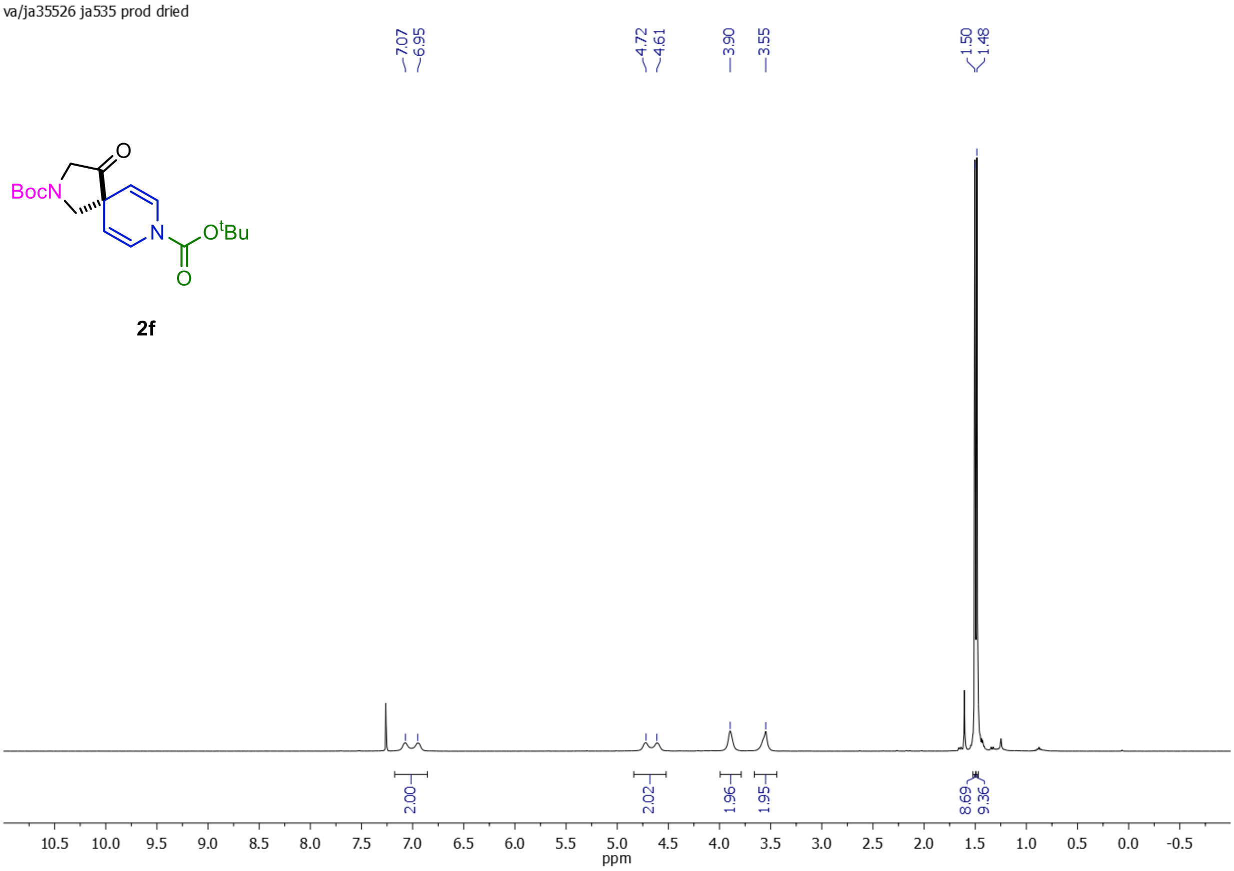

^{13}C NMR (101 MHz, $CDCl_3$) of **2f**

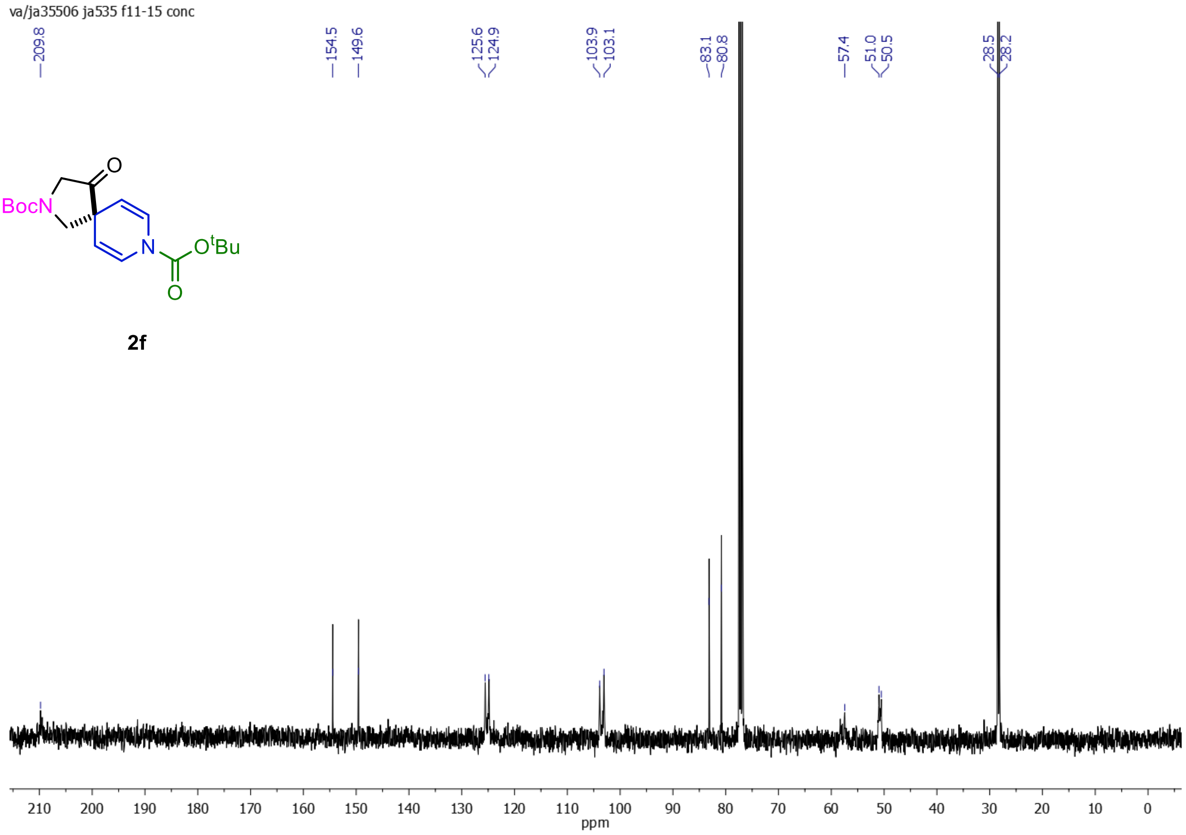

^{1}H NMR (400 MHz, $CDCl_3$) of **2g** (see procedure)

84191 ja515 f14-16.10.fid

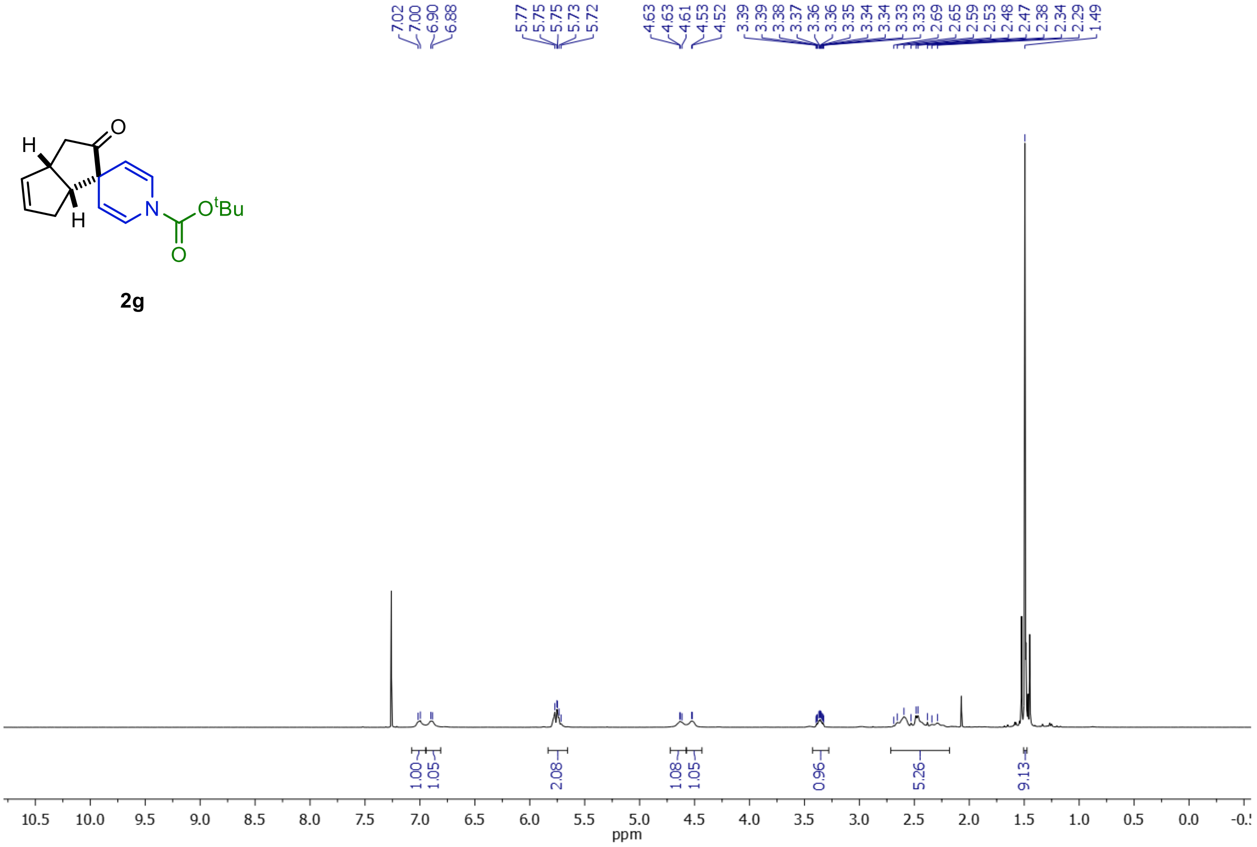

^{13}C NMR (101 MHz, $CDCl_3$) of **2g**

va/ja27660 ja515 f14-15 conc

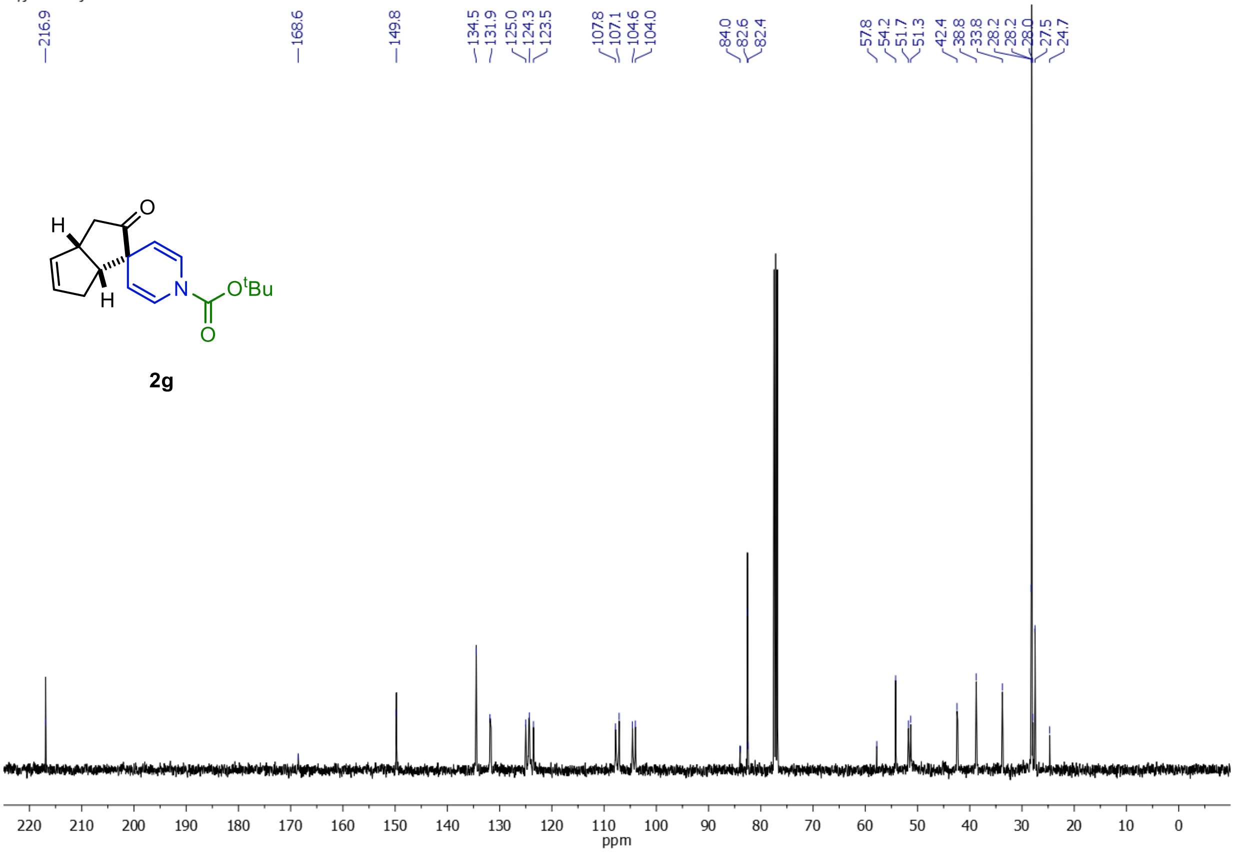

^{1}H NMR (500 MHz, CDCl$_3$) of **2h** (see procedure)

16601 NV16 cryo500.10.fid

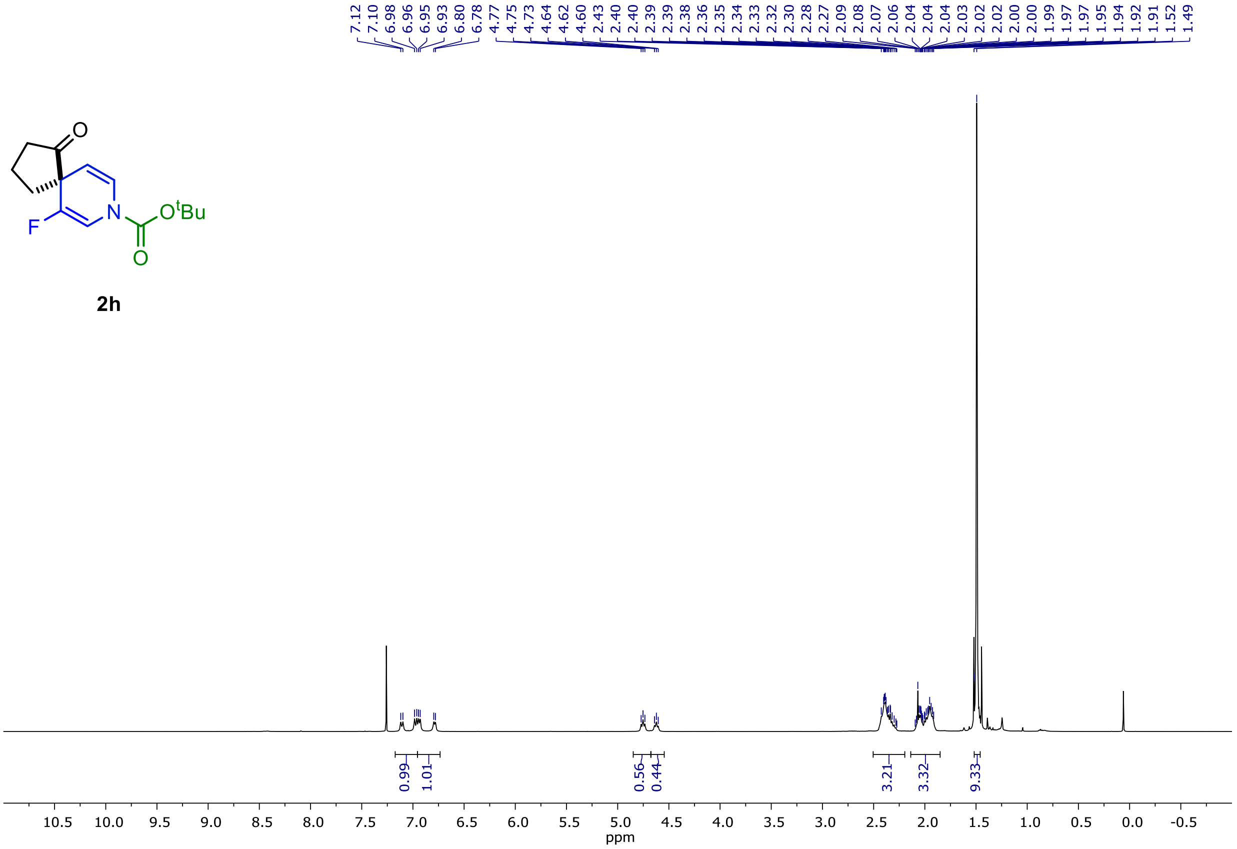

^{13}C NMR (126 MHz, CDCl$_3$) of **2h**

16601 NV16 cryo500.11.fid

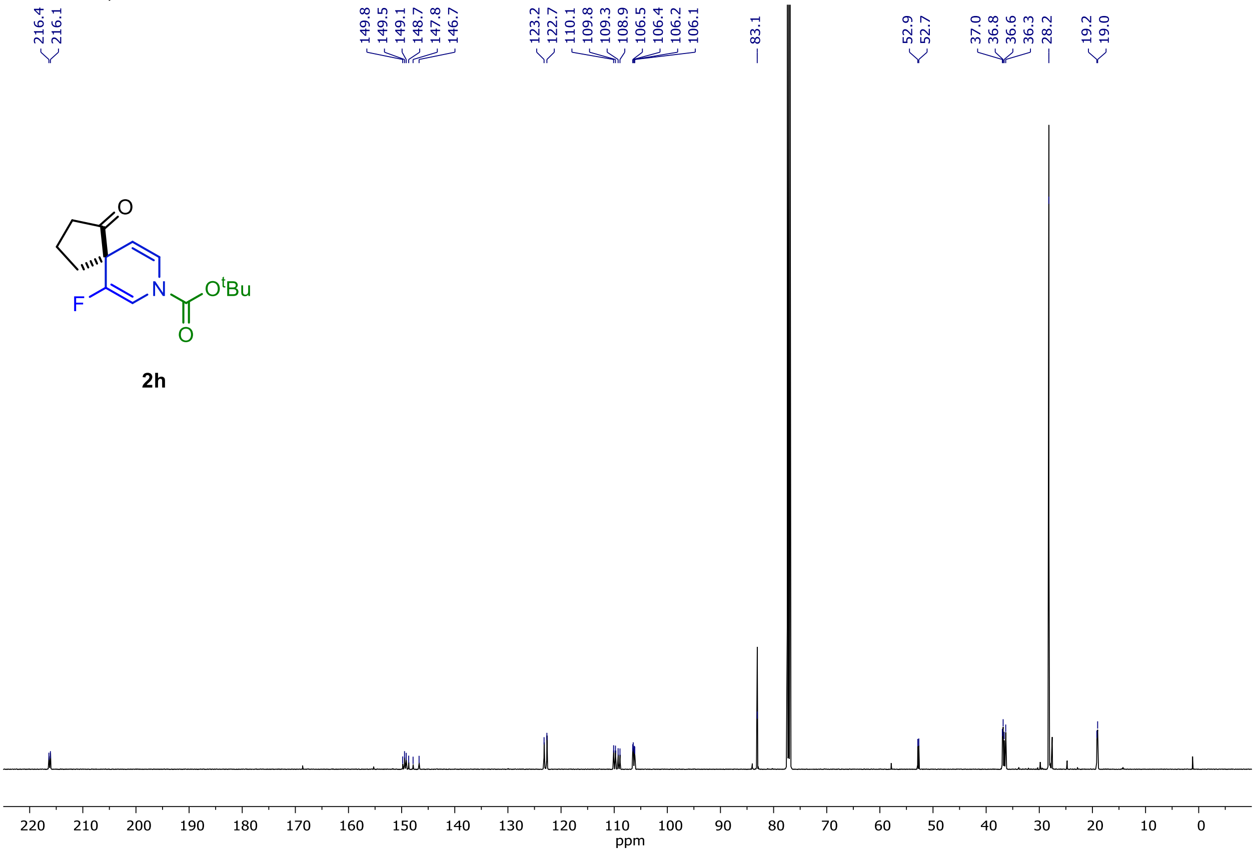

^{1}H NMR (500 MHz, $CDCl_3$) of **2i** (see procedure)

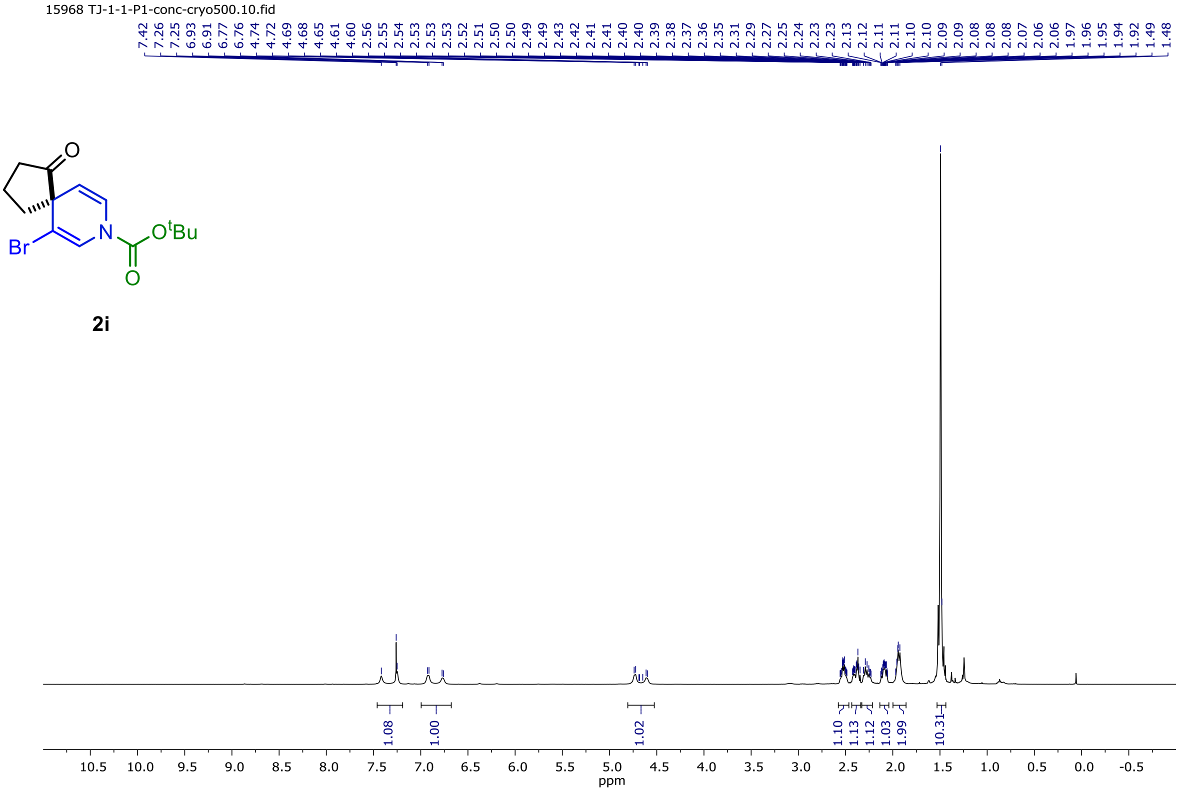

^{13}C NMR (126 MHz, $CDCl_3$) of **2i**

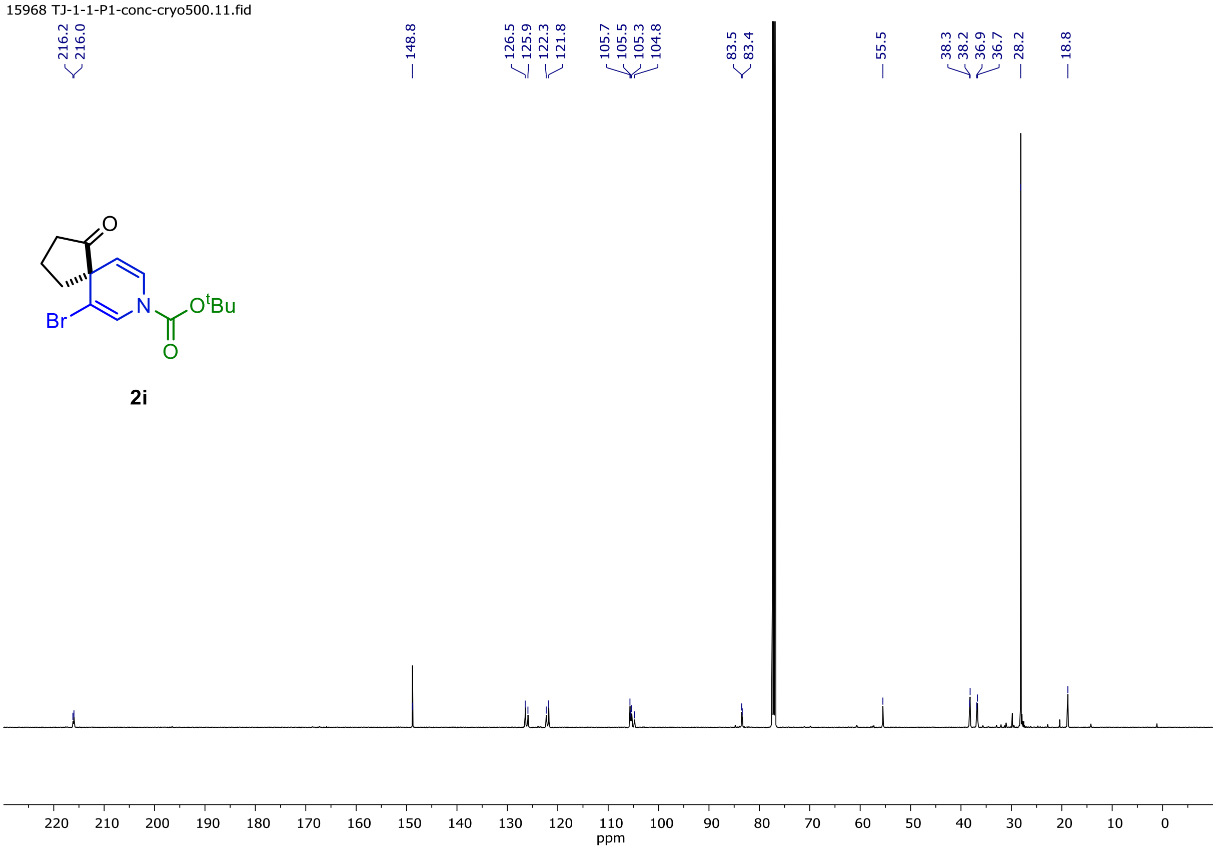

^{1}H NMR (400 MHz, $CDCl_3$) of **2j** (see procedure)

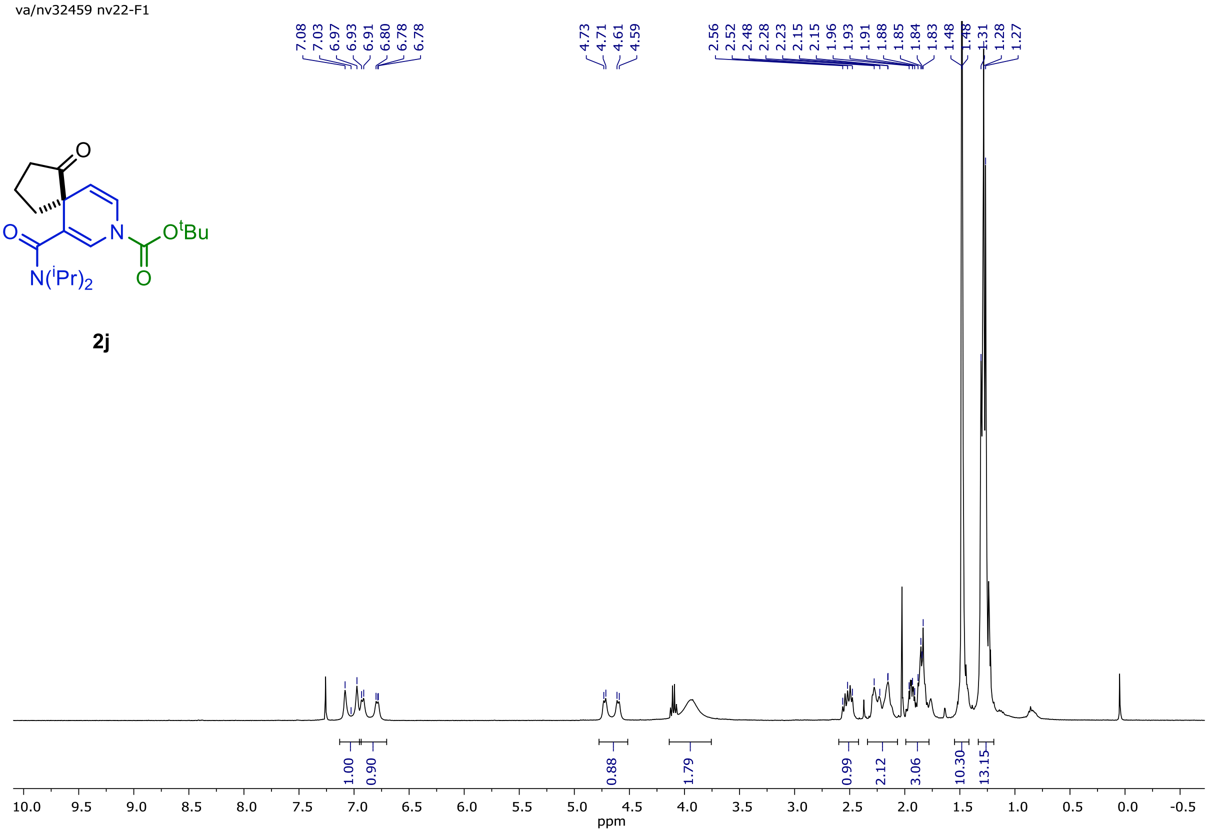

^{13}C NMR (101 MHz, $CDCl_3$) of **2j**

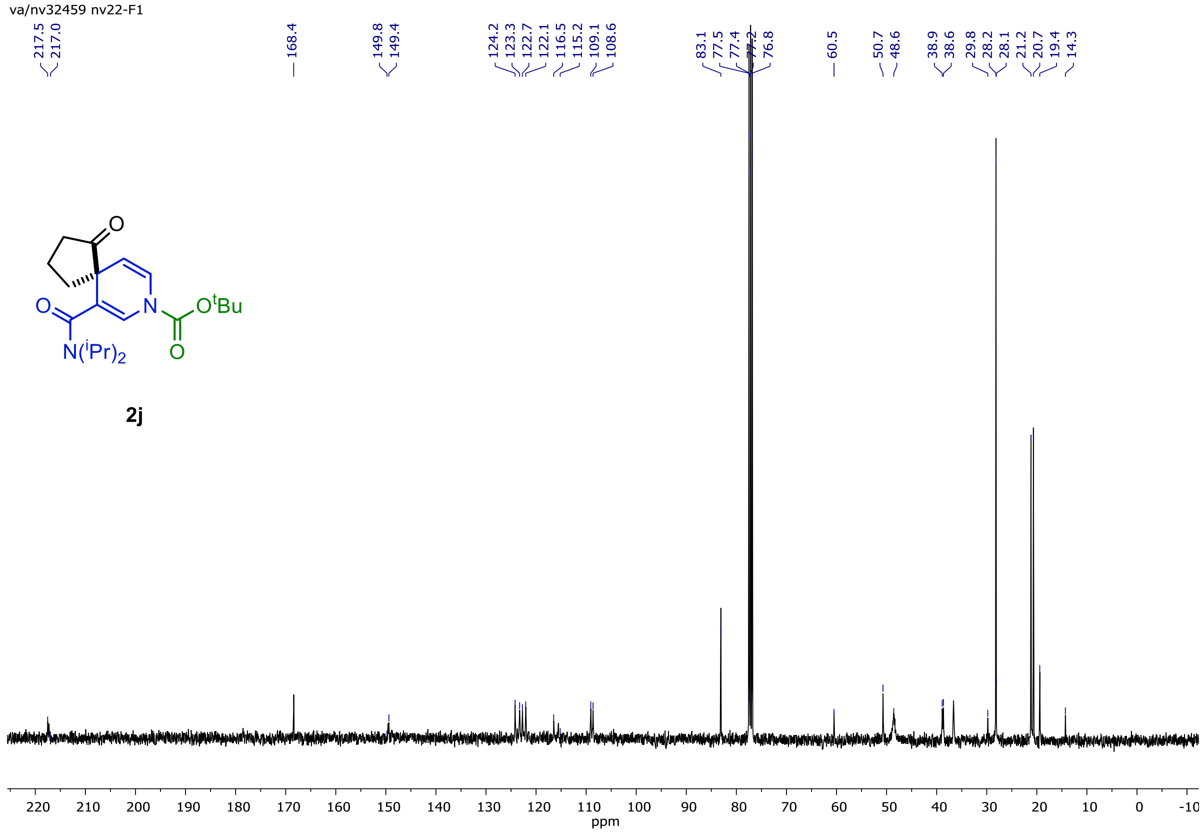

^{1}H NMR (500 MHz, $CDCl_3$) of **2k** (see procedure)

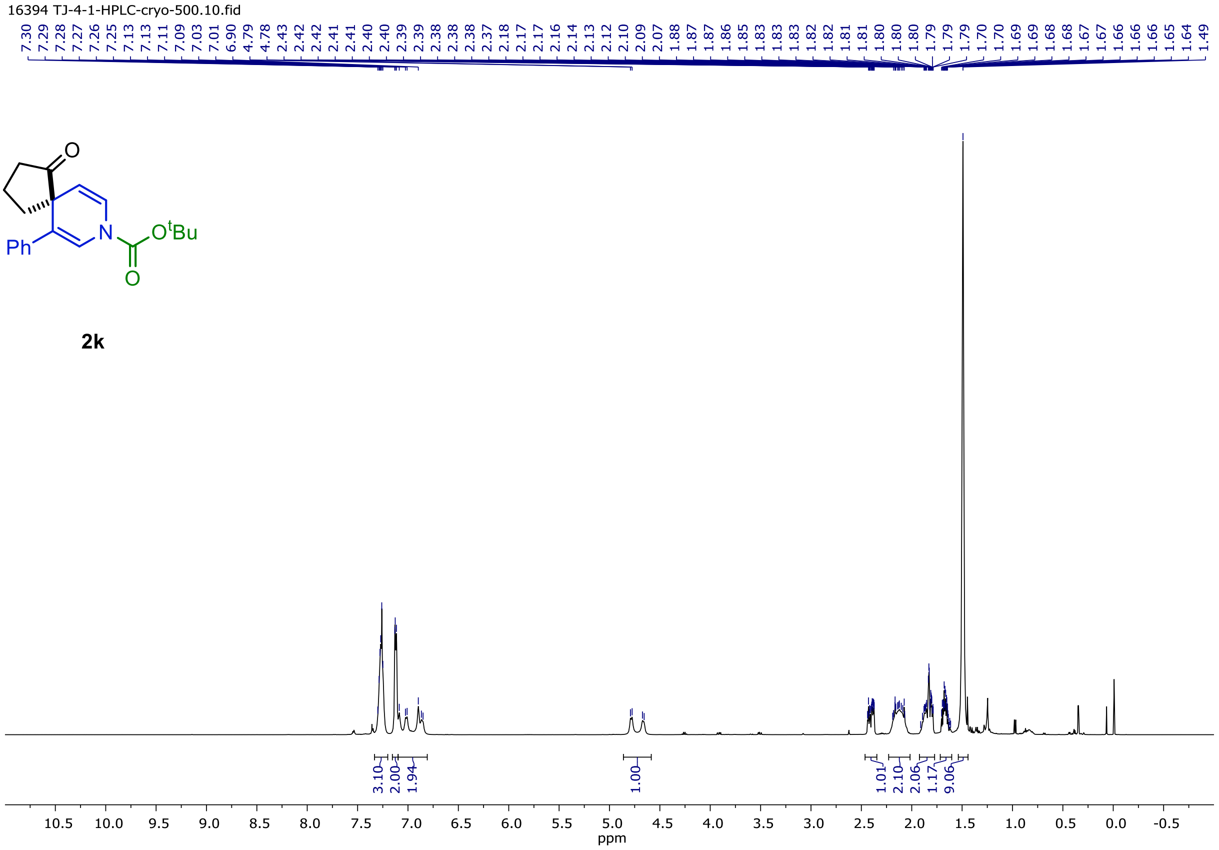

^{13}C NMR (126 MHz, $CDCl_3$) of **2k**

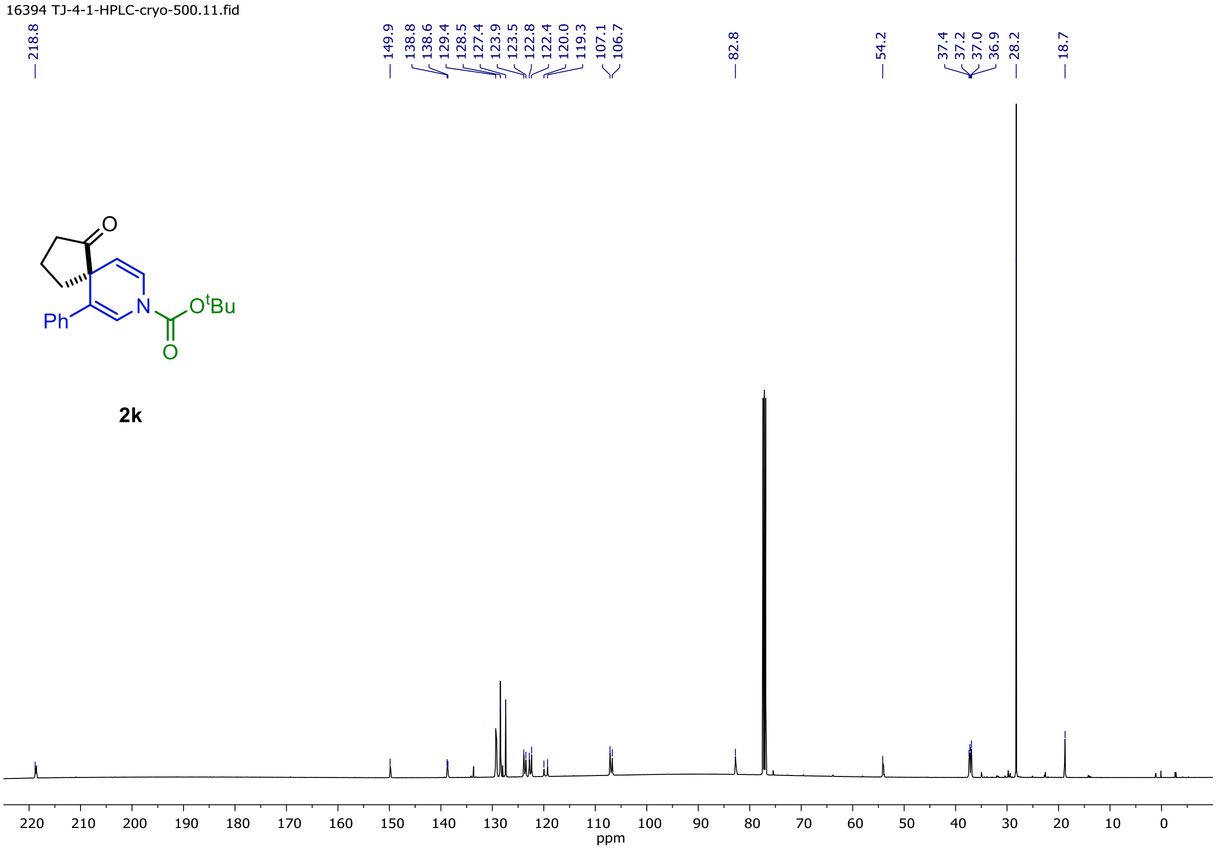

^{1}H NMR (400 MHz, $CDCl_3$) of **2l** (see procedure)

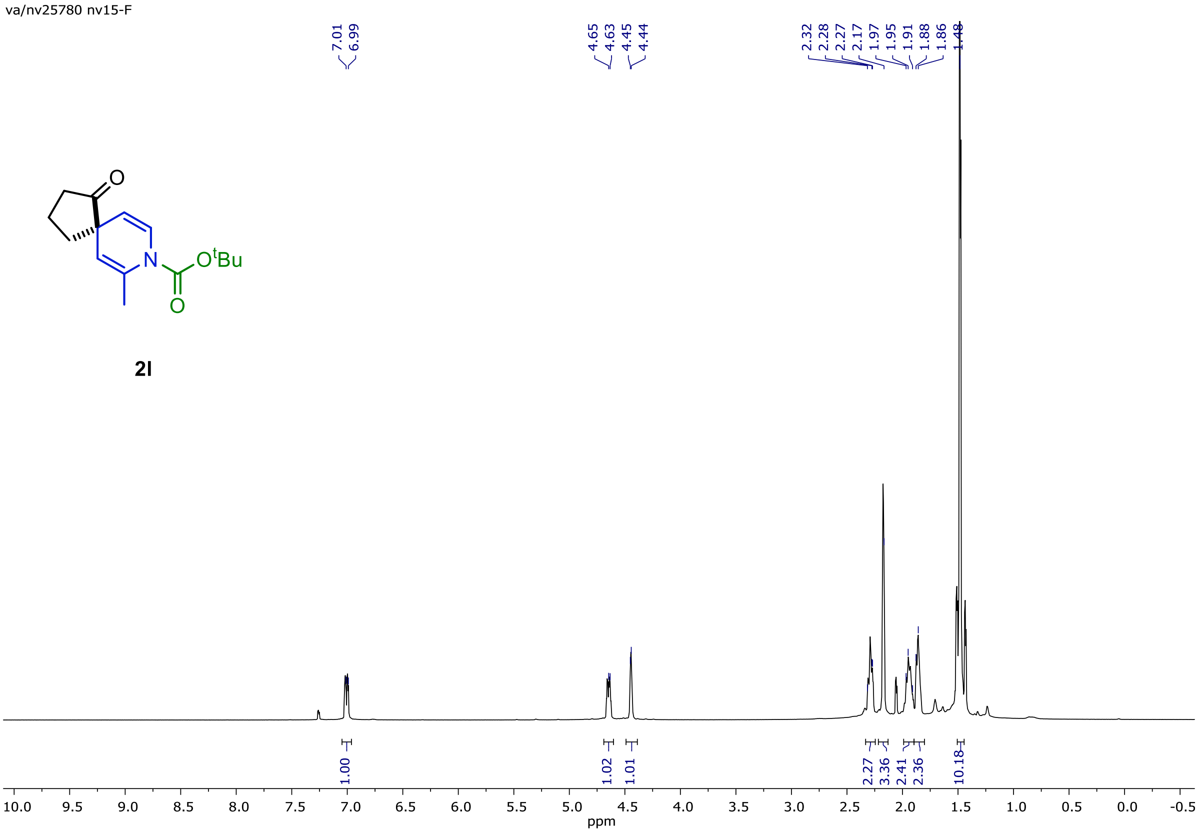

^{13}C NMR (101 MHz, $CDCl_3$) of **2l**

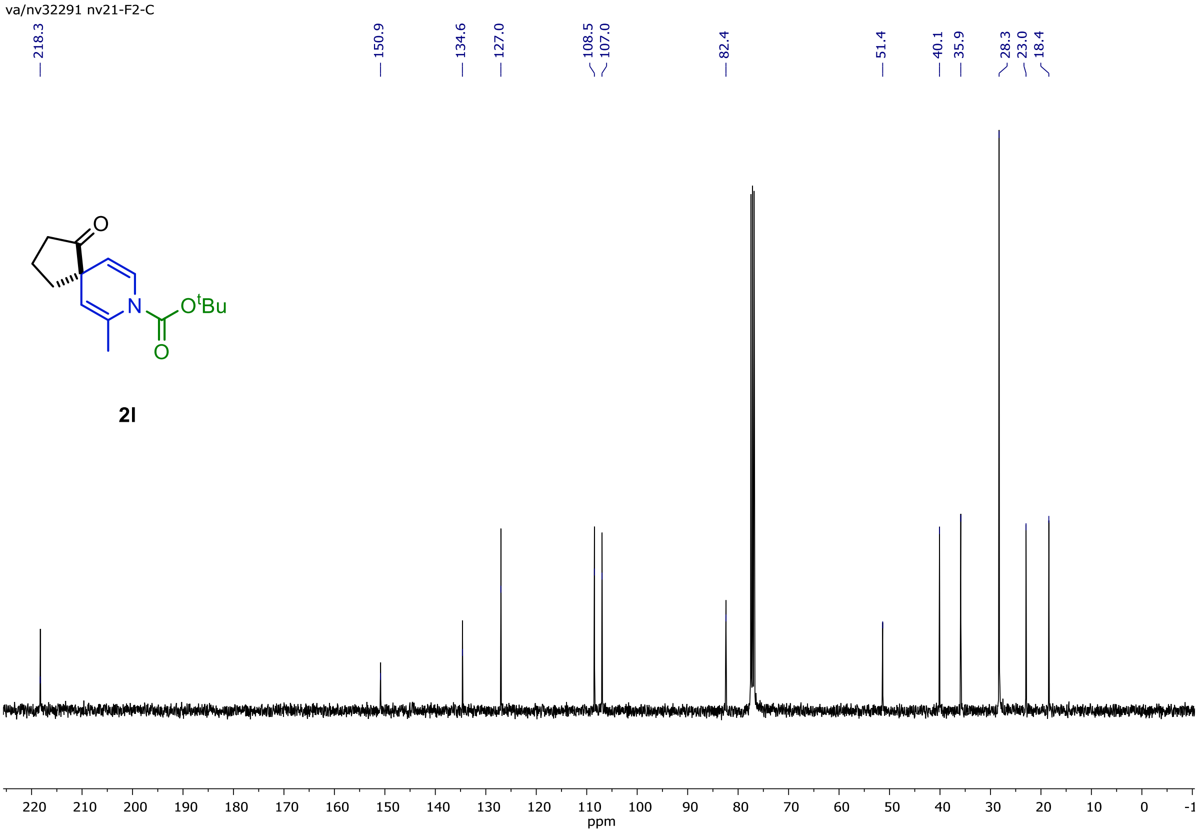

^{1}H NMR (400 MHz, $CDCl_3$) of **2m** (see procedure)

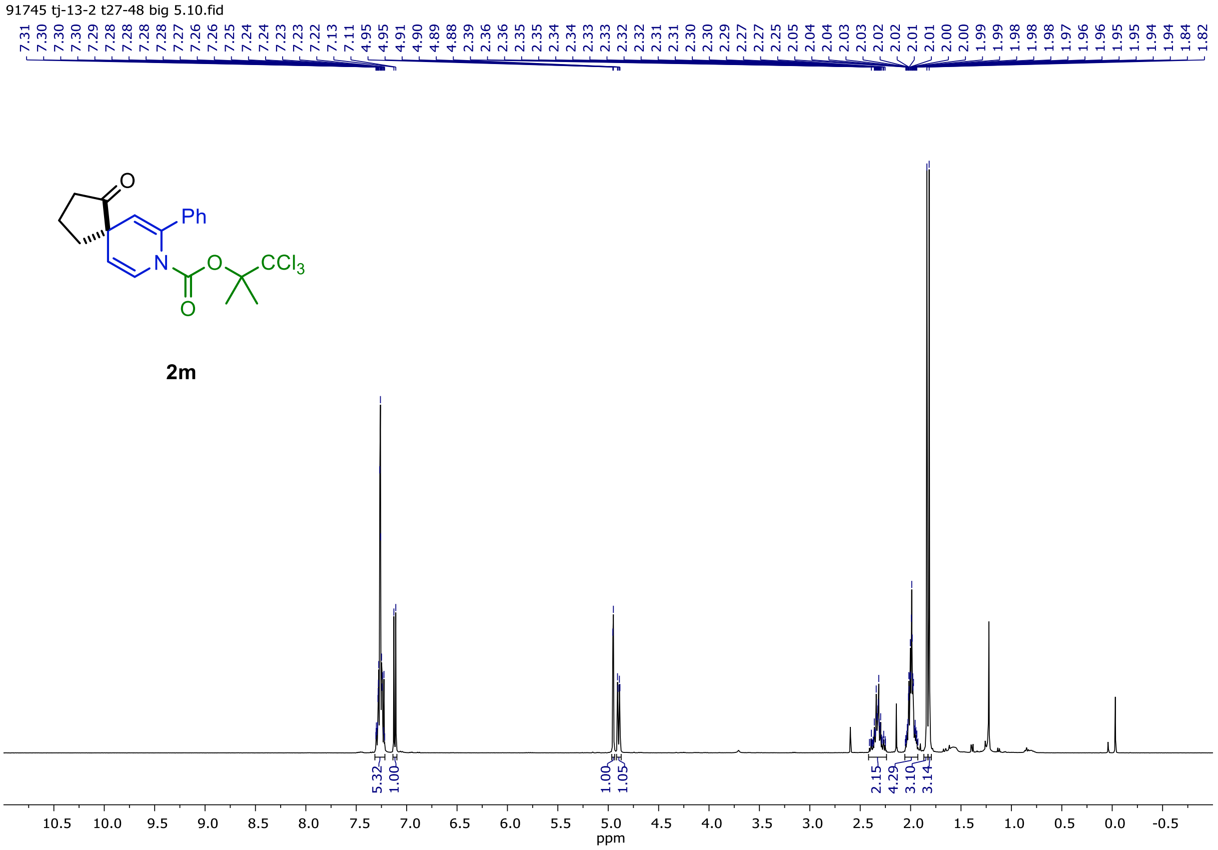

^{13}C NMR (101 MHz, $CDCl_3$) of **2m**

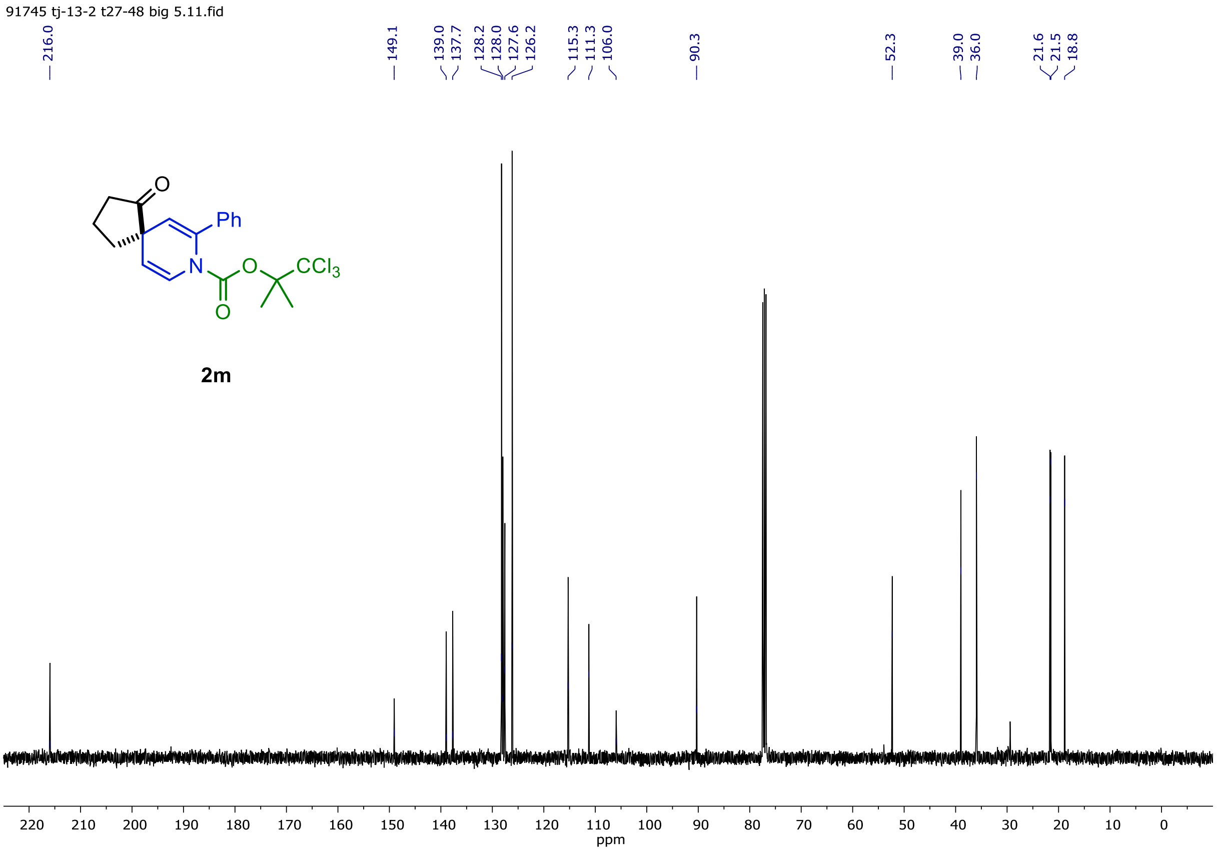

^{1}H NMR (400 MHz, $CDCl_3$) of **2n** (see procedure)

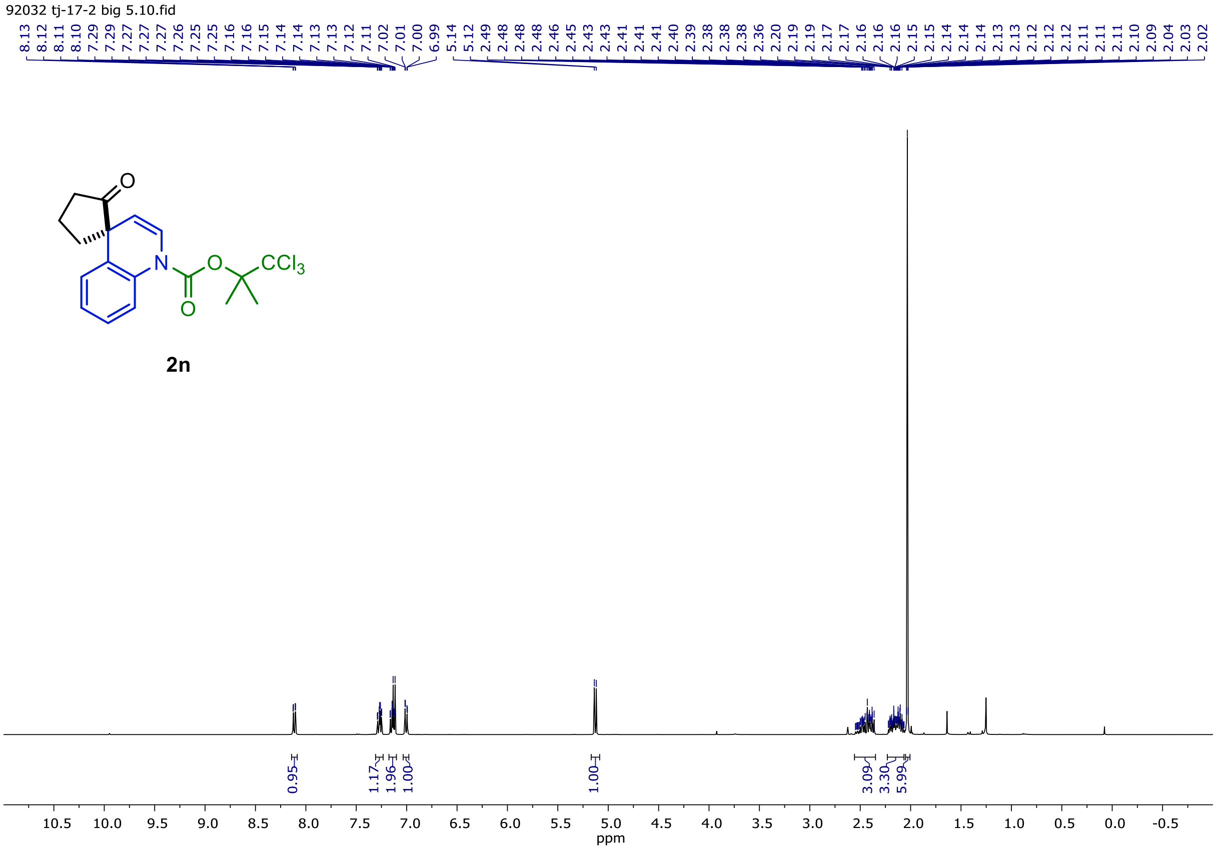

^{13}C NMR (101 MHz, $CDCl_3$) of **2n**

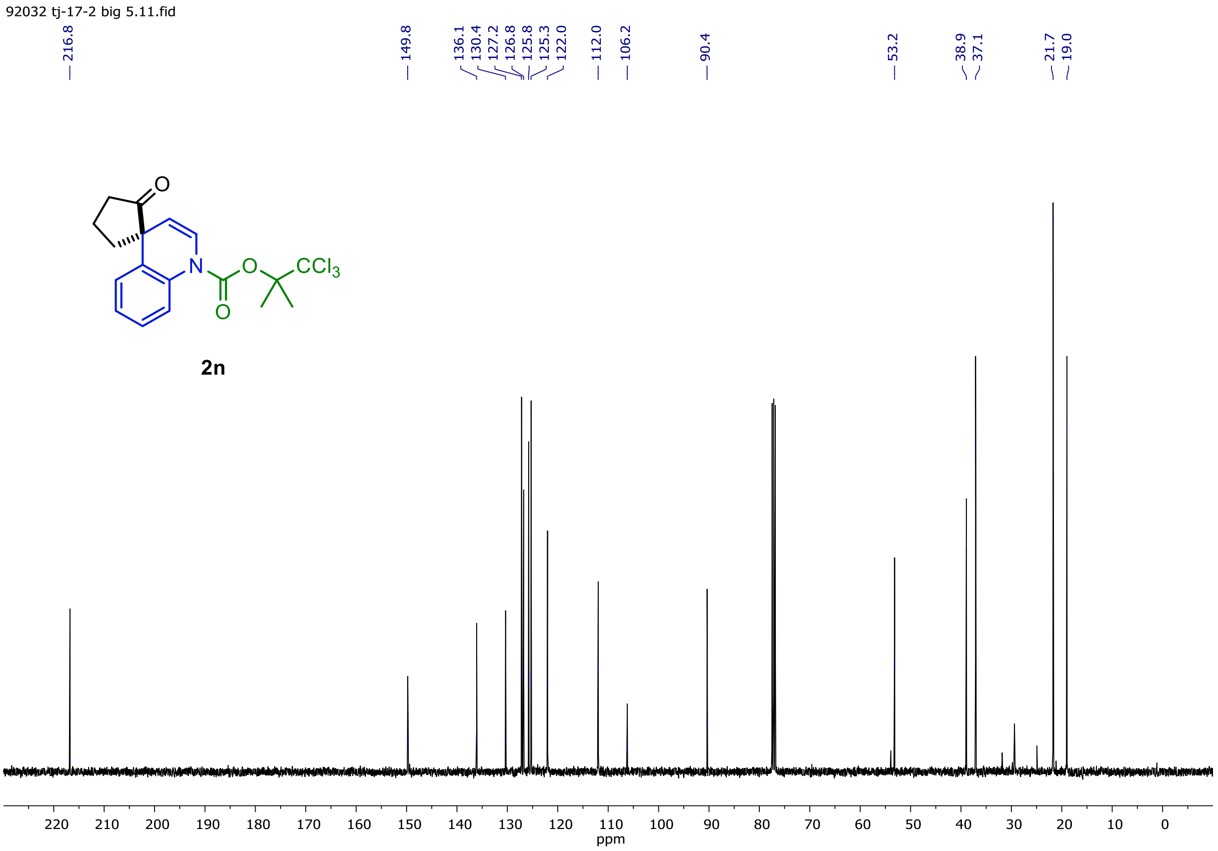

^{1}H NMR (500 MHz, $CDCl_3$) of **2o** (see procedure)

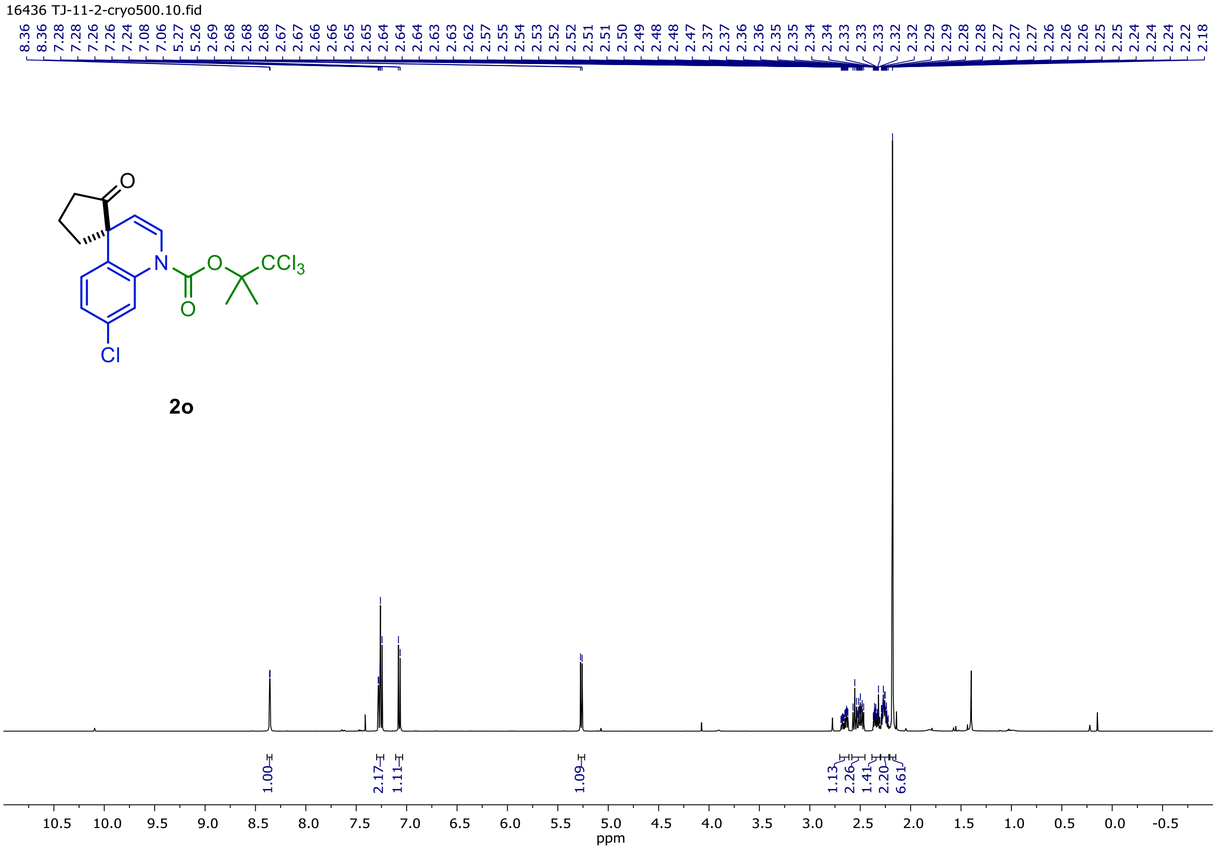

^{13}C NMR (126 MHz, $CDCl_3$) of **2o**

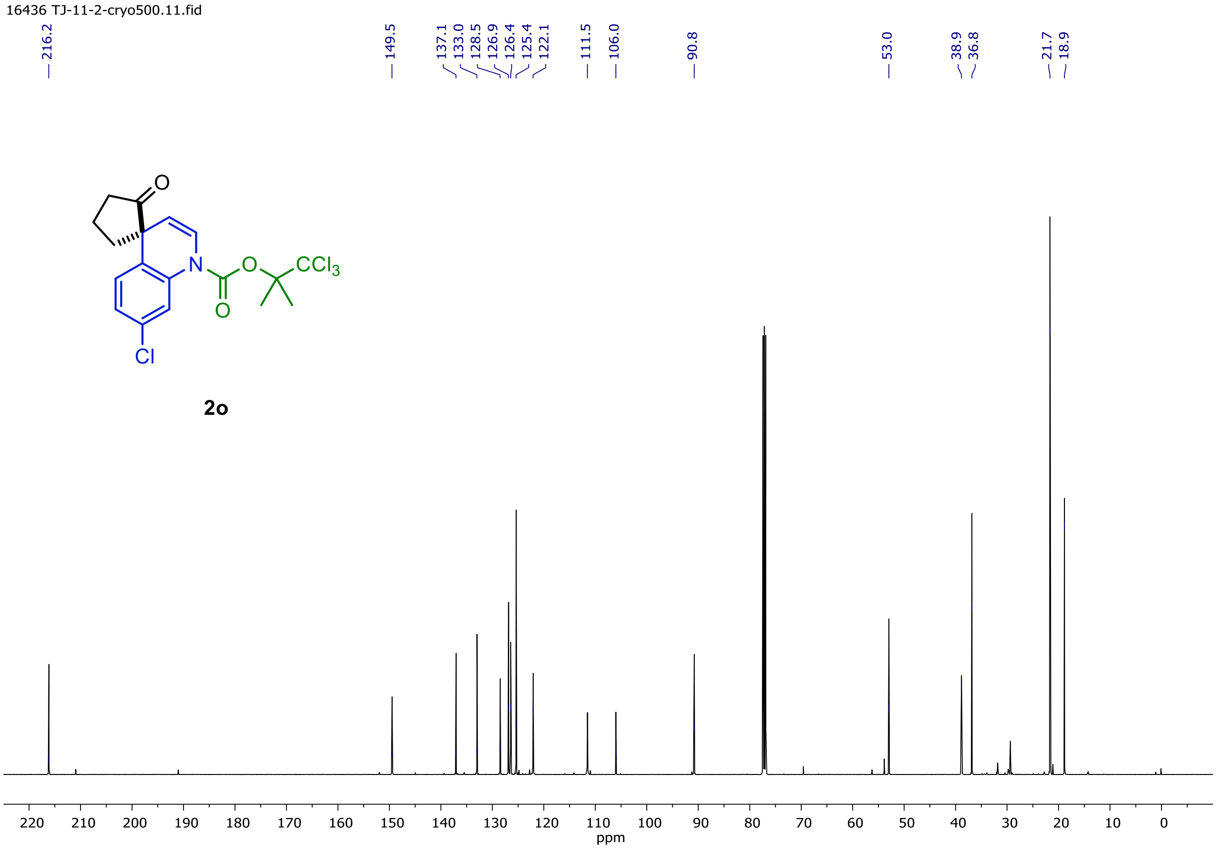

^{1}H NMR (400 MHz, $CDCl_3$) of **2p** (see procedure)

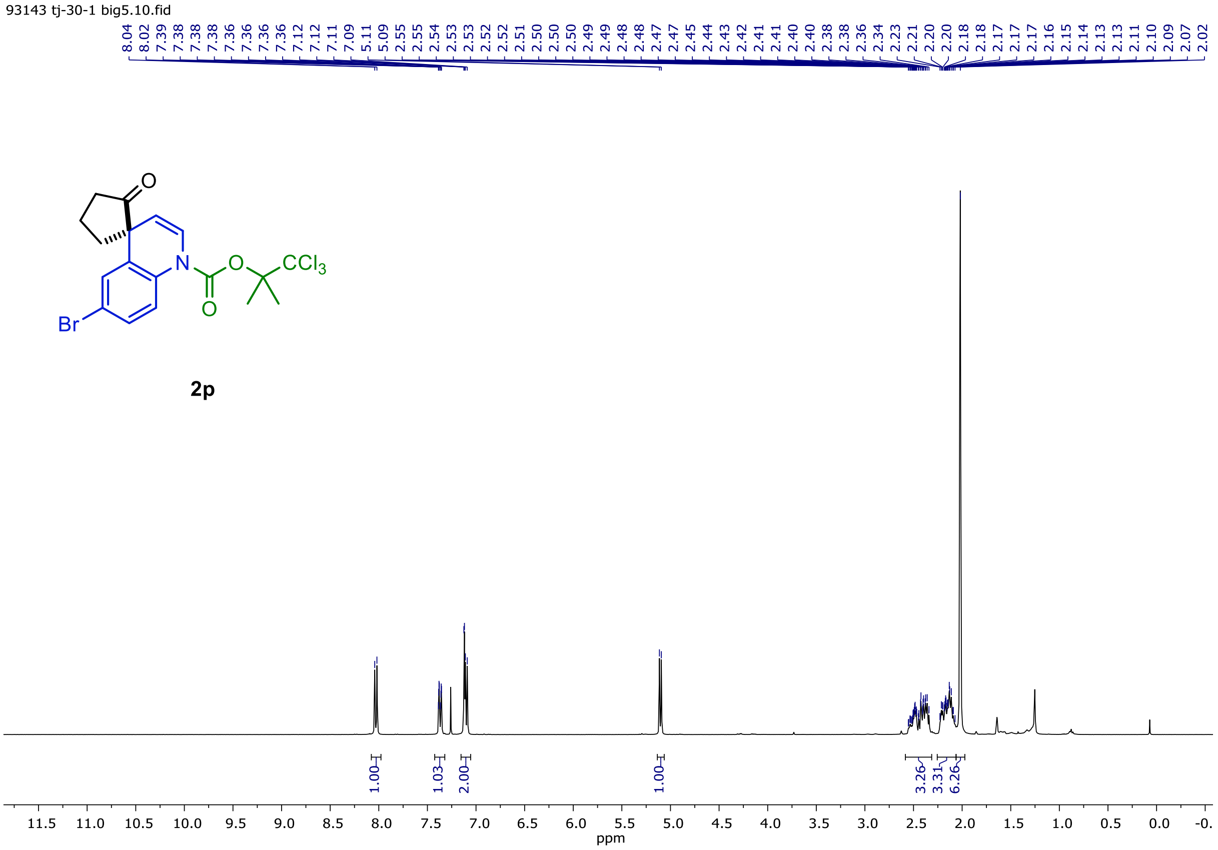

^{13}C NMR (101 MHz, $CDCl_3$) of **2p**

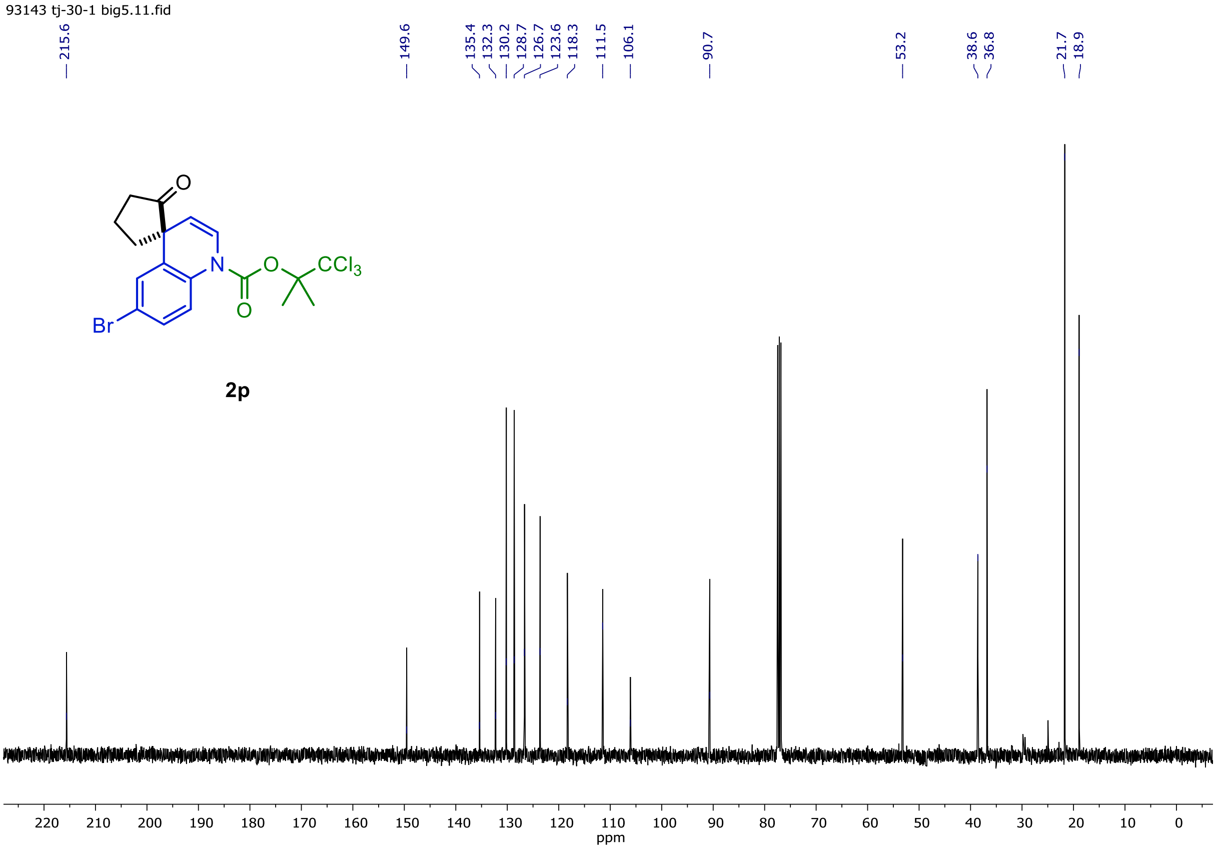

^{1}H NMR (400 MHz, $CDCl_3$) of **2q** (see procedure)

93142 tj-29-1 big5.10.fid

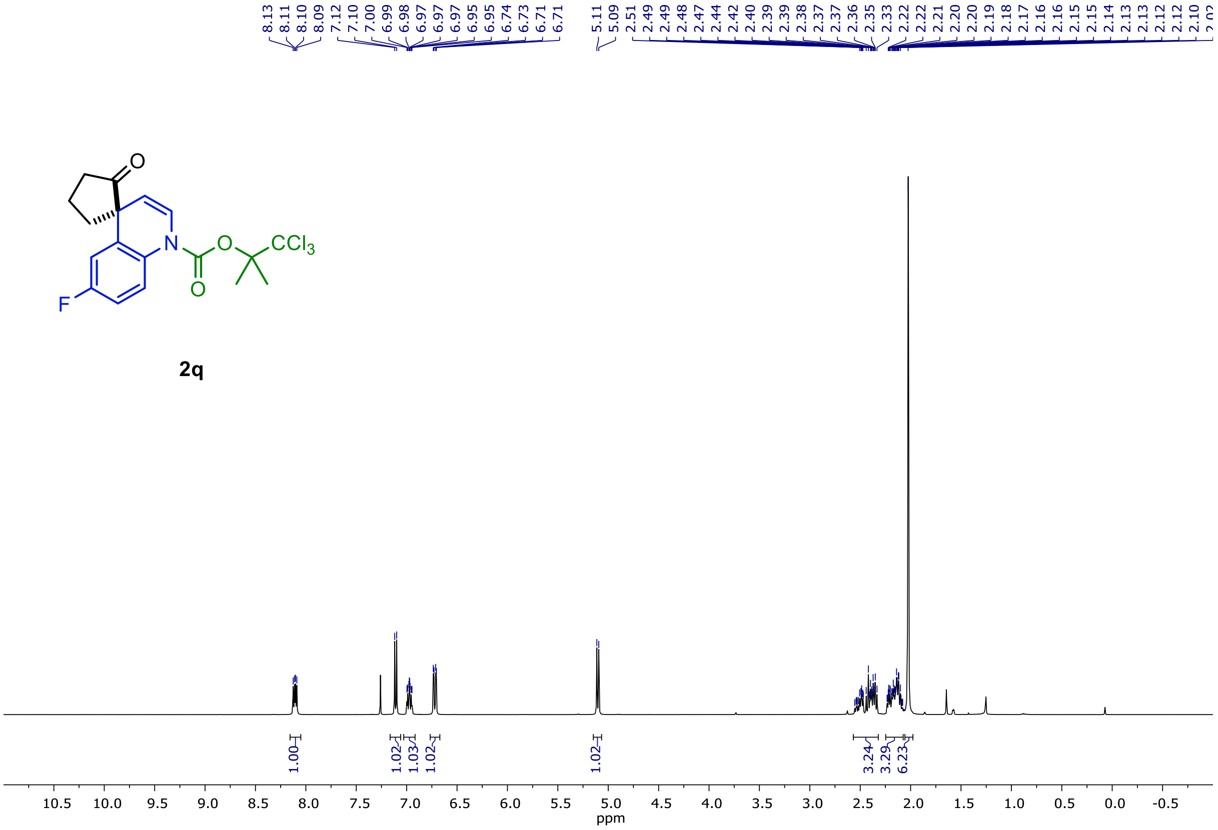

^{13}C NMR (101 MHz, $CDCl_3$) of **2q**

93142 tj-29-1 big5.11.fid

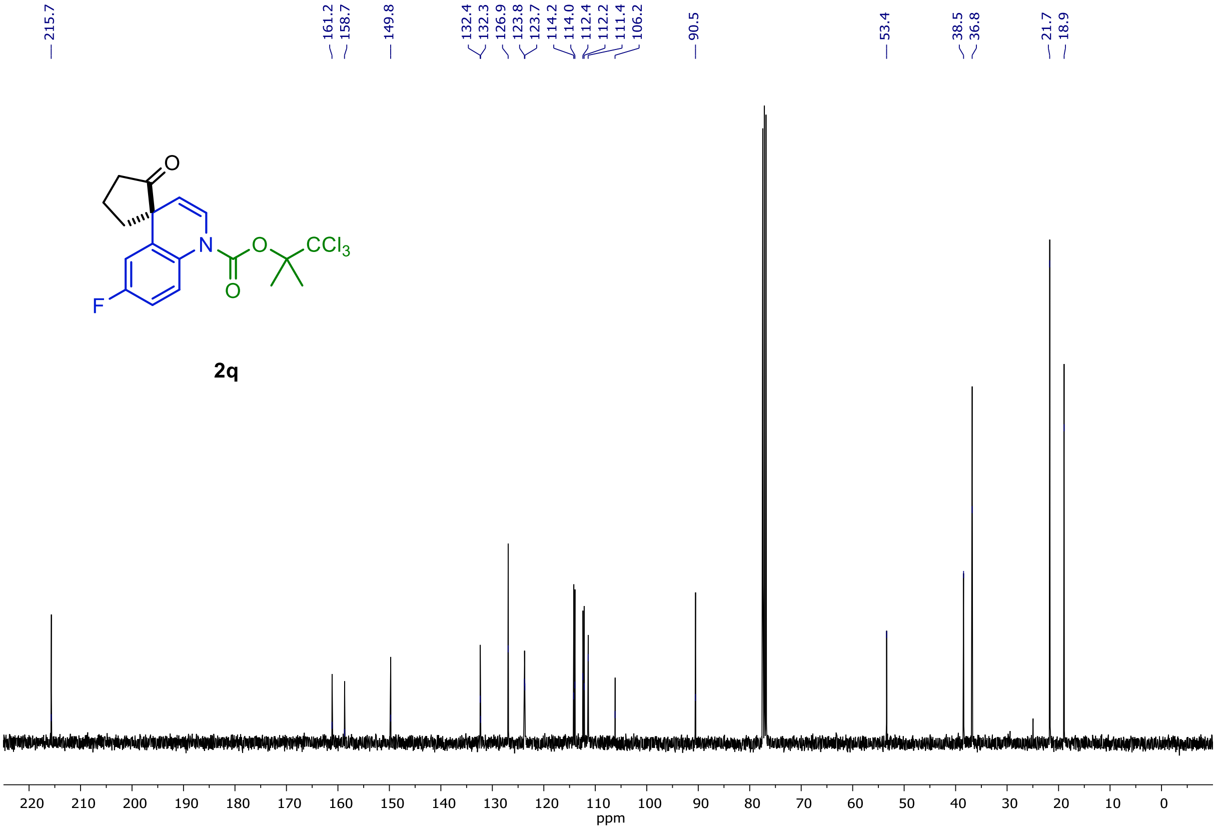

^{1}H NMR (400 MHz, $CDCl_3$) of **2r** (see procedure)

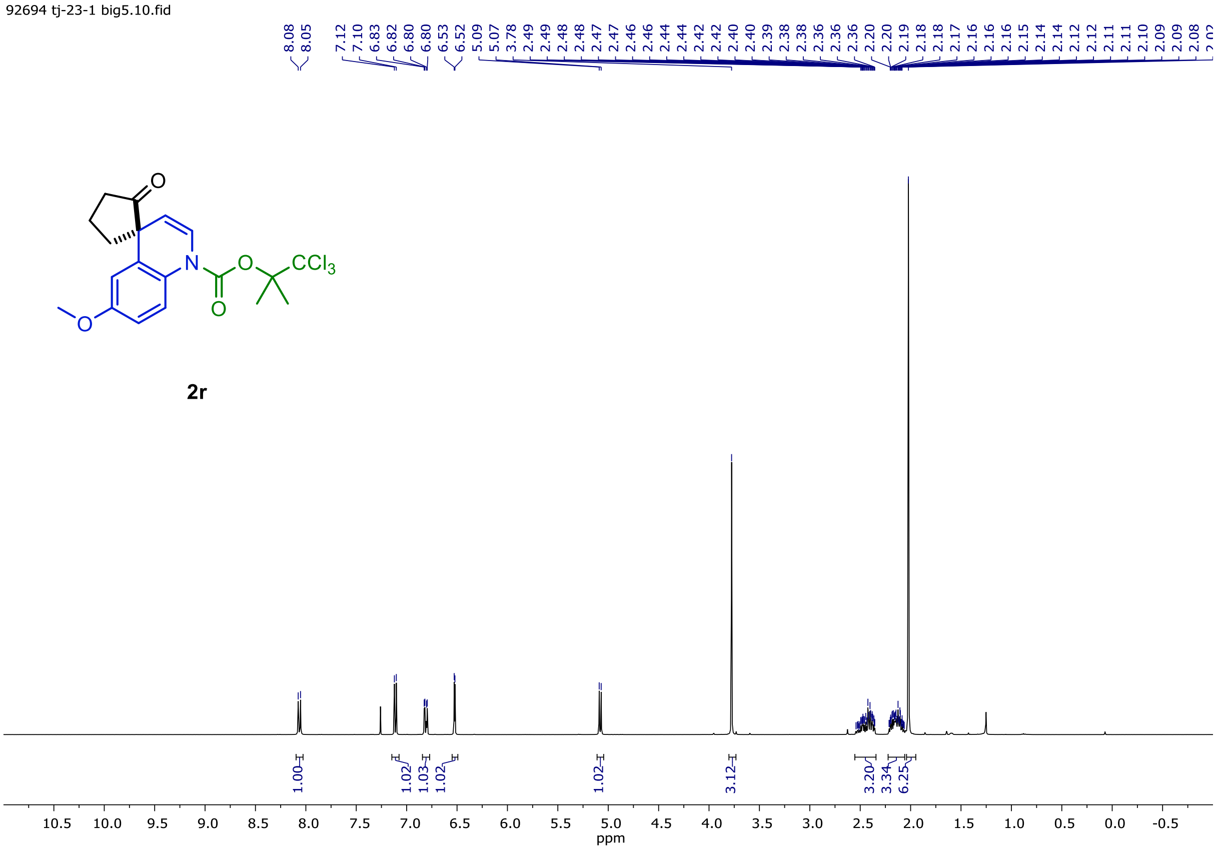

^{13}C NMR (101 MHz, $CDCl_3$) of **2r**

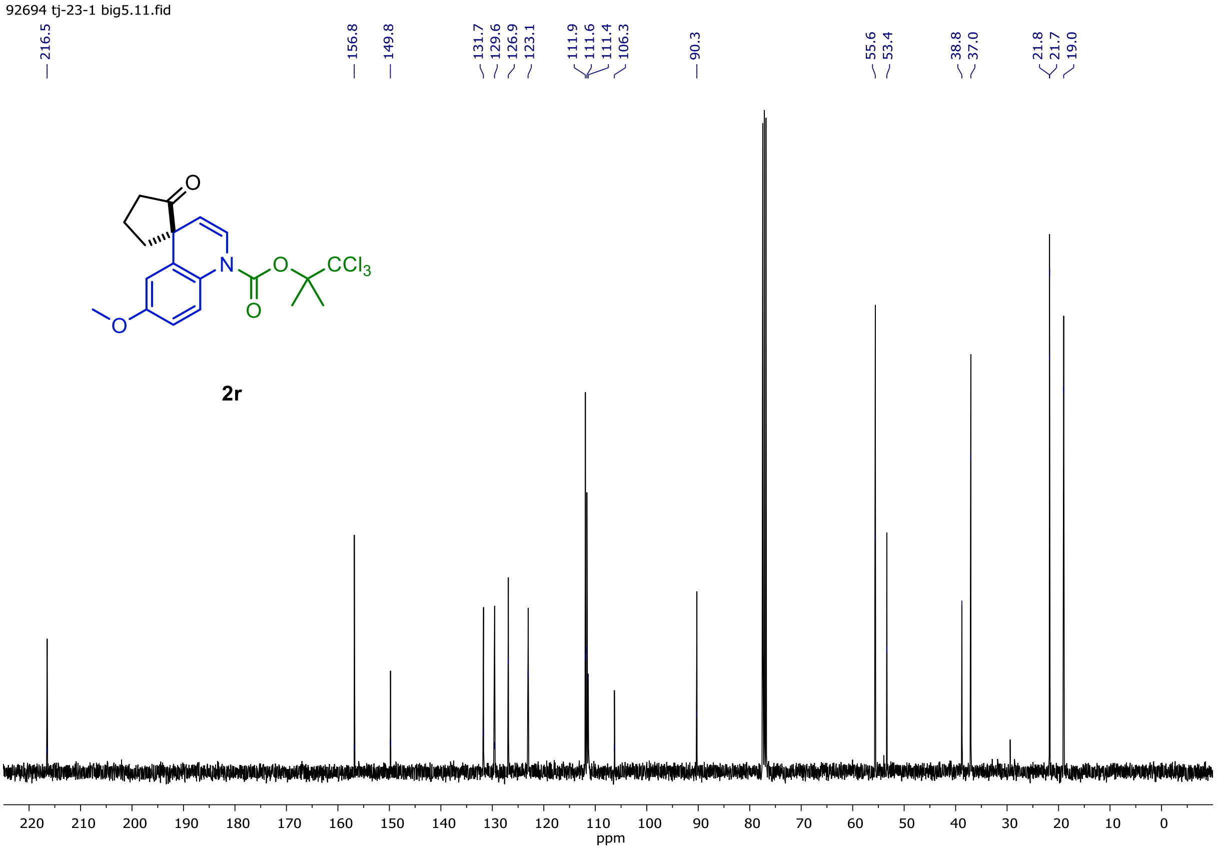

^{1}H NMR (400 MHz, $CDCl_3$) of **2s** (see procedure)

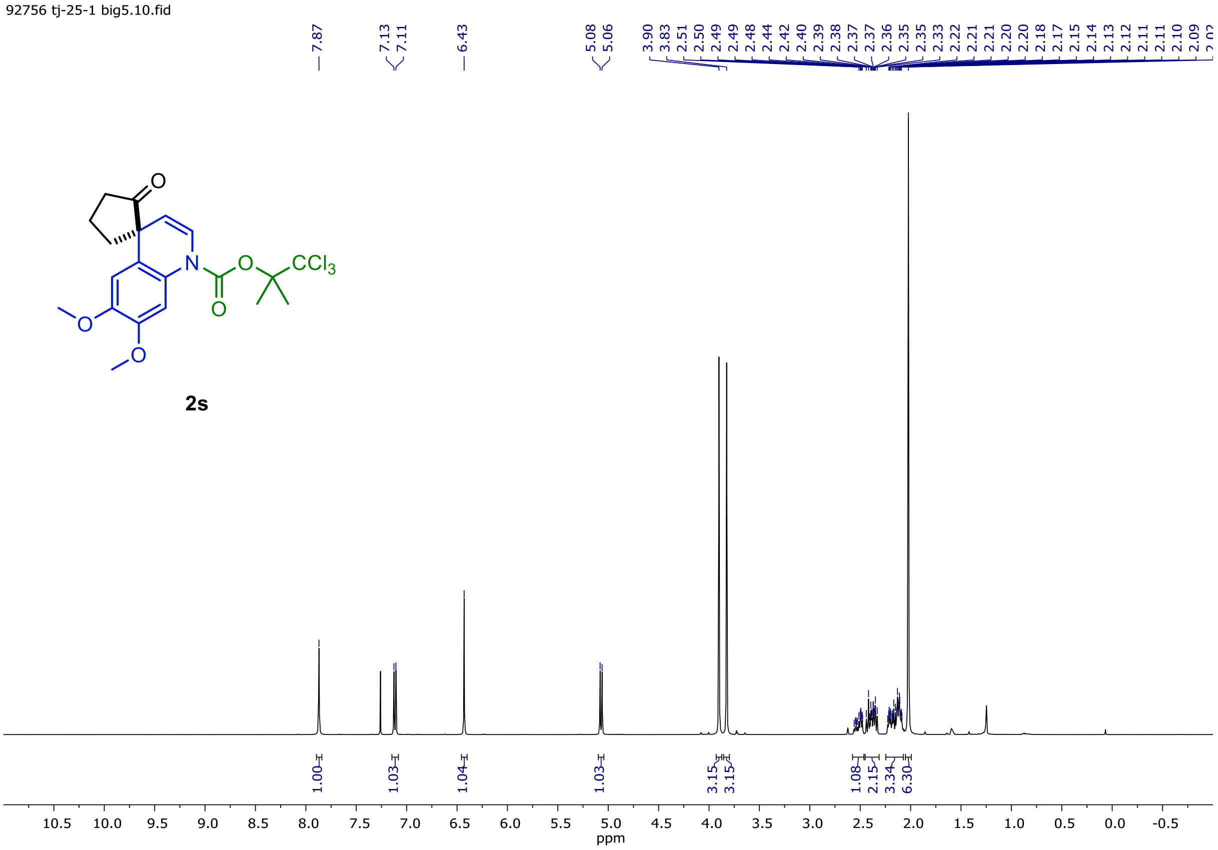

^{13}C NMR (101 MHz, $CDCl_3$) of **2s**

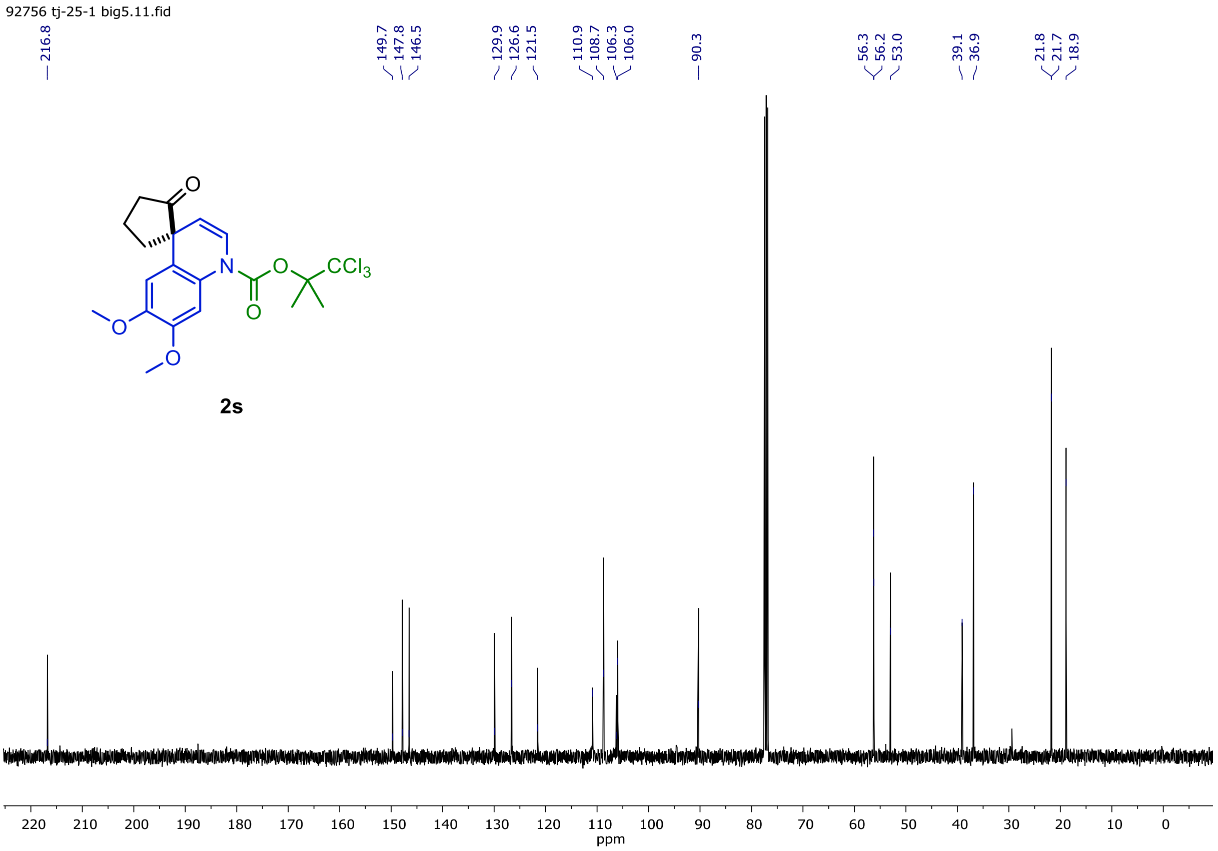

^{1}H NMR (400 MHz, $CDCl_3$) of **2aa** (see procedure)

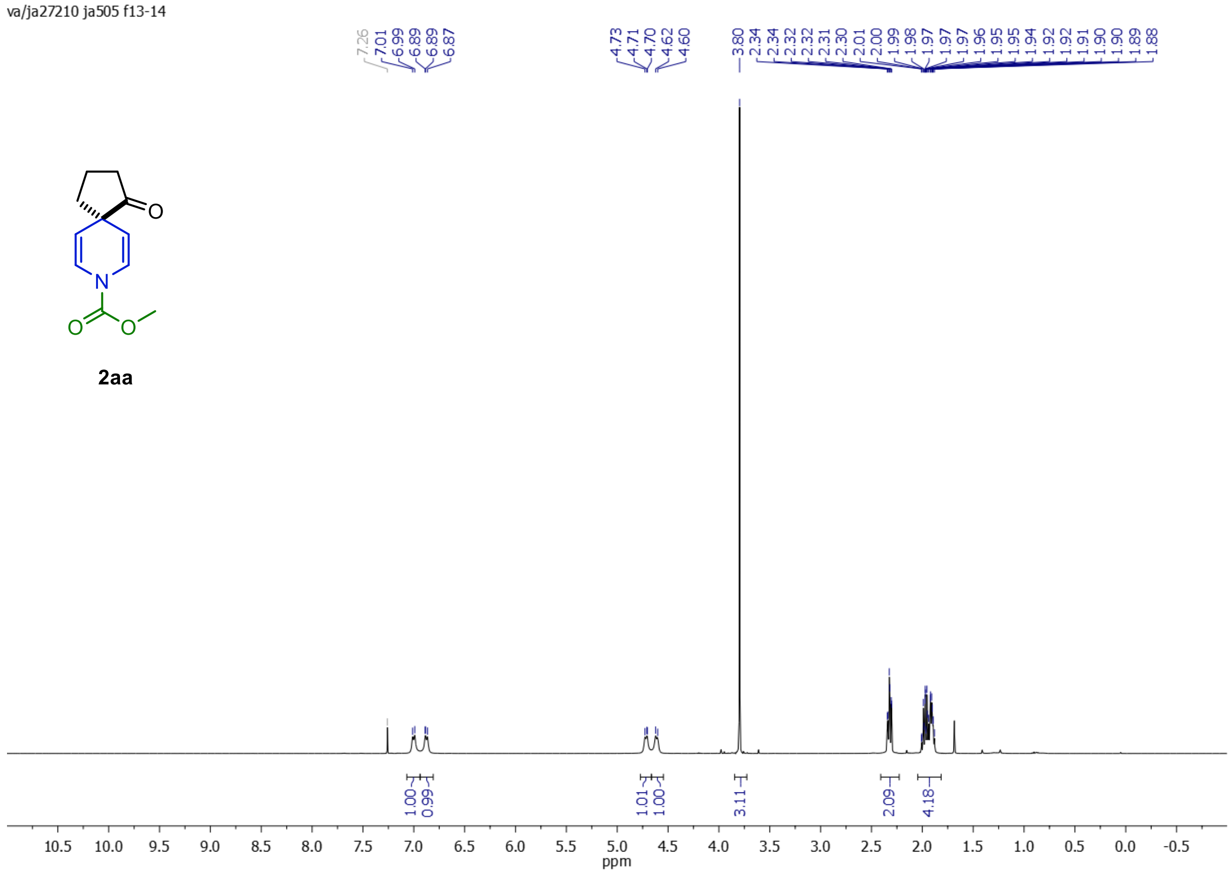

^{13}C NMR (101 MHz, $CDCl_3$) of **2aa**

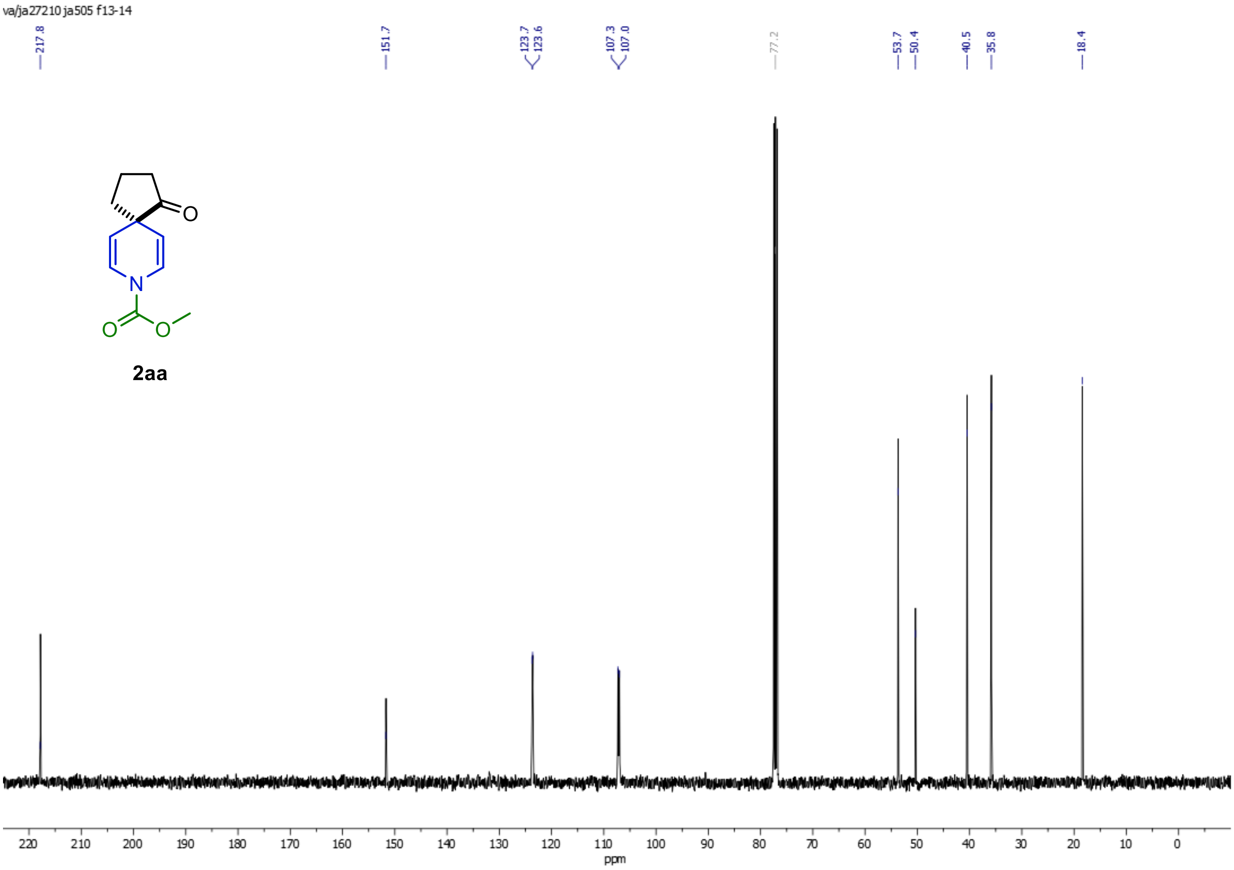

^{1}H NMR (400 MHz, $CDCl_3$) of **3aa** (see procedure)

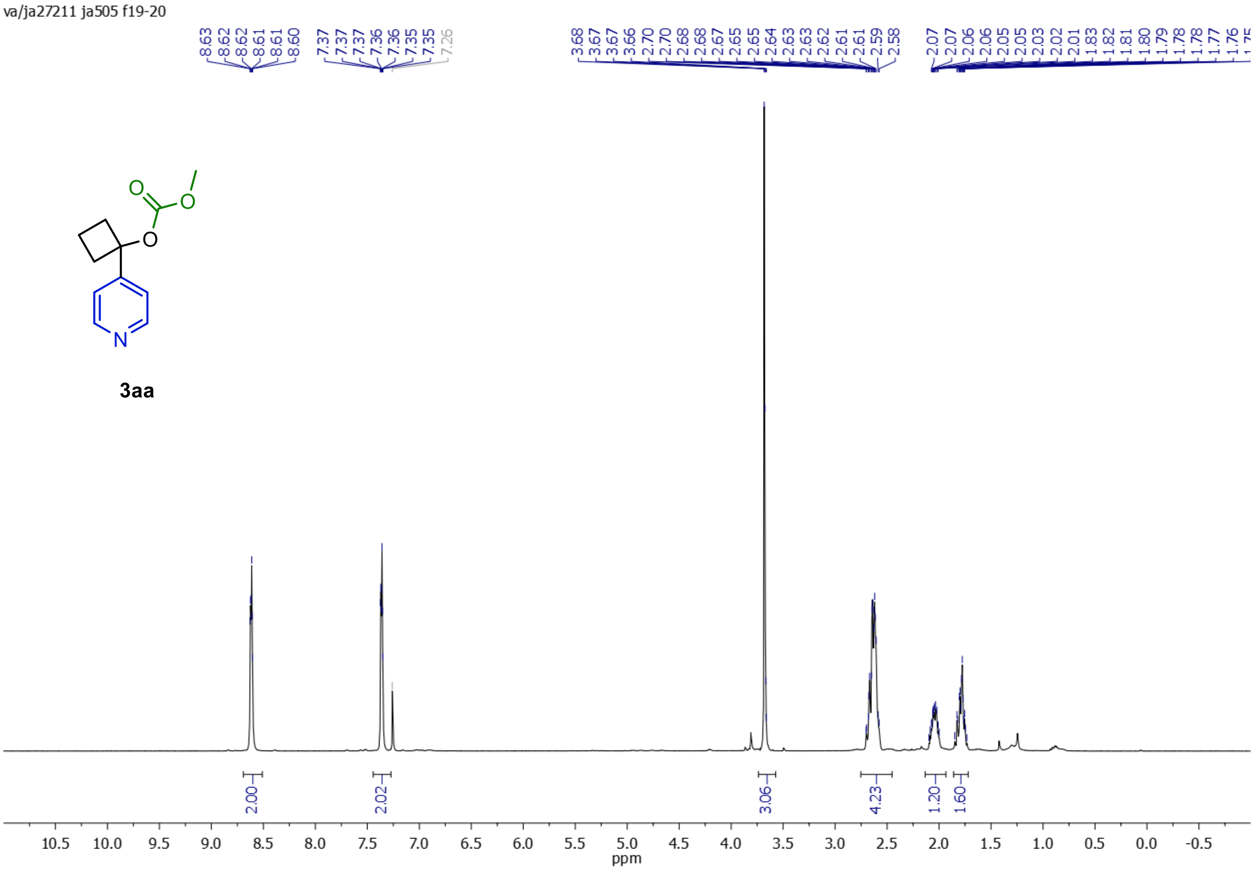

^{13}C NMR (101 MHz, $CDCl_3$) of **3aa**

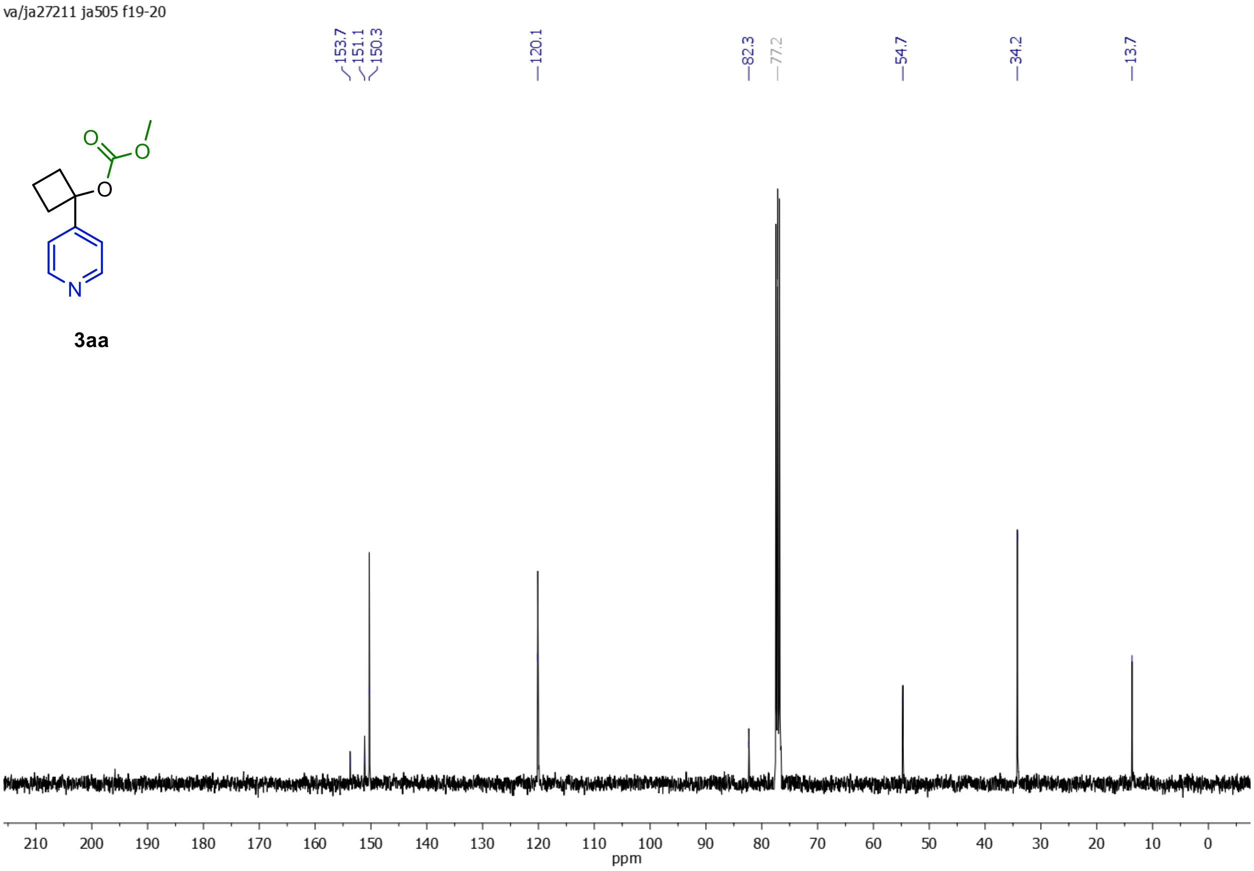

^{1}H NMR (400 MHz, $CDCl_3$) of **2ab** (see procedure)

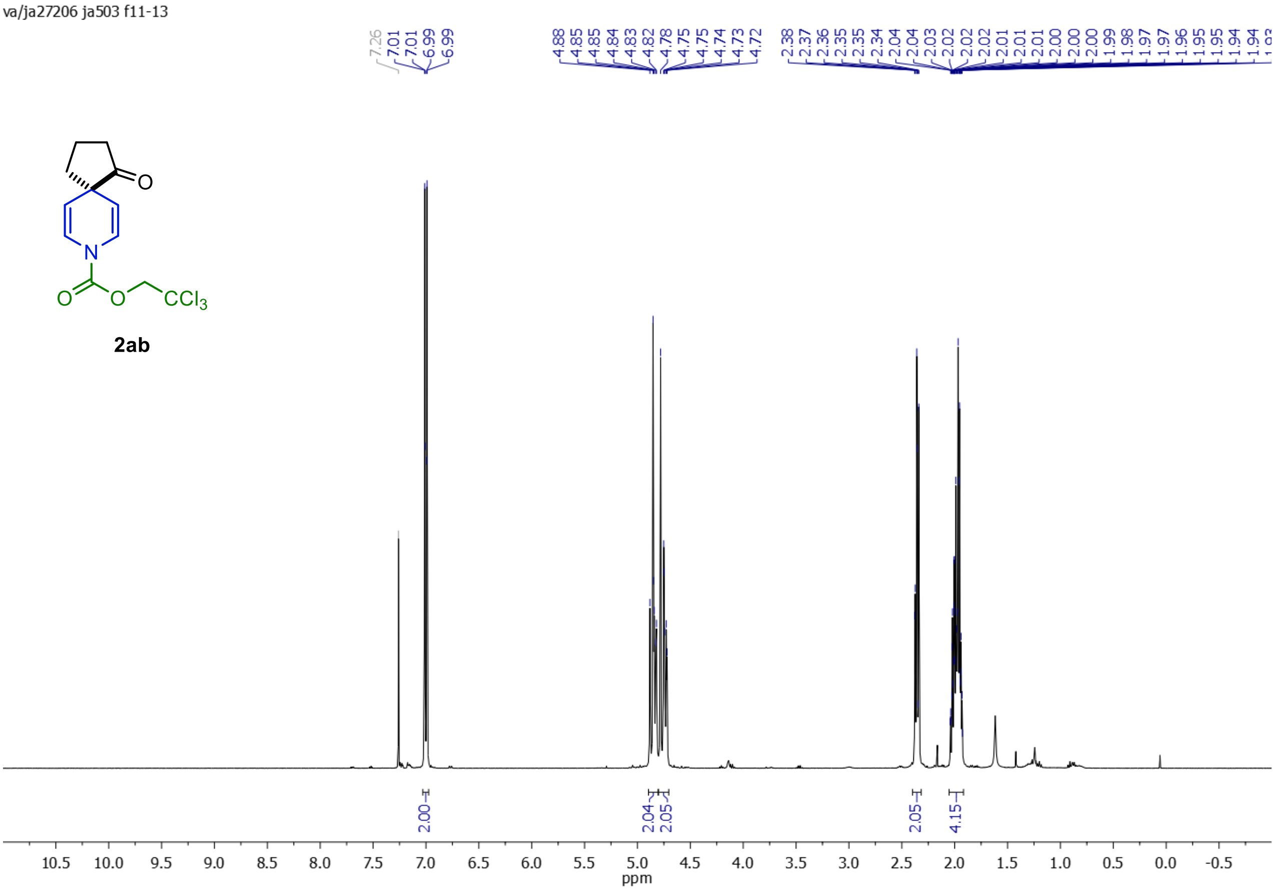

2ab

^{13}C NMR (101 MHz, $CDCl_3$) of **2ab**

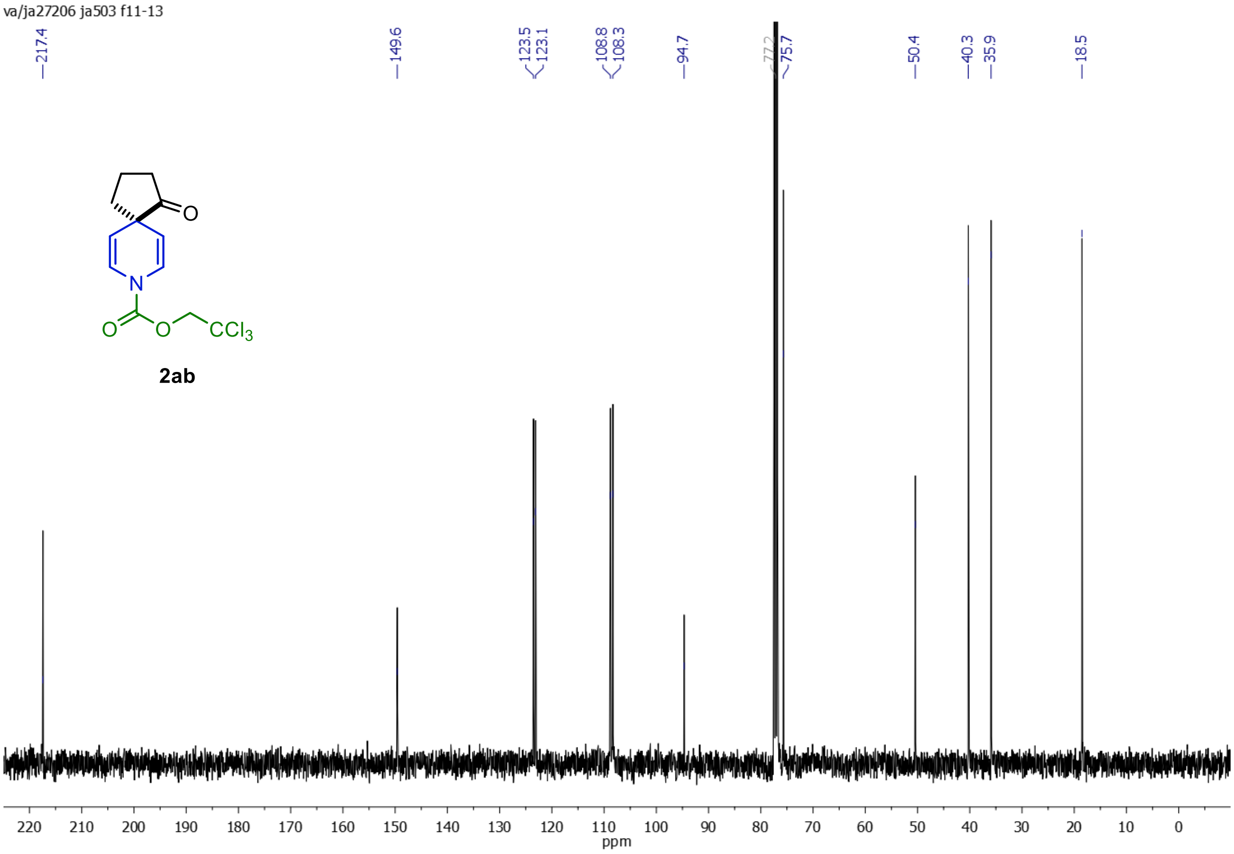

2ab

^{1}H NMR (400 MHz, CDCl$_3$) of **3ab** [(see procedure)]

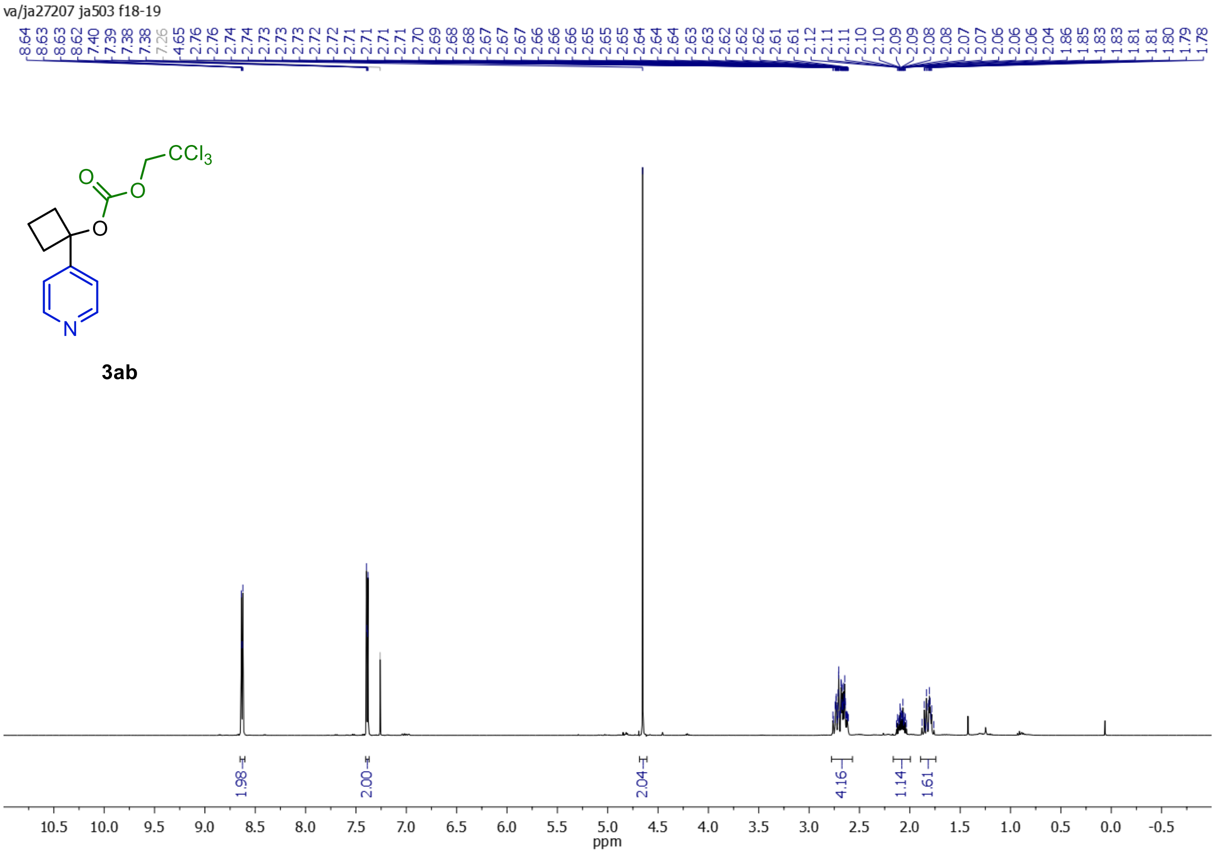

^{13}C NMR (101 MHz, CDCl$_3$) of **3ab**

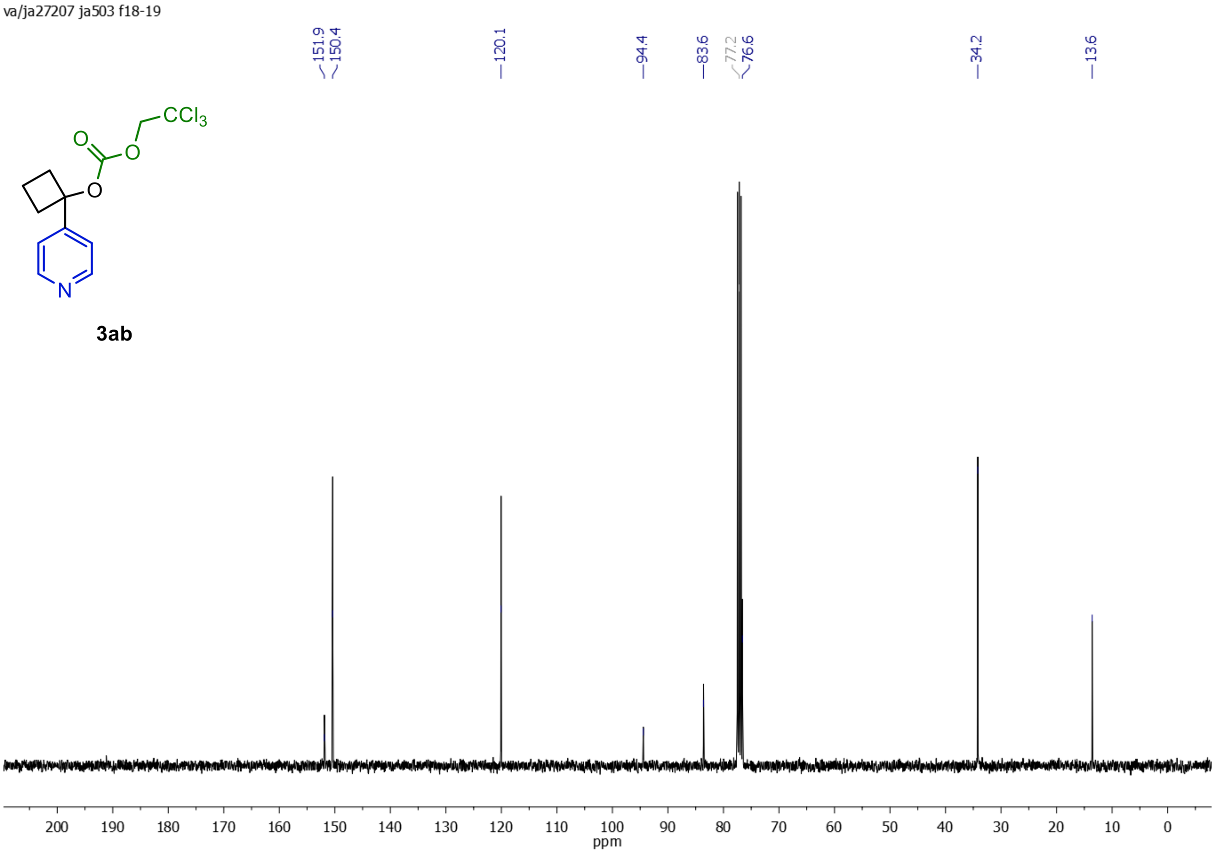

^{1}H NMR (400 MHz, $CDCl_3$) of **2ac** (see procedure)

84194 ja523 f11-12.10.fid

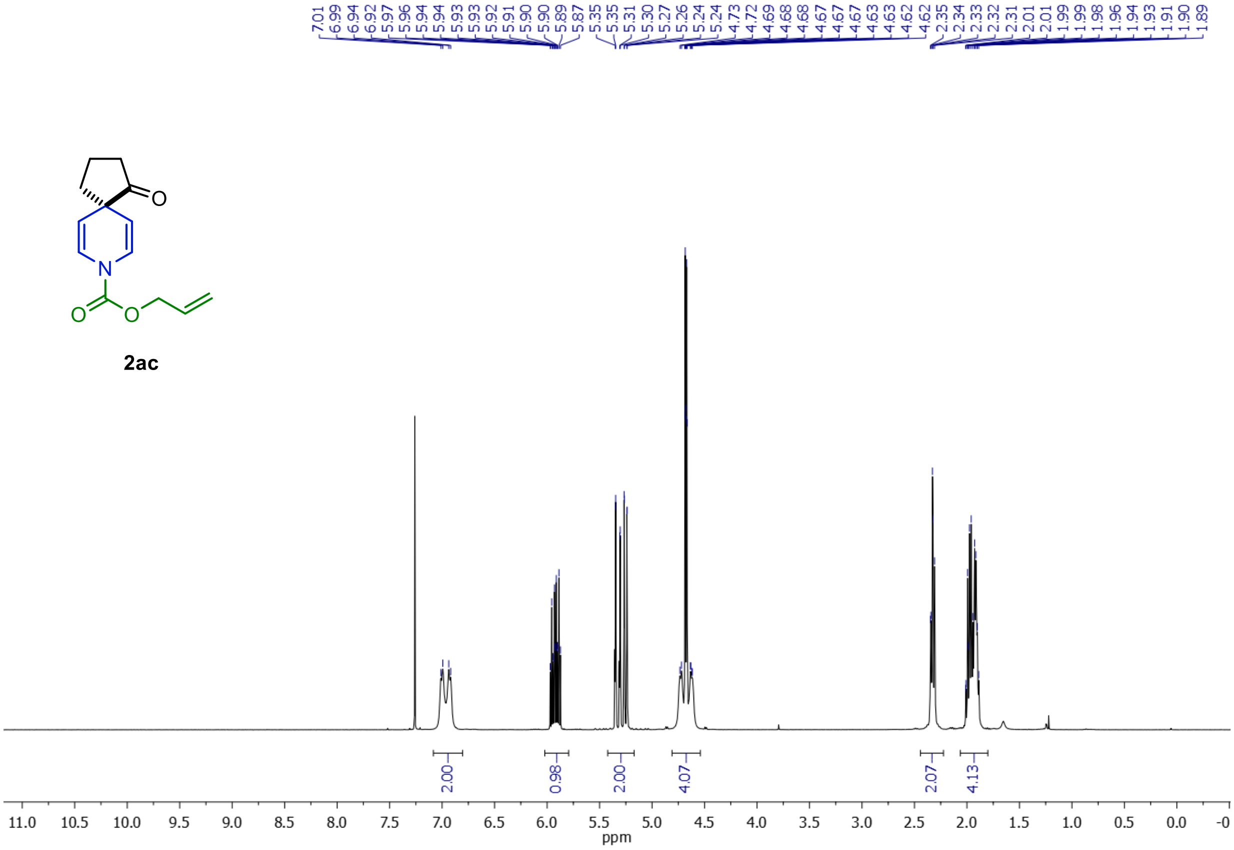

^{13}C NMR (101 MHz, $CDCl_3$) of **2ac**

84194 ja523 f11-12.14.fid

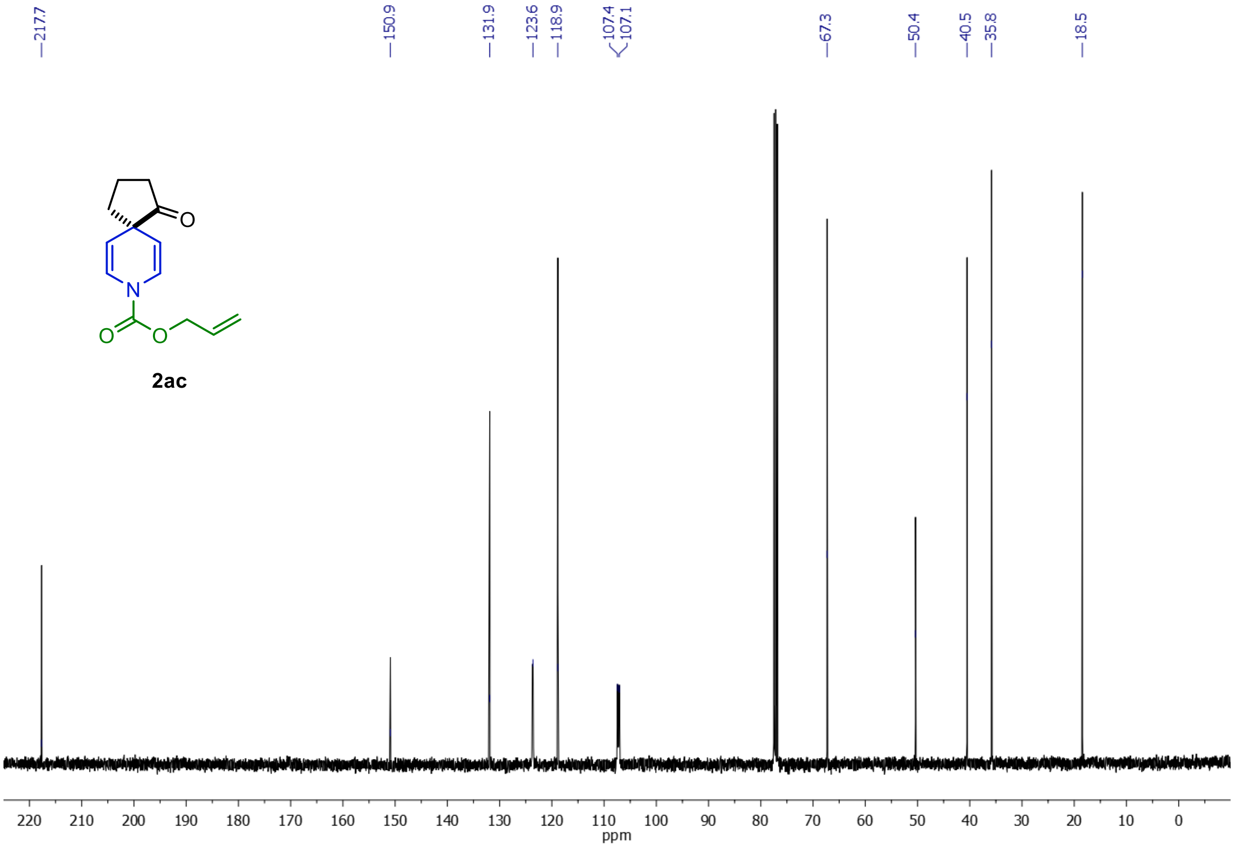

^{1}H NMR (400 MHz, $CDCl_3$) of **2ad** (see procedure)

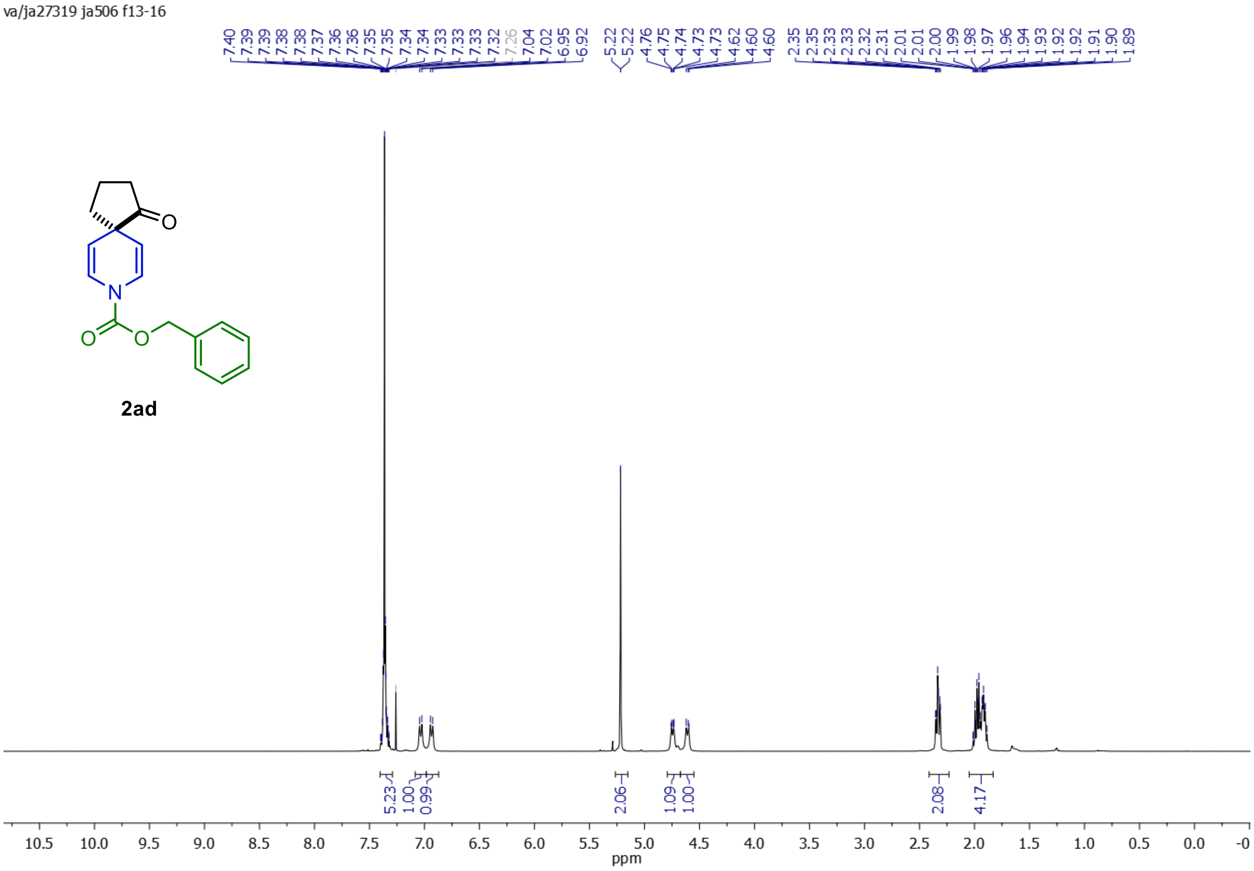

^{13}C NMR (101 MHz, $CDCl_3$) of **2ad**

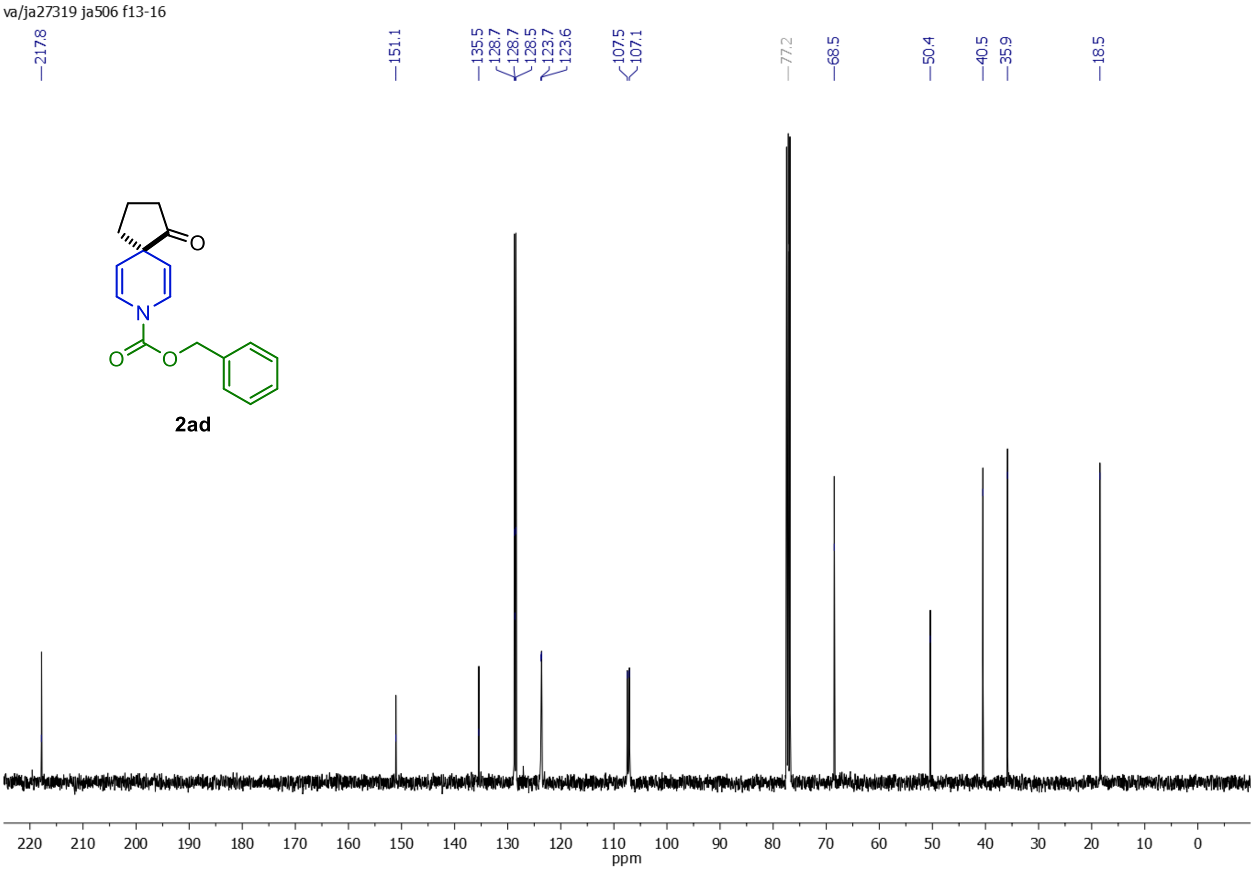

^{1}H NMR (400 MHz, $CDCl_3$) of **3ad** (see procedure)

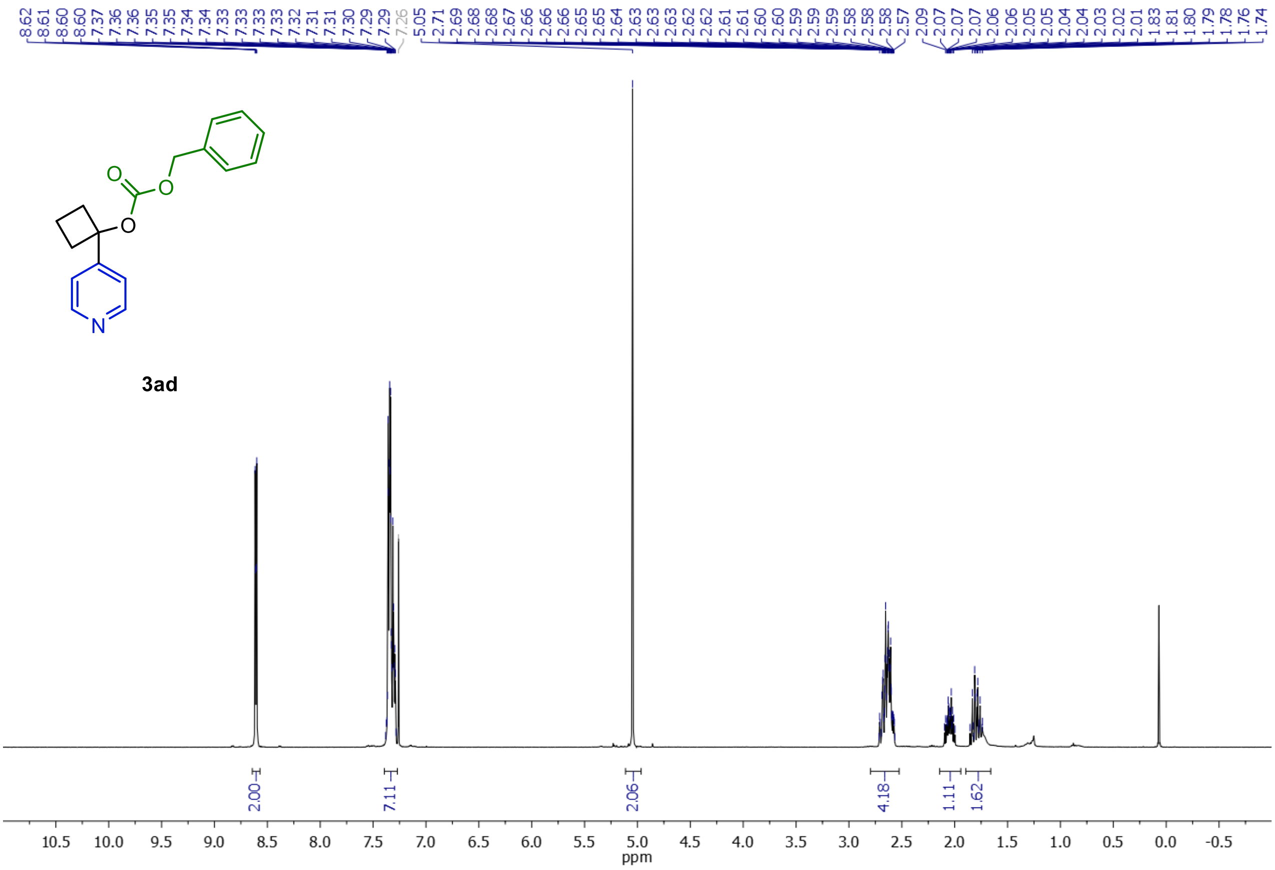

^{13}C NMR (101 MHz, $CDCl_3$) of **3ad**

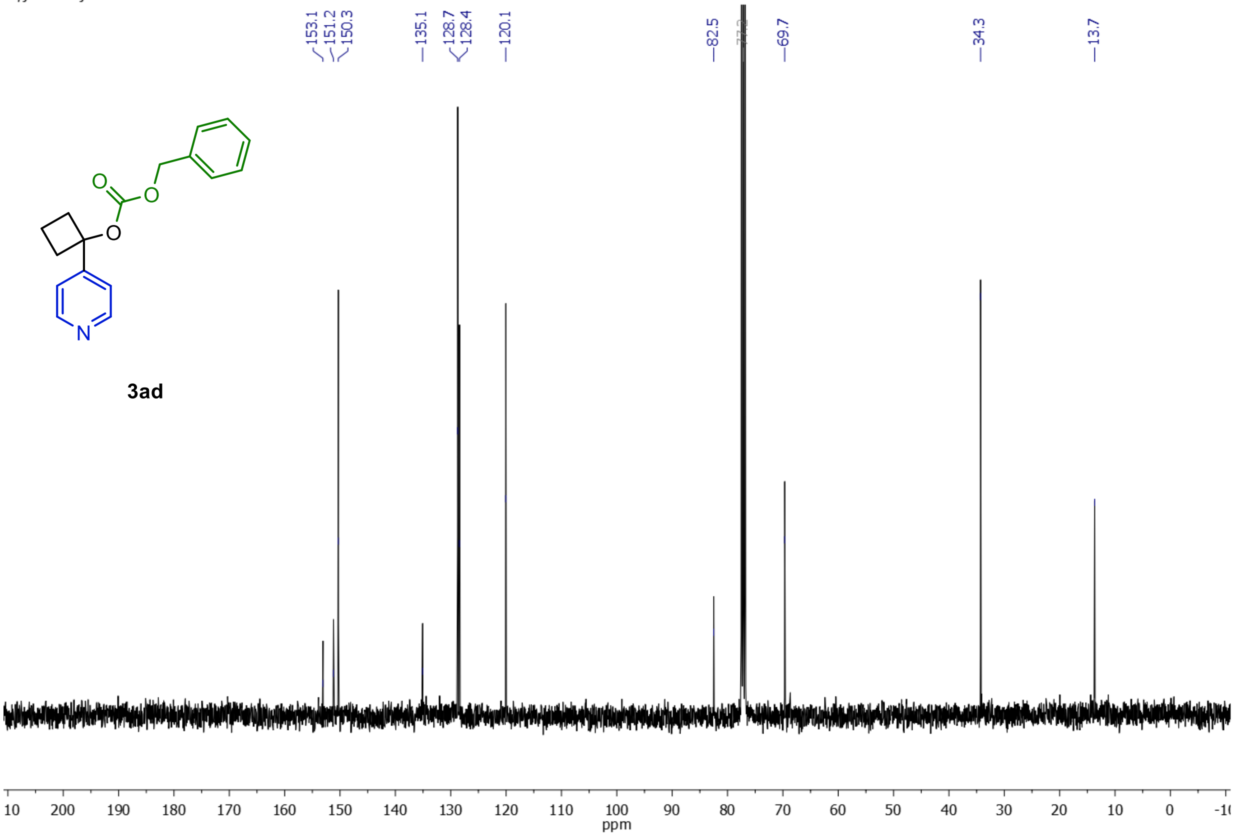

^{1}H NMR (400 MHz, $CDCl_3$) of **2ae** (see procedure)

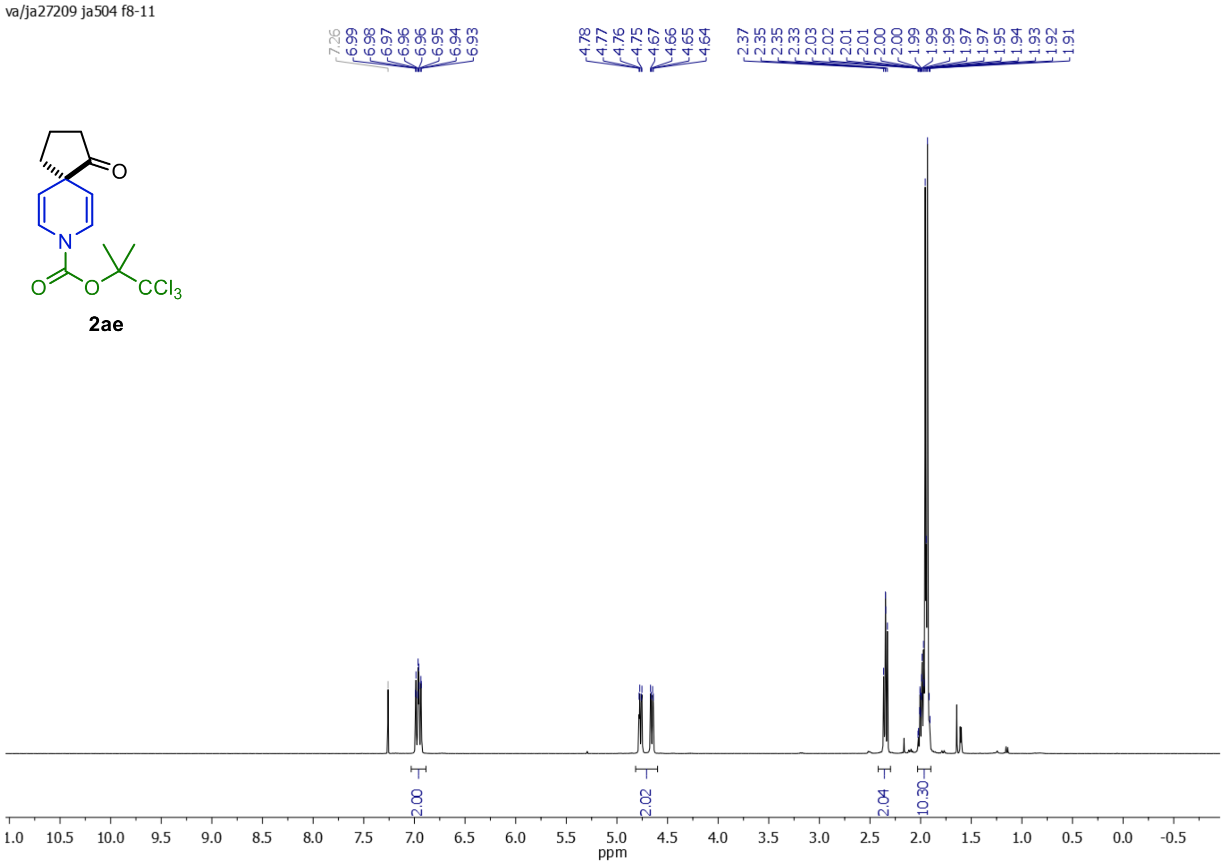

^{13}C NMR (101 MHz, $CDCl_3$) of **2ae**

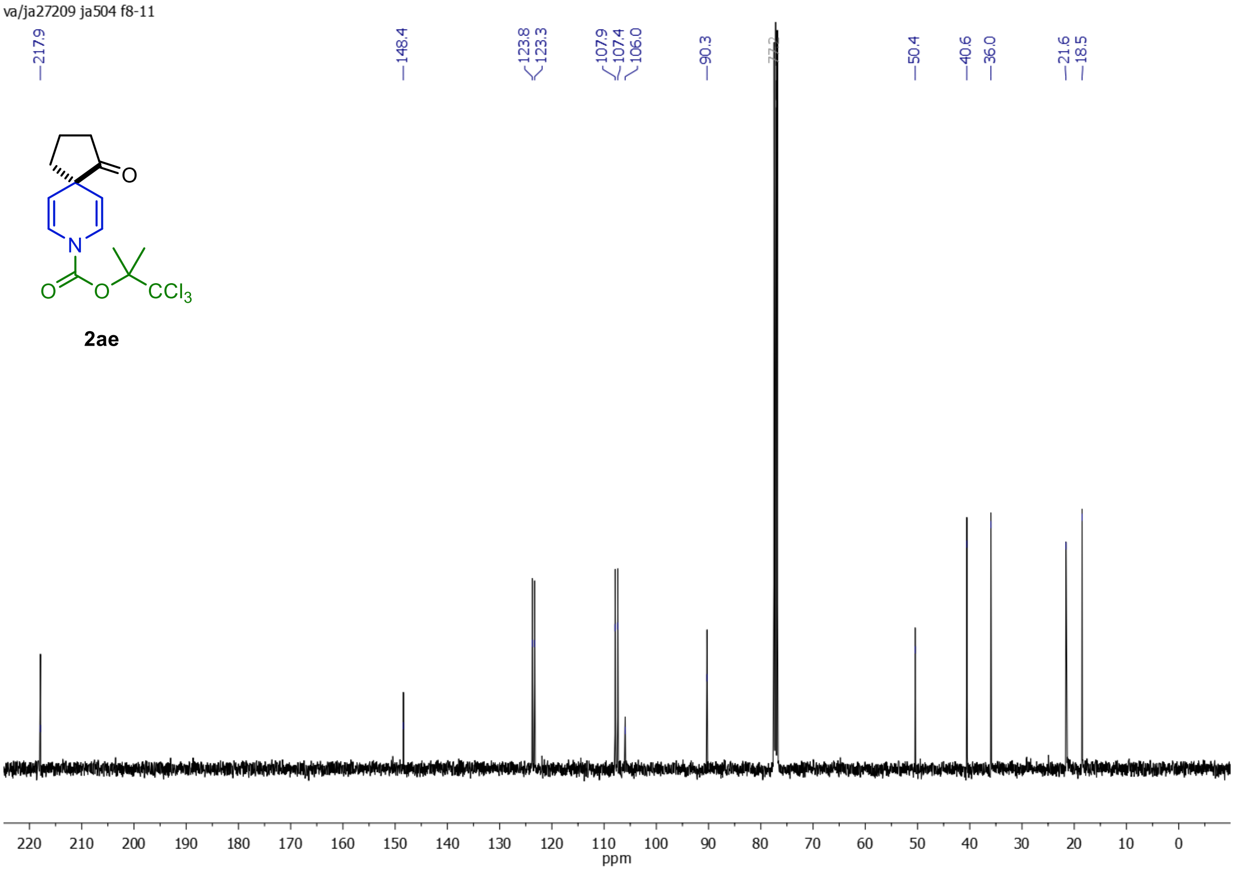

^{1}H NMR (400 MHz, $CDCl_3$) of **2af** (see procedure)

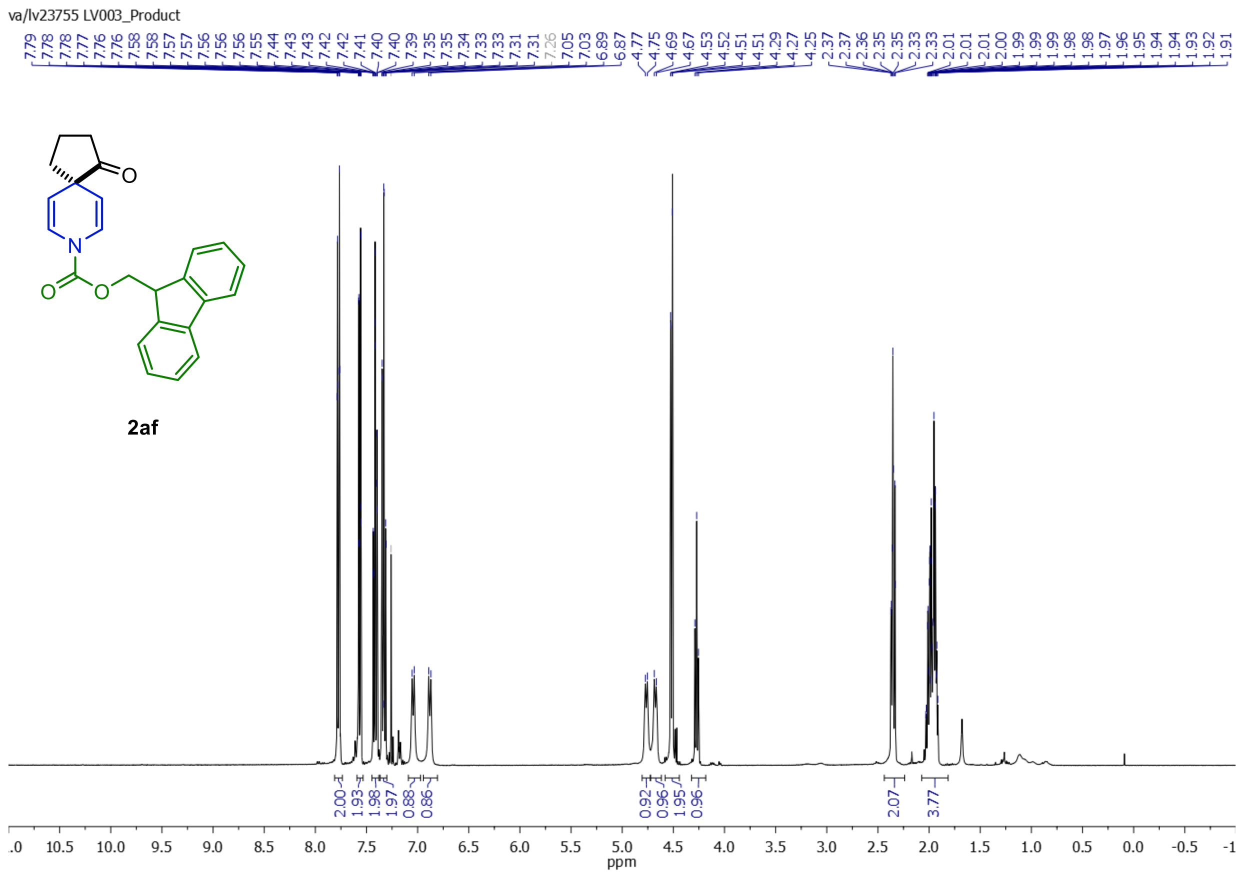

^{13}C NMR (101 MHz, $CDCl_3$) of **2af**

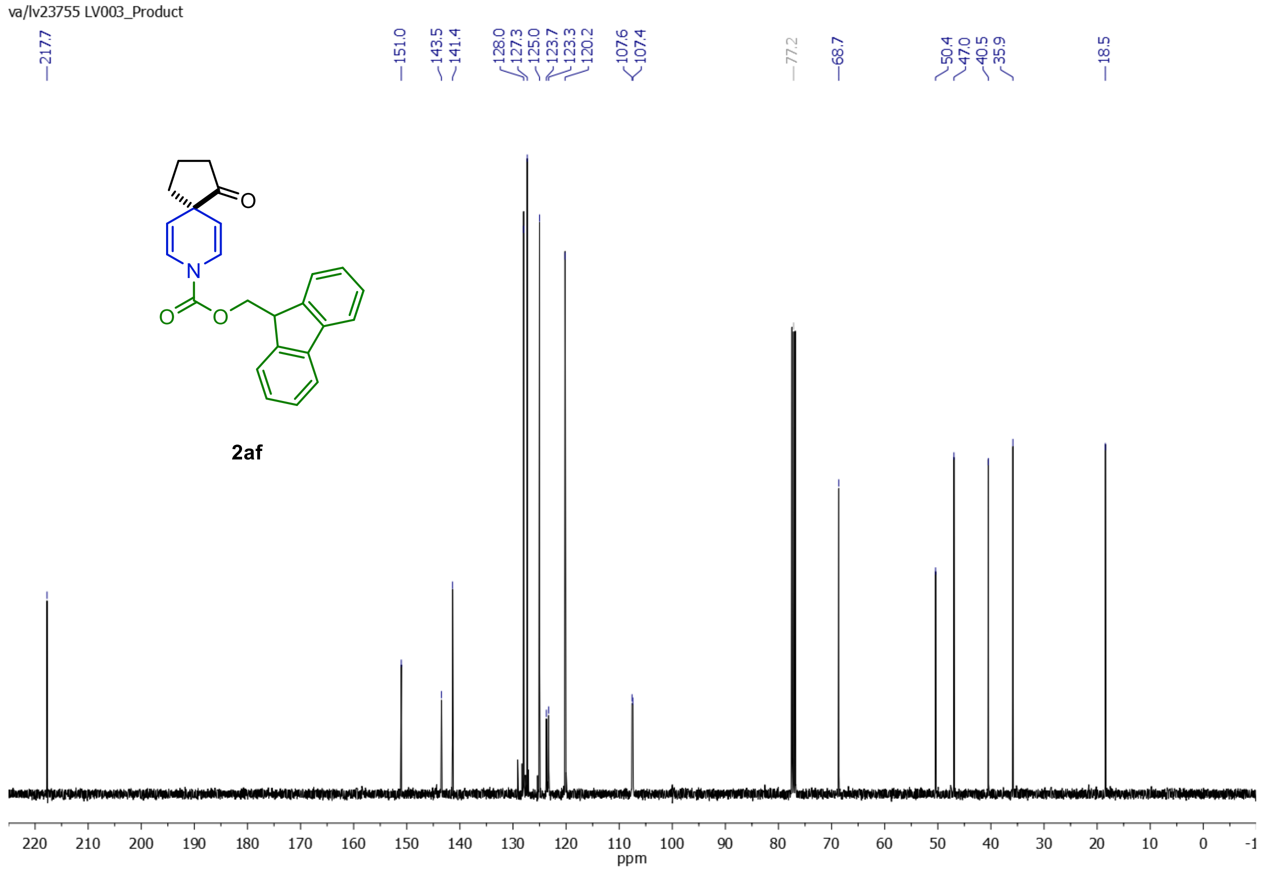

^{1}H NMR (400 MHz, $CDCl_3$) of **2ag** (see procedure)

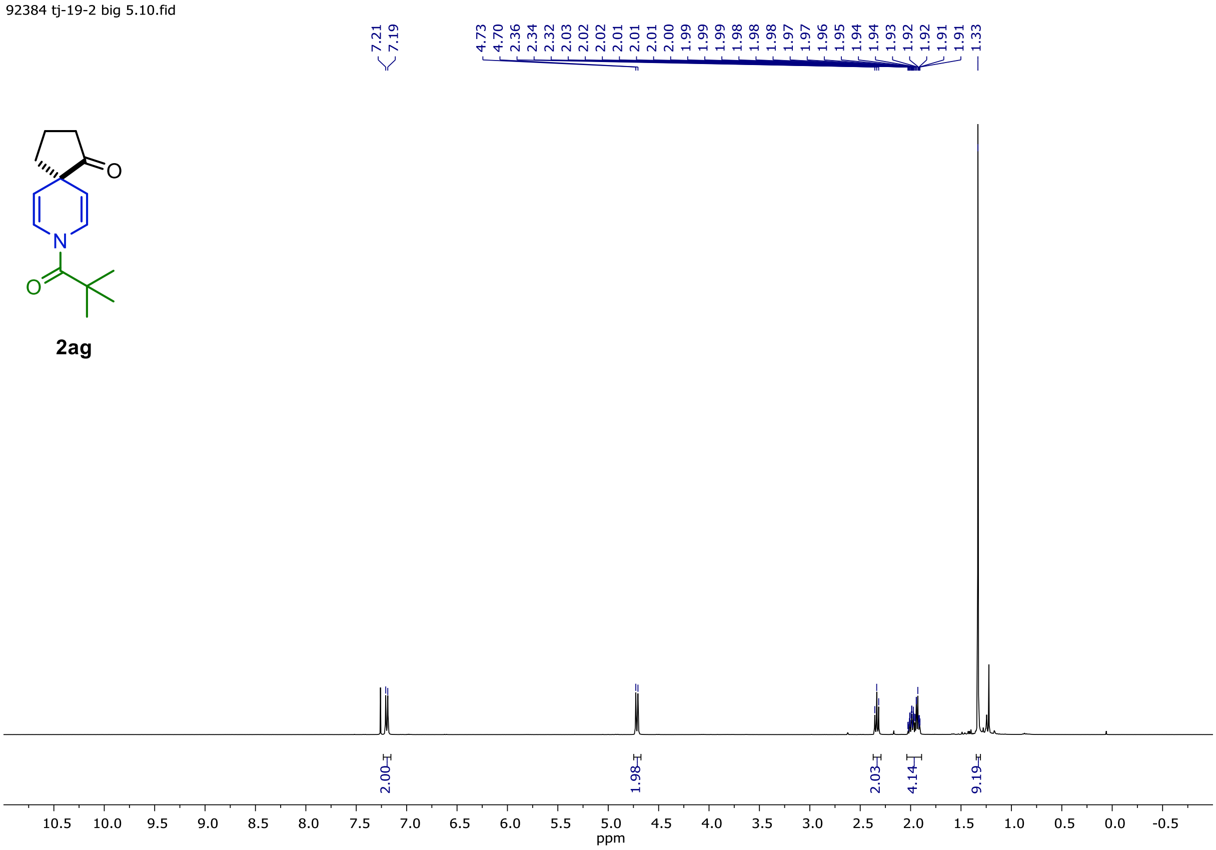

^{13}C NMR (101 MHz, $CDCl_3$) of **2ag**

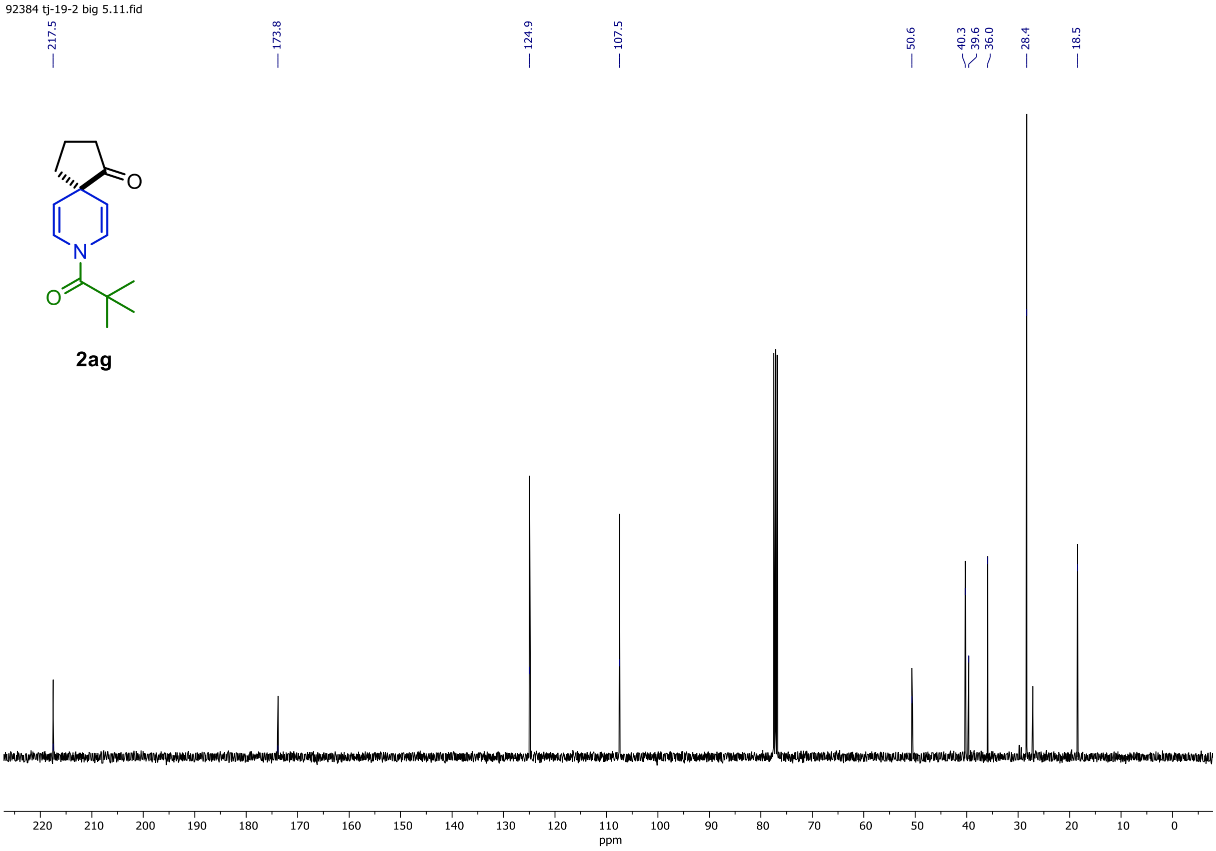

^{1}H NMR (400 MHz, $CDCl_3$) of **2ah** (see procedure)

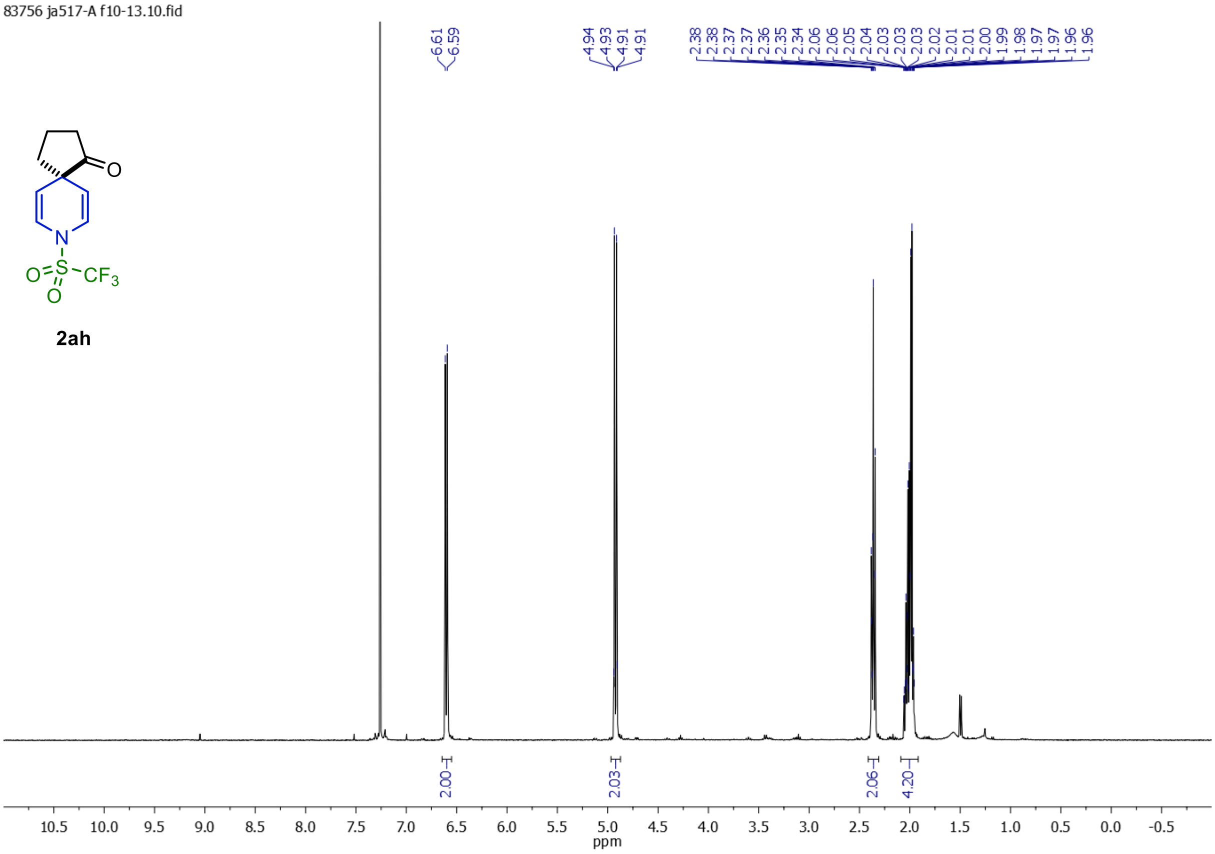

^{13}C NMR (101 MHz, $CDCl_3$) of **2ah**

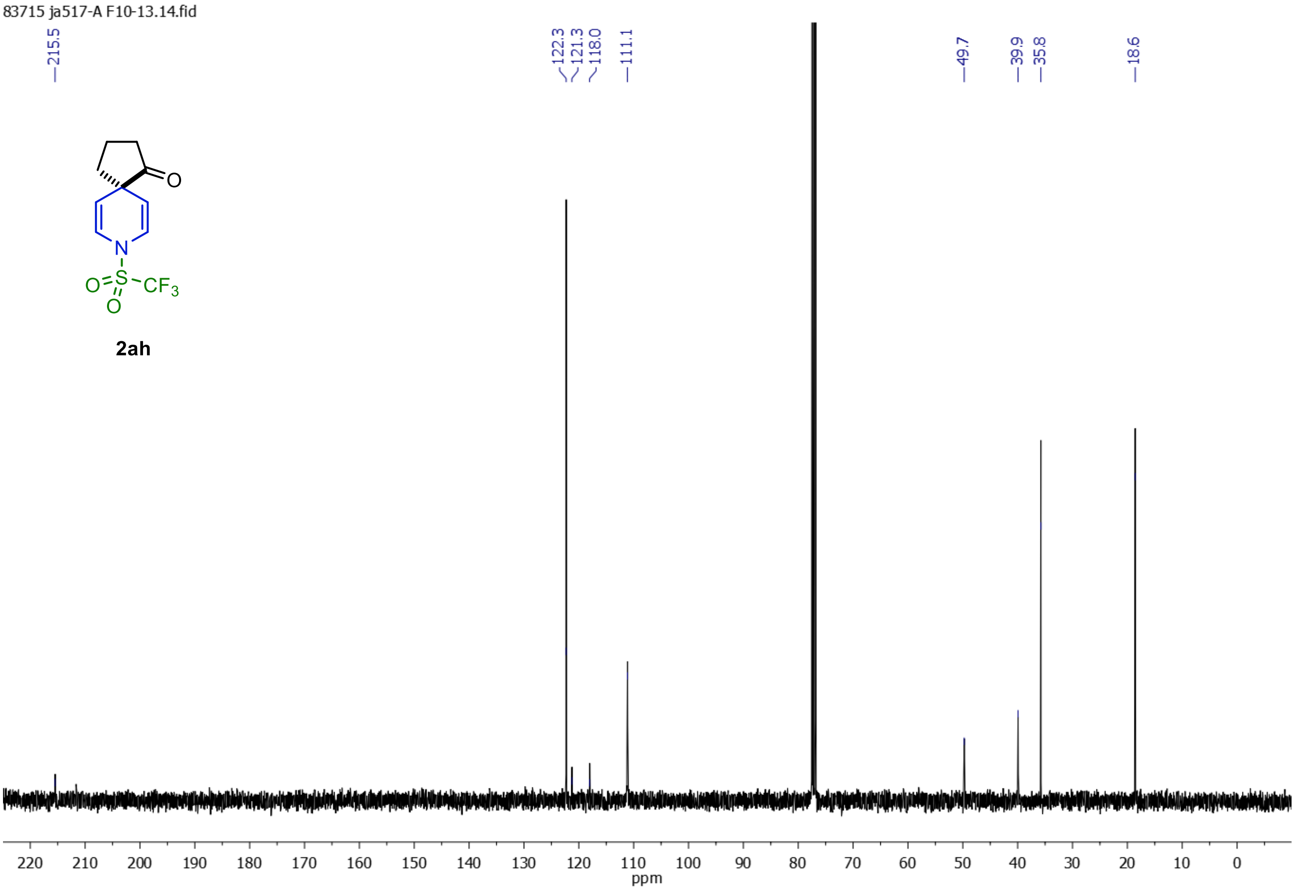

^{1}H NMR (400 MHz, $CDCl_3$) of **2ai** (see procedure)

83916 ja520-A f14-17.10.fid

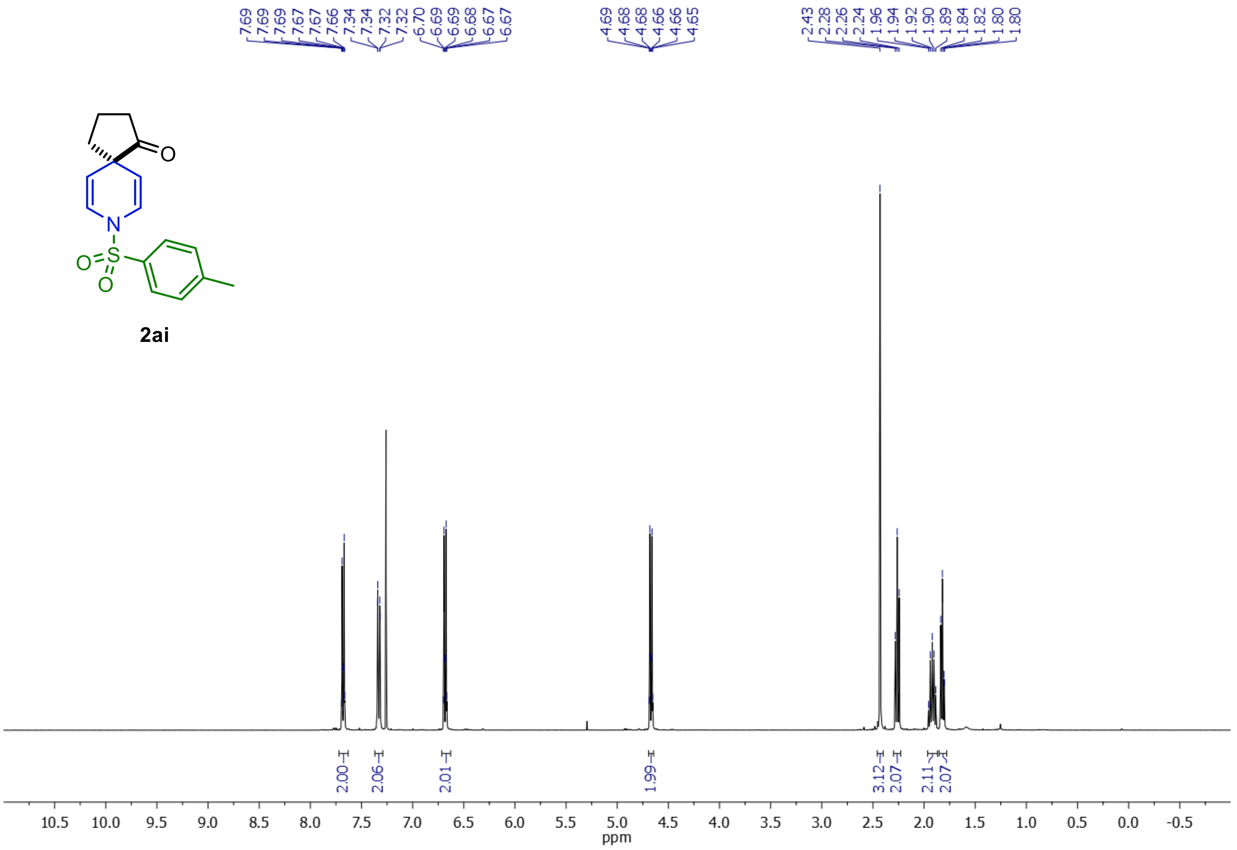

^{13}C NMR (101 MHz, $CDCl_3$) of **2ai**

83916 ja520-A f14-17.14.fid

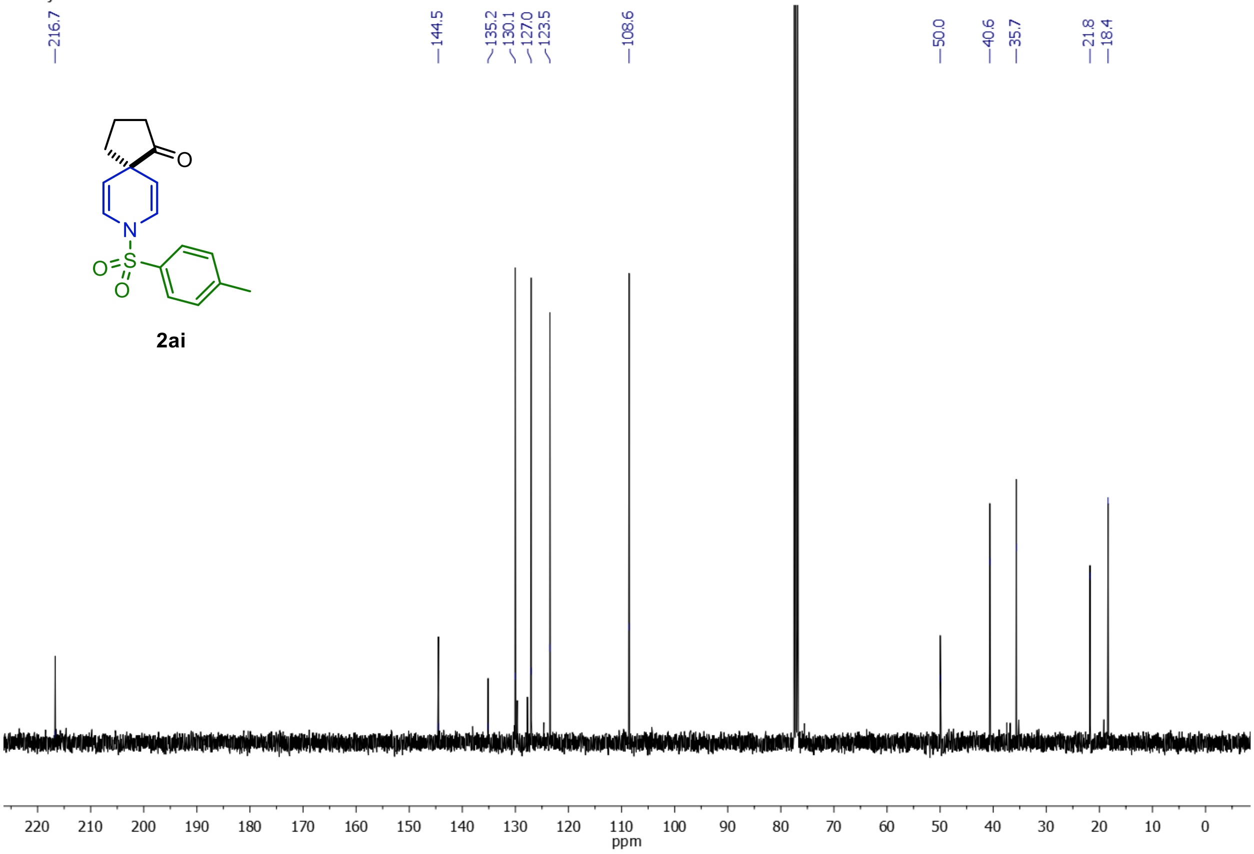

^{1}H NMR (400 MHz, CDCl$_3$) of **2aj** (see procedure)

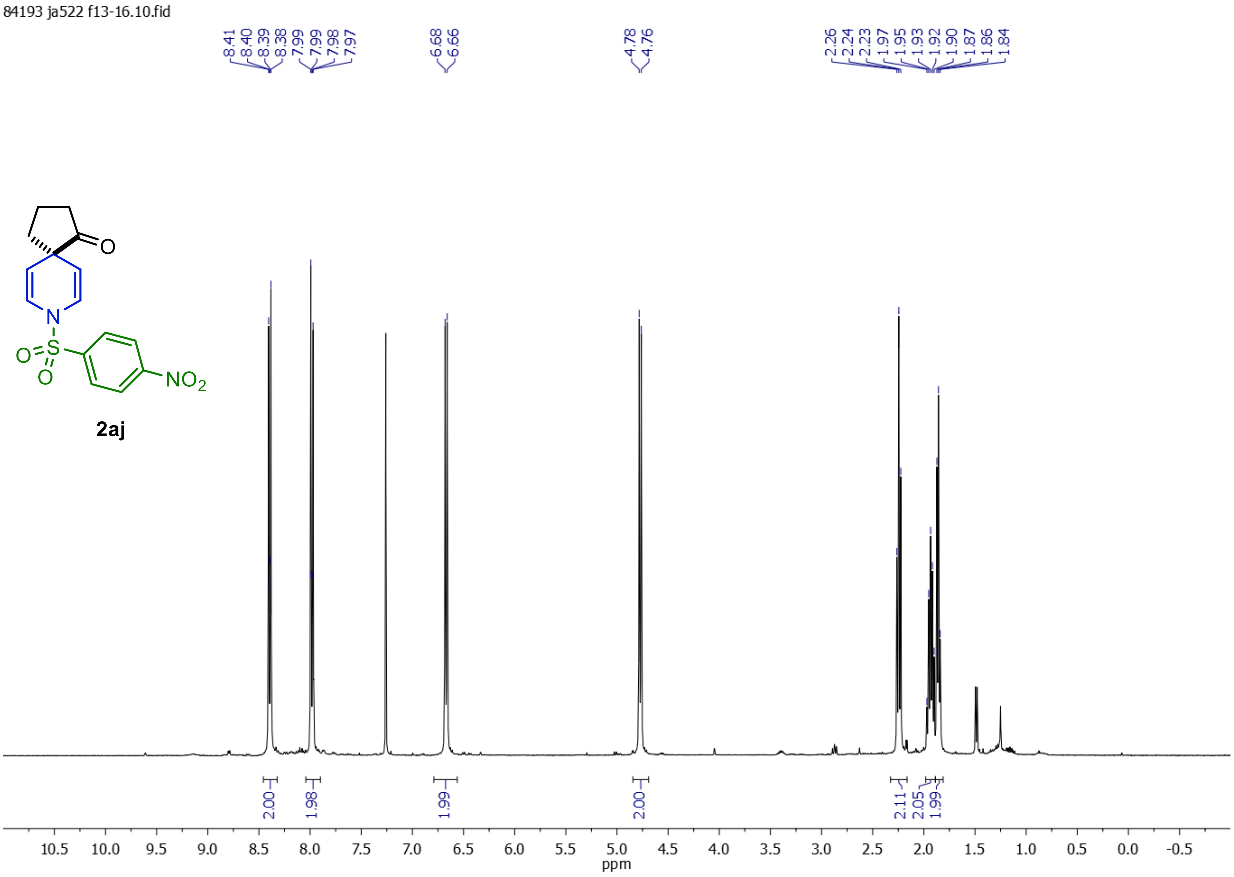

^{13}C NMR (101 MHz, CDCl$_3$) of **2aj**

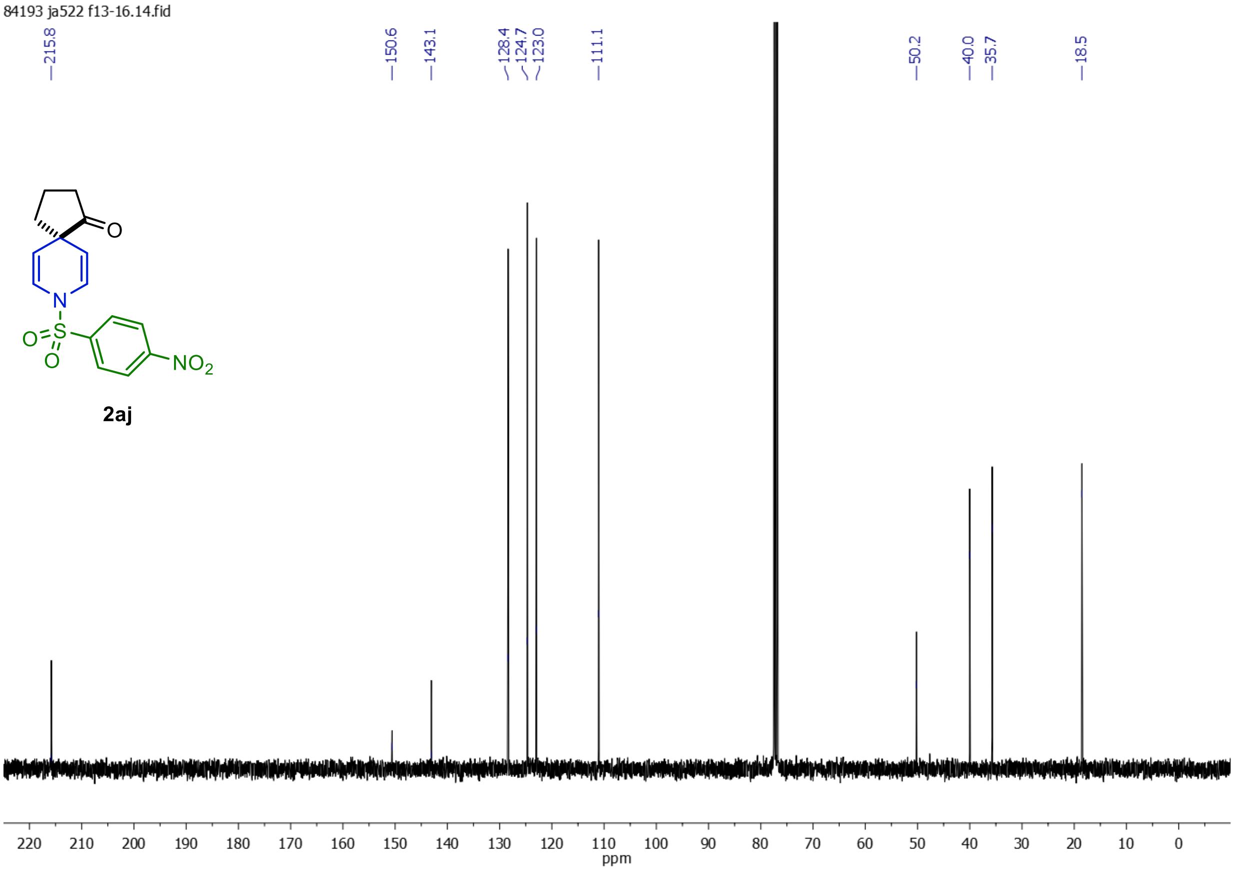

^{1}H NMR (400 MHz, $CDCl_3$) of **4a** (see procedure)

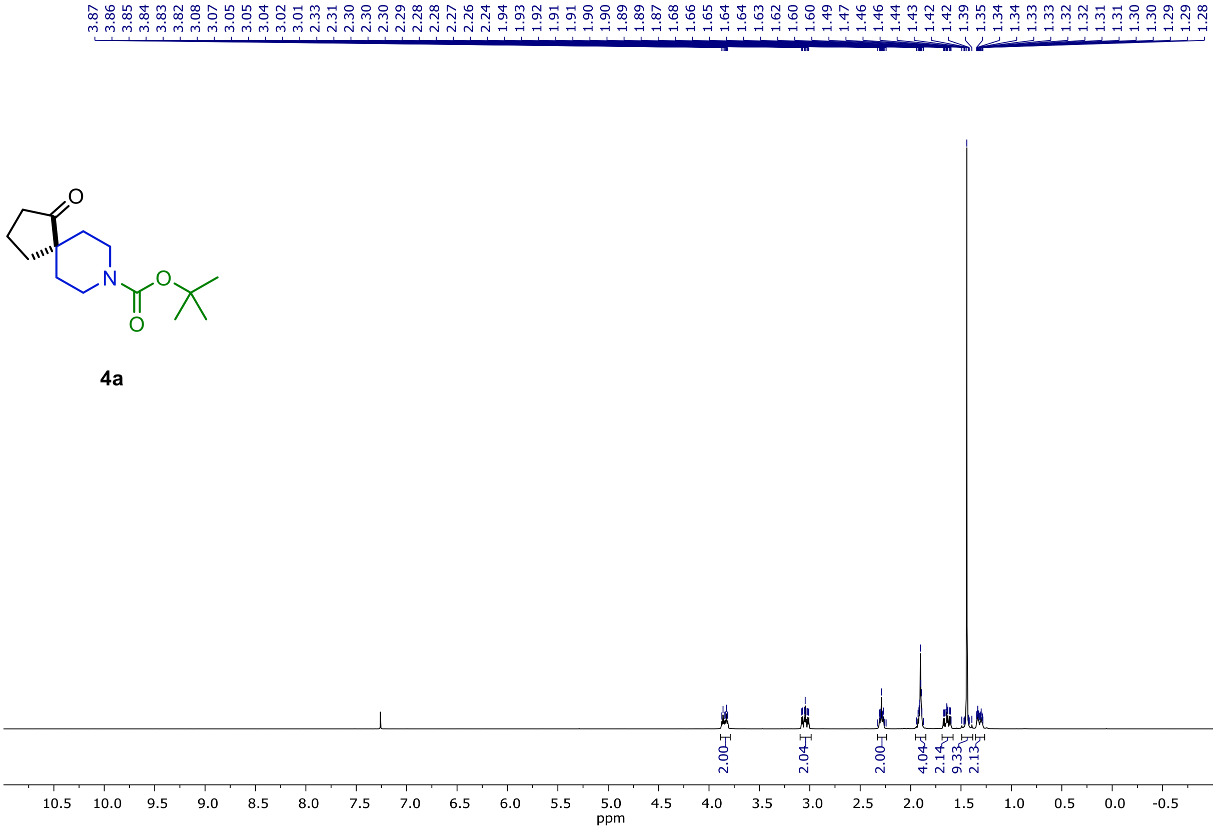

^{13}C NMR (101 MHz, $CDCl_3$) of **4a**

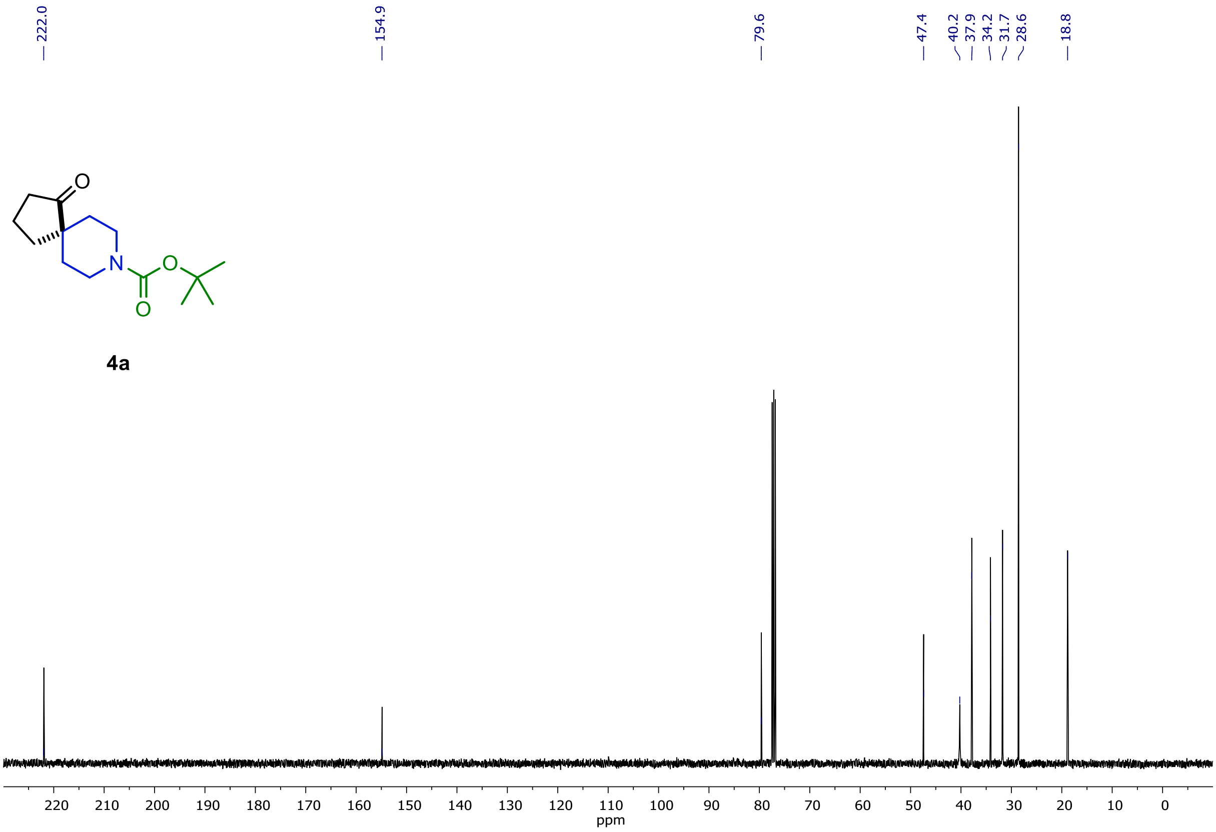

^{1}H NMR (400 MHz, $CDCl_3$) of **4b** (see procedure)

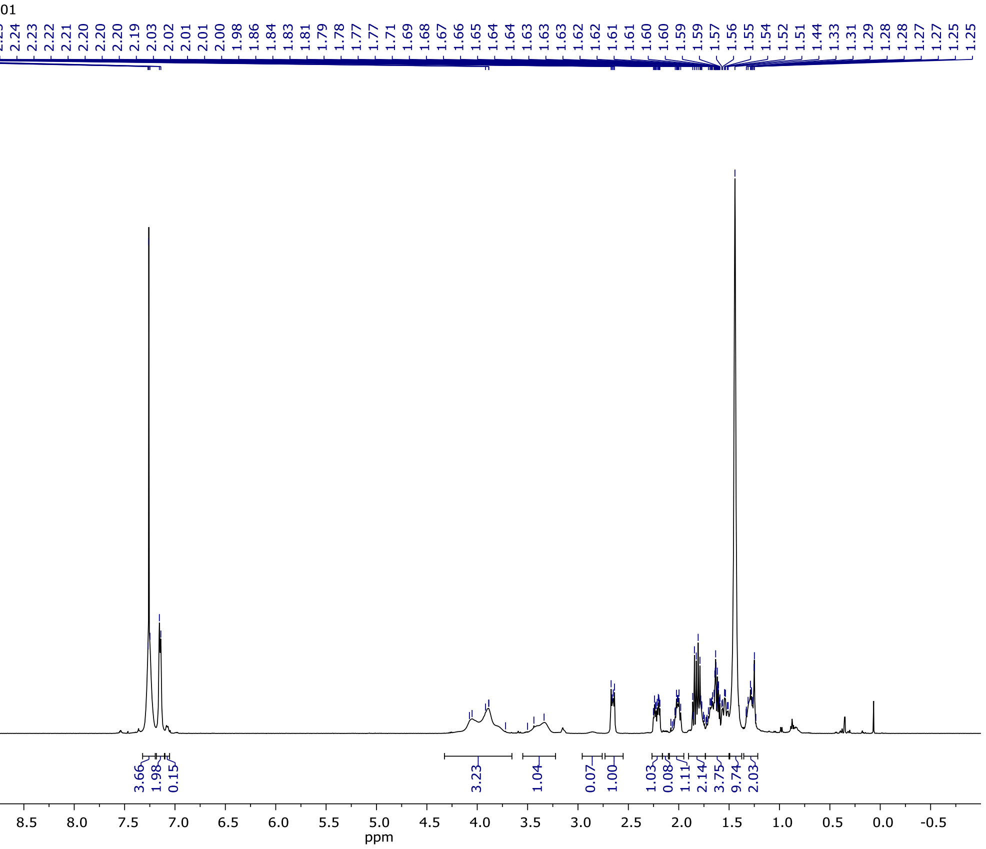

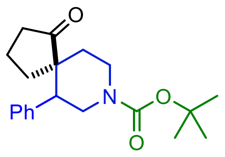

4b

^{13}C NMR (101 MHz, $CDCl_3$) of **4b**

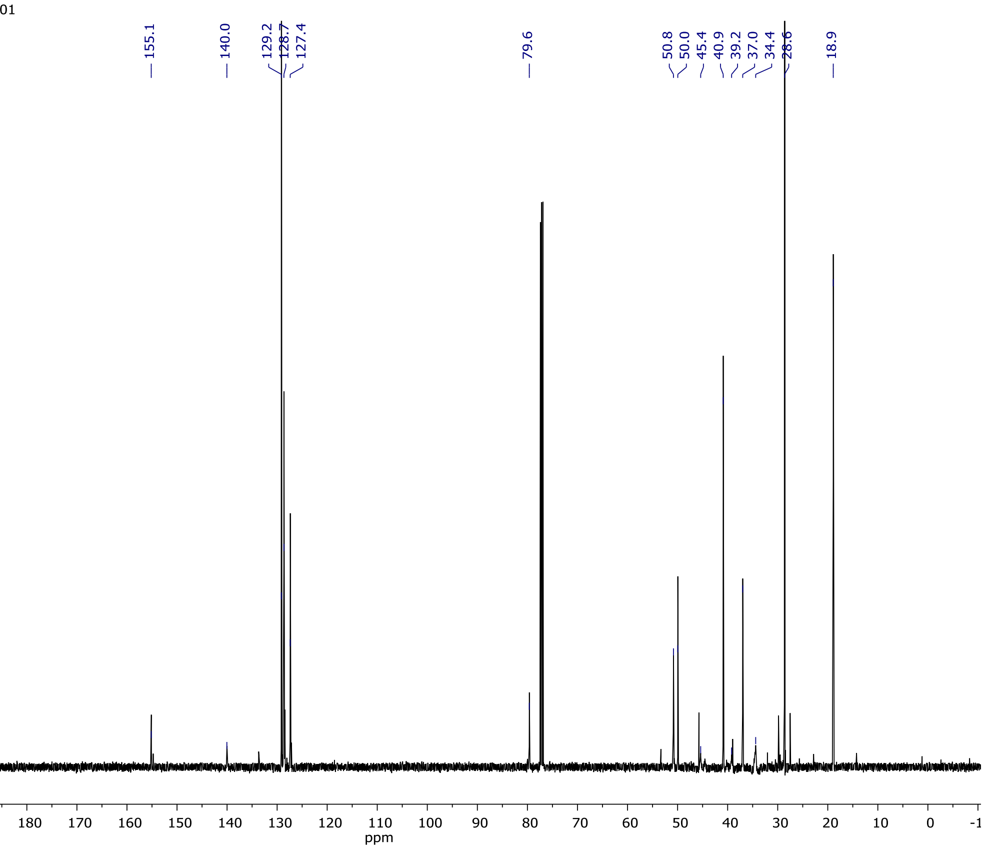

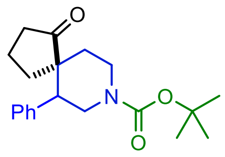

4b

5. REFERENCES

1) C. Wolf, G. E. Tumambac, C. N. Villalobos, *Synlett* **2003**, *12*, 1801-1804.

2) C. M. Poteat, Y. Jang, M. Jung, J. D. Johnson, R. G. Williams, V. N. G. Lindsay, *Angew. Chem. Int. Ed.* **2020**, *59*, 18655-18661.

3) J.-P. Deprés, A. E. Greene, P. Crabbé, *Tetrahedron* **1981**, *37*, 621-628.

4) J. D. Bender, P. A. Leber, R. R. Lirio, R. S. Smith, *J. Org. Chem.* **2000**, *65*, 5396-5402.

5) G. Karig, N. Thasana, T. Gallagher, *Synlett* **2002**, *5*, 808-810.

6) Q. Zhou, B. Zhang, L. Su, T. Jiang, R. Chen, T. Du, Y. Ye, J. Shen, G. Dai, D. Han, H. Jiang, *Tetrahedron* **2013**, *69*, 10996-11003.

7) Bruker, SAINT+ v8.39.0 Integration Engine, Data Reduction Software, Bruker Analytical X-ray Instruments Inc., Madison, WI, USA, **2018**.

8) Bruker, SADABS 2018, Bruker AXS area detector scaling and absorption correction, Bruker Analytical X-ray Instruments Inc., Madison, Wisconsin, USA, **2018**.

9) G. M. Sheldrick, *Acta Crystallogr. A: Found. Adv.* **2015**, *71*, 3-8.

10) G. M. Sheldrick, *Acta Crystallogr., Sect. A: Found. Crystallogr.* **2008**, *64*, 112-122.

11) G. M. Sheldrick, *Acta Crystallogr. C* **2015**, *71*, 3-8.

12) O. V. Dolomanov, L. J. Bourhis, R. J. Gildea, J. A. K. Howard, H. Puschmann, *J. Appl. Crystallogr.* **2009**, *42*, 339-341.
